# Poster presentations

**DOI:** 10.1054/bjoc.2000.1350

**Published:** 2000-08-10


British Journal of Cancer (2000) **83**(Suppl 1), 29–84
© 2000 Cancer Research Campaign
doi: 10.1054/ bjoc.2000.1350, available online at http://www.idealibrary.com on IDEAL®

### P1 ALTERED PATTERNS OF HEPARAN SULPHATE PROTEOGLYCAN EXPRESSION IN HUMAN EPITHELIAL OVARIAN CANCER

EJ Davies[1], A McGown[1], J Shanks[2], G David[3], JT Gallagher[4] and G Jayson[4], [1]Department of Drug Development and Imaging, Paterson Institute for Cancer Research, Manchester, UK, [2]Dept Histopathology, Christie Hospital, Manchester UK, [3]Centre for Human Genetics, University of Leuven, Belgium, [4]CRC Department of Medical Oncology, Christie Hospital, Manchester, UK

Heparan sulphate proteoglycans (HSPG) have been implicated in a number of processes related to the malignant phenotype, including malignant transformation, metastasis and angiogenesis. Changes in expression of HSPG have been reported in certain cancers, but most studies have only looked at syndecan 1 expression. The present study reports the immunohistochemical expression and cell localisation of syndecan 1, 2, 3 and 4, glypican and perlecan in human ovarian cancer using specific monoclonal antibodies and relates the findings to established prognostic factors. We performed immunostaining on formalin and frozen sections of normal ovary (n=9), benign ovarian tumours (n=15), borderline tumours (n=3) and epithelial ovarian cancer (n=90). Preliminary findings suggest altered expression and distribution of syndecan 1 in ovarian cancer tissue, which may suggest a role in metastasis. Scanning confocal microscopy suggests that syndecan 1 is present in the cytoplasm of epithelial cancer cells and this finding may represent internalisation or increased secretion of syndecan 1. Syndecan 4 also shows moderate cytoplasmic staining on light microscopy. The results indicate altered expression of HSPG in ovarian cancer, and increased intracellular localisation of syndecan 1 and syndecan 4. There is also intense reactivity for syndecan 3 and perlecan in tumour vessels, possibly suggesting a role in angiogenesis. This and the results of further studies including the prognostic significance of these changes by univariate and multivariate analysis will be presented.

### P2 THE INTERACTION OF $VEGF_{165}$ WITH HEPARAN SULPHATE

Chris Robinson*⁺, Sally Stringer*⁺ & John Gallagher⁺, The Cancer Research Campaign Department of Drug Development* and the CRC and University of Manchester Department of Medical Oncology⁺, Paterson Institute for Cancer Research, Christie Hospital, Manchester M20 4BX, UK

Vascular endothelial growth factor (VEGF) is a highly specific mitogen for cultured endothelial cells, and a potent stimulator of angiogenesis *in vivo*. The ability of the most abundant VEGF isoform, $VEGF_{165}$, to bind heparin and the related cell surface glycosaminoglycan heparan sulphate (HS) has been shown to play a significant role in the functioning of this growth factor. In light of the central role of VEGF in pathological conditions which are angiogenesis-dependent, notably the neovascularisation observed in growing tumours, the regulation of VEGF by heparin-like molecules is attracting much interest as a possible target for therapeutic intervention.

Heparan sulphate (HS) is a linear sulphated polysaccharide found covalently linked to core proteins in the extracellular matrix and on the surface of almost all mammalian cell types. It has a unique molecular structure consisting of short regions of high sulphation level (S-domains) interspersed by longer, predominantly non-sulphated *N*-acetyl-rich stretches. The complex structure of HS is the key to its biological activities as the regions of sulphation confer on this polymer the ability to bind specific protein ligands and through this binding modulate their functions.

We are studying the interaction of $VEGF_{165}$ with heparin/HS in order to characterise the sequence requirements within these polysaccharides that are responsible for their effects on VEGF function. Initially, Scatchard analysis of the $VEGF_{165}$-HS interaction revealed that binding occurs in a 1:1 ratio of $VEGF_{165}$ dimers to HS chains. Whilst in-solution $VEGF_{165}$-binding studies using specifically cleaved HS species showed that the interaction requires both highly sulphated ‘S-domain’ regions of the HS chains as well as the predominantly non-sulphated *N*-acetyl-rich sequences. These results support a model for the $VEGF_{165}$-HS interaction where S-domain regions interacting with the two heparin-binding sites of the $VEGF_{165}$ dimer are connected by a stretch of largely unsulphated sequence. Competitive inhibition studies and work with HS oligosaccharides suggest that the S-domain regions may have to be at least 8 monosaccharides in length and that 2-*O*-sulphation appears to be particularly important for the $VEGF_{165}$ interaction.

Currently, affinity chromatography and HPLC are being used to identify specific S-domain sequences capable of $VEGF_{165}$ binding and preliminary results appear to confirm the importance of 2-*O*-sulphation. Meanwhile, enzyme protection assays and oligosaccharide binding experiments are in progress to isolate the entire binding sequence from within HS. Attempts are also underway at transforming a HS-deficient cell line with the VEGF-receptor KDR to set up a biological activity assay system for potentially interesting HS fragments identified in these studies.

### P3 DIFFERENTIAL REGULATION OF VEGF FAMILY MEMBERS BY C-*ERB*B LIGANDS IN HEAD AND NECK SQUAMOUS CELL CARCINOMA (HNSCC)

P O-charoenrat*[1], P Rhys-Evans[1], and SA Eccles[2], [1]Royal Marsden Hospital, [2]Institute of Cancer Research, Sutton, Surrey SM2 5NG, UK

Aberrant expression of (proto)-oncogenic tyrosine kinases such as *c-erb*B receptors contributes to the progression of HNSCC. We have previously shown that EGFR/c-*erb*B activation potentiates invasion via upregulation of tumour cell motility and matrix metalloproteinase activity. However, in addition, upregulation of vascular endothelial growth factor-A (VEGF-A) expression via EGFR and/or c-*erb*B-2 signalling has been described in other tumour types. This raises the possibility that potentiation of angiogenesis might also contribute to the pathogenesis of HNSCC which overexpress members of the c-*erb*B family. Using semiquantitative RT-PCR, we found that all four known members of the VEGF family (VEGF-A, VEGF-B, VEGF-C and VEGF-D) were expressed in a panel of 15 HNSCC lines. We found a direct correlation between EGFR and VEGF expression levels in tissue specimens from HNSCC patients. To determine whether *erb*B activation was directly linked to VEGF expression we studied the effects of three major ligands with different selectivity of binding to c-*erb*B receptors; namely TGF-α, betacellulin (BTC) and heregulin-β1 (HRG-β1). All ligands upregulated all four isoforms of VEGF-A (most notably isoform 121) and also VEGF-C (BTC optimum concentrations 1–10 nM; TGF-α and HRG-β1 10–100 nM) but had no effect on VEGF-B. All ligands simultaneously down-regulated the expression of VEGF-D. An antagonistic EGFR antibody (ICR62) reduced the basal mRNA levels of VEGF-A (all isoforms) and VEGF-C but had no detectable effects on VEGF-B and VEGF-D. ICR62 also reversed the effects (whether stimulatory or inhibitory) of all three c-*erb*B ligands on VEGF-A, VEGF-C and VEGF-D expression. An anti-c-*erb*B-2 mAb (ICR12) had no effect on basal or ligand-modulated expression of any VEGF in these cell lines, although they co-express this receptor, which is a preferred heterodimerization partner for all receptors. The VEGF-C results are particularly interesting since this ligand has been implicated in lymphangiogenesis, and its expression correlates with lymph node metastasis in these HNSCC patients. Our results show that the four VEGF genes are regulated by c-*erb*B signalling pathways in a strikingly different manner, suggesting that they serve distinct, although perhaps complimentary (VEGF-A and VEGF-C) or antagonistic (VEGF-D) functions. The EGFR signalling pathway plays a major role in VEGF regulation in HNSCC, even in response to ligands which bind primarily (HRG-β1) or additionally (BTC) to other family members, notably c-*erb*B-4, presumably by forming active heterodimers.

### P4 IMMUNOHISTOCHEMICAL EXPRESSION OF VASCULAR ENDOTHELIAL GROWTH FACTOR (VEGF) AND VESSEL COUNTING AS PROGNOSTIC INDICATORS IN NODE-NEGATIVE COLORECTAL CANCER

E Tsiompanou, GM Boxer, T Levine & RHJ Begent, Oncology Dept, Royal Free Campus, UCL Medical School, London NW3 2PF, UK

Current tumour staging practices in colorectal cancer are proven inadequate to identify those patients with node-negative disease that will eventually relapse. New prognostic markers are needed to help us select high risk patients that will subsequently benefit from treatment at an earlier stage when there is minimal residual disease. The present study was designed to assess the VEGF expression and the microvascular count in node-negative colorectal cancers and to investigate the correlation of these two parameters with survival rate. Formalin-fixed, paraffin sections from 56 patients with primary colorectal carcinomas (12 Dukes A, 44 Dukes B) that have been completely excised, were immunostained for VEGF (R & D Systems) and CD34 (for blood vessel identification). VEGF reactivity was examined at or adjacent to the invasive margin and was graded on a scale of 0 to 3+. In addition, for each case, binding to vascular endothelium was noted. Vessel counts were also assessed at the invasive edge of the tumour, following the criteria of Weidner et al for assessing microvessel density in human tumour specimens[1]. Findings were correlated to survival in days and to outcome (dead/alive). Kaplan-Meier plots were generated and in Dukes B patients a statistically significant difference was demonstrated between VEGF+ve and VEGF–ve group (p=<0.05). The presence of VEGF reactivity was significantly correlated with patient outcome (p=<0.03) by Fishers Exact Test, as was the presence of VEGF reactivity within the vascular endothelium (p=0.018), both tests indicating that VEGF expression is an indicator of poor prognosis. The maximum vessel density per case, recorded for the Dukes B patient group (median=32.5, range 9–60,) was shown to be lower for those cases with a poor outcome (Mann Whitney U-test: p=0.019). These results suggest that assessment of VEGF expression by immunohistochemistry in Dukes B patients may be used to identify patients at risk of relapse. Moreover, using this assay it may be possible to select node-negative patients for therapeutic intervention, using treatment modalities such as chemotherapy or antibody-directed cancer therapy. Our results, which suggest that a low microvascular count is a marker of poor prognosis, are contrary to the findings of most other investigators. Further studies are required to establish the significance of this parameter. This work was supported by the Cancer Research Campaign.

1 Weidner N, Semple JP, Welch WR and Folkman J (1991) Tumour angiogenesis and metastasis-correlation in invasive breast carcinoma. *N Engl J Med* **324:** 1–8

### P5 A COMPARISON OF SERUM AND PLASMA LEVELS OF VASCULAR ENDOTHELIAL GROWTH FACTOR DURING THE MENSTRUAL CYCLE IN HEALTHY FEMALE VOLUNTEERS

C McIlhenny*, JC Doughty, WD George, University Department of Surgery, Western Infirmary, Glasgow G11 6NT, UK

**Aims** It has been suggested that serum levels of the angiogenic cytokine VEGF may alter with the menstrual cycle and be responsible for the reported increase in recurrence rate in breast cancer patients operated on during the follicular phase. However, it is now apparent that many haematological cells are capable of releasing VEGF, especially platelets. The extent to which these cells normally contribute to the circulating VEGF pool in vivo and the physiological relevance of this is not known. The measurement of serum concentrations of VEGF has therefore been speculated upon as being an artificial situation, which measures both the freely circulating VEGF and that released by platelets upon activation during the clotting process. We therefore conducted the first prospective trial to compare serum and plasma levels of VEGF in healthy pre-menopausal volunteers.

**Methods** 20 healthy premenopausal women were recruited and had blood samples were taken over one menstrual cycle with an average of 8 samples taken per patient. Plasma and serum samples were then analysed for sex hormones and VEGF 165.

**Results** Serum VEGF levels were found to be significantly higher than plasma VEGF levels ($p<0.005$). The majority of the measurements for plasma levels of VEGF at all phases of the cycle were under the limit of detection of the VEGF ELISA kit. We found no significant difference between serum and plasma VEGF in the luteal and follicular phases of the cycle. We found no significant correlation between plasma or serum levels of VEGF and either FSH, LH, Oestradiol or Progesterone levels.

**Discussion** We conclude that varying levels of serum VEGF are not responsible for any possible survival advantage of being operated on for breast cancer during the follicular phase of the cycle. Plasma VEGF levels are much lower than serum levels and are usually beneath the limit of the ELISA assay utilised in our study. Development of a more sensitive assay is required before conclusions can be reached about its physiological significance. In addition, with no cyclical variation in circulating VEGF demonstrated, menstrual phase need not be taken into account if serum or plasma VEGF is to be used as a prognostic factor in the future.

### P6 IMAGING VASCULAR ENDOTHELIAL GROWTH FACTOR *IN VIVO* WITH POSITRON EMISSION TOMOGRAPHY

VA Carroll[1,2], M Glaser[1], E Aboagye[1], D Brown[1], S Luthra[1], F Brady[1], R Bicknell[2], AL Harris[2] and P Price[1], [1]CRC PET Research Group, Hammersmith Hospital, ICSM, London and [2]ICRF Molecular Oncology labs, Institute of Molecular Medicine, University of Oxford, Oxford, UK

Tumour growth and spread is dependent on angiogenesis – defined as the growth of new blood vessels from the pre-existing vasculature. Blocking antibodies to vascular endothelial growth factor, an important angiogenic factor are currently in phase II/III clinical trials for anti-angiogenic therapy. We are developing quantitative assays to image expression of the target molecule VEGF *in vivo* by positron emission tomography (PET), initially in animal models.

An anti-VEGF monoclonal antibody denoted VG76e was shown to completely inhibit VEGF stimulated endothelial cell growth. Iodine 124 was chosen as a suitable positron emitter due to its long half-life ($t_{1/2}$=4days) and was generated by the 124Te (p,n) reaction. VG76e was radiolabelled with $^{124}$I both directly by the iodogen technique or via lysine residues with 'Bolton-Hunter' labelling reagents. Following iodination, immunoreactivity was analysed by binding to VEGF in a radioimmunoassay. Immunoreactivity of VG76e was less than 10% after direct labelling with iodogen, but increased to 45% when conjugated to iodinated succinimidyl esters. HT-1080 fibrosarcoma cells that secrete high levels of VEGF and are known to form secondary lung tumours were injected sc into nude mice. Once tumours had reached a diameter of 0.7 mm $^{124}$I-VG76 was injected intravenously and mice were scanned 24 h later in a HIDAC animal scanner. PET images showed localisation to the tumour as well as the thoracic cavity. Tumour and normal tissue including heart, lung, liver, kidney, intestine and spleen were excised and radioactivity was determined by gamma counting. Ex vivo biodistribution confirmed antibody localisation to the tumour (5.9% id/g) and lung (3.5% id/g). PET may be one useful strategy for effective monitoring of antiangiogenic therapy with VG76e *in vivo* and in addition, to assess the role of VEGF in the growth of secondary cancer.

### P7 A POSITRON EMISSION TOMOGRAPHY (PET) STUDY ON THE EFFECTS OF COMBRETASTATIN A4 ON MURINE LIVER METASTASES

AT McGown[1], JV Moore[1], S Zhao[1], JA Hadfield[1], DL Hastings[2], ML Waller[2] and GR Pettit[3], [1]CRC Paterson Institute, [2]Christie Hospital, Manchester M204BX, [3]Arizona State University, USA

The combretastatins have been shown to be capable of causing the destruction of tumour vasculature in model systems (Dark et al., (1997), BJC, **77**, 1829; Grosios et al., (1999), BJC, **81**, 1318). We have used positron emission tomography (PET) as a non-invasive measurement of tumour biology and therapeutic response in liver metastases (murine T50/80 breast) following treatment with combretastatin A4. This has allowed us to study the outcome of therapy in individual animals in a clinically relevant internal body site. [18F]-Fluorodeoxyglucose ([18F]-FDG) was used as an indicator of metabolism and scanning was carried out using high-density avalanche chamber (HIDAC) detection. The distribution of [18F]-FDG in the absence of disease was characterised, showing clear delineation of the brain, heart, and urinary bladder. In untreated mice with liver metastases a strong correlation ($r^2$=0.98) was found between the estimation of [18F]-FDG by PET and *ex vivo* counting. The spatial resolution of the instrument (2.6 mm FWHM) allowed us to distinguish total metastatic tumour masses of 200 mm$^3$, although smaller individual deposits could be seen.

Single i.p. treatment with combretastatin A4 (75–140 mg/kg) resulted in an average 30% decrease in tumour volume 24 hours following administration. A histological examination of these tumours showed extensive necrosis consistent with vascular damage. This technology is now being used to study other combretastatin analogues. In conclusion PET imaging with [18F]-FDG provides a non-invasive assay of cellular metabolism. The ability to sequentially scanned the same animal both before and after treatment, and to study the effects of therapy in clinically relevant internal sites makes this extremely powerful tool in the development of novel anticancer therapies. This has been used to demonstrate the anti-vascular effect of combretastatin A4 on metastatic deposits of the liver.

### P8 DESIGN AND SYNTHESIS OF NOVEL COMBRETASTATIN ANALOGUES

K Gaukroger*[1,3], JA Hadfield[1,2], NJ Lawrence[3] and AT McGown[1], [1]CRC Drug Development Group, [2]CRC Radiochemical Targeting and Imaging, Paterson Institute for Cancer Research, Christie Hospital NHS Trust, Manchester M20 4BX, [3]Department of Chemistry, UMIST, PO Box 88, Sackville Street, Manchester M60 1QD, UK

The combretastatins are stilbenes isolated from the South African tree *Combretum caffrum*. These agents are characterised for their ability to interact with tubulin and cause catastrophic destruction of blood vessels (vasculature) within tumours. Tumours are thus deprived of oxygen and nutrients, leading to death of the cancer cells. The simplicity of the combretastatins offers promise for the rational design of new chemotherapeutic agents and we are currently studying the structure-activity relationship of substituted stilbene derivatives of this type based on the lead compound, combretastatin A-4. The main synthetic approaches to combretastatin A-4 and analogues are based on Witting reactions from which both *E* and *Z* stereoisomers are isolated. A series of novel analogues with substitution both in the A and B rings of combretastatin have been synthesised. We are also investigating the effect of functionalising the olefinic bond. The Heck reaction has been utilised to synthesis stilbenes with a methyl group on the olefinic bond. These modified stilbenes have been characterised by their ability to inhibit the growth of P388 and K562 cells *in vitro*. This study has shown that altering the trimethoxy aryl motif on the A ring and changing the configuration of the olefinic bond of the stilbene from *Z* to *E* leads to a large loss in cytotoxic activity. The ability of these compounds to interact with tubulin and to disrupt the microvasculature is currently being studied. These data are being used to design new analogues with improved antivascular activity.

The funding for this project comes from the Association for International Cancer Research.

### P9 *IN VITRO* CHARACTERIZATION OF THE NOVEL TUMOUR VASCULAR TARGETING AGENT ZD6126

AL Holder[1], GM Tozer[1], C Kanthou[1], PD Davis*[2], S Douglas[3] and D Blakey[3], [1]Gray Laboratory CRT, Northwood, HA6 2JR, [2]Angiogene Pharmaceuticals Ltd, [1]Oxfordshire, OX9 5SX, [3]AstraZeneca plc, Macclesfield, SK10 4TG, UK

Certain tubulin-binding agents such as colchicine and vinca alkaloids disrupt tumour vasculature at doses approaching their maximum tolerated dose. ZD6126, a phosphate prodrug of N-acetylcolchinol, is a new tubulin binding agent which, unlike colchicine and the vinca alkaloids, has selective tumour vascular damaging activity at well tolerated doses. These studies aimed to help characterise the properties of ZD6126 (ANG453) responsible for its *in vivo* activity using *in vitro* assays to measure toxicity/antiproliferation, permeability changes to cell monolayers and cell shape changes.

The toxicity/antiproliferative effects were measured in proliferating human endothelial cells (HUVECs), which were drug treated for 2 hours, washed, given fresh medium and then incubated for a further 4 days. Viability was then assessed by neutral red staining. For permeability studies, HUVECs were plated on to fibronectin coated microporous filter cell culture inserts to give a confluent monolayer. The passage of FITC-Dextran was then measured through both treated and untreated cell monolayers and unseeded inserts to enable a % change in permeability to be calculated. Immunofluorescence studies were performed using HUVECs stained with an anti β-tubulin antibody followed by an FITC-linked conjugate in order to evaluate effects on the microtubule cytoskeleton and changes in cell shape.

The toxicity/antiproliferative effects of both N-acetylcolchinol and ZD6126 in HUVECs were found to occur at concentrations >100 μM. In contrast, changes to the microtubule cytoskeleton leading to changes in cell shape and permeability were observed at concentrations much lower than those causing toxicity. Depolymerization of the microtubules and increased permeability (20–30%) of cell monolayers were seen at 0.01 μM ZD6126. These results suggest that, at concentrations of active drug which cause anti-vascular activity *in vivo*, change in endothelial cell shape and function, rather than direct endothelial cell cytoxicity, are likely to be responsible for the selective anti-vascular activity of ZD6126.

### P10 ZD6126: A NEW AGENT CAUSING SELECTIVE DAMAGE OF TUMOUR VASCULATURE

Davis PD*[1], Hill SA[2], Galbraith SM[2], Chaplin DJ[2], Naylor MA[2], Nolan J[2] and Dougherty GJ[1], [1]Angiogene Pharmaceuticals Ltd., Oxfordshire OX9 5SX, UK and [2]Gray Laboratory Cancer Research Trust, Northwood HA6 2JR, UK

Tubulin-binding agents such as colchicine, vinca alkaloids and combretastatins disrupt tumour vasculature by a direct effect on immature endothelium. Only the combretastatins, however, have been reported to have significant vascular damaging activity below maximum tolerated doses. We have developed a new class of tubulin binding agents with selective tumour vascular damaging activity at well tolerated doses. ZD6126 (ANG453), a phosphate prodrug of N-acetylcolchinol, is synthesised in four steps from colchicine. Treatment of non-confluent, proliferating HuVEC cultures (as a model of immature endothelium) with ZD6126 produces changes in cell shape at concentrations that are without effect on confluent, quiescent cultures. At one thirtieth of the maximum tolerated dose ZD6126 causes marked reduction in the functional vascular volume of CaNT tumours in CBA mice, 6 h after drug administration. Widespread necrosis is induced in these tumours, leaving only a rim of viable cells. ZD6126 at well tolerated doses has significant anti-tumour activity in both syngeneic tumour (CaNT) and xenograft (FaDu) models.

### P11 ANTI-TUMOUR ACTIVITY OF ZD6474, A VEGF RECEPTOR TYROSINE KINASE INHIBITOR

J Kendrew[1], SR Wedge[1], DJ Ogilvie[1], M Dukes[1], LF Hennequin[2], ESE Stokes[1] and B Curry[1], [1]Cancer and Infection Research, AstraZeneca Pharmaceuticals, Alderley Park, Macclesfield, Cheshire SK10 4TG, UK; and [2]AstraZeneca Pharma, Centre de Recherches, Z.I. La Pompelle, B.P. 401, 51064 Reims, France

ZD4190, a small molecular weight VEGF receptor tyrosine kinase inhibitor (RTKI), has previously been shown to elicit broad-spectrum anti-tumour activity in human tumour xenografts, following chronic oral daily dosing (Wedge *et al.*, Cancer Res, *60*: 970–975, 2000). ZD6474, another VEGF RTKI, also demonstrates significant activity in a histologically diverse panel of human tumour models (lung, breast, ovarian, vulval, prostate, colon). This profile is consistent with inhibition of VEGF signalling and an indirect (i.e. anti-angiogenic) anti-tumour effect.

The effect of ZD6474 treatment on established tumours of varying sizes was investigated. ZD6474 (100 mg/kg/day, p.o.) was administered to groups of mice bearing CaLu-6 human NSCLC tumours, with mean volumes of 0.3, 0.5, 1.0 and 1.5 $cm^3$ (n = 10). ZD6474 was found to inhibit the growth of all tumours, irrespective of the tumour volume at start of treatment. A similar experiment was performed with mice bearing different sized PC-3 human prostate tumours (mean volumes of 0.3, 0.5, 0.9 and 1.4 $cm^3$). In contrast to the responses observed in the CaLu-6 model (growth-inhibition), ZD6474 was found to induce *regression* in all PC-3 tumours with the most pronounced effects being evident in the largest tumours.

The PC-3 tumour model was further used to examine the effect of intermittent ZD6474 treatment (50 mg/kg/day, p.o.). Mice bearing PC-3 tumours were treated with ZD6474 for 2 weeks. Treatment was then withdrawn and tumours allowed to re-grow for 4 weeks until reaching a volume of ~ 0.7 $cm^3$. Upon re-treatment with ZD6474 for 4 weeks, all tumours were observed to regress to a volume of ~ 0.2 $cm^3$. This study indicates that human tumour xenografts remain responsive to re-treatment with an inhibitor of VEGF signal transduction.

### P12 A PHASE II STUDY OF HIGH DOSE ORAL THALIDOMIDE IN PATIENTS WITH METASTATIC RENAL CELL CARCINOMA (RCC) OR METASTATIC MELANOMA (MM)

J Stebbing[1], C Benson[1]*, T Eisen[2], I Mak[3], L Pyle[1], K Smalley[2], ME Gore[1], [1]Dept. of Medical Oncology, Royal Marsden Hospital, London SW3 6JJ, [2]Dept of Oncology, Royal Free & University College Medical School, London W1P 8BT, [3] Dept of Clinical Neurophysiology, Chelsea & Westminster Hospital, London SW10 9NH, UK

Angiogenesis is an important new target for anti-cancer drugs. Thalidomide suppresses levels of the angiogenic factors, tumour necrosis factor-α (TNFα) and basic fibroblast growth factor (bFGF). We have performed a phase II study in previously treated patients with metastatic RCC (20 patients) and MM (19 patients). The median age of patients (pts) was 51 years, range 26–74 years. All pts had pre-treatment sensory nerve action potential (SNAP) testing, repeated every 3 months. We also measured levels of TNFα, bFGF, vascular endothelial growth factor (VEGF), IL-6 and IL-12, before and during treatment. Pts were treated with escalating doses of thalidomide over 6 weeks to a target dose of 600 mg po daily.

Results: The response rate in pts with metastatic RCC was 10% (2 pts). However, an additional 10 pts achieved stable disease (SD) ≥ 12 weeks, 7 of these had SD ≥ 24 weeks. There were no objective responses in pts with MM although 1 patient achieved SD ≥ 12 weeks. Tumours were heterogenous and mean cytokine levels did not correlate with response to treatment. Further analysis is awaited. Main toxicities (WHO) were lethargy (13 pts grade II, 2 pts grade III), constipation (10 pts grade II, 2 pts grade III) and neuropathy (3 pts grade II, 1 pt grade III). In pts with RCC, 17/20 reached 600 mg od and only 4 required dose reductions due to toxicity. Conclusions: Thalidomide has activity in patients with metastatic RCC. We are currently engaged in a phase III study comparing its efficacy against medroxyprogesterone acetate.

### P13

WITHDRAWN

### P14 LOCALISATION OF HYPOXIC CELLS USING A POLYCLONAL ANTISERUM WHICH RECOGNISES PROTEIN ADDUCTS OF THE 2-NITROIMIDAZOLE PROBE SR4554

Anthea Hardcastle, Sue Clinton, Phyllis Goddard, Lisa Brunton, Melanie Valenti, Paul Workman and G Wynne Aherne, CRC Centre for Cancer Therapeutics, Institute of Cancer Research, Sutton, Surrey SM2 5NG, UK

Hypoxia within solid malignant tumours causes lack of response to both radiotherapy and conventional chemotherapy. Additionally it is associated with the development of metastatic disease. Detection of hypoxia would therefore be an important advance in the treatment of some cancers. SR4554 N-(2-hydroxy-3,3,3,-trifluoropropyl)-2-2-(2-nitroimidazoyl) acetamide, a new non-invasive probe for hypoxia, undergoes selective nitroreduction and forms adducts to macromolecules in hypoxic cells that can be detected by Magnetic Resonance Spectroscopy (MRS). It has been approved for Phase I trial as a tumour hypoxia probe. Immunochemical detection of SR4554 adducts would provide an additional methodology for validation of the compound as a hypoxia marker.

Ten rabbits were immunised with a variety of SR4554-protein (bovine serum albumin, Bowman's Birk Inhibitor and ovalbumin) conjugates prepared by different methodologies under radioreduction. Antisera production was monitored by a sensitive antigen coated enzyme linked immunosorbent assay (ELISA) with time resolved fluorescent end point detection (Delfia). The ELISA was also used for preliminary characterisation of the antisera. The SR4554 conjugate used for coating in the ELISA was different from that used for immunisation. All animals showed an immune response in this screen by binding to the coated conjugate. This indicates that the animals had produced antibodies that recognise an epitope on SR4554 protein conjugates which is not the protein moiety of the conjugate used as immunogen. This is possibly an SR4554 adduct since competitive displacement experiments showed that the antisera did not recognise unconjugated SR4554.

Nude mice carrying xenografts of human tumours (CH1, SKOV3, BE, HN5, HT29 and COLO26) were dosed with SR4554 (80 mg/kg ip) and tumour obtained at various timepoints. Tumours from undosed animals were used as controls. The antiserum showing the best titre in the ELISA has been used for immunohistochemical detection of SR4554 adducts in sections cut from these tumours using a biotinylated secondary antibody followed by a preformed avidin/biotin/horse radish peroxidase complex and subsequent visualisation with diaminobenzidine (ABC/DAB Vector Laboratories). The same sections have been stained for platelet/endothelial cell adhesion molecule-1 (Pecam 1/CD31) and Ki67 antigen (Mib1) to co-localise tumour angiogenesis and proliferation with the presence of SR4554 adducts. Compared to untreated controls, areas of brown immunoperoxidase staining of varying intensity were visible in the tumours. These reagents in conjunction with other techniques are presently being used to validate the use of SR4554 as a marker of hypoxia in ongoing preclinical studies and in the forthcoming Phase 1 trial.
Supported by the Cancer Research Campaign Programme Grant SP2330/0201.

### P15 BLOOD OXYGEN LEVEL DEPENDENT (BOLD) MRI IMAGING: A NON INVASIVE ASSESSMENT OF HYPOXIA AND PERFUSION IN SOLID TUMOURS

N Shah*[1], P Hoskin[1], A Sibtain[1], H Baddeley[2], J Taylor[2], MI Saunders[2], Marie Curie Research Wing & The Paul Strickland Scanner Centre, Mount Vernon Hospital, Northwood HA6 2RN, UK

**Purpose** Non invasive MRI evaluation of changes in tumour oxygenation using carbogen (5% $CO_2$, 95%$O_2$) breathing to enhance oxygen uptake *in vivo*.

**Background** Gradient recalled echo (GRE) MRI imaging can assess blood flow changes *in vivo*. MRI can utilise the respective paramagnetic and diamagnetic properties of tissue deoxyhaemoglobin and oxyhaemoglobin providing a functional sequence of blood oxygen level dependent (BOLD) contrast scans to study the modification of tissue hypoxia.

**Methods** Subjects underwent GRE MRI for BOLD enhancement whilst breathing a sequence of air (5 minutes), carbogen (10 minutes) and air (10 minutes). Specified regions of interest in solid tumours and normal tissues were analysed pre and post carbogen breathing. A BOLD signal change of more than 5% of the whole region was interpreted as a significant change due to carbogen breathing.

**Results** 35 patients with solid tumours (pre-treatment) were successfully assessed. In 15 other subjects, technical difficulties including patient movement, intolerance of carbogen, mask displacement and magnetic disruption artefacts did not allow a full assessment. Patients with head and neck tumours constituted approximately 50% of the technical failures.

BOLD enhancement during carbogen breathing was observed in 20 patients (57%). By histological subtype these included 6/8 SCC head and neck, 2/4 NHL, 1/6 rectal carcinoma, 5/8 bladder carcinoma, 2/3 prostate carcinoma and 4 other sites (sacral osteosarcoma, buttock melanoma, breast carcinoma and prostatic leiomyosarcoma). A reduction in signal was observed in 1 patient with a head and neck carcinoma.

**Conclusion** Changes in BOLD MRI sequences have been observed in 57% of patients during carbogen breathing supporting other work using direct measurements which demonstrate an increase in tumour oxygenation. The relative components of perfusion change and oxygenation cannot be evaluated. Modifications in the gas mixture and breathing apparatus are being assessed to reduce technical failure. Studies are necessary to correlate the above findings with clinical outcome and the suitability of this technique for assessing therapies modifying hypoxia.

### P16 RADIOIMMUNOTHERAPY IS MORE EFFECTIVE IN SMALL TUMOURS DUE TO HIGHER DOSE AND DOSE RATE

A Mayer*[1], AA Flynn[2], E Tsiompanou[2], RB Pedley[2], J Dearling[2], R Boden[2], RHJ Begent[2], [1]Oncology Centre, Addenbrooke's NHS Trust, Cambridge CB2 2QQ, [2]Cancer Research Campaign Targeting and Imaging Group, Royal Free and University College Medical School, London NW3 2PF, UK

We report an experiment to investigate the potential reasons for the enhanced therapeutic effect of radioimmunotherapy (RIT) in small tumours. Theoretically a greater proportion of viable radiosensitive areas in small tumours, higher antibody uptake or a higher dose and dose rate may account for this.

Six mice per group bearing small (median tumour size 0.06 $cm^3$, range 0.02–0.11 $cm^3$) and large LoVo xenografts (median tumour size 0.38 $cm^3$, range 0.16–0.57 $cm^3$) received either RIT using a $^{131}I$ labelled anti-CEA antibody A5B7, 5-FU modulated with leucovorin (LV) or no treatment at all. Tumour size was measured for 22 days. The %injected activity/gram was determined by a laboratory gamma counter in a separate study. Radioluminography was used to assess the distribution of the antibody in viable and necrotic areas as defined on the corresponding digitised histological section. Beta point dose kernels were then used to generate the dose distribution from each radioluminograph. High power microscopy images of small and large tumours were reconstructed to form a mosaic of the original section to estimate the proportion of viable areas in the tumour volume.

Mice with large (p=0.002) and small (p=0.0003) xenografts grew significantly less when treated with RIT compared to the control group. 5-FU was found ineffective in this experiment. Comparison of the groups with small and large tumours treated with RIT showed that small tumours grew significantly less compared to large tumours (p<0.02), while there was no significant difference of small and large tumour growth in the group treated with 5-FU and the control group. The total amount of radioactivity in small and large tumours was not significantly different, but a significantly higher amount of % injected activity per gram of tumour was found in small tumours (median 26.6%, range 19.9–59.2% vs median 8.1%, range 1.6–12.5%, p=0.0007). In addition, a significantly greater % injected activity per gram was found in both viable (47.7% vs 11.1%, p=0.007) and necrotic (18.8% vs 3.9%, p=0.006) parts of small tumours compared to large tumours. Consequently, a significantly higher dose rate at 24 hours post injection was calculated for viable (56.2+/–23.7 vs 13.3+/–7 cGy/h) and necrotic parts (19.2+/–16.3 vs 4.9+/–4.7 cGy/h) of small tumours compared to large tumours (p=0.0007). Mosaics of images of tumour morphology showed that a significantly larger % of the total volume was viable for larger tumours (85.2% vs 71.6%, p=0.039).

Our experiment confirms that RIT is more effective in small tumours due to higher dose and dose rate throughout the tumour. The improved tumour control rate in small tumours favours RIT as an attractive option for adjuvant treatment in colon cancer.

### P17 OPTIMISING CORONARY ARTERY RADIATION USING TARGETED RADIOIMMUNOTHERAPY, 

EC Sims[1]*, MEB Powell[2], MT Rothman[3], TD Warner[1], [1]Dept of Vascular Inflammation, William Harvey Research Institute, St. Bartholomew's & The Royal London School of Medicine and Dentistry, [2]Dept of Clinical Oncology & [3]Dept of Cardiology, Bart's & The London NHS Trust, UK

**Introduction** Coronary artery disease is the leading cause of mortality in the West with over 1.2 million angioplasties performed annually. Despite the introduction of stents, restenosis occurs in 20–35% of vessels. Intracoronary artery radiation has emerged as potential therapy for the prevention of restenosis. Targeted radiation using radiolabelled antibody would optimise delivery and fractionation of radiotherapy and minimise radioprotection issues.

**Aims** To assess the feasibility of coating a stent with antigen and to evaluate the qualitative and quantitative uptake of antigen specific antibody *in vitro* and *in vivo*.

**Method** Lengths of polythene tubing (similar to coated intracoronary stents) were incubated in either biotinylated bovine serum albumin (experimental arm) or phosphate buffered saline (control). A circuit mimicking the circulatory system was devised. It incorporated the tubes (vessels), a peristaltic pump (heart) and a 20 ml reservoir of heparinised human blood containing fluorescein-tagged antibiotin antibody (5 µg/ml). After 24 hours in this circuit the tubes were incubated with trypsin to recover bound antibiotin antibody. Uptake of antibiotin antibody was then quantified using curves created from fluorescence measurement of standard pre-prepared antibody dilutions in 0.5% trypsin.

**Results** Uptake of anti-biotin antibody by tubes is shown in the table. The reading of 14050 was estimated to equate to antibody uptake of 3.7 µg per tube.

| Antigen | Fluorescence reading | Standard error |
|---|---|---|
| Tube + Biotin-BSA (n=4) | 14050 | ±469 |
| Tube + PBS (control) (n=4) | 147 | ±8 |
| Assay Background (0.5% trypsin) (n=4) | 145 | ±6 |

**Conclusion** Polythene tubing can be readily coated with antigen. Using an *in vitro* flow system antibody may be specifically targeted to this antigen. No uptake of labelled antibody to tubes lacking antigen occurred. Further results using an *in vivo* system will be presented.

These results show for the first time that highly selective targeted radioimmunotherapy in vascular brachytherapy using radio-labelled antibody has important therapeutic potential.

### P18 PATTERN OF LOCAL RECURRENCE AFTER CONSERVATIVE SURGERY AND RADIOTHERAPY FOR SOFT TISSUE SARCOMA 

Susan Cleator[1]*, Chris Cottrill[2], Clive Harmer[1], [1]Sarcoma Unit, Royal Marsden Hospital NHS Trust, London SW3 6JJ, [2]Dept of Radiotherapy, St Bartholomew's Hospital, London EC1A 7BE, UK

**Purpose** Over the past two decades our centre has adopted a policy of conservative surgery followed by adjuvant radical-dose radiotherapy for all medium- and high-grade soft tissue sarcomas. Low grade tumours are treated post-operatively only if they demonstrate multiple recurrences or if complete resection is not technically feasible. From the patients treated in this manner at the Royal Marsden Hospital over the last 15 years we studied 16 who had relapsed locally. We discuss technical factors which may have contributed to local relapse in some cases and the geographical relationship between sites of recurrence and the phase 1 and 2 radiotherapy volumes.

**Patients and methods** We examined those cases recorded on our soft tissue sarcoma database between January 1986 and September 1999 who had recurred locally following surgery and radical post-operative radiotherapy. We excluded patients with residual macroscopic disease following surgery and any patients with metastatic disease at presentation. We recorded details relating to original tumour stage, histological type, grade and surgical margins. The cohort included 7 patients with T1 (44%) and 9 patients with T2 tumours (56%). The tumours were grade 1, 2 and 3 in 2 (13%), 5 (31%) and 9 (56%) cases respectively. The majority of patients were treated with a phase I volume corresponding to the entire muscle compartment which received 50 Gy in 25 fractions over 5 weeks. The phase II was a reduced volume corresponding to the tumour bed and received 10 Gy in 5 fractions during the sixth week of treatment.

**Results** Mean time to local recurrence was 26 months. Four recurred within the phase I volume (25%), 8 (50%) recurred within the phase II volume and 4 (25%) outside the irradiated volume. Seven patients who relapsed had positive surgical excision margins originally; of these, 2 recurred in the phase I volume, 4 in the phase II and I outside the irradiated volume. Nine patients had clear operative margins originally; of these, 2 recurred within the phase I, 4 in phase II and 3 outside the field. With a mean follow-up time since completion of radiotherapy of 63 months, 7 patients have died, of whom 6 were disease-related.

**Conclusion** Prospective, multi-centre data collection and ideally a randomised trial are required to formulate an improved treatment policy.

**Keywords:** ***sarcoma, post-operative radiotherapy, recurrence, conservative surgery***.

### P19 RADIOTHERAPY TO LIMB PROSTHESES: A NEW METHOD TO IMPROVE DOSE HOMOGENEITY 

Dr KE Sherwin*[1], R Kestelman[2], SJ Thomas[2], AR Geater[2], Dr NG Burnet, Dr CB Wilson[1], Dr MV Williams[1], Departments of [1]Oncology and [2]Medical Physics, Addenbrooke's Hospital, Cambridge CB2 2QQ, UK

A metal prosthesis within the target volume adversely affects the homogeneity of the radiotherapy dose distribution. In rare cases when radiotherapy is required after prosthetic replacement of a bone tumour, a significant shadow is cast by the prosthesis.

We report a novel technique devised in our department to deliver radiotherapy to a limb and its metal bone prosthesis to homogenise dose distribution and minimise the risk of underdosage in the 'shadow' of the prosthesis. Radiotherapy is delivered using parallel opposed fields to the limb with sparing of a lymphovascular corridor and the area in the shadow of the prosthesis boosted using a 'negative' keyhole cutout of the prosthesis. To date we have treated four patients with this technique, with no local failures, median follow-up 2 years.

Standard dosimetry calculations use an estimate of the electron density of titanium alloy to be four times that of tissue. Using a 'Modified Batho' algorithm the treatment planning system suggested a 10% dose deficit either side of the prosthesis. In vivo exit dosimetry was consistent with this estimate. The keyhole cutout boost was used to deliver 10% of the daily fraction size to correct for this. Due to the high density of the prosthesis, it was clearly visible using on-line portal imaging, allowing interactive positioning of the boost field.

We have been able to carry out in vitro dosimetry experiments to estimate the attenuation of megavoltage photons caused by the shaft of the prosthesis following the generous loan of a non-extendable prosthesis by a patient, which was replaced when he unexpectedly grew. The dose beyond the prosthesis in 'tissue' has been measured using a Roos parallel-plate ion chamber in a water tank. This confirms that both 'Effective Depth' and 'Modified Batho' algorithms give a good approximation to the measured dose using an electron density of 3.4 for titanium alloy.

We believe that this is a valuable technique which improves homogeneity of radiotherapy to a site which includes a metal prosthesis. In typically similar relatively radioresistant tumours a 10% dose attenuation could have a significant impact on local control and hence overall outcome for the patient.

### P20 AUTOCRINE STIMULATION OF EWING'S SARCOMA GROWTH 

J Withey* and SA Burchill, Candlelighter's Children's Cancer Research Laboratory, ICRF Cancer Medicine Research Unit, St. James's University Hospital, Leeds LS9 7TF, UK

Growth factors and their receptors are important in normal cell growth, activating signalling pathways which can stimulate or inhibit cell division, differentiation and migration. Aberrant expression of these proteins can contribute to tumorigenesis by modulating tumour cell attachment, growth and angiogenesis. The aim of this study was to determine whether growth factors and their receptors provide a growth advantage in the neural-crest derived Ewing's Sarcoma (ES).

Six ES cell lines were studied (TC-32, RD-ES, TTC-446, SK-ES-1, A673, SK-N-MC). Growth of four cell lines was examined under normal, reduced and serum-free conditions. A neuroblastoma cell line (SK-N-SH) was used as a control. To determine if cell survival was due to a secreted factor, four ES cell lines were used to condition flasks of media for 1 or 2 days. ES cells were then cultured in this conditioned media and cell number determined at 0, 48 and 72 h. Atlas cDNA arrays (Clontech) were used to look at the range of growth factors and receptors expressed by all 6 ES cell lines to identify potential autocrine and paracrine loops.

All four ES cell lines studied maintained their cell number in serum-free media for up to 72 h; in contrast the neuroblastoma cell line showed a significant decrease in cell number ($p<0.001$). Conditioned media from all 4 ES cell lines increased cell number, indicating that ES cells secrete a factor or factors which have a survival and/or mitogenic effect. Using cDNA arrays we identified 4 growth factors which are abundantly expressed in all 6 cell lines and a further 11 which are expressed in more than half the cell lines. Expression of growth factors and receptors of interest has been confirmed by RT-PCR and/or western blot analysis.

In summary autocrine and/or paracrine loops provide ES cells with a growth/survival advantage. These loops are potential targets for modulating ES behaviour and may provide a new therapeutic strategy for ES.

### P21 EXPRESSION OF TRANSFORMING GROWTH FACTOR RECEPTORS TYPE1 AND TYPE2 IN SUPERFICIAL TRANSITIONAL CELL CARCINOMA OF THE BLADDER

P Maheshkumar[1]*, JE Martin[2], VH Nargund[1] and DM Berney[2], [1]Dept of Urology and [2]Dept of Histopathology and Morbid Anatomy, Barts and The London NHS Trust, London ECIA 7BE, UK

**Objectives** Transforming growth factor-beta (TGFβ) is a potent inhibitor of cellular proliferation in cells of epithelial origin. It acts through two membrane receptors (TGFβRI and TGFβRII). It has been shown in colonic, gastric and head and neck malignancies that there is decreased expression of TGFβRII due to genetic mutations leading to initiation and progression in these tumours. Previous studies in transitional cell carcinoma (TCC) bladder have shown conflicting results on the role of TGFβ receptors in the biology of this disease.

**Materials** Formalin fixed tumour tissue from 49 patients with newly diagnosed superficial TCC bladder (pTa=16 and pT1=33) were analyzed by immunohistochemistry for TGFβRI and TGFβRII following microwave antigen retrieval.

**Results** TGFβRI and TGFβRII were both present in all superficial TCC's irrespective of grade and stage. TGFβRI was strongly expressed in the basal layer and TGFβRII was strongly expressed in both basal and superficial layers, similar to normal urothelium. TGFβRI was moderately expressed in superficial TCC in contrast to TGFβRII, which was strongly expressed as in normal urothelium. Both receptors were also expressed in invasive areas.

**Conclusion** We suggest that loss of TGFβ receptors is infrequent in superficial bladder cancer and plays little part in the oncogenesis of the early stages of TCC bladder.

### P22 INDUCTION OF EXPRESSION OF FGF RECEPTOR 2-IIIB IN OVARIAN CANCER

IA Steele*[1], RJ Edmondson[2], B Bolger[3], J Bulmer[4], HY Leung[1], BR Davies[1], [1]Dept of Surgery. The Medical School, University of Newcastle-upon-Tyne, [2]Northern Gynaecological Oncology Centre, Queen Elizabeth Hospital, Gateshead, [3]Obstetrics and Gynaecology and [4]Pathology, Royal Victoria Infirmary, Newcastle-upon-Tyne, UK

**Introduction** Ovarian cancer is the leading cause of death from gynaecological malignancy in the UK today. Due to its asymptomatic progression, little is known about the early stages of the disease, however, the majority of ovarian carcinomas arise from the ovarian surface epithelium (OSE). The fibroblast growth factors (FGFs) are a family of structurally related polypeptides, implicated in a wide range of solid tumours. They act through a series of high-affinity membrane receptors whose response is mediated by multiple ligands. FGFR2-IIIb is a spliced variant of FGFR2 which binds the ligands FGF-1 and FGF-7 with high affinity. Epithelial ovarian cancers are more highly committed to an epithelial phenotype than the relatively uncommitted OSE from which they arise. For instance induction of E-cadherin and CA125 expression observed in the majority of ovarian cancers. Thus, we hypothesised that expression of the epithelial marker FGFR2-IIIb may accompany metaplasia in the malignant transformation of ovarian surface epithelium.

**Methods and Materials** Primary cultures of human OSE were generated by scraping the surface of ovaries from women undergoing surgery for benign conditions. Total RNA was isolated from cells and samples of epithelial ovarian cancers snap frozen at the time surgery. Expression of FGFR2-IIIb, FGF-1 and FGF-7 mRNA was determined by RT-PCR. Formalin fixed, paraffin-embedded sections of normal and tumour tissue were stained with antisera to FGF-1 and a mouse anti-human FGFR2-IIIb immunoglobulin chimera. The proliferative effects of FGF-1 and FGF-7 on normal (n=3) and cancer cell lines (n=3) was determined using a DNA synthesis assay.

**Results** The expression of FGFR2-IIIb mRNA was undetectable in normal OSE, whilst expression of both FGF-1 and FGF-7 mRNA was positive. Conversely, expression of FGFR2-IIIb mRNA was clearly detectable in 8/16 (50%) of ovarian cancers. FGF-7 mRNA expression was retained in 90% of ovarian cancers, whereas only 8/16 (50%) expressed FGF-1 mRNA. Imunocytochemistry demonstrated a similar pattern of results for expression of the receptor and FGF-1 proteins. Ovarian cancer cell lines expressing the receptor showed a significant increase in cellular proliferation in response to FGF-1 and FGF-7, whereas primary cultures of normal OSE failed to respond to the ligands.

**Discussion** Expression of FGFR2-IIIb was induced in a significant proportion of the epithelial ovarian cancers and ovarian cancer cell lines that express this receptor responded to its ligands, FGF-1 and FGF-7. Signalling through this receptor may be mediated via an autocrine or paracrine mechanism and may represent an important stage in epithelial ovarian cancer development.

### P23 GONADOTROPHINS DIRECTLY STIMULATE THE PROLIFERATION OF HUMAN OVARIAN SURFACE EPITHELIAL CELLS

RJ Edmondson,[1] JM Monaghan[1], BR Davies[2], [1]Northern Gynaecological Oncology Centre, Queen Elizabeth Hospital, Gateshead, Tyne and Wear; [2]Dept of Surgery, University of Newcastle upon Tyne, UK

**Introduction** Over 85% of ovarian cancers arise from the ovarian surface epithelium. Epidemiological evidence suggests that high levels of the gonadotrophins, LH and FSH may be associated with the development of ovarian cancer. Although it has been shown in a mouse model *in vivo* that gonadotrophins can stimulate proliferation of the epithelium(1), little is known about the effects of gonadotrophins on the human ovarian surface epithelium.

**Methods** We have established primary cultures of ovarian surface epithelium, by scaping the surface of the ovaries, from patients undergoing oopherectomy for benign disease(2). Epithelial phenotype was confirmed by staining with a panel of cytokeratin antisera. Total RNA was isolated and the presence or absence of LH and FSH receptor mRNA was determined using RT-PCR. The effects of gonadotrophins on DNA synthesis was determined by $^{3}H$ thymidine incorporation assay.

**Results** We have shown that the mRNA encoding the LH receptor is expressed by 6 of 6 primary cultures. Only 1 of 6 cultures appears to express the FSH receptor. LH causes a modest but significant induction of DNA synthesis in all cultures when given at physiological doses. FSH induces a smaller but significant induction of DNA synthesis in cultures which express the FSH receptor mRNA.

**Conclusions** We have shown that physiological doses of gonadotrophins can cause direct stimulation of human ovarian surface epithelium. These observations lend support, at the cellular level, for the gonadotrophin theory of ovarian carcinogenesis. We are currently studying other physiological effects of gonadotrophins on the OSE, including the rate of apoptosis.

1 Davies B, et al (1999) Administration of gonadotropins stimulates proliferation of normal mouse ovarian surface epithelium. *Gynecol Endocrin* **13:** 74–81
2 Kruk P, Maines-Bandiera S and Auersperg N (1990) A simplified method to culture human ovarian surface epithelium. *Lab invest* **63:** 132–136

### P24 PROFILE OF EGFR EXPRESSION IN HUMAN MALIGNANCIES: EFFECTS OF EXPOSURE TO EGF AND ITS BIOLOGICAL INFLUENCE ON ESTABLISHED HUMAN TUMOUR CELL LINES

Nouri AME, Thompson C, Cannell H, Symes M, Amirghofran Z, Uro/Oncology Research Unit, The Royal London, London E1 1BB, UK

The aim of this study was to compare the profile of EGFr expression in transitional cell carcinoma of the bladder (TCC) and in oral squamous cell carcinoma (OSCC). In addition, to study the influence of EGF stimulation on the expression of major histocompatibility complex (MHC) class I antigens, placental alkaline phosphatase (PLAP) as well as changes in tumour cell sensitivity to cisplatin using immunocytochemical staining, a colorimetric assay and SDS-gel electrophoresis.

The results showed that:

1. Strong EGFr expression in could be seen in 22/88 (27%) cases of TCCs. In oral tumours the values for non-invasive ameloblastoma and invasive OSCC were 4/25 (16%) and 30/41 (73%) respectively.
2. EGFr expression in tumour cell lines paralleled that of tumour biopsies. The number of lines expressing high and low EGFr expression amongst TCCs were 4 and 4 and in OSCCs were 3 and 1 respectively.
3. Exposure of tumour cell lines to EGF led to:
   i. An increase in EGFr expression (stimulatory indices SI, ranged from 1.06 to 2.58) for TCCs but a decrease in the case of OSCCs (SI ranged from 0.01 to 0.85). The corresponding SI values for class I antigens were 0.95 to 1.16 and 0.10 to 0.84.
   ii. A significant reduction in expression of PLAP by OSCC cell lines.
   iii. An increased susceptibility of OSCC cell lines to cisplatin by as much as 14% ($p<0.001$).

These data demonstrated the over expression of EGFr in a significant proportion of TCCs. As for oral tumours it depended on whether they were of an invasive or non-invasive type. In the invasive cases the majority over-expressed EGFr. The exposure of OSCC but not TCC tumour cells to EGF resulted in down regulation of EGFr and class I antigens. The expression of PLAP was also significantly reduced. Exposure of OSCC cell to EGF resulted in their increased susceptibility to cisplatin. The data supports the notion that the mitogenic activation of some tumour cells by EGF resulted in a reduction of their immune visibility, differentiation status and an increase in chemo-sensitivity.

### P25 KINETIC AND *IN VIVO* ANALYSIS OF THE SOLUBLE IGF2 RECEPTOR

J Morrice*[1], G Groeger[1], MH Brown[2], AN Barclay[2], CF Graham[1] & AB Hassan[1], [1]Department of Zoology & [2]Sir William Dunn School of Pathology, University of Oxford, South Parks Road, Oxford OX1 3PS UK

The aim of this study was to engineer IGF2R constructs that would bind IGF-II with high affinity and affect IGF-II driven growth. The insulin-like growth factor 2 receptor (IGF2R) binds and internalises mannosylated proteins and IGF-II. Loss of IGF2R would therefore be expected to increase circulating levels of IGF-II, mannosylated proteins and proteolytic enzymes. Latent TGFβ binds to IGF2R before activation by plasmin hence a decrease in IGF2R would also reduce levels of active TGFβ, a potent growth inhibitor. IGF2R may therefore be a tumour suppressor gene, evidence supporting this includes early onset LOH and IGF2R gene mutation in hepatocellular, breast, ovarian and colorectal cancer.

IGF2R is composed of 15 homologous extracellular domains. Mannosylated proteins bind to domains 3 and 9 and IGF-II binds to domain 11. Domain 13 is thought to enhance IGF-II binding. sIGF2R (domains 1–15) was cloned and its affinity for IGF-II was compared with peptides designed around domain 11 using BIAcore. sIGF2R bound IGF-II with the highest affinity, $9.55 \times 10^{-9}$ M (see table). The lower affinities of domains 10–12, 11–12 and 11 for IGF-II, compared to domains 11–13 and 10–13, supports the observation that domain 13 enhances IGF-II binding. A point mutation (T to C converting $Ile^{1572}$ to Thr) abolished IGF-II binding and was used as a control. Insulin and IGF-I did not bind. β-glucuronidase bound with high affinity to the full length construct but did not bind to construct 10–13. The effect of pH on IGF-II affinity was also analysed: $k_a$ was not significantly affected by pH however $k_d$ was significantly reduced at pH 5.5, the approximate pH of lysosomes. No binding was observed at pH 4.5. The pH within a tumour environment is approximately pH 6.8.

| Domains | $k_a$ (M/s) | $k_d$ (/s) | $K_D$ (M) |
|---|---|---|---|
| 1–15 | $1.18 \times 10^6$ | 0.0113 | $9.55 \times 10^{-9}$ |
| 10–13 | $1.64 \times 10^5$ | $8.08 \times 10^{-3}$ | $4.93 \times 10^{-8}$ |
| 11–13 | $1.0 \times 10^5$ | $4.26 \times 10^{-3}$ | $4.26 \times 10^{-8}$ |
| 11–12 | $6.13 \times 10^4$ | $8.2 \times 10^{-3}$ | $1.34 \times 10^{-7}$ |
| 10–12 | $2.48 \times 10^4$ | $8.6 \times 10^{-3}$ | $3.46 \times 10^{-7}$ |
| 11 | $9.0 \times 10^4$ | 0.0138 | $1.53 \times 10^{-7}$ |

The effect of the IGF2R constructs on IgF2-dependent cell lines (tera-2 and T3/73) is currently being investigated and will be presented.

This work is supported by the CRC Grant 2390/0201.

### P26 THE SOLUBLE IGF2 RECEPTOR REDUCES SMALL INTESTINE ADENOMA NUMBER IN THE *APC$^{MIN/+}$* MOUSE

JA Howell and AB Hassan*, Cell and Development Group, Department of Zoology, University of Oxford, South Parks Road, Oxford OX1 3PS, UK

The *Apc$^{Min/+}$* mouse develops multiple, small intestinal, adenomas that progress to invasive tumours depending on genetic background. Insulin-like growth factor-II (IGF-II) is an embryonic growth factor which is ubiquitously expressed in human cancer and whose gene is imprinted, with expression from the paternal allele. IGF-II appears to be the most abundant mRNA in colorectal cancer, with frequent biallelic expression. Genetic manipulation of IGF-II supply modifies the growth of intestinal adenoma in *Apc$^{Min/+}$* (Hassan and Howell 2000 Cancer Res 60; 1070–76). IGF-II supply can also be altered by the IGF-II/M6P receptor (IGF2R), which normally functions to internalise IGF-II for degradation. There is frequent LOH (6q26–7) and IGF2R gene mutation in hepatocellular, breast, ovarian and colorectal cancer.

In the present study, we investigate whether limiting the supply of IGF-II, by expressing a soluble form of the IGF2R, modifies adenoma growth in the intestine of *Apc$^{Min/+}$* mutant mice. Female *Apc$^{Min/+}$* (isogenic C57BL6J) were mated with male mice over-expressing a soluble IGF2R transgene delivered with a keratin 10 promoter transgene, *K10sIgf2r$^{/+}$* (Zaina et al 1998 Endocrinology, 139: 3886–95, inbred onto 129/JSv). Transgene expression is mainly confined to the colon, uterus, skin, with lower expression in the small intestine. Animals were housed with sex matched littermates and were fed normal chow (3.5% fat). Genotypes were determined by PCR and the small intestine and colon processed as previously described (Hassan and Howell 2000 Cancer Res 60; 1070–76). Combination of *K10sIgf2r$^{/+}$* and *Apc$^{Min/–}$* resulted in a 3 fold reduction in small intestine adenoma number by post-natal day 150, when compared to *Apc$^{Min/+}$* littermate controls (6.35±1.4, n=34, *vs* 18.1±2.6 n=24, respectively, mean±SEM adenoma/mouse, p=0.0001 Mann Whitney). Although transgene expression reduced small intestine surface area (*K10sIgf2r$^{/+}$*, *Apc$^{Min/+}$*, 26.7±0.7 cm$^2$ *vs* *Apc$^{Min/+}$*, 31.8±1.1 cm$^2$, mean±SEM, p=0.0003), it failed to result in equivalent effects on adenoma diameter. We conclude that soluble IGF2R receptor can modify the effects of small intestinal adenoma growth. Expression of the transgene has been previously shown to be greater in the mouse colon, and experiments are now in progress which investigate the effect of soluble IGF2R on colorectal adenoma progression.

Experiments were performed following approval of the Home office and Departmental ethics committee and following the guidelines of the UKCCR. *Supported by Senior Clinical Research Fellowship Grant (2390/0101) of the Cancer Research Campaign.

### P27 THE *IN VITRO* METABOLISM OF ANTAGONIST G, A BROAD SPECTRUM GROWTH FACTOR ANTAGONIST, IN THE LIVER CYTOSOL OF MOUSE, RAT AND MAN

MA Mark*, S Clive, AJ MacLellan, J Cummings, JF Smyth, DI Jodrell, ICRF Medical Oncology Unit, University of Edinburgh, Western General Hospital, Edinburgh EH4 2XU, UK

Antagonist G ($NH_2$-Arg-DTrp-MePhe-DTrp-Leu-Met-$NH_2$), a substance P analogue, is a broad spectrum neuropeptide antagonist being developed clinically as treatment for small cell lung and other cancers. The enzyme involved in the metabolism of Antagonist G is widely distributed, the highest specific activity being detected in cytosolic fractions of the liver (Jones et al, 1995, Biochem. Pharmacol., 50(5), 585). The enzyme is capable of both deamidation (optimal at neutral pH) and carboxypeptidase activity (optimal at acidic pH). The *in vitro* metabolism of Antagonist G proceeds from the c-terminus via deamidation to form Metabolite (Met) I and then removal of methionine to form Met II. In pharmacokinetic (PK) studies of Antagonist G, administered by i.v. infusion, in female Wistar rats the relationship between dose and steady state concentration appears to be non-linear suggesting clearance is saturable and suggesting metabolism is a significant mechanism of drug clearance. Liver cytosol preparations (LC preps) from mice, rats and humans were prepared and incubated with Antagonist G to investigate species differences in metabolism. Incubations were performed at neutral pH to favour deamidation to Met I and to limit the formation of Met II. Experiments were performed in triplicate with rodent LC preps being a mix of at least 2 animals. Mouse livers were obtained from female *nu nu* mice, and rat livers from female Wistar rats. Human liver specimens were obtained fresh during hepatic surgery (n=4). Previous PK studies with Antagonist G utilised electrochemical detection, but we have developed a new reverse phase HPLC method using UV photodiode array detection which allows the simultaneous determination of both Antagonist G and its metabolites (Cummings et al, 1999, J. Chromatog. B, 732 277). When used to analyse LC preps, the mean retention times of Antagonist G and Met I and Met II, were 15.1 mins, 13.4 mins and 12.3 mins respectively. The rate of formation of Met I was equivalent to the disappearance of Antagonist G and conformed to Michaelis Menten kinetics in all 3 species. However, estimates of Vmax (the maximum production rate of metabolism) and Km (the concentration of substrate at which half of the active sites are filled), estimated using GraphPad Prism™ differed between species. Vmax was greater in the human LC preps (2110 ± 497 (mean ± 95% C.I.) µmoles $min^{-1}$ mg $protein^{-1}$) compared to the mouse (849 ± 167) and rat (832 ± 69), the rodent species giving similar estimates. Km also varied between species (rat 19.3 ± 6.2 µM, compared to mouse, 39.6 ± 25.4 and human, 133 ± 80.3). Non-linear PKs have been identified in the phase I clinical trial but a PK model has not been fully defined, perhaps because the plasma concentrations achieved (≤ 46 µM) are less than 50% of the estimated Km for the LC prep.

### P28 RAPID DETECTION OF CIN STATUS IN HUMAN TUMOUR CELL LINES USING A CENTROMERIC FISH 3 PROBE SYSTEM

Jaffrey RG*, Marsh S, Cassidy J, McLeod HL, Oncology Group Dept of Medicine and Therapeutics, University of Aberdeen AB25 2ZD, UK

Chromosome instability (CIN) is a characteristic that is seen, as aneuploidy, in a majority of tumour types. The role of CIN is poorly understood in the progression to malignancy and in chemotherapy resistance. The loss or gain of chromosomes (aneuploidy) is generally viewed in the early stages of tumour evolution, accumulating as the tumour becomes increasingly advanced. Several recent studies have focused their attention to the possible mechanisms of action of this CIN phenotype. We have proposed a model for the rapid detection of the CIN phenotype in human cancer. Analysis of centromeric probes by FISH was performed in 11 human cancer cell lines with defined CIN status: 7 CIN+ (BE, CACO2, H630-R10, H630, HT29, L-DAN and MCF-7), and 4 CIN- (RKO, DLD-1, LOVO and WIL-2) and a normal leukocyte control. Primary analysis of these cell lines used 11 centromeric probes and clearly separated the two subgroups based on mean centromeric signal per cell (p<0.001). All CIN+ cell lines had a mean centromere number >2.8 and all CIN-cells were <2.1. Analysis of predictive performance identified several probes with a high ability to predict CIN status (κ=1). Use of data from probes for centromeres 11, 17 and 18 gave 100% ability to rapidly determine CIN phenotype in human cancer cell lines. This centromeric CIN model will help researchers to accelerate the pursuit of the complex and subtle mechanisms involved in chromosome catastrophe.

### P29 DETECTION OF AMPLIFIED AND TRANSCRIBED SEQUENCES AT THE CHROMOSOME 12Q13–15 LOCUS IN NGP NEUROBLASTOMA CELLS BY ALU-PCR

LIB Pinto[1], ADJ Pearson[2], R Perry[3], J Lunec[1], [1]Cancer Research Unit, [2]Dept Child Health, [3]Dept Pathology, University of Newcastle NE2 4HH, UK

Alu-PCR fingerprinting has been used to disclose genetic abnormalities in cancer that may be associated to activation of oncogenes or inactivation of tumour suppressor genes. This technique is based on polymerase chain reaction (PCR) amplification of genomic DNA with primers specific for the Alu repeat sequences, which are distributed ubiquitously throughout the genome. Several types of primers based on the consensus Alu sequence have been designed. This primers can be used alone or in combination with other Alu primers or primers to other interspersed repeat elements to amplify inter-Alu sequences from human DNA. We have used this strategy to study 7 neuroblastoma cell lines compared to 8 normal samples (4 blood samples and 4 cerebellum samples). Amplification of 2 DNA fragments was detected in the NGP cell line. The fragments have been cloned into the p-GEM-T-easy vector (Promega) and automated sequenced. The sequences were flanked by Alu repeat elements and the inter-Alu sequences were searched against the GENEBANK database. A novel 623 bp sequence was disclosed and the other sequence was revealed to be part of the advillin gene. The 623 bp fragment and a 808 bp PCR product from the advillin gene were used as probes for Southern-blot analysis of genomic DNA of the NGP cell line and confirmed the amplification observed by Alu-PCR fingerprinting. Both fragments were also used as probes for Northernblot and increased expression on filters carrying total RNA from the NGP cell line was disclosed when compared with total RNA from the cell lines SHEP and SKNSH. Radiation hybrid mapping revealed that the 623 bp fragment was amplified from a region of chromosome 12q13–15, where the MDM2 and the advillin loci have been previously mapped. The results indicate that Alu-PCR fingerprinting is useful for the detection and cloning of amplified genomic regions and the subsequent identification of amplified genes in tumour cells.

### P30 EXPRESSION OF GENES PAX-3 AND PAX-7 IN NEURAL CREST LINEAGE TUMOURS

C Parker[1],* W Wang[2], I Hart[3], K Sisley[4], S Kumar[5] and P Kumar[1], [1]Manchester Metropolitan University, Manchester M13 9PT, [2]Aberdeen University, Aberdeen, [3]St. Thomas' Hospital, London SE1 7EH, [4]Royal Hallamshire Hospital, Sheffield, [5]Manchester University, Manchester M13 9PT, UK

The developmental genes Pax-3 and Pax-7 have been shown to be expressed in the early embryo in developing muscle and elements of the brain and nervous system. Recent studies have shown expression of these genes in both alveolar and embryonal forms (RMS-A and RMS-E) of Rhabdomyosarcoma. In the study here RT-PCR was used to screen melanoma cell lines and primary malignant ocular melanoma tissue along with small cell lung cancer (SCLC) cell lines, all of which are neural crest lineage related. Eleven out of thirteen melanoma cell lines were found to express Pax-3 but not Pax-7. Two cell lines from ocular melanomas, did not express Pax-3 although one of the cell line did express Pax-7. All 8 occular melanoma tissue samples screened also expressed Pax-3 along with 2/3 of the SCLC cell lines. Interestingly a non tumorigenic cell line melan A, did not show Pax-3 expression but did express Pax-3 after transfection with ras.

These results indicate that Pax-3 and Pax-7 may have important role in the development and progression of neural lineage tumours and may serve as prognostic markers for these tumour types. Furthermore the results show that Pax-3 may be involved in the ras transformation of neural lineage tumours.

### P31 PREVALENCE OF *BRCA1* AND *BRCA2* MUTATIONS IN A LARGE POPULATION BASED SERIES OF BREAST CANCER CASES

Bettina Kuschel*, For the The Anglian Breast Cancer Study Group, University of Cambridge Dept of Oncology, Strangeways Research Laboratories, Worts Causeway, Cambridge CB1 8RN, UK

**Background** Estimates of the contribution of *BRCA1* and *BRCA2* to breast cancer incidence in outbred populations have been based on studies that are either small or have selected for cases diagnosed at an early age. Only one of these has reported an estimate of the breast cancer risk associated with a mutation in these genes, and there is no published ovarian cancer risk estimate derived from a population based case series.

**Method** A population-based series of breast cancer cases Anglian Cancer Registry comprising a prevalent series of cases diagnosed under age 55 was screened for mutations in *BRCA1* and *BRCA2* by multiplex heteroduplex analysis. In order to assess the importance of survival bias, we also screened the subgroup of an incident population based case series diagnosed under age 35 together with the incident cases with the strongest family histories. Pedigree information from mutation carriers was used to estimate penetrance and the proportion of familial risk of breast cancer due to *BRCA1* and *BRCA2*.

**Results** We identified 8 (0.7%) *BRCA1* and 15 (1.2%) *BRCA2* mutation carriers in 1220 prevalent breast cancer cases. Mutation prevalence was substantially higher in cases diagnosed before 35 years of age and with increasing number of relatives affected with breast or ovarian cancer. However, most mutation carriers were diagnosed in the older age groups, and a minority reported a first degree relative with breast cancer. Breast cancer penetrance by age 80 was estimated to be 48% (95% CI 7–82%) for *BRCA1* mutation carriers and 74% (7–94%) for *BRCA2* mutation carriers. Ovarian cancer penetrance for *BRCA1* and *BRCA2* combined was 22% (6–65%) by age 80. Seventeen per cent of the familial risk of breast cancer was attributable to *BRCA1* and *BRCA2*. At birth, the estimated prevalence of *BRCA1* mutation carriers was 0.07%–0.09% and 0.14%–0.22% for *BRCA2*. Updated results, with data from the mutation analysis of 288 incident cases will also be presented.

**Conclusions** Mutations in the breast cancer susceptibility genes *BRCA1* and *BRCA2* are rare in the population and account for a small fraction of all breast cancer in Britain, even in a population based series of young diseased patients without any survival bias.

### P32 ESTROGEN RECEPTOR GENE POLYMORPHISMS AND MALE BREAST CANCER

IE Young*[1], KM Kurian[1], MAF MacKenzie[1], IH Kunkler[2], BB Cohen[3], CM Steel[3], 1/C.R.C. Laboratories, Molecular Medicine Centre & 2/Dept. of Clinical Oncology, Western General Hospital, Crewe Road, Edinburgh EH4 2XU, 3/School of Biomedical Sciences, University of St. Andrews, St. Andrews, Fife KY16 9TS, UK

In a previous study we have shown an association between a polymorphism in the CYP17 gene and male breast cancer[1]. Other studies, looking at polymorphisms in the estrogen receptor gene, have not shown a consistent association with female breast cancer[2]. The aims of this study were to investigate the frequencies of two estrogen receptor gene polymorphisms in a group of male breast cancer patients from South East Scotland.

Cases were a consecutive series of 76 male breast cancer patients treated in S.E. Scotland between 1974 and 1998. 64 DNA samples were available (from blood samples in 25 cases, archival tissue in 39). Control DNA samples were obtained from 77 healthy males representative of the S.E. Scotland population. The relevant fragments of intron 1 and exon 2 containing the polymorphic sites were amplified by polymerase chain reaction (PCR). The intron 1 products were digested overnight by Pvull at 37°C and the exon 2 products by BstUI at 60°C. The products were then separated on 4% agarose gels, stained with ethidium bromide and visualised under ultraviolet light.

The distribution of alleles of exon 2 of the estrogen receptor gene is shown in the table:

| Allel | Cases (n=63*) | Controls (n=77) |
|---|---|---|
| W:W | 46 | 67 |
| W:P | 16 | 10 |
| P:P | 1 | 0 |

(W=Wild type, P=Polymorphic i.e. cut by BstUI)
*PCR unsuccessful with one DNA sample

27% of males with breast cancer carry a polymorphic allele in exon 2 compared to 13% of controls. This difference is statistically significant ($\chi^2$=4.361, 1 df, p=0.0368). The odds ratio for the risk of developing breast cancer in a male with a polymorphic allele in exon 2 is 2.476 (95% CI 1.041 to 5.890). There were no significant differences between cases and controls in the distribution of intron 1 alleles (data not shown).

We have demonstrated an association between a polymorphism in exon 2 of the estrogen receptor gene and male breast cancer. This is now the second modifying polymorphism that we have associated with an increased risk of male breast cancer. We believe that this further illustrates the potential importance of these low-penetrance genes in the development of breast cancer.

1 Young et al (1999) *Br J Cancer* **81:**(1) 141
2 Andersen et al (1994) *Hum Genet* **94:** 665

### P33 IMMUNOHISTOCHEMICAL DETECTION OF ESTROGEN RECEPTOR β IN PARAFFIN-EMBEDDED HUMAN BREAST TISSUES

GP Skliris*[1], PJ Carder[2], J Askaa[3], AF Markham[1] and V Speirs[1], [1]Molecular Medicine Unit, [2]Pathology, St. James's University Hospital, Leeds LS9 7TF, [3]DAKO A/S Denmark

Estrogen receptor (ER) is a prognostic marker for predicting response to endocrine therapy in breast cancer. The cloning of a novel ER, ERβ, has been reported and has been implicated in breast cancer. The presence of ERβ has been provided from *in situ* hybridisation studies and RT-PCR, but little is known concerning the protein. For ERβ to have potential as a diagnostic tool, optimisation of immunostaining for use on paraffin sections is required as most clinical material is processed in this way. The aim of this study was to investigate the expression and distribution of ERβ protein in breast tumours. Twenty tumour breast biopsies, mostly invasive ductal carcinomas, were randomly selected. Five µm sections were cut, mounted on silanised slides and dried overnight, before being microwaved in the presence of citrate buffer pH 6.0. ERβ staining was performed using two different antibodies, of monoclonal and polyclonal origin, while staining for ERα, which acted as a reference point was conducted using the monoclonal antibody ER1D5. Specific strong staining was mainly exhibited in nuclei of epithelial cells using both ERβ antibodies, while occasional cytoplasmic staining was sometimes detected in stromal cells. When the monoclonal ERβ antibody was applied, stronger staining intensity was observed in the nuclei, accompanied with less background. Additionally, ERβ was co-expressed with ERα in most tumours, confirming previous mRNA studies. Specificity of ERβ staining was confirmed in sections where primary antibodies were omitted, substituted with PBS, or pre-absorbed with 5 times excess of blocking peptide. Our results indicate that ERβ protein is detectable in human breast tumours using immunohistochemical techniques. Routine measurement of ER subtypes in these tumours is likely to enhance the ability to individualise therapy and offer a more selective approach in defining the most appropriate initial hormonal intervention. *Supported by Yorkshire Cancer Research*

### P34 BCL-2, ANGIOGENESIS, NECROSIS AND MACROPHAGES IN EARLY BREAST CANCER

KJ O'Byrne*[1], RD Leek[3], RA Walker[2] and AL Harris[3], University Depts of [1]Oncology and [2]Pathology, Leicester Royal Infirmary, LE1 5WW; [3]Imperial Cancer Research Fund Medical Oncology Unit, Churchill Hospital, Oxford OX3 9DS, UK

Bcl-2 is an anti-apoptotic factor the expression of which is paradoxically associated with a favourable outcome in solid tumours including breast cancer. A number of studies have shown a consistent inverse relationship between bcl-2 expression and apoptosis and tumour necrosis (TN). Angiogenesis, the formation of new blood vessels from an existing vasculature, is necessary for a tumour to grow beyond 1–2 mm in diameter and is associated with both macrophage infiltration (MphiI) and TN in breast cancer. An inverse correlation between bcl-2 expression and angiogenesis, assessed by the Chalkley microvessel counting method (MVC), has been found in non-small cell lung cancer (NSCLC). We evaluated the inter-relationships between bcl-2 expression and MVC, p53, estrogen receptor (ER), epidermal growth factor receptor (EGFR), vascular endothelial growth factor (VEGF), thymidine phosphorylase (TP), and tumour necrosis factor(TNF)-α expression, TN, MphiI and clinicopathological features in a retrospective study of 82 patients with operable breast cancer. An inverse relationship was found between Bcl-2 and MVC (p=0.02), tumour grade (p=0.01), p53 (p=0.005), EGFR expression (p=0.048), TN (p=0.002) and MphiI (p=0.02). Bcl-2 was positively correlated with post-menopausal status (p=0.04), increasing patient age (p=0.03) and ER immunopositivity (p<0.0001). Bcl-2 was not associated with tumour size, nodal status or the expression of VEGF, TP and TNF-α. In conclusion, although not of prognostic significance, bcl-2 immunopositivity was associated with favourable prognostic indices in operable breast cancer. In particular bcl-2 expression may result in the inhibition of apoptosis and necrosis induced by tumour hypoxia with a consequent reduction in macrophage infiltration and subsequent angiogenesis.

### P35 LOCALLY ADVANCED BREAST CANCER IS ASSOCIATED WITH A HIGH RATE OF HER2 OVEREXPRESSION

*Bartlett J, Anderson A, *Watters A, Leonard R and Cameron D, Medical Oncology Unit, Western General Hospital, Edinburgh and *Department of Surgery, Glasgow Royal Infirmary, UK

In different series of breast cancer the overexpression of HER2 protein varies from around 9% to 39%, depending on the antibody selected and the patient group. Overexpression is associated with more aggressive behaviour, is inversely correlated with ER expression and presents a target for antibody therapy with the humanised mouse monoclonal antibody, trastuzumab. In many studies there is a close correlation between protein overexpression and gene amplification (Robertson et al, 1996). There is also controversial data that suggests that overexpressing tumours show a dose response effect when exposed to anthracycline chemotherapy in the clinic [Muss et al 1996; Thor et al 1999; Cameron et al 1999]. A series of Edinburgh patients presenting with inoperable breast cancer was treated with infusional 5 fluorouracil and anthracyclines or cyclophosphamide/methotrexate with conventionally adequate outcomes [Davidson et al 1999].

A random selection of 32 patients with T4 disease has been studied for HER2 gene amplification using the Pathyvision FISH diagnostic technique which is reproducible and quantifiable and is superior to IHC which is semiquantitative and may produces either false positives or false negatives [Allred AJCP 2000].

**Results**

| | Inflammatory | ER+VE | Grade 3 | Size>5 cm | 3 yr survival |
|---|---|---|---|---|---|
| FISH+ve(14) | 17% | 49% | 66% | 67% | 57% |
| FISH–ve(18) | 44% | 79% | 59% | 75% | 89% |

Grade and size are not different between the groups but there is an inverse relationship between ER and HER2 +ve patients as expected. Neither disease free nor overall survival is significantly different but there is a trend (p=0.06) to improved survival in the HER2 –ve group. The rate of gene amplification in this series of T4 tumours is 44% which is markedly higher than published rates for gene amplification in breast cancer (Salmon et al, 1987, Mitchell & Press, 1999). It appears that in addition to the high stage of disease in this patient group the rate of amplification is also a factor in the poor outcome for patients. These data have important implications for the prognosis and management of patients with locally advanced breast cancer.

### P36 PERSISTENCE OF C-ERBB-2 OVEREXPRESSION AFTER NEOADJUVANT CHEMOTHERAPY FOR PRIMARY BREAST CANCER

RJ Burcombe*[1], A Makris[1], PI Richman[2], F Daley[2], S Noble[2], GD Wilson[2], [1]Marie Curie Research Wing & [2]The Gray Laboratory, Mount Vernon Hospital, Northwood HA6 2RN, UK

Overexpression of the c-erbB-2 (HER-2) proto-oncogene in breast cancer patients is associated with worse prognosis and has been implicated in resistance to chemotherapeutic agents, including anthracyclines. The use of neoadjuvant chemotherapy provides an *in vivo* model to study the effects of treatment on c-erbB-2 expression.

48 women with primary breast cancer (mean age 57 years, range 27–78) were studied. All patients underwent diagnostic core biopsy prior to 6 cycles of neoadjuvant FEC chemotherapy (5-Fluouracil 600 mg/m$^2$, Epirubicin 60 mg/m$^2$, Cyclophosphamide 600 mg/m$^2$). Surgery was performed after clinical and radiological assessment of response. Tamoxifen was commenced post-operatively in patients with ER positive tumours.

Immunohistochemical analysis for c-erbB-2 was performed on core biopsies and subsequent surgical specimens using a monoclonal mouse anti-c-erbB-2 antibody (Zymed laboratories). Two independent observers assessed the specimens. Membrane staining in > 10% of tumour cells was scored positive.

Tumours from eight patients (17%) were c-erbB-2 positive on both the core biopsy and surgical specimen. All other tumours stained negative for c-erbB-2 before and after treatment. Clinical response to treatment (CR and PR) was observed in 6/8 (75%) and 28/40 (70%) of c-erbB-2 positive and negative tumours respectively.

**Conclusion** Overexpression of c-erbB-2 was not affected by neoadjuvant anthracycline-based chemotherapy and in this cohort of patients did not predict for resistance to treatment.

### P37

WITHDRAWN

### P38 THE P53 STATUS OF SPORADIC BREAST TUMOURS ASSESSED BY DIFFERENT METHODS

PM Duddy*[1], AM Hanby[2], DM Barnes[2] and RS Camplejohn[1], [1]Richard Dimbleby Dept. of Cancer Res., St. Thomas' Hospital, London SE1 7EH, [2]ICRF Clinical Oncology Unit, Guy's Hospital, London SE1 9RT, UK

Indirect methods of assessing p53 do not give an accurate assessment of the mutation status of tumours. In sporadic tumours, *p53* mutations commonly are found in hotspot regions of the gene, such as the core DNA-binding domain. As a result, direct sequencing of the *p53* gene has concentrated on exons 5–8, although a significant minority of mutations have been found in other parts of the gene such as the oligomerisation domain (Hainaut *et al. Nucleic Acids Res. 1998*; **26**: 205–213).

The aim of this investigation was to examine the ability of the yeast-based functional assay, the FASAY, to detect *p53* mutations in breast cancers when compared with immunohistochemistry and automated sequencing of the whole *p53* gene (exons 1–11). To achieve this, all 3 methods were carried out on a cohort of aggressive breast tumours. In those tumours, in which the FASAY analysis indicated the presence of a mutation, cDNA was extracted from red yeast colonies and was sequenced in order to identify the base change in the *p53* gene. The FASAY detected all 24 mutations found in the series of 48 tumours, whilst initial automated sequencing of genomic DNA detected 18/24 mutations. A second round of automated sequencing, carried out using an independent source of genomic DNA, detected mutations in 3 of the 6 tumours which originally appeared to lack a mutation in genomic DNA. All but 1 of the mutations originally missed by sequencing of genomic DNA were point mutations. Five mutations in this series (21%) were outside the commonly investigated exons 5–8, reinforcing the need to extend sequencing beyond this region. All 14 tumours with strong immunohistochemical staining had *p53* mutations; the majority of mutations missed by immunohistochemistry produced a truncated protein. Strong staining was never seen in tumours lacking a *p53* mutation. The FASAY proved to be a rapid, reliable and effective method for identifying those breast tumours harbouring *p53* mutations and could well provide the 'gold-standard' for detection of these genetic defects.

### P39 EXPRESSION OF CYCLOOXYGENASE 2 (COX-2) IN HUMAN BREAST CARCINOMAS

CJ Witton*, LF Clark, TG Cooke and JMS Bartlett, Dept of Surgery, Queen Elizabeth Building, Glasgow Royal Infirmary, Alexander Parade, Glasgow G31 2ER, UK

Nonsteroidal anti-inflammatory drugs (NSAIDs) have been found to be protective against colorectal cancer. These compounds inhibit the catalytic activity of cyclooxygenase (COX) which is involved in the regulation of prostaglandin synthesis(1). Prostaglandins in turn control the production of vascular endothelial growth factor (VEGF) and this growth factor promotes angiogenesis. This process is important for tumour growth and metastasis. Aberrant or increased expression of COX-2 has been reported in colorectal, lung, oesophageal and breast cancers. COX-2 has previously been found to be localised to tumour cells in breast (2). In colorectal cancer it has been suggested that COX-2 expression is regulated by HER2 (3).

We have examined COX-2 in formalin fixed tissue from a randomly selected cohort of patients who were treated in Glasgow for breast cancer between 1984 and 1997. Follow up data was available for all these patients. COX-2 expression was analysed with immunohistochemistry (IHC) using a monoclonal antibody to COX-2. COX-2 staining was carried out using the NexES II IHC Staining System. This is a fully automated, temperature controlled staining system which eliminates variations in results due to temperature variations in the laboratory. Epitope retrieval was performed within a microwave pressure cooker in Tris-EDTA (TE) buffer. COX-2 staining was assessed independently by two investigators using a histoscore based on percentage and intensity of staining with 300 representing a very high intensity stain in all tumour cells and 0 representing no tumour cells stained.

COX-2 staining was localised to the cytoplasm of tumour cells with low to medium intensity (histoscores 120–220). The mean COX-2 histoscore for tumours scored as positive for HER2 by Fluorescent in situ hybridisation (FISH) (n=22, mean=153, sd=20.08) was higher those scored negative (n=55, mean=163, sd=23.30) but this was not significant. The mean histoscores of those patients who had died from their disease (n=42, mean=161, sd=28.71) and those who hadn't (n=58, mean=160, sd=21.25) were also not significantly different.

COX-2 staining therefore did not correlate with HER2 status, in contrast to evidence in colorectal cancer (3), or survival. However since this enzyme has been found in all tumours, treatment with NSAIDs or specific COX-2 inhibitors to decrease COX-2 may have potential in the treatment of breast cancer. Future work will examine expression of VEGF in breast cancer to relate it to this COX-2 data.

1 Shiff SJ, Rigas B (1999) *J Exp Med* **190:** 445–450
2 Hwang D, Scollard D, Byrne J, Levine E (1998) *J Natl Cancer Inst* **90:** 455–460
3 Vadlamudi R, Mandal M, Adam L, Steinbach G, Mendelsohn J, Kumar R (1999) *Oncogene* **18:** 305–314

### P40 LOSS OF EXPRESSION OF THE HIC-1 GENE (HYPERMETHYLATED IN CANCER) FROM 17P13.3 IN BREAST CANCER

G Nicoll, DN Crichton, AM Thompson*, Department of Surgery & Molecular Oncology, University of Dundee DD1 9SY, UK

In addition to the p53 gene, a new cancer gene, HIC-1 (Hypermethylated In Cancer) on chromosome 17p may be of clinical importance in sporadic breast cancer. HIC-1 lies in band 17p13.3 telomeric to p53 at 17p13.1, where loss of heterozygosity studies have suggested clinically significant involvement of a gene associated with poor prognosis in breast cancer. Regional DNA hypermethylation (resulting in suppression of gene expression) of 17p13.3 has been shown to precede 17p structural changes in human carcinogenesis. Thus, loss of HIC-1 expression and p53 malfunction may be common synergistic events in sporadic breast cancer.

Using RT-PCR, HIC-1 transcripts were identified in MCF7 (wild type p53) but not in MDAMB231 (mutant p53), which were used as positive and negative controls respectively. Mdm2 and actin were used as internal controls for the integrity of the RT-PCR process.

HIC-1 expression was sought in the RNA extracted from 31 primary, untreated human breast cancers and was detected in only 6/31 (19%) cancers despite positive internal controls for mdm2/actin and repeat testing of samples.

These data suggest that loss of function of the HIC-1 gene on 17p13.3, a chromosomal region associated with poor prognosis, occurs in a substantial majority (81%) of breast cancers. This presents the opportunity for therapeutic manipulation of the methylation status of genes such as HIC-1 as a novel approach to breast cancer treatment.

### P41 CHROMOSOMAL RADIOSENSITIVITY IN G2-PHASE LYMPHOCYTES IS ASSOCIATED WITH GOOD PROGNOSIS IN BREAST CANCER

A Riches[1], P Bryant[1], AM Thompson[2]*, PE Preece[2], CM Steel[1], [1]School of Biology, University of St Andrews, [2]Department of Surgery & Molecular Oncology, University of Dundee DD1 9SY, UK

Patients who are cancer-prone as a result of certain genetic disorders exhibit a high G2 chromosomal radiosensitivity. It has been demonstrated that peripheral blood lymphocytes from some breast cancer patients also have an increased sensitivity to radiation damage in G2 compared with age matched controls.

The aims of this study were to evaluate the frequency of lymphocyte G2 radiation sensitivity in breast cancer patients in our region and to compare G2 sensitivity to clinical and pathological parameters.

Pretreatment blood samples were collected from 65 women with breast cancer and lymphocytes set up in culture for analysis by the G2 assay. Chromatid aberrations per 100 metaphase spreads (with the unirradiated background subtracted) were determined.

Thirty patients (46%) exhibited an increased radiation sensitivity of peripheral blood lymphocytes compared with 14% (9/66) of control samples (patients undergoing minor surgery with no history of malignancy). In the high G2 group, 81% of cancers were oestrogen receptor positive, while only 45% were oestrogen receptor positive in the low G2 group. Patients with a high G2 score were more likely to be axillary node negative, had smaller cancers and a better prognosis (based on the Nottingham Prognostic Index) than breast cancer patients with a low G2 score.

These data suggest that a high G2 assay score (increased radiation sensitivity) distinguishes a group of women with breast cancer who have a good prognosis. The gene or genes underlying this increased sensitivity to radiation may play an important role in the pathogenesis of breast cancer.

### P42 DOXORUBICIN-CMF CHEMOTHERAPY FOR MULTIPLE NODE-POSITIVE BREAST CANCER – UK EXPERIENCE

*A Anderson, *DA Cameron, *RCF Leonard, Department of Clinical Oncology, Western General Hospital, Edinburgh, Scotland on behalf of the UK Breast Cancer Collaborative Group

Sequential doxorubicin and CMF chemotherapy, as designed by Bonadonna et al [JAMA 1995; 273: 542–547], has the best non-myeloablative medium and long term results in the treatment of multiple node-positive breast cancer. Between 1994 and 1998, 75 patients (pts) [median age 48, range 26–62] with operable multiple node positive breast cancer [median 10 nodes; 3 nodes, 2 pts; 4–9 nodes, 35 pts; and 10+ nodes, 38 pts,] were treated with doxorubicin 75 $mg/m^2 \times 4$ followed by intravenous CMF [cyclophosphamide 600 $mg/m^2$; 5-fluorouracil 600 $mg/m^2$ and methotrexate 40 $mg/m^2$], $\times$ 8 cycles; both q 21 days. Adjuvant locoregional radiotherapy was given to 61 pts using standardised fractionation at 4500–6000 cGy between cycles 2 and 3 of the CMF regimen. Tamoxifen 20 mg/day for 5 years was given to 57 ER-positive pts on completion of chemotherapy.

The projected overall survival is 95% at 3 years for the 4–9 node group and 70% for the 10+ node group [difference between groups; Kaplan Meier statistic p=0.027], similar to that reported by Bonadonna. Tumour grade [15 grade 1 or 2; 60 grade 3], estrogen receptor status and use of tamoxifen are not significant factors in survival analysis. Disease-free survival for the same groups and follow-up periods are 62% and 60%. Site of first relapse was locoregional in 8, distant (with or without local) 18. Local control at 3 years is 80%. Toxicity was generally acceptable in all age-groups. 60 had treatment delays 1–6 weeks, 11 had dose reductions and 9% pts were admitted for neutropenic sepsis. There were no toxic deaths and no clinical cardiac toxicity has been observed.

The regimen has been tested more widely in the UK with 6 centres providing a total of 280 patients. This is the largest series of patients treated with the regimen and the outcomes will be presented.

### P43 ANASTROZOLE (ARIMIDEX™) VERSUS TAMOXIFEN AS 1ST-LINE THERAPY FOR ADVANCED BREAST CANCER (ABC) IN POST-MENOPAUSAL (PM) WOMEN – COMBINED ANALYSIS FROM TWO IDENTICALLY DESIGNED MULTICENTER TRIALS

JFR Robertson, On behalf of the Arimidex 1st-Line study group, Dept of Surgery, Nottingham City Hospital, UK

Anastrozole (‘Arimidex’) (AN) is a non-steroidal aromatase inhibitor used to treat ABC in PM women whose tumors recur/progress on tamoxifen (TAM). We report on two randomized, double-blind trials (performed in the US and Canada [T30] and Europe/rest of the world [T27]), which compared AN 1 mg od and TAM 20 mg od as first-line therapies for ABC in PM women with ER+ve and/or PR+ve or unknown tumor receptor status. The design allowed for combined data analysis. Primary endpoints were time to progression (TTP), objective response (OR), and tolerability.

1021 patients (n=353 [T30]; n=668 [T27]) were included in the combined analysis. Median duration of follow-up was 18.2 months. Median TTP was 8.5 months for AN and 7.0 months for TAM with an estimated hazard ratio relative to the control arm, TAM, of 1.12 (lower 95% CL, 1.00) showing AN to be at least equivalent to TAM. OR (defined as complete or partial response [CR+PR]) was 29% for AN and 27% for TAM. Efficacy of AN relative to TAM was explored within subgroups defined retrospectively by receptor status, prior hormonal treatment, site of disease and age. These analyses showed a significant prolongation of TTP in the AN arm (median values of 10.7 and 6.4 months for AN and TAM, respectively, 2-sided p=0.022) in patients confirmed as ER+ve and/or PR+ve (57.7% of combined trial population). In patients with visceral lesions, AN was equivalent to TAM in terms of TTP. Both AN and TAM were well tolerated by most patients. However, both thromboembolic events and vaginal bleeding were reported in half as many patients treated with AN as those treated with TAM [4.5 versus 7.6% (thromboembolic) and 1.2 versus 2.9% (vaginal bleeding), respectively].

In conclusion, in PM women with hormonally-sensitive ABC, these data suggest that AN should be considered as an alternative first-line treatment to TAM.

### P44 ADJUVANT FEC AND CMF CHEMOTHERAPY DOSE INTENSITY IN THE LEEDS BREAST UNIT

RA Popescu*[1], A Henry[2], DJ Dodwell[2] and TJ Perren[1], [1]ICRF Cancer Medicine Research Unit, St. James's Hospital Leeds LS9 7TF, [2]Radiotherapy Dept, Cookridge Hospital LS16 6QV, UK

A clear relationship exists between dose intensity of a variety of adjuvant chemotherapy regimens and outcome in patients with early breast cancer. However, the number of clinical trials that report the achieved dose intensity in this population is small, and data is scarce on dose intensity delivered in routine clinical practice.

Between 1 March 1998 and 30 September 1999, 160 patients received adjuvant cytotoxic treatment in the Leeds Breast Unit. Using the computerised chemotherapy prescription records, we identified all women scheduled to receive 6 courses of adjuvant Bonadonna CMF or FEC chemotherapy, and assessed the number of chemotherapy cycles administered, treatment delays, toxicity and dose intensity achieved.

*Adjuvant FEC chemotherapy* (Fluouracil 600 $mg/m^2$ iv d1, Epirubicin 60 $mg/m^2$ iv d1 and Cyclophosphamide 600 $mg/m^2$ iv d1 every 3 weeks) was administered to 86 women (mean age 46, range 31–63). 84 patients were given 6 cycles, one patient 5 and another 4 cycles of treatment (mean 5.97 cycles). 49 patients (57%) completed their treatment without any delays, 17 were delayed for one week and 13 for two weeks; 2 patients were delayed for 4 or more weeks. Of all cycles, 11% were delayed, of which 4/5th due to neutropenia or sepsis. Likelihood of dose reductions increased as chemotherapy courses were accumulating, but 82% of patients receiving a sixth course had 95% or more of the planned dose given. Relative dose intensity (DI) administered was on average 95% of planned DI, the median relative DI was 100%. Of all 86 patients, 12 (14%) achieved less than 90% of planned DI, and 7 (8%) were given less than 80% of planned DI.

*Bonadonna CMF* was given to 33 patients (mean age 55, range 32–75). Twenty-four patients completed the planned 6 cycles, but 7 patients (21%) received 5 cycles and 2 (6%) 4 cycles (mean 5.64 cycles). Of all chemotherapy cycles administered, 26% were delayed, 19% due to neutropenia or sepsis and 6% due to radiotherapy scheduled after the 3rd course. Dose reductions were necessary in 25 patients (76%). The average relative dose intensity achieved was 82% (median 83%, minimum 50%). Two thirds of patients (22) had less than 90% of planned DI, one third less than 80% and 4 patients (12%) less than 65%. In 11 patients, planned radiotherapy given after the 3rd chemotherapy cycle significantly prolonged total treatment duration, thus contributing to reducing achieved DI. For the remaining 22 patients, relative achieved DI was WW%.

There was no correlation between age of patients or nadir blood counts after the first cycle of FEC (where available) and the achieved DI. Patients with early occurrence of delays or dose reductions were most likely to attain a lower DI. In conclusion, a high relative dose intensity is achievable in the majority of patients treated with FEC chemotherapy in routine clinical practice. In our small series, CMF was more toxic and patients achieved lower relative DI.

### P45 THE ROLE OF ZOLADEX IN THE PREVENTION OF OVARIAN DAMAGE DURING CHEMOTHERAPY FOR PRIMARY BREAST CANCER

EF Shah*[1], J Woodside[1], R Chatterjee[2], R Stein[3], C Saunders[1], [1]Dept of surgery, [2]Dept of Reproductive Medicine, [3]Dept of Oncology, University College London, 67–73 Riding House St, London W1P 7LD, UK

**Aims** The aim of this study was to determine whether temporary interruption of the hypothalamic-pituitary axis by administration of a luteinising hormone releasing hormone agonist, goserelin, during cytotoxic chemotherapy, would diminish the incidence of chemotherapy-induced premature ovarian failure in premenopausal women with breast cancer.

**Methods** A retrospective analysis was carried out on women selected from the Cancer Research Campaign Adjuvant Breast Trial for women under fifty years old. Twelve patients, aged 33–48 years at diagnosis, were evaluated at a mean interval of 2.4 years after completion of treatment. Six had been treated with goserelin during their course of adjuvant chemotherapy and the other six, who served as controls, received only the chemotherapy. Chemotherapy consisted of cyclophosphamide, methotrexate and 5-fluorouracil in both groups. At interview, patients completed questionnaires on their menstrual and reproductive history. Serum oestradiol, follicle-stimulating hormone (FSH) and luteinising hormone levels were measured in all patients. Ovulation was assessed by mid-luteal serum progesterone peak levels.

**Results** Eight patients, three in the control and five in the study group, developed biochemical evidence of premature ovarian failure with low serum oestradiol and high FSH levels. Questionnaire evaluation showed four patients, three in the control and one in the study group, retained their menstrual cycles, and three of these had positive ovulation tests.

**Conclusions** Goserelin did not prevent chemotherapy-induced premature ovarian failure in women with breast cancer. However the results are inconclusive as patients in the goserelin group were perimenopausal. The study should be repeated prospectively in a larger sample of patients below 40 years old.

### P46 CHEMOTHERAPY USAGE IN WOMEN UNDER 35 WITH BREAST CANCER

Richard Sainsbury* and Bob Haward, Huddersfield Royal Infirmary HD3 3EA and NYCRIS University of Leeds LS16 9QB, UK

**Introduction** There is debate as to whether all young women require chemotherapy. Young age is often taken as an independent risk factor although it is known to be associated with an excess of Grade 3 lesions. A recent paper from Denmark (1) suggests that survival rates can be improved if all women under 35 receive chemotherapy.

**Methods** We used the Yorkshire Cancer Registry to look at chemotherapy rates for the years 1980–1994 and examined at the relationship with survival.

**Results** 1534 patients under 35 were treated over this time of whom 304 (19.8%) received adjuvant chemotherapy.

The 5-year overall survival for this group was 60% (95% confidence intervals 54.8–65.8) for those receiving chemotherapy and 63% (60.6–66.0) for those who did not. Forty-one patients presented with overt metastatic disease, if they are excluded from the analysis the 5-year survival rates increased to 63% and 64% respectively. The paper from Denmark does not give 5-year survival rates and thus direct comparisons of 5-year survival are not possible.

There was no significant improvement in survival for those receiving chemotherapy for either the individual time cohorts or the group as a whole. A Wilcoxon (Breslow) test for equality of survivor functions showed no significant differences between the groups receiving chemotherapy and those who did not (p=0.31). The percentage of chemotherapy usage in this age group increased from 8% in the years 1980–84 to 17% in 1985–1989 and to 32% in 1999–1994.

**Conclusion** The amount of chemotherapy given over this time period was low but increased significantly year-on-year. There is no evidence from our large data set that the addition of chemotherapy affected five-year survival rates.

1 Kroman N, Jensen M-B, Wohlfahrt J, et al (2000) Factors influencing the effect of age on prognosis in breast cancer: a population based study. *BMJ* **320:** 474–9

### P47 CORE BIOPSY IN BREAST CANCER – AN ANALYSIS OF THE OPTIMUM NUMBER OF CORES FOR ACCURATE ASSESSMENT OF HISTOLOGICAL GRADE

C McIlhenny*[1], JC Doughty[1], WD George[1], EA Mallon[2], [1]University Department of Surgery, [2]Department of Pathology, Western Infirmary, Glasgow, G11 6NT, UK

**Aims** Core biopsy is well established in the investigation of breast disease and is increasingly used in patients with locally advanced breast cancer prior to commencing neoadjuvant chemotherapy. Histological grade is one of the important prognostic factors in these patients, and accurate assessment of this by core biopsy is important in predicting outcome. To date no author has reported on the number of cores required to accurately grade a tumour pre-operatively. This prospective single-blind study was carried out to determine the number of passes required to establish both a histological diagnosis of cancer and for accurate determination of the grade of the tumour.

**Methods** Twenty mastectomy specimens were examined immediately after surgery. These were orientated as they would have been in vivo and biopsied percutaneously. Ten consecutive core biopsies were taken using a spring-loaded device and labelled sequentially. Sections were then labelled randomly, to blind the investigators. The tumour was then processed for final histology.

**Results** A single Core Biopsy achieved an accurate histological diagnostic rate of 73.7%. This figure rose to 89.5% with a second pass and did not become any more accurate with subsequent passes. To accurately reflect tumour grade, a single needle pass predicted grade in only 31.6%. This rose to 68.4% for three needle passes, and to 73.7% for four passes. These differences were statistically significant ($p < 0.05$).

**Conclusions** From our study, two core biopsies are sufficient to accurately diagnose breast disease, and additional passes do not increase this figure. If core biopsies are being used to grade a tumour, then a minimum of 4 cores are recommended for a diagnostic accuracy of 75%.

### P48 PATTERNS OF SUPRACLAVICULAR FOSSA FAILURE IN EARLY BREAST CANCER

NJ Thorp*, I Syndikus, Dept. of Radiotherapy, Clatterbridge Centre for Oncology, Wirral, Merseyside CH63 4JY, UK

Isolated supraclavicular fossa (SCF) relapse following treatment for early breast cancer is a rare event but can be difficult to manage and impacts on the individual's quality of life. It is common practice to treat this area with prophylactic adjuvant radiotherapy in high risk patients. This requires an anterior field to be matched to the tangential fields, leading to a higher risk of radiation related toxicity. Some authors therefore dispute the need for routine SCF irradiation. Until recently the standard radiotherapy technique used at our centre for the adjuvant treatment of early breast cancer employed two long tangential fields with no additional anterior field. In vivo dosimetry studies have demonstrated an adequate axillary dose but a subtherapeutic dose to the SCF.

We undertook a retrospective study of patients with early breast cancer treated using this technique in 1993 recording tumour and treatment characteristics. We then determined the overall rate of SCF relapse and the rate of isolated SCF relapse in this group of patients in whom the SCF was suboptimally treated.

A total of 361 patients were identified. There were fourteen cases of ipsilateral SCF recurrences (3.9%) of which 6 had distant metastases diagnosed at the time of the SCF relapse. The remaining 8 patients had isolated SCF disease at the time of diagnosis but 1 patient developed distant metastases 19 months later. Of the 7 patients with distant metastases 6 have died and 3 of the 7 with isolated SCF recurrence are dead. Hence 4 of the 7 patients with isolated SCF recurrences remain alive and disease free and 1 patient with associated bone metastases remains alive 24 months following the diagnosis of SCF disease.

This study confirms the low rate of isolated SCF recurrence in patients who have not received prior optimal radiotherapy to the SCF. However, 3 of these patients died with no other sites of documented disease, suggesting an impact on survival. Data remains sparse and a large randomised controlled study would be required to clarify the situation.

### P49 ONCOLOGICAL SAFETY OF SKIN SPARING MASTECTOMY AND IMMEDIATE RECONSTRUCTION BY BREAST SURGEONS

KL Kirkpatrick, C Choy, ACA Leris, K Mokbel, N Williams, C Kouriefs, A Mostafa and R Carpenter, St. Bartholomew's Hospital, West Smithfield, London EC1A 7BE, UK

**Aims** To assess the oncological safety, complication rate and patients' satisfaction after skin sparing mastectomy (SSM) and immediate reconstruction for breast cancer.

**Methods** We retrospectively reviewed the records of 136 patients who had Becker implant (N=100), Latissimus Dorsi Flap (N=28) or TRAM Flap (N=8) breast reconstruction after skin sparing mastectomy. All data was collected prospectively as part of continuing audit of reconstructive procedures at this institution.

**Results** The median age was 52 years (27–74). After a median follow-up of 4 years, there were 6 locoregional recurrences, 20 breast cancer related deaths and one death unrelated to breast cancer. The overall implant loss rate was 18% (23/128). There was no flap loss, however recipient breast skin necrosis occurred in 2 patients and wound infection was observed in 19 patients (14%). 87% of patients were satisfied with the cosmetic outcome (median satisfaction score=9, range 0–10). Two patients who underwent TRAM flap reconstruction developed incisional hernias (25%). Capsule formation occurred in 35 patients and capsulotomy was performed in these 35.

**Conclusions** SSM with immediate reconstruction by breast surgeons appears to be an oncologically safe procedure and is associated with satisfactory cosmesis at 4 years. However the complication rate is significant and should be taken into consideration when consenting patients during surgical budget planning.

### P50 DOES RADIOTHERAPY IMPACT ON THE OUTCOME OF IMPLANT-ONLY AND LD FLAP/IMPLANT RECONSTRUCTIONS?

Landau D*[1,3], Sacks N[2,3], Gui G[2], A 'Hern R[4], Ross G[1,3], Depts. of [1]Radiotherapy, [2]Surgery, and [4]Computing, The Royal Marsden Hospital, London, and [3]St. George's Hospital, London, SW3 6JJ, UK

**Background** The impact of adjuvant post-mastectomy radiotherapy (RT) on disease-free and overall survival in breast cancer has been demonstrated in two recent randomised trials. With increasing use of immediate breast reconstruction there has been variability in referral for locoregional RT. The aim of this study was to investigate the impact of RT on the frequency of surgical revision of breast reconstructions, either implant-only or latissimus dorsi (LD) flap, after mastectomy.

**Procedure** Notes were reviewed of patients who had undergone postmastectomy reconstruction by NS and GG to identify the frequency of removal of prostheses or other surgical revision of the reconstructions. Indications for mastectomy, type of reconstruction, details of prior or subsequent RT and details and timing of surgical revision were recorded.

**Major findings** 216 patients underwent 253 reconstructions from August 1994 to April 1999. 199 reconstructions were immediate and 17 delayed, 129 were implant only and 124 latissimus dorsi flap and implant. The indications for mastectomy were multifocal invasive disease (63 breasts), DCIS (47), prophylactic (44), recurrent invasive carcinoma (37), advanced disease (17), extensive intraductal component (14), retroareolar tumour (9), positive margins (5), patient choice (5), inflammatory carcinoma (4), unknown (4), palliative (2), large phylloides tumour (1) and patient not suitable for RT (1). Median follow-up was 14 months. 50 reconstructions were on previously irradiated chest walls and another 40 went on to receive RT. All patients received either 2 Gy/# to 46–60 Gy, 40 Gy in 15# or 45 Gy in 25#. 34 implants were removed and 10 required other surgical revision.

The addition of RT to LD flap reconstruction did not increase the need for surgical revision (11%). For the implant only group the surgical revision rate was 41% with RT vs 18% without RT (p=0.049). Timing of RT and RT dose did not affect the revision rate. The mean time to implant removal was 8 months (range 0.3–55) for all cases; for infected prostheses the mean time to removal was 2 months and for capsule/cosmesis/pain 11 months. 59% of removals were within 6 months and 79% within 12 months post-op.

**Conclusion** RT has previously been shown to increase the revision rate following implant-only reconstruction. We have demonstrated that this is not the case for LD flap reconstructions. Possible explanations for this difference are improved vascularity of the periprosthetic tissue and increased 'padding' with the LD flaps compared with implant-only reconstructions.

### P51 THE ROLE OF ADJUVANT RADIOTHERAPY IN SMALL INVASIVE BREAST CANCERS

JCC Hu, ACA Athow, ACA Leris, NJ Williams, C Kouriefs, C Choy, C Wells, K Mokbel and R Carpenter, St. Bartholomew's Hospital, West Smithfield, London ECIA 7BE, UK

**Aims** To analyse outcome of patients with small tumours (less than or equal to 1 cm), managed by breast conserving surgery with or without radiotherapy.

**Patients and methods** 110 patients with tumours equal to or less than 1 cm in maximum diameter were identified from the St. Bartholomew's Hospital database. The notes, histology reports and mammograms were retrospectively reviewed. During this period policy on the management of small tumours changed and those less than 1 cm in diameter with negative axillary lymph nodes and no adverse histological features were managed with breast conserving surgery alone; i.e. no adjuvant radiotherapy was given. The final surgical margins were clear in all cases in both groups.

**Results** The median age at diagnosis for patients treated by local resection alone (group 1) was 59 years (45–81) and in group 2, 57 years (38–80). Median tumour size was 7 mm in group 1, and 9 mm in group 2. Seventy-eight (71%) of 110 patients underwent axillary surgery (clearance or sampling). Four patients were found to have lymph node metastases, all of whom received radiotherapy. After a median follow-up of 4 years for group 1, there were 2 local recurrences, these occurred at 4 and 6 years from date of diagnosis. In group 2 after a median follow up of 6 years there were no local or distant recurrences.

**Conclusions** We conclude that a highly selective policy of avoiding the use of adjuvant radiotherapy in small, invasive, node-negative breast cancers without adverse histological has an acceptably low local recurrence rate. These patients require continued follow up to ensure local control is maintained long term.

### P52 INCREASED BREAST TEMPERATURE IN THE 'AT RISK' BREAST

L Hayes*[1], P Wilson[2], J Affen[2], R Greenhalgh[2], J Cooley[5], DG Evans[3], L Tetlow[4], A Howell[2], Departments of Surgery[1], Medical Oncology[2], Genetics[3], Biochemistry[4], Physics[5], University Hospital of South Manchester, Nell Lane, Manchester M20 2LR, UK

Breast surface temperature rises during the luteal phase of the menstrual cycle to a greater extent than core body temperature.[1] We wished to test the hypothesis that this breast specific temperature might be higher in women at increased risk of breast cancer. We have therefore compared the breast temperature throughout the luteal phase of the menstrual cycle in 12 control breasts and in 8 breasts at risk related to contralateral breast cancer.

**Patients and methods** Brassieres fitted with 12 temperature sensors were supplied by Professor Hugh Simpson in Glasgow. The sensors were worn for 90 minutes each evening through one luteal phase. Subjects also measured their daily oral temperature and collected daily saliva for progesterone assay. Breast temperature data were analysed by fitting a cosine function with a period of 14 days to the grouped data enabling the two series to be summarised into three parameters – phase, amplitude and level. Cycles were synchronised based on progesterone peak. Two sets of data from the controls were excluded due to presumed instrument fault or poor subject compliance.

**Results** The contralateral breasts of patients with a history of breast cancer showed a higher mean breast surface temperature (35.90° C) than the controls (35.41° C), the amplitude of cyclical temperature fluctuation was significantly reduced in the contralateral breasts (0.127° C compared to 0.255° C) and there was a lower probability of detecting a luteal rhythm in these breasts.

**Conclusion** These results support the data of Simpson et al[1] and suggest a real difference in the temperature profile of the contralateral breast in breast cancer patients, independent of body temperature. This leads to the hypothesis that a 'high risk' breast may also exhibit this type of temperature profile. The mechanism by which this alteration in local temperature is brought about is unclear but is likely to be a result of local hormonal influences over-riding basal body temperature. Further investigation of these phenomena is warranted.

1 HW Simpson, K Griffiths, C McArdle, AW Pauson, P Hume, A Turkes (1993) The luteal heat cycle of the breast in health. *Breast Cancer Research & Treatment* **27:** 239

### P53 MAPPING STUDY OF A POST-MORTEM LUNG SHOWS GENETIC CHANGES OF CANCER IN ADJACENT BRONCHIAL EPITHELIUM

M Ocejo-Garcia*, JM Coulson, PJ Woll, CRC Academic Department of Clinical Oncology, Nottingham City Hospital NG5 1PB, UK

Lung cancer is believed to develop through an accumulation of genetic alterations which lead to morphological changes of increasing disorder. Deletions, shown by loss of heterozygosity (LOH) at polymorphic loci on different chromosomes, are frequently seen in both small cell lung cancer (SCLC) and non-small cell lung cancer (NSCLC). Here we investigated genetic alterations in samples taken during autopsy at specific distances from the tumour in a lung from a female heavy smoker (20 cpd, 50 years) with SCLC. This kind of mapping study is often difficult to undertake in SCLC since post-mortem samples are rarely available.

**Methods** We compared the alterations found in the tumour with its corresponding pre-invasive lesions. The samples analysed included a tumour sample, bronchial epithelium at 1 cm and at 5 cm from the tumour and normal lung tissue from the other healthy lung. After genomic DNA extraction of the human samples, these were analysed for LOH at a number of microsatelite loci by radioactive ($\gamma^{32}P$) PCR and gel analysis. Microsatellite markers included D3S4103 within the *FHIT* gene at locus 3p14.2, D3S1289 at locus 3p21, D3S1293 at locus 3p25, D13S153 within the retinoblastoma (RB) gene at locus 13q14.3 and D17S513 within the p53 gene at 17p13.1. Results were visualised by autoradiography.

**Results** Normal lung and distant (5 cm) samples were heterozygous for the microsatellite markers studied whereas their corresponding tumour sample showed LOH for the same markers. The sample adjacent to the tumour (1 cm) showed LOH for the microsatellite marker D3S4103 located within the *FHIT* gene (3p14.2). The same allele was shown to be lost in the tumour and its paired adjacent (1 cm) sample indicating a common clonal origin.

**Discussion** Loss of function of the tumour suppressor *FHIT* has been previously reported to be common in lung cancer and especially frequent in smokers. It has also been suggested as a marker for the early detection of lung cancer (Sozzi *et al.*, Cancer Research, v.58, p.5032; 1998). Using a mapping approach, we have shown that normal bronchial epithelium adjacent to the tumour shows LOH at the microsatellite marker D3S4103, suggesting a field effect of carcinogens. We propose that LOH at this locus could be used as a potential early marker for the detection of SCLC. The understanding of tumour development will enable its early detection, an improved staging of lung cancer and the identification of new therapeutic targets. We are currently expanding this study to include a larger number of patients and further markers.

### P54 MULTICENTRE PHASE II STUDY OF CARBOPLATIN (AUC 5) AND VINORELBINE IN SMALL CELL LUNG CANCER (SCLC)

DJ Dunlop*[1], ME O'Brien[2], SM Lee[3], S Hill[4], N Thatcher[5], [1]ST Mungo Institute, Glasgow, [2]Royal Marsden Hospital, London, [3]Middlesex/UCL Hospital, London [4]Pierre Fabre Ltd, Winchester, [5]Christie Hospital, Manchester, UK

Based on existing data suggesting efficacy and tolerability of carboplatin and vinorelbine in poor prognosis SCLC, (Gridelli et al, 1997. Proc Am Soc Clin Oncol 16, ab 1620), 4 centres in the UK collaborated to establish further safety and efficacy using an AUC calculated dose of carboplatin, (AUC×5)mg intravenously on d1 q28 days, and vinorelbine 30 mg/m² on days 1 and 8 of a 28 day cycle. Chemonaive patients with an ECOG performance status 0–2 were eligible with poor prognosis SCLC or good prognosis disease medically unfit for intensive chemotherapy. Dose modifications were based on nadir blood counts on treatment days or grade 3/4 myelosuppression morbidity. Evaluable patients for response had to complete 2 cycles of therapy. All patients were evaluable for toxicity and survival. 58 patients were enrolled over a 12 month period. Patient characteristics included; mean age 65 yrs, (range 45–86 yrs), 39 patients had poor prognosis disease, 19 patients had good prognosis SCLC, mean ECOG performance status was 2. 10 patients were inevaluable for response due to early withdrawal due to disease progression or toxicity. 202 cycles of therapy were administered. 50% of patients experienced WHO grade 4 neutropenia but over the whole study ther were only 3 septic deaths. Only 4 patients (7%) experienced WHO grade 3/4 platelet toxicity, all of these cycles being uncomplicated. Non-haematological toxicity was observed in <5% of patients, (3/58 grade 3/4 nausea/vomiting, 2/58 grade 3 alopecia, 1 patient grade 3 diarrhoea, 1 patient grade 3 neuropathy). 6/48 (12.5%) achieved CR, 20/48 (42%) achieved PR, 10/48 (20.5%) had SD, and 12/48 (25%) had PD. The overall response rate was 54.5%. Median survival for all patients was 26 weeks, (95% confidence interval 15–37 weeks). 17 patients are still alive.

**Conclusion;** The regimen is well tolerated with good clinical efficacy comparable to that reported with other combinations. Despite significant myelotoxicity, morbidity related to this was minimal. This AUC calculated dose of carboplatin produced significantly more myelosuppression than the mg/m² dose used in previous studies.

### P55 PHASE II STUDY OF OCTREOTIDE PLUS STANDARD CHEMOTHERAPY IN THE TREATMENT OF SMALL CELL LUNG CANCER

MP Decatris*, A Thomas, L Fresco, K Jeffries, S Conray, A Benghiat and KJ O'Byrne, University Dept Oncology, Leicester Royal Infirmary LE1 5WW, UK

Octreotide is an octapeptide somatostatin analogue routinely employed in the treatment of neuroendocrine tumours. Octreotide inhibits small cell lung cancer (SCLC) growth *in vitro* and *in vivo*. Recent work has shown that somatostatin analogues enhance the cytotoxicity of chemotherapy agents whilst reducing their side effects. We are evaluating the efficacy and tolerability of Octreotide, 100 mcg tds s/c daily, in combination with standard cytotoxic chemotherapy – (adriamycin, cyclophosphamide, etoposide [ACE]; vincristine, adriamycin, cyclophosphamide; [VAC]; cisplatin, etoposide [CE]) in previously untreated patients with SCLC in an ongoing phase II study. Octreotide is continued for 6 months. Eleven patients (pts), 9 male and 2 female, median age 61 years (range 50–73) and median ECOG performance status = 1, have been recruited to date. Seven pts had extensive and 4 limited disease. The 11 pts have received a total of 47 cycles of chemotherapy. Neutropenic pyrexia occurred in 4 patients on one occasion only (9% of treatment cycles). Other grade 3/4 toxicities seen were nausea (1 pt), vomiting (1 pt), diarrhoea (1 pt), constipation (1 pt), lethargy (1 pt), anaemia (1 pt), and thrombocytopenia (1 pt). Three patients with bulky disease and tumour obstruction of the main airway developed evidence of pulmonary abscess formation in the right upper lobe. After a median of 4 chemotherapy cycles (range 1–6), 9 of 11 pts were evaluable for response. A radiological objective tumour response was seen in 8 pts (2 CR and 6 PR). The remaining patient had stable disease after 3 cycles of VAC chemotherapy. Two pts have died, one 8 months after initial treatment while on second line chemotherapy for relapsed disease and the other with a pulmonary abscess whose general condition deteriorated after 2 cycles of chemotherapy. After a median follow up time of 4 months, 9 patients are alive of whom 2, with limited disease at presentation, are disease free (1 by CT scan and 1 by CXR). These preliminary results suggest that the combination of Octreotide with standard cytotoxic chemotherapy regimens has acceptable toxicity and activity in SCLC justifying continued recruitment of patients to the study.

### P56 WEEKLY CHEMOTHERAPY IN POOR PROGNOSIS SMALL CELL LUNG CANCER (SCLC): A SINGLE CENTRE PHASE II TRIAL

HE Innes*, DB Smith, PI Clark and E Marshall, Department of Medical Oncology, Clatterbridge Centre for Oncology, Merseyside CH63 4JY, UK

Many patients with small cell lung cancer present with poor prognostic features (poor performance status and/or extensive disease). Current treatment for this group with the CAV regimen (cyclophosphamide, doxorubicin and vincristine) remains unsatisfactory; median survival is typically only 6 months but associated with significant toxicity. Studies have also highlighted a risk of early mortality (up to 10%) following the first cycle of chemotherapy, possibly related to sepsis.

VAPEC-B is a weekly low dose combination chemotherapy initially developed for intermediate grade non-Hodgkin's lymphoma (NHL). We have found this regimen it to be particularly well tolerated by patients irrespective of age or performance status and weekly administration enables close monitoring of patients. It also appears to be as effective as CHOP chemotherapy in NHL, a regimen that is almost identical to CAV. We have conducted a phase II study using a modified version of this regimen consisting of: adriamycin 35 mg/m² weeks 1 and 3, cyclophosphamide 350 mg/m² week 1, vincristine 1.4 mg/m² weeks 2 and 4 and etoposide 50 mg bd for 5 days in week 3. This is repeated 3 times to give a total duration of 12 weeks. All patients receive prophylactic ciprofloxacin and a reducing schedule of oral steroids.

Entry criteria included: patients with poor prognostic features but considered eligible for palliative chemotherapy, creatinine <1.5 × normal, bilirubin <2.5 × normal, measurable disease and no CNS disease. Primary end-points were response rate and toxicity with time to disease recurrence and overall survival as secondary end-points. During the first phase 12 eligible patients were entered (March to August 1999). The median age was 68 y (57–77 y). 8 of the patients were poor performance status; 4 were good performance status but were elderly and/or had extensive stage disease.

Treatment was generally poorly tolerated. Only 1 patient completed treatment according to the planned schedule, the rest terminated the regimen early and/or required treatment delays or dose reductions. The median duration of treatment was 6 weeks (3–12). Reasons for early discontinuation were toxicity (4), progressive disease (3) and early death (2). Two patients were converted to alternative treatment (1 to CAV and 1 to oral etoposide). The main toxicities were haematological, 5 patients having grade 3 or 4 neutropenia although only 1 required hospitalisation. Mucositis was generally mild, with only 1 patient having ≥ grade 3. 10 patients were assessable for response. There was 1 complete response, 6 partial responses and 3 patients with progressive disease. The median overall survival was 26 weeks.

In summary, weekly low dose chemotherapy does not appear to improve tolerability or survival in this group of patients with small cell lung cancer with poor prognostic features. Of note is that the median survival is comparable with previously published data despite the very brief median duration of treatment.

### P57 A RANDOMISED PILOT STUDY OF SRL172 (MYCOBACTERIUM VACCAE) IN PATIENTS WITH SMALL CELL LUNG CANCER (SCLC) TREATED WITH CHEMOTHERAPY

L Assersohn[1], BE Souberbielle[3], L Spencer[1], R Bass[2], R Mendes[1], IE Smith[1], MER O'Brien[1,2], [1]Royal Marsden NHS Trust, Sutton, Surrey, [2]Kent Cancer Centre, Maidstone, Kent, [3]Dept of Molecular Medicine, King's College, London, UK

**Introduction** SRL172 is a preparation of heat killed *Mycobacterium vaccae*. When used with autologous cells in animal models, it proved to be a very potent immunological adjuvant. In human studies, promising results with SRL172 have been obtained in NSCLC and mesothelioma when used in combination with chemotherapy. An interim analysis was performed in July 1999.

**Methods** Patients were randomised to receive chemotherapy with (n=14) or without (n=14) SRL172. The chemotherapy was either platinum-based (MVP, n=9) or anthracycline-based (ACE, n=19). SRL172 was given intradermally on Day 0, weeks 4, 8 and then 3–6 monthly. The response was assessed using CT scanning every 2 courses.

**Results** The treatment arms were well balanced with respect to disease extent (LD vs ED). A median survival of 8.5 months was obtained with chemotherapy alone and 13.8 months with chemotherapy and SRL172 (p=0.07). The trend was the same with both chemotherapy regimens in combination with SRL172. 26 of the 28 patients in this pilot study have now died.

**Conclusions** In this pilot study with SCLC patients, the trend seen in improved median survival, with the combination of chemotherapy and SRL172, is the same as that previously reported for NSCLC and mesothelioma patients. This trend was seen for both platinum- and anthracycline-based regimens. The final report will be available for the meeting.

We are grateful to SR Pharma for their support.

### P58 IS THERE A RELATIONSHIP BETWEEN PRE-TREATMENT HAEMOGLOBIN AND OUTCOME IN NON-SMALL CELL LUNG CANCER TREATED WITH RADIOTHERAPY?

A Sibtain,* N Shah, MI Saunders, PJ Hoskin, Marie Curie Research Wing, Mount Vernon Hospital, Northwood HA6 2RN, UK

**Introduction** There is accumulating evidence that haemoglobin levels correlate with outcome in head and neck, bladder, anal, prostate, and cervical cancer treated with radiotherapy. This effect may be due to impaired oxygen delivery in anaemic patients, increasing tumour hypoxia and thus radioresistance, although other mechanisms may also be implicated e.g. Haemolysis, malnutrition, advanced disease etc.

**Aim** To assess the effect of the pre-treatment haemoglobin level in patients with non-small cell lung cancer treated with radiotherapy.

**Methods and Materials** A retrospective review of patients treated with radical radiotherapy for non-small cell lung cancer between 1990 and 1999, analysing pre-treatment haemoglobin levels, disease-free and overall survival for each presenting stage and for each sex.

**Results** In 165 identified patients, the median age was 66 (range 35–78) years and the sex ratio was 123 male: 42 female. Thirty-two patients were stage I, 24 stage II, 55 stage IIIa and 54 were stage IIIb. Forty-six patients were treated by conventionally fractionated radiotherapy, 27 by CHART and 76 by CHARTWEL. Sixteen patients received chemotherapy and CHARTWEL. Median follow-up time was 16.2 months. One hundred and twenty-seven patients have died, and 4 are alive with disease. The median overall survival for male patients was 18.3 months and for females 16.4 months. Haemoglobin levels ranged from 17.4 to 10 g/dl in the male patients and from 16.6 to 8.6 g/dl in the female patients. 27% (33/123) of the males and 23% (10/42) of the females had a level below the lower limit of the normal range (13–17 g/dl) in males and 11.5–16.5 g/dl in females). There was no correlation between haemoglobin and local disease free survival (males r=0.10, females r=0.003), metastasis free survival (males r=0.11, females r=0.28), or overall survival (males r=0.12, females r=–0.46). No significant correlation coefficient emerged when each stage or method of fractionation was individually analysed, except in females with stage I disease, when a negative correlation was found (r=–0.81). Kaplan Meier survival analyses revealed no significant differences in disease-free or overall survival when the patients were classified by the haemoglobin level being either within or below the normal range.

**Conclusion** No correlation between pre-treatment haemoglobin has been observed in this cohort of patients, implying factors other than anaemia-related hypoxia influence treatment outcome.

### P59 DOES TREATMENT DELAY AFFECT SURVIVAL IN NON-SMALL CELL LUNG CANCER (NSCLC)? A RETROSPECTIVE ANALYSIS OF DELAY AND OTHER FACTORS

H Bozcuk, WMC Martin, Department of Oncology, Norfolk & Norwich Hospital, Norwich NR1 3SR, UK

The Joint Council for Clinical Oncology and British Thoracic Society have made recommendations on waiting times for patients with lung cancer[1,2] and the Department of Health is introducing from April 2000 a policy by which patients suspected of lung cancer are to be seen within two weeks, yet to date there is no evidence that early treatment is associated with improved survival.

**Objectives** We studied adherence to delay targets and survival in relation both to these targets and to other clinical and laboratory parameters.

**Subjects** A cohort of 189 NSCLC patients presenting in year 1998 to Norfolk & Norwich Hospital.

**Outcome Measures**

1. Waiting times in patients' pathway recorded were: time from GP to starting treatment, time from GP to first hospital appointment, time to see surgeon, time to surgery (from decision to operate), time to see oncologist, time to starting radiotherapy (RT) (from decision), time to chemotherapy from decision.
2. Other parameters recorded were patient and tumour demographic data, laboratory parameters, nature of treatment received and survival.

**Results** In multivariate analysis, waiting time to start treatment did not affect survival. Combined-modality treatment, laboratory abnormality, stage and bone/liver metastases were highly significant survival predictors (p<0.001, <0.001, 0.001, 0.007 resp). Median time to treatment was 50 days. Compliance with 4-week standard of time to surgery was 80% but most patients had waited over 7 weeks to see the surgeon. There was 53.5% compliance with 1-week standard to see oncologist, 42% with 4-week standard to starting radical RT, 62% with 2-week standard to palliative RT, 28% and 84% respectively with 1-week (good practice) and 3-week (max acceptable) to starting chemotherapy.

**Conclusion** Since combined treatment significantly improves survival, NSCLC results will be benefited more by improving resources especially oncology (equipment, drugs, personnel) and in thoracic surgery than by attempting to reduce waiting time to see a physician in 2 weeks.

1 Joint Council for Clinical Oncology (1993) Reducing delays in cancer treatment
2 The Lung Cancer Working Party of BTS. Roy Coll Phys London (1993) *Thorax* 1998; **53:**(Suppl 1) S1–8

### P60 PALLIATIVE CHEMOTHERAPY FOR MESOTHELIOMA – SIGNIFICANCE OF PERFORMANCE STATUS (PS)

RK Gregory*, MER O'Brien, S Ashley, IE Smith, Lung Unit, Royal Marsden Hospital (RMH), Fulham Road, London SW3 6JJ, UK

Chemotherapy can provide useful palliation in malignant mesothelioma (1). However not all patients benefit and the decision on who to treat is based on a number of factors including rate of progression of disease, PS and patient preference. Oncologists are often reluctant to offer palliative chemotherapy to patients of poor PS (>2).

**Methods** We performed a retrospective analysis of patients with mesothelioma treated at the RMH with palliative MVP (mitomycin C, vinblastine, cisplatin) with respect to PS, response to chemotherapy and time to disease progression.

**Results** Data were available for 94 patients. Of these 3 were PS 0, 53 were PS 1, 31 were PS 2 and 7 were PS 3. Overall objective response rate was 15%, symptomatic response rate was 71%. In particular, 6/7 of the PS 3 patients had a symptomatic response. Median duration of symptom relief was 4 months and there was no difference between the different PS groups. All patients had symptomatic progression within 18 months of commencing treatment, PS did not predict for rapid symptom progression following treatment.

**Summary** Symptomatic response to palliative chemotherapy in mesothelioma is high at >70% and the benefit does not appear to be confined to patients of good PS. It may be that patients who could potentially benefit from treatment are not being offered chemotherapy on the basis of a poor PS.

1 GW Middleton, IE Smith, MER O'Brien et al (1998) Good symptom relief with palliative MVP chemotherapy in malignant mesothelioma. *Annals of Oncology* **9:** 269–273

### P61 INVESTIGATION OF LOSS OF HETEROZYGOSITY AT THE FOLLOWING LOCI: 1P36, 5 Q(APC LOCUS), 7Q31, 17 P(P53 LOCUS) AND 18 Q(DCC LOCUS) IN HUMAN COLON, OESPHAGEAL AND STOMACH CANCER

RN Hourihan*[1], E Baird[2], GC O'Sullivan[3], JG Morgan[1], Departments of Microbiology[1], Medicine[2], and Surgery[3], National University of Ireland, Cork, Ireland

Simultaneous analysis of loss of heterozygosity (LOH) in adenomatous polyposis coli (APC), deleted in colon cancer (DCC) and p53 genes, in addition to microsatellite markers on chromosomes 1 p and 7 q has not yet been reported. The objective of this study was to determine the frequency of LOH at each of the 5 distinct loci. DNA was extracted from 68 patients' tumour and matched normal mucosa. 53%, 29% and 18% of these patients had colon, oesophageal and stomach cancers respectively.

A VNTR polymorphism (DCC) and RFLP analysis of Dde1 and BstU1 sites were investigated in order to determine heterozygosity of APC and p53 respectively.

It was shown that 40%(27/69), 14%(8/58) and 26%(11/41) were heterozygous for DCC, APC and p53 respectively. LOH analysis of the paired samples showed frequencies of 26%, 36% and 0% for DCC, p53, and APC in that order.

Regarding microsatellite markers, D1S228 on 1 p and D7S522 on 7 q, DNAPCR products were subjected to high-resolution spreadex electrophoresis, stained with syber green nucleic acid stain and illuminated at 254 nm. It was shown that 42%(26/62) were heterozygous for D1S228 and 54%(34/63) were heterozygous for D7S522. LOH analysis showed frequencies of 4%(1/26) and 9%(3/34) for D1S228 and D7S522 respectively. Two of the three D7S522 LOH positives were oesophageal samples. This is the first study to demonstrate LOH of microsatellites using this technique. LOH frequencies were confirmed using ABI prism 310 and gene scan analysis.

It was shown that two patients exhibited LOH at both DCC and p53. One patient was shown to display LOH at both 1 p and p53.

### P62 THE ANALYSIS OF COPY NUMBER CHANGES IN BARRETT'S OESOPHAGUS USING COMPARATIVE GENOMIC HYBRIDISATION

J Croft*[1], E Parry[1], J Baxter[2], T Brown[2], P Griffiths[2], JM Parry[1], [1]Centre for Molecular Genetics and Toxicology, UWS, Swansea, [2]Morriston Hospital, Morriston, Swansea, Wales

The technique of Comparative Genomic Hybridization (CGH) has been used to characterise genome wide changes that occur during the progression of Barrett's metaplasia through low-grade dysplasia (LGD) and high-grade dysplasia (HGD) to adenocarcinoma. Biopsies were obtained from 10 patients with LGD, 15 HGD and 5 adenocarcinomas. CGH allows the global analysis of the genome for net loss or gain of chromosome regions in a single hybridization, by competitive binding of test and genomic reference DNA to normal metaphase chromosomes. Hybridization of the DNA samples is visualised by the use of two different fluorochromes. The ratio of fluorescence intensities along each chromosome reflects the relative ratios of test and reference sequences. Thus, CGH produces a map of DNA sequence copy number as a function of chromosomal location.

The data demonstrates that high grade dysplasia is a stage of substantial karyotypic instability, with the amplification seen on 4 q present in 60% of samples. By contrast, adenocarcinomas show less widespread chromosome changes with amplification of chromosome 8 q being the most common feature, indicating the presence of multiple copies of the *myc-C* oncogene in the adenocarcinomas.

Despite ongoing efforts to characterize the molecular changes in Barrett's oesophagus, its pathogenesis remains poorly understood. It is hoped, that this work will lead to a greater understanding of the host/cell factors involved, subsequently helping to identifying a clinically useful marker for the progression of this condition and therefore allowing clinicians to stratify patients into high versus low risk groups.

### P63 SENESCENCE-ASSOCIATED BETA-GALACTOSIDASE ACTIVITY IN NORMAL, METAPLASTIC AND DYSPLASTIC MUCOSAE OF THE UPPER GI TRACT

AJ Fletcher-Monaghan*, M Downey, R Stuart, JJ Going and WN Keith, CRC Dept of Medical Oncology, University of Glasgow, CRC Beatson Labs, Glasgow G61 1BD, UK

Background: Increased beta galactosidase (BGal) activity at pH 6 has been associated with senescence of cells in culture and in vivo. Neoplastic transformation is associated with escape from senescence, and dysplasia and neoplasia could therefore be associated with downregulation of senescence-associated beta-galactosidase activity (SA-BGal). Aims: To investigate senescence-associated (SA) beta galactosidase activity in normal, metaplastic, dysplastic and neoplastic mucosae of the upper GI tract, including Barrett's oesophagus. Patients and methods. Histochemical staining for non-specific (pH 4) and SA-BGal (pH 6) was performed on frozen sections of 350 endoscopic biopsies from 46 patients (26 with long segment Barrett's oesophagus, 15 with gastric cancer, 2 with Barrett's adenocarcinoma and 3 with squamous oesophageal carcinoma). An intensity and extent-weighted staining score (range 0–6) was assigned to surface (luminal), intermediate and basal mucosal/epithelial compartments in all biopsies.

**Results** Squamous epithelium was consistently negative for SA-BGal. Strongest expression was detected in the luminal compartment of duodenal mucosa (mean score $3.7 \pm 0.5$, n=18), 'specialised' Barrett's mucosa (mean $2.2 \pm 0.12$, n=105) and gastric intestinal metaplasia (mean $2.37 \pm 0.40$. n=16). Neither low grade (n=5) nor high grade (n=3) Barrett's dysplasia correlated directly or indirectly with decreased SA-BGal activity but invasive adenocarcinomas showed significantly reduced activity (mean $1.3 \pm 0.29$, n=17), with no activity detectable in 6. Conclusions: SA-Bgal expression marks intestinal differentiation in the upper GI tract. It is not downregulated in dysplastic Barrett's mucosa but is in invasive gastric and oesophageal adenocarcinomas, and may therefore represent a late event in the oesopagogastric dysplasia/carcinoma sequence, associated with invasion.

### P64 ADENOCARCINOMAS OF THE GASTROOESOPHAGEAL JUNCTION AND DISTAL STOMACH ARE GENETICALLY DIFFERENT

SC Stocks[1,2], FA Carey[2], AM Thompson*[3], D Johnston[3], NM Kernohan[2], NR Pratt[1], [1]Departments of Human Genetics, [2]Molecular & Cellular Pathology, [2]Surgery & Molecular Oncology, University of Dundee DDI 9SY, UK

The incidence of adenocarcinoma arising at the gastro-oesophageal junction is rapidly increasing in the West and may reflect the increasing prevalence of Barrett's oesophagus. In contrast, the incidence of histologically similar cancers arising in the body and antrum of the stomach is declining. The epidemiological data suggest that the aetiology and pathogenesis of these cancers is distinct.

The aim of this study was to investigate the pattern of genetic abnormalities in adenocarcinomas of the gastrooesophageal junction (n=11) and distal stomach (n=17) by screening genomic DNA recovered from histologically confirmed frozen cancer samples. Regions of chromosomal imbalance (amplification or deletion) were determined by screening the genomic DNA by comparative genomic hybridisation (CGH).

Amongst the 28 cancers, genetic loss was frequent at chromosomes 17 p and 22 q while amplification was identified at 3 q, 5 q, 8 q, 12 q, 13 q, 20 p and 20 q. The most notable difference between the two tumour sites was at the DCC (Deleted in Colon Cancer) locus on 18 q which was deleted in 4/11 (36%) gastrooesophageal cancers but deleted in only 1/17 (6%) of the distal gastric cancers.

This novel difference between adenocarcinomas of the gastrooesophageal junction and distal stomach suggests a genetic mechanism may underlie the known epidemiological differences between these two tumour sites.

### P65 *GSTM1* AND *CYP1A1* POLYMORPHISMS AND INCIDENCE OF COLORECTAL ADENOMA

M Gunter[1]*, M Watson[2], S Bingham[1], W Atkin[3], N Day[4], A Loktionov[1], [1]MRC Dunn Human Nutrition Unit, Hills Road, Cambridge CB2 2XY; [2]Norfolk and Norwich Hospital; [3]ICRF, London; [4]Strangeways Research Laboratory, Worts Causeway, Cambridge, UK

GSTM1 and CYP1A1 metabolise a number of important environmental carcinogens and polymorphisms in these genes have been associated with several cancers (Zhong et al, 1993; Carcinogenesis, 14, p1821 and Sivaraman et al, 1994; Cancer Res, 54, p3692). There is controversy as to whether homozygosity for the *GSTM1* null allele confers increased risk for colorectal cancer and no evidence to date regarding colorectal adenomas. In addition, there is little information regarding association of *CYP1A1* exon 7 and *Msp* I polymorphisms with colorectal adenoma. Our aim was to investigate any association between these polymorphisms and incidence of colorectal adenoma.

**Methods** *GSTM1* genotypes were examined by RFLP analysis in 716 subjects with colorectal adenoma and 724 polyp-free controls. *CYP1A1* genotypes were similarly examined in 674 of these individuals from one arm of the cohort (319 cases, 355 controls).

**Results** *GSTM1* 63% of the cohort were homozygous for the *GSTM1* null allele, 37% displayed an active allele. Following statistical analysis, an unadjusted OR of 1.20 (95% confidence interval: 0.97–1.49) was observed for null vs active alleles. Individuals carrying at least one *GSTM1* A allele appeared to be protected: OR=0.76 (0.58–0.99)

*CYP1A1* As expected, the Msp I and exon 7 polymorphisms were in complete linkage disequilibrium. The putative high risk genotype Val/Val was rare (0.2%) in this group. 94% of individuals were Ile/Ile homozygotes, and 5.8% were Ile/Val heterozygotes. Carriers of the Val allele were not shown to be at risk of colorectal adenoma: OR=0.58 (0.30–1.10). Homozygous carriers of the *Msp* I polymorphism (m1/m1) were also rare (0.74%) in this group. 83.7% of individuals were homozygous carriers of the wild-type (w1/w1) allele and 15.6% were heterozygotes. Possession of the putative high risk allele did not confer increased risk of adenoma: OR=0.84 (0.56–1.27).

**Conclusions** Any associations between *GSTM1* and *CYP1A1* polymorphisms and risk of colorectal adenoma appear to be weak. The analyses of interactions between these genes with various lifestyle variables, such as diet are in progress.

### P66 EPIDERMAL GROWTH FACTOR RECEPTOR EXPRESSION IN PRIMARY COLORECTAL CANCER AND SECONDARY LYMPH NODE METASTASES

*McKay JA, Murray LJ, Ross VG, Curran S, Murray GI, Cassidy J, McLeod HL, Department of Medicine and Therapeutics, and Pathology, University of Aberdeen, Aberdeen, AB25 2ZD, UK

Colorectal cancer is a common malignancy, and at present patient prognosis is determined primarily by extent of tumour spread at presentation. However, patients with an identical pathological disease stage can display widely differing outcomes in terms of survival and response to chemotherapy. Therefore it is fundamentally important to identify molecular markers of more aggressive tumour phenotypes in order to adjust patient treatment accordingly. The epidermal growth factor receptor (EGFR) is a major regulator of several distinct, diverse cellular pathways, and a wide range of human tumours overexpress EGFR *in vivo*. EGFR is the focus of various anticancer agents currently under investigation, but little information is available on EGFR expression in colorectal cancer. To assess EGFR as a target for colorectal cancer, this study examined EGFR protein expression in a large series of 249 primary colorectal cancer samples for which uniform clinicopathological data is available. EGFR expression in lymph node metastases from a subset of 42 patients was also evaluated to examine the role of EGFR in invasion. We have used immunohistochemistry to determine the *in situ* localisation of EGFR protein within these samples. Seventy three percent (181/249) of primary tumours displayed EGFR immunostaining, the majority of which showed immunoreactivity in >50% of tumour cells (123/181 tumours). EGFR expression was localised primarily to the cell membrane (73/181, 40%), in some cases accompanied by cytoplasmic staining (94/181, 52%). In a small number of samples, immunoreactivity was observed solely in the cytoplasm of tumour cells (14/181, 8%). Using preliminary outcome data, patients whose tumours expressed EGFR protein were found to have a significant survival advantage ($P<0.05$), which remained following adjustment for Dukes' stage. In addition, the degree of EGFR expression in colorectal tumours was correlated with differentiation grade ($P<0.05$), with poorly differentiated tumours tending to express EGFR to a lesser degree than moderately/well differentiated tumours. No relationship was observed between EGFR expression levels and Dukes' stage, site or age of the patient. EGFR protein expression was observed in 22/42 (52%) of the lymph node metastases, and using a 5% cut-off point for positive expression, 25/42 (60%) samples showed agreement with the corresponding primary colorectal tumour ($P>0.05$). The majority of non-concordant cases (15/17, 88%) showed EGFR expression in the colorectal but not the lymph node tumour, suggesting that loss of EGFR protein may be involved in the metastatic process. Ongoing work will update the survival information available, and also investigate any relationship between EGFR protein and possible interacting factors (e.g. c-erbB-2, β-catenin) in colorectal cancer.

### P67 DUAL-PROBE IS MORE EFFECTIVE THAN SINGLE-PROBE CRYOTHERAPY FOR HEPATIC METASTASES

Huang A*, Mathur P, McCall J[1], Weston M[2], Quinn H, Glover C, Henderson DC[3] and Allen-Mersh TG, Department of Surgery, [1]Radiology, [2]Anaesthesiology & [3]Immunology, Chelsea & Westminster Hospital, 369 Fulham Rd, London SW10 9NH, UK

**Aims** Cryotherapy for colorectal liver metastases may result in a fall in serum carcinoembryonic antigen (CEA) that is thought to be predictive of survival. Cytokines are released that may be beneficial in stimulating the immune system leading to an antitumour response. This study determined whether dual- or single-probe percutaneous cryotherapy was more effective in reducing CEA and stimulating immune responses.

**Methods** Between July 1996 and December 1999 twenty-two single-probe treatments were given to 15 patients and fourteen dual-probe treatments to 7 patients with unresectable colorectal liver metastases. Single-probe cryotherapy was performed with either 3.6 mm or 6.3 mm probes (ice-ball volumes 18 $cm^3$ & 59 $cm^3$ respectively; Spembley Medical Ltd); dual-probe was performed with two 6.3 mm probes simultaneously (ice-ball volume 205 $cm^3$). Each percutaneous treatment was performed for one freeze/thaw cycle (20 mins each) under computed tomography guidance. Serum levels of CEA, interleukin 2 soluble receptor α (IL-2sRα), soluble necrosis factor receptor I (sTNF RI), interleukin 6 (IL-6) and C-reactive protein (CRP) were measured pre-operatively, at day 1, day 14, day 28 and at more than 40 days from cryotherapy.

**Results** Single-probe cryotherapy resulted in significant increases in IL-6 and CRP (p=0.001 & p<0.0001 respectively) that returned to basal levels by 14 days. The rise in sTNF RI was significant on day one (p=0.04) with no significant changes in IL-2sRα at any time. There was *no significant fall* in CEA and serum levels more than 40 days from cryotherapy were significantly *higher* than pre-treatment levels (p=0.008). In contrast dual-probe cryotherapy stimulated significant rises in IL-2sRα, sTNF RI, IL-6 and CRP (all p<0.05) on day one that returned to basal levels between 14 and 28 days after cryotherapy. These changes were accompanied by a significant rise in CEA (p=0.003) on day one that fell to *below pre-treatment levels* between 14 and 28 days (p=0.008 & p=0.005 respectively). CEA levels more than 40 days from cryotherapy were not significantly different from pre-treatment levels (p=0.5).

The rise in IL-2sRα in the dual-probe group was significantly higher than the single-probe group at 14 days (p=0.04) as was the increase in CRP at day one (p=0.02). The initial rise in CEA and subsequent fall between 14 and 28 days were significantly different between the two groups (p=0.0001, p=0.04 & p=0.003 respectively).

**Conclusion** Dual-probe compared with single-probe cryotherapy produced a significant fall in CEA suggesting the former was more effective at destroying tumour. Acute phase immune reaction as reflected by elevated IL-6 and CRP levels were seen in both groups but dual-probe cryotherapy also stimulated a significant IL-2sRα rise indicating a lymphocytic response. Thus a large ice-ball was necessary to reduce CEA and stimulate a lymphocytic response in the cryotherapy of colorectal liver metastases.

### P68 A PHASE II STUDY OF CAELYX™ (LIPOSOMAL DOXORUBICIN) IN THE TREATMENT OF ADVANCED GASTRIC CANCER

AL Thomas*, K Jeffries, WP Steward, KJ O'Byrne, University Dept of Oncology, Leicester Royal Infirmary, UK

Treatment of advanced gastric cancer remains disappointing. Anthracycline-containing chemotherapy regimes have response rates of 9–40% but are associated with significant toxicity. This is confounded by the fact that these patients (pts) are frequently elderly and have poor performance status. In other solid tumours (breast and relapsed ovarian cancer), Caelyx™, stealth liposomal doxorubicin, has documented objective response rates equivalent to standard single-agent therapies. In addition significantly fewer side-effects such as alopecia, nausea and vomiting, mucositis and neutropenic sepsis are seen. We conducted a phase II study to evaluate the efficacy and tolerance of Caelyx™ in advanced gastric cancer. Twenty previously untreated pts with locally advanced (4 pts; 20%) or metastatic (16 pts; 80%) gastric cancer have been enrolled. Median age: 70 years (range 41–82) and performance status (WHO) 0 in 6, 1 in 9 and 2 in 5 pts. Caelyx™ was administered at 45 $mg/m^2$ IV every four weeks. To date median event free survival in all 20 pts is 63 days (range 1–291 days) and median overall survival is 155 days (range 16–415). A total of 63 cycles have been given with a median of 3 cycles per pt. All pts were evaluable for toxicity. Although grade 3 nausea was seen in 1 pt, nausea and vomiting were rare metoclopramide being used as emesis prophylaxis. Grade 3 constipation was seen in 1, grade 3 mucositis in 1, grade 3/4 lethargy in 2 and an allergic drug reaction in 1 pt. The most severe side-effect were grade 3 pneumonitis in 1 pt and grade 4 hand-foot syndrome in another. No other grade 3/4 toxicities were seen including alopecia or myelosuppression. Fifteen pts were evaluable for response: 1 pt had a partial response, 6 stable disease and 8 progressive disease. The 5 pts not evaluable for response included 1 pt with an allergic reaction to the Caelyx™ infusion who was withdrawn from the study, 3 with clinical deterioration after 1 cycle and a fifth who awaits initial re-staging. In conclusion Caelyx™ is safe and well tolerated in pts with advanced gastric cancer. Survival data are comparable to other regimens but with the advantage of minimal toxicity. Recruitment is ongoing and updated information will be presented.

### P69 THE COMBINATION OF RALTITREXED (TOMUDEX) AND MITOMYCIN-C IN THE TREATMENT OF ADVANCED COLORECTAL CANCER: A PHASE-II-STUDY

JE Michels[1], M Harvey[1], A Darby[1], AM Iveson[2], L Nokes[1], TJ Iveson[1,2], Royal South Hants Hospital, Southampton[1], Salisbury District Hospital, Salisbury[2], UK

5FU based chemotherapy is now established treatment for advanced colorectal cancer. There is a move to combination treatment and the combination of Mitomycin-C with infusional 5FU has been shown to be superior to infusional 5FU alone. The specific thymidylate inhibitor Raltitrexed has been shown to have equal efficacy to bolus 5FU and folinic acid.

This phase II study therefore evaluated the combination of Raltitrexed (3 mg/m$^2$ iv every 3 weeks) with Mitomycin C (7 mg/m$^2$ every 6 weeks) as chemotherapy treatment for advanced colorectal cancer. Patients initially received 12 weeks treatment before assessment with CT scan and those responding received a further 12 weeks treatment. It was planned to enter 29 patients into the study but the study was closed after 22 patients had been entered because of 3 unexpected deaths. Patient characteristics were as follows: 15 male and 7 female with a median age 64 years (range 27–75). 15 had a colonic primary and 7 a rectal primary. Sites of metastases were: liver (16), lymph nodes (8), peritoneum (4), other (4). 7 patients had >1 site of metastatic disease.

There were 4 partial response (18%), 8 (36%) had stable disease, 8 (36%) progressive disease and 2 (9%) were not evaluable. The median time to disease progression was 3.1 months (95% CI 2.5–3.7). The median overall survival was 11.6 months (95% CI 6.1–17.2).

There was significant grade 3/4 toxicity: nausea/vomiting (27%), diarrhoea (18%), neutropenia (18%), thrombocytopenia (14%), renal impairment (9%), and anaemia (5%).

There were 3 deaths due to a combination of prolonged diarrhoea, neutropenic sepsis and renal failure. While the combination of Raltitrexed and Mitomycin-C is active in advanced colorectal cancer in view of the marked toxicity seen in some patients we would not advocate the use of this combination in phase III studies of combination chemotherapy for advanced colorectal cancer.

### P70 PRE-OPERATIVE CHEMORADIOTHERAPY FOR LOCALLY ADVANCED CARCINOMA OF THE RECTUM

R Cooper[1], P Finan[2], P Sagar[2], D Johnston[2], H Sue-Ling[2], P Quirke[2] and D Sebag-Montefiore[1], Leeds Cancer Centre, Cookridge Hospital[1], Leeds LS16 6QB, and Leeds General Infirmary[2], Leeds LS1 3EX, UK

**Purpose** To evaluate the effectiveness of chemoradiotherapy (CRT) prior to surgery in locally advanced carcinoma of the rectum, treated in a single centre.

**Patients and methods** Between February 1996 and August 1999 29 patients with locally advanced rectal carcinoma were treated with pre-operative CRT. Radiotherapy was delivered using a three/four field prone technique giving a dose of 45 Gy in 25 fractions over 5 weeks. Folinic acid (20 mg/m$^2$, as a bolus) and 5-Fluorouracil (350 mg/m$^2$, as a short infusion) was administered, for the first and last five days of radiotherapy.

**Results** 26/29 patients (93%) completed CRT as scheduled. Grade 3/4 toxicity was experienced by 3 patients (10%). Resection, following CRT was performed in 25/29 patients (87%) and in 23/29 (79%) the intent was curative. In 4 patients no resection was performed due to unresponsive/progressive disease. Of the 23 curative resections 6 patients (26%) had a histopathological complete response, 3 (13%) TIS-T2, 11 (48%) T3 and 3 (13%) T4. Four (17%) were node positive and 19 (83%) node negative. The circumferential resection margin was involved in 4/23. For all patients who underwent curative resection the Mandard score of pathological response was performed and correlated with outcome. Perioperative morbidity was also evaluated for this group of patients. At a median follow-up of 30 months (range 4–44) 19/23 (83%) are alive, 2/23 (8%) have local recurrence and 3/23 (13%) metastases.

**Conclusion** The CRT regimen evaluated in this study is associated with a high curative resection rate. Furthermore, toxicty was low and compliance high. At a median follow-up of 30 months outcome measures are encouraging.

### P71 CHEMORADIATION FOLLOWED BY IMMEDIATE BOOST IN SQUAMOUS CELL CARCINOMA OF THE ANUS

Dr M Osborne*, Dr R Glynne-Jones, Dr A Makris, Mount Vemon Hospital, Northwood, Middlesex HA6 2RN, UK

Chemoradiation (CRT) is now established as the primary treatment for anal carcinoma, allowing avoidance of colostomy in the majority of patients. Standard treatment in the UK (UKACCCR-Anal cancer trial) involves initial CRT followed by reassessment at 6 weeks and subsequent radiotherapy (RT) boost in responders. This practice contrasts with the recognised detrimental influence of gaps in treatment in cervical and head and neck cancer. We therefore report on 27 patients with anal carcinoma in whom the gap has been abandoned and a reduced boost delivered immediately on completion of CRT.

27 consecutive patients (11 m, 16 f), mean age 67 years (range 37–85), with anal carcinoma (26 scc, 1 adenosquamous) have been treated since 1995. Stage at diagnosis was T1 (2), T2 (10), T3 (6), T4 (9), N+ (4). RT was delivered in three phases at 1.8 Gy per fraction daily. Phase 1-parallel opposed fields including the inguinal nodes to a dose of 30.6 Gy/17 fractions,phase 2-reduced pelvic field to a total dose of 45 Gy. Phase 3 (boost)-planned volume to the primary achieving a total dose of 50.4 Gy/28 fractions (9 patients), 52.2 Gy/29 fractions (12 patients), 54 Gy/30 fractions (6 patients) according to stage. Inguinal region treated to the same dose where nodes involved. Concurrent chemotherapy (CT) was given with 5FU 750 mg/m$^2$ d1–5, 21–25 and either Mitomycin C 12 mg/m$^2$ d1 (15 patients) or Cisplatin 20 mg/m$^2$ d1–5, 21–25 (12 patients).

Mean follow up 12 months (range 2–35 months). 2 patients failed to complete RT as planned. 1 due to Grade 4 bowel toxicity limiting total dose to 43.2 Gy/24 fractions, 1 patient developed a rectovaginal fistula and completed 50.4 Gy/28 fractions/112 days. No haematological delays with CT. Acute toxicity: Skin grade 3- 26/27 (96%), Diarrhoea grade 4- 1/27 (4%), grade 3- 2/27 (7%), grade 2- 21/27 (77%), Vomiting grade 1- 3/27 (11%). Late toxicity to date: anal stenosis 1, sphincter disturbance 1. Responses: clinical CR 20 (74%), PR 6 (22%), PD 1 (4%). Relapses: 5 (19%), 2 at primary site, 2 primary and distant, 1 distant alone.

Chemoradiation in anal carcinoma and boosting without a gap is shown in this group of patients to be feasible with acceptable toxicity.

### P72 ANORECTAL IRRADIATION IN PELVIC RADIOTHERAPY: AN ASSESSMENT USING IN-VIVO DOSIMETRY

*D Hayne[1], U Johnson[2], DD'Souza[2], H Payne[3], PB Boulos[1], [1]Dept of Surgery, University College London W1P 7LD, [2]Dept Medical Physics and [3]Dept Of Oncology, Middlesex Hospital W1N HAA, UK

**Objectives** To compare in vivo techniques of measuring radiation doses received by the anorectum during pelvic radiotherapy with the dose predicted by a GE *TARGET*™ treatment planning system.

**Background** Radiation damage to the anorectal region following pelvic radiotherapy is a cause of severe morbidity in some patients. Functional and structural changes in the anorectum, in relation to the site and dose of pelvic radiation received, have not been fully investigated. For this purpose an accurate technique for measurement of the dose has been developed.

**Methods and study design** Five patients with cancers of the prostate, cervix and uterus were CT planned using the *TARGET*™ system. A Scanditronix rectal probe containing 5 n-type photon-detecting diodes was placed in the anorectum during the planning CT. The probe position was standardised with the 5 diodes at 2 cm intervals from the anal verge. The probe diodes were calibrated for 10 MV photons. Doses were measured for each diode on 5 consecutive fractions in the first patient. The anal canal was included in the target volume because of local invasion. The probe was then used to measure radiation doses during 2 fractions in the remaining 4 patients. Thermo-luminescent devices (TLDs) were also attached over the sites of the diodes to further verify measured doses. *TARGET*™, diode measured and TLD measured doses were compared.

**Results** Diode measured doses in the first patient, were reproducible (SD<1% of mean. Range 0.2–0.9%). Diode measured doses were all within 2.5% of *TARGET*™ predicted doses. TLD measured doses on the same 5 occasions were less reproducible than diode measured doses (SD<5% of mean. Range 2.4–4.7%) and were all within 5% of *TARGET*™ predicted doses.

Diode read consistently lower than predicted doses, on average by 2%. TLDs read consistently higher than predicted doses on average by 2.8%.

In the other 4 patients diodes and TLDs within the target volume were within 7% of predicted doses. Outside the target volume diodes and TLDs agreed but were less comparable with predicted doses. Greatest variability was seen at the edges of the target volume where dose varies widely across small distances. (Range –68% to +54% of predicted dose).

**Conclusions** The results in the first patient suggest that *TARGET*™ planned doses are accurate. The reproducibility of results can be explained by all of the diodes being in the target volume. Anal canal stenosis in this patient may also have minimized probe movement.

Changes in the position of the probe at CT planning and between readings probably accounts for measured differences in the other patients. Measurement on more than 2 fractions might improve results.

### P73 PERIPHERAL RIM ENHANCEMENT (PRE) OF HEPATIC COLORECTAL CANCER (CRC) METASTASES DURING ENHANCED COMPUTED, TOMOGRAPHY (CT): A NEW PROGNOSTIC INDICATOR

D Farrugia*[1], D Elias[2], I Vlahos[2], C Mulatero[1], J Kellaway[1], P Wilson[1], A Martin[2], ML Slevin[1], RH Reznek[2], Departments of Medical Oncology and Radiology, St. Bartholomew's Hospital, London, ECIA 7BE, UK

PRE of hepatic CRC metastases during contrast enhanced imaging is a recognised phenomenon possibly relating to new vessel formation (angiogenesis) at the growing edge of tumour masses. We used dynamic CT to evaluate PRE in hepatic CRC metastases and to correlate this with prognosis. Fifty one patients (pts) with metastatic disease underwent baseline CT scanning before (20 pts), or after commencing (30 pts) palliative chemotherapy (timing unspecified in 1 pt). Single level dynamic CT scans were performed at 3 second intervals after intravenous iohexol (300 mg/ml, 6 mls/sec) from early arterial to late portal phase. Parameters measured included morphology of PRE in terms of definition, thickness and intensity of rim enhancement, total hepatic metastatic volume (TMV), % hepatic replacement by tumour (PHR) and hepatic perfusion index (HPI): a ratio of hepatic arterial to total hepatic blood flow in normal liver. Median age of pts was 66 years, 72% had performance status (PS) 0–1, and 46 received or were receiving chemotherapy. Median survival from baseline PRE evaluation was 350 days (range: 13–1210) and 22 pts were alive at analysis. It was possible to consistently group PRE into 4 categories: absent (group 0, n = 12), illdefined blush (group 1, n = 18), well-defined thin rim (group 2, n = 11) and well-defined thick rim (group 3, n = 10). Univariate analysis using Kaplan-Meier test identified high PRE score (≥2), TMV > median, low PS and high primary tumour grade as adverse prognostic factors. Median survival was 240 days (95% CI: 177–303) in PRE groups 0–1 (n=30) compared to 146 days (95% CI: 54–238, p=0.035) in groups 2–3 (n = 21). Age, gender, Dukes stage, PHR, HPI and plasma CEA levels are not significant at this stage. Inclusion of serial scans over time from 19 pts (total: 76 scans from 51 pts) increased adverse prognostic significance of higher PRE score (p=0.0035). Thus the morphology of PRE on CT has potential prognostic value. A multivariate analysis is planned with continued recruitment. We are evaluating the impact of chemotherapy on PRE and possible correlation between PRE and other parameters of tumour angiogenesis.

### P74 NO ASSOCIATION BETWEEN THE GSTMI, GSTTI, AND GSTPI GENE POLYMORPHISMS, SQUAMOUS CELL CANCER OF THE HEAD AND NECK AND SECOND PRIMARY TUMOURS

S Jefferies*[1], Z Kote-Jarai[1], R Houlston[1], M-J Frazer-Williams[1], R A'Hern[2], MPT Collaborators and R Eeles[1]

Individuals who have had an index squamous cell cancer of the head and neck (SCCHN) have a 10–20% chance of developing a second primary tumour (MPT). Traditionally any familial clustering of head and neck cancer has been attributed to sharing of the same environmental risk factors, such as tobacco and alcohol. However, there is some preliminary evidence that there may be a genetic component to SCCHN. There is an increased risk of cancer in first-degree relatives of individuals with SCCHN and this risk rises markedly when the affected individual has had two primary tumours. Carcinogens are detoxified by the phase II enzymes glutathione S-transferases (GST) which may have an important role in the development of tumours. These enzymes, due to the presence of polymorphisms causing loss of function, may provide some explanation for observed individual variation in cancer susceptibility. GSTM1 and GSTT1 null genotypes have been associated with increased risk of developing SCCHN. The GSTP1 gene has a polymorphism at nucleotide 313 resulting in a Ile to Val substitution which consequently lowers the enzymatic activity of the protein. This has also been suggested to increase cancer susceptibility.

We investigated the association between genetic polymorphisms in GST genes and patients with index squamous cell cancer of the head and neck who developed second primary tumours (SPT) in a case-control study. GSTM1, GSTT1, and GSTP1 genotypes were determined in 52 cases of MPT and for 280 controls by PCR and RFLP. The associations between specific genotypes and the development of MPT were examined by use of the Chi-squared test.

The results for this study did not show any increased risk for the development of MPT with the presence of null genotypes for GSTM1, GSTT1 or heterozygous or homozygous polymorphism of the GSTP1 gene. We therefore conclude that polymorphisms in the GSTM1, GSTT1 and GSTP1 genes do not play a major role in cancer susceptibility in this group. Tobacco and alcohol exposure and metabolic enzyme genotype may affect risk for development of MPT and we are currently assessing this for the MPT cases versus matched controls.

MPT Collaborators: J Henk[2], M Gore[2], P Rhys-Evans[2], D Archer[2], K Bishop[2], E Solomon[3], S Hodgson[3], M McGurk[3], J Hibbert[3], MO'Connell[3], M Sauders[4], M Partridge[3], E Chevretton[3], F Calman[3], K Shotton[5], A Brown[6], S Whittaker[7], D Goldgar[8], W Foulkes[9]. [1]Cancer Genetics, Institute of Cancer Research, 15 Cotswold Rd, Sutton, Surrey. [2]The Royal Marsden Hospital Trust, Downs Rd, Sutton, Surrey, SM2 5PT. [3]Guys and St Thomas' and Kings Hospital Trusts. [4]Mount Vemon Hospital Trust, Rickmansworth Road, Northwood, Middlesex, HA6 2RN. [5]Mid-Kent Oncology Centre, Hermitage Lane, Maidstone, Kent, ME16 9QQ. [6]Queen Victoria Hospital, East Grinstead. [7]Royal Surrey County Hospital, Egerton Road, Guildford, GU2 5XX. [8]IARC, Lyon, France. [9]McGill University, Montreal, Canada.

### P75 THE USE OF IRIDIUM 192 IMPLANTS FOR T1/T2 NO SQUAMOUS CELL CARCINOMA OF THE TONGUE AND FLOOR OF MOUTH – CONTROL AND COMPLICATIONS

S Sothi*, J Townley, J Glaholm and A Goodman, The Cancer Centre, Queen Elizabeth Hospital, Birmingham, UK

We present the results of patients with T1 and T2 N0 squamous cell carcinoma of the tongue and floor of mouth treated with iridium 192 implantation at our centre between 1994 and 1998. Thirty four patients were treated by two consultants. Twenty six had T1 tumours, 8 had T2. Median age was 61 years (range 35 to 84 years). Median tumour size was 2.0 cm (range microscopic to 3.5 cm). All patients had histologically proven squamous cell carcinoma. Seven had muscle invasion. Seven patients had laser excision prior to brachytherapy, five with incomplete excision and 2 with close margins. The other 27 were biopsied only. Twenty seven patients were treated with brachytherapy alone and all received 60Gy with a median dose rate of 0.52 Gy/hr (range 0.34 to 0.87 Gy/hr). Seven patients were treated with a combination of brachytherapy (30 Gy) and external beam radiotherapy (41.25 Gy in 15 fractions) to primary site and first station nodes. Six had brachytherapy followed by external beam treatment starting 1–6 weeks after implantation (median 2 weeks). In one patient brachytherapy was performed two weeks after completion of external beam radiotherapy.

Median follow up was 35 months (range 3 to 62 months). Two out of 34 (6%) patients have recurred locally, both had simultaneous regional node recurrence. Both were treated with brachytherapy alone. Six patients (18%) developed regional node recurrence, five were treated with brachytherapy alone. Two patients with regional recurrence have been successfully salvaged with surgery. Five patients have died, three with recurrent disease. Actuarial two year survival was 85% and two year cause specific survival was 91%. Sixteen patients developed soft tissue necrosis. Fifteen had mild to moderate necrosis healing with conservative treatment. One patient had persistent symptomatic tongue ulceration which healed after hyperbaric oxygen. None required surgery. Four patients had osteoradionecrosis. Three had been treated with brachytherapy alone. One healed with conservative treatment, two after hyperbaric oxygen and the other required surgery and hyperbaric oxygen. All four remain alive and well.

In conclusion, interstitial therapy with iridium 192 is effective treatment for small T1 and T2 N0 squamous carcinomas of the tongue and floor of mouth. Supraregional specialisation is required in view of small patient numbers.

### P76 PLASMA TRANSFORMING GROWTH FACTOR BETA 1 (TGFβ1) LEVELS AND ACUTE RADIATION MORBIDITY IN PATIENTS RECEIVING RADICAL RADIOTHERAPY TO THE HEAD AND NECK REGION

J Dickson, [1]CML West, [2]N Slevin, [2]AJ Sykes, [1]CRC Experimental Radiation Oncology Group, [2]Christie Hospital NHS Trust, Wilmslow Road, Manchester M20 4BX, UK

**Introduction** There is increasing evidence that plasma TGFβ1 levels can be used as a predictor of both prognosis and acute radiation morbidity in a number of malignancies including breast cancer NSCLC. This prospective study was designed to investigate the relationship between plasma TGFβ1 levels and acute radiation morbidity in a group of patients receiving radical radiotherapy for carcinomas of the head and neck region.

**Materials and methods** Plasma was obtained prior to the start of and in the final week of a course of radical radiotherapy. TGFβ1 levels were measured by sandwich ELISA, using a commercially available kit. Data on the severity of the acute reaction were available at the end of treatment and at six week follow-up. Plasma TGFβ1 levels were correlated with acute reaction score (as measured by the LENT SOMA scale) using non-parametric tests.

**Results** Plasma TGFβ1 levels were available on 75 patients prior to the start of treatment and in 68 patients in the final week of treatment. 19 patients had elevated pre-treatment values and 13 patients had elevated values in the final week of treatment (normal 2.59 ng/ml). There was a weak positive correlation between end of treatment TGFβ1 levels and acute reaction score at first follow up (p = 0.057). Patients whose TGFβ1 levels had normalised at the end of treatment had a significantly lower median reaction score those whose TGFβ1 level remained elevated (6 versus 31.5, p = 0.003).

**Conclusion** Plasma TGFβ1 levels show promise in predicting those patients who develop severe acute radiation morbidity following radical radiotherapy for head and neck malignancies.

This work was supported by the Christie Hospital Endowment Fund and the Cancer Research Campaign.

### P77 A PILOT STUDY OF POSTOPERATIVE CHART AND CHARTWEL IN HEAD AND NECK CANCER

N Shah*, M Saunders, S Dische, Marie Curie Research Wing, Mount Vernon Hospital, Northwood, HA6 2RN UK

**Purpose** A phase 2 feasibility study of CHART (Continuous hyperfractionated accelerated radiotherapy) and CHARTWEL (CHART weekend less) in the post operative treatment of patients with squamous cell carcinoma of the Head and Neck.

**Methods** 24 patients (17M:7F) with a median age of 64 years (range 34–80) were treated with postoperative radiotherapy from 1991 to 1999. All patients presented with primary squamous cell carcinoma which at surgery had shown adverse pathological factors for recurrence. Patients were treated using a CHART (n=11) or CHARTWEL (n=13) schedule from 49.5 Gy to 54 Gy.

**Results** Adjuvant radiotherapy was given to 17 patients following radical surgery as initial management. In 7 other patients, recurrence after surgery had occurred and was treated by further surgery and postoperative radiotherapy (1 primary tumour recurrence, 4 nodal recurrences and 2 recurrent primary and nodal disease). Primary tumour sites included oral cavity (9), oropharynx (8), pinna (3), larynx (2), columnella (1) and unknown primary (1).

Treatment was commenced from a median time of 6.9 weeks (range 4.4–16.6) after radical surgery. All patients completed treatment, with one patient's treatment extended by one day due to gastrostomy tube complications.

A confluent radiation mucositis occurred in 20/23 evaluable patients, and mucositis settled in four to ten weeks after commencing radiotherapy. Moderate to severe dysphagia occurred in 15/23 patients. Narcotic analgesics were given in 14/23 patients during the period of mucositis. Skin erythema was noted in all patients with dry and moist desquamation appearing from 2 weeks in the region of the high dose field. Resolution of the desquamation occurred in all patients from 4 weeks. Mild subcutaneous oedema was observed in 11 patients from 12 weeks after treatment. All mucosal reactions have healed with no consequential late necrosis. No cases of transient or permanent radiation myelitis have been observed.

Over a median follow up period of 16 months, local control is maintained in 17 patients (70%). 7 patients have relapsed and died of disease. A mean survival of 49 months (range 1–75 months) is observed.

**Conclusion** This pilot study demonstrates acceptable morbidity for CHART/CHARTWEL in the postoperative setting. A prospective multicentre randomised trial using an accelerated schedule of radiotherapy versus conventional fractionation for the radical postoperative treatment of primary Head and Neck cancer is currently in preparation.

### P78 THE TIME SPAN BETWEEN SYMPTOM ONSET AND THE START OF RADIOTHERAPY IN THE TREATMENT OF HEAD AND NECK TUMOURS – A RETROSPECTIVE REVIEW

E Pettet*, H Phillips, S Jackson & MI Saunders, Mount Vernon Hospital, Rickmansworth Road, Northwood HA6 2RN, UK

**Introduction** The importance of the overall duration of a course of radiotherapy for head and neck cancer has been emphasised in the past 10 years. Very little importance has however been given to the time that the patient spends between onset of symptoms and commencement of radiotherapy treatment. This research has set out to investigate the time span between symptom onset and the start of radiotherapy in patients with head and neck cancer.

**Patients and Methods** The time span between the onset of symptoms and the commencement of radiotherapy in patients with head and neck cancer was assessed over a one year period. During this time patients were interviewed and their hospital records reviewed. The duration of delays experienced at the various stages of patient management was noted. The time spans identified were:

[1] Patient delays – the time taken for the patient to seek professional advice usually from a general practitioner or dentist.

[2] Specialist delays, that is the time taken for the patient to be referred to the hospital specialist usually an ENT or orofacial maxillary surgeon for the diagnosis to be made and a management decision taken with the clinical oncologist.

[3] Planning delays – the time taken from seeing an oncologist to the first day of radiotherapy treatment.

Patients were then subdivided into 6 sites of malignancy: nasopharynx, larynx, hypopharynx, oropharynx, oral cavity and "other". The data analysed as to whether the patients underwent surgery and post-operative radiotherapy or radiotherapy only.

**Results** The findings of this study show that on an average a head and neck patient takes 94 days (13.5 weeks) to seek professional advice from the onset of symptoms and then spends a further 123 days (17.5 weeks) in the system before receiving radiotherapy. On an average 44 days elapsed between first seeing the clinician and referral to the hospital, 20 days were taken to achieve a diagnosis and a further 10 days to make the management decision towards surgery, radiotherapy or a combined modality approach. Once referred to the oncologist 49 days elapsed between that consultation and commencement of radiotherapy. Analysis at the time delays for individual sites will be given in detail and their relevance as to outcome.

### P79 INTERSTITIAL PHOTODYNAMIC THERAPY IN HEAD AND NECK CANCER

Hopper C*, Suhr M, Jones L, Nakanishi H, Jaeger R, Brookes J, National Medical Laser Centre and University College London Hospitals NHS Trust

**Background** For many years, the mainstay of treatment for oral squamous cell carcinoma has been surgery and radiotherapy. Photodynamic therapy (PDT) is a local treatment that can be applied to such tumours and has the advantage of being a repeatable, tissue-sparing treatment that leaves no lasting damage. The depth of photodynamic effect is limited by the ability of the activating light to penetrate through tissue. In practice, this has resulted in PDT being of value only in the management of thin tumours.

**Aim** The aim of this study was to see if light could be delivered into tumour tissue to produce a photodynamic effect.

**Procedure** All patients received 0.15 mg/kg Foscan and 4 days later had their tumours treated with laser light of 652 nm delivered intratumorally from a point source laser fibre at 10–20 Joules/cm$^2$. 38 treatments on 26 patients with advanced head and neck cancer have been carried out, initially by direct fibre placement, latterly under ultrasound, CT and MRI control.

**Major findings** The treatment was well tolerated in all patients and produced consistent tumour necrosis. No specific neurotoxicity was seen even when tumour was treated around nerves such as the hypoglossal, vagus and brachial plexus. The most important limiting factor was the proximity of the tumour to major blood vessels. In one patient, there was a rarely encountered event of tumour erosion through the carotid artery, and a blow-out was seen 8 days following treatment.

**Significance** No tumour type was immune to the PDT effect and normal tissue recovered well following treatment. This is a repeatable treatment with no cumulative toxicity and is an option when other treatments have failed

**Conclusion** Interstitial PDT offers a minimally invasive, local treatment for malignant disease which is potentially applicable to any site in the body.

### P80 COST UTILITY OF TAXOTERE VS VINORELBINE OR TAXOL IN ADVANCED BREAST CANCER

Brown R[1], Hutton J[1], [1]MedTap International, London, UK

The objective of this study was to capture the costs and quality of life (QOL) related outcomes for advanced breast cancer (ABC) patients management with Taxotere (TAX) in comparison to vinorelbine (VIN) and taxol (TOL). An updated version of the Hutton et al. (Pharmacoeconomics 1996) model was used to simulate the experiences (associated costs and outcomes) of patients undergoing treatment for ABC, from the onset of chemotherapy to death. Published clinical trials were used to establish response rate, time to progression, median survival, rate of grade four febrile neutropenia, and toxicity rate related to chemotherapeutic agent. Costs were taken from published sources and reflect the UK National Health Service and were discounted at 6%. Average patient costs were found to be £4268 for VIN, £7817 for TAX and £7645 for TOL. The estimated Quality Adjusted Life Year values were 0.48 for VIN, 0.73 for TAX and 0.65 for TOL. This means that VIN produces an additional 175 days of good quality life, TAX produces an additional 266 days and 237 days for TOL. The incremental cost per quality adjusted life year (QALY) for TAX were £14,500 compared with VIN and £1,990 compared with TOL. Various sensitivity analyses were undertaken and did not change the findings greatly. The cost-effectiveness ratios are within the range of generally acceptable technologies. Patients managed with TAX have improved QOL in comparison to these alternative chemotherapies and a longer median survival.

### P81 COSTS OF FAILURE OF 1ST LINE CHEMOTHERAPY FOR ADVANCED BREAST CANCER IN THE UK

Tran G[1] Burrell AD[2]*, Jackson DL[2]*, [1]Lewin Group, Bracknell UK, [2]Aventis Pharma, West Malling, Kent, UK

**Objective** The objective of this study was to summarise medical resource utilisation and estimate the overall costs of treatment failure following first-line chemotherapy in patients with advanced breast cancer in the UK.

**Design** The study was designed to abstract data from medical notes of patients who had withdrawn from 1st line chemotherapy for advanced breast cancer. The medical notes of these patients were retrieved and clinical outcomes, as well as medical resource utilisation data were abstracted from date of study treatment failure (withdrawal) to 200 days following withdrawal, or up to the date of last contact or death. All data was abstracted by trained nurses onto specifically designed case report forms. The main analytic framework was an assessment of the overall costs of treatment failure associated with the 23 patients withdrawn due to disease progression, adverse events or other reasons. The cost evaluation only included direct costs as the study perspective was the UK NHS.

**Study end points** Medical resource utilisation measures were anti-tumour therapy and setting in which these therapies were administered, hospitalisation days (days spent in each type of ward), consultations and location in which patients attended these consultations, tests/procedures and setting in which these were performed.

**Results**

| Reason for Withdrawal | Number of Patients | Median cost per Patient (£) |
|---|---|---|
| Disease progression | 9 | 5,590.8 |
| Adverse Event | 8 | 2,959.22 |
| Other | 6 | 1,726.12 |
| *All patients* | 23 | 3,728.44 |

The overall cost of treatment failure was found to be high, with a significant proportion of this cost (55.8%) attributable to hospitalisation. Disease progression implied the greatest associated costs. However, the small sample size of this study does not allow a definitive conclusion.

**Conclusions** Treatment failure due to progression appears to incur additional costs. Cost offsets may be possible from therapies which prolong time to progression or are associated with less adverse events which require treatment discontinuation.

### P82 A SYSTEMATIC REVIEW OF DOCETAXEL, PACLITAXEL AND VINORELBINE IN THE TREATMENT OF ADVANCED BREAST CANCER IN THE UK

Lorna Anderson[1], Jane Cox[1], [1]Anderson Cox Consulting, 48 Hazeldene Road, Chiswick, London W4 3JB, UK

A systematic literature review was undertaken to help determine which is the most effective therapy for treatment of advanced breast cancer within UK licensed treatment regimens. As there is a lack of head to head Phase III randomized controlled trials comparing docetaxel, paclitaxel, and vinorelbine. Cochrane Collaboration methodology was followed to design a literature search. A cut-off point of 18 August 1999 was decided upon and the following databases were searched: EMBASE, EMBASE Alert, HealthStar, CancerLit, and MEDLINE. Five Phase III trials (three for docetaxel, one for paclitaxel, one for vinorelbine) were found within the pre-specified inclusion criteria. Study populations were similar, and dosing schedules were in accordance with their UK licensed indications. Patient-weighted averages for efficacy and safety from the docetaxel studies were calculated and compared with the paclitaxel and vinorelbine studies. When compared as single agents, docetaxel appears to produce a greater overall response rate than paclitaxel or vinorelbine in this patient population. Docetaxel is also associated with greater median time to progression and median survival than vinorelbine, although complete response rates appear similar for the two agents. Grade III/IV safety outcomes, where comparisons allowed, were largely similar, with docetaxel associated with a higher incidence of neutropenia, asthenia, and diarrhoea, but a lower incidence of anemia compared to vinorelbine. Comparable data for paclitaxel were not available from the publication. A quantitative meta-analysis of data from these studies is ongoing. Whilst indirect comparisons from disparate studies should be interpreted with caution, this represents the best currently available evidence, and suggests that docetaxel may be more effective in terms of overall response rate, time to progression, and survival in this patient population.

### P83 AN AUDIT OF DOCETAXEL AS A SECOND LINE AGENT IN TREATMENT OF METASTATIC BREAST CANCER IN SOUTH EAST WALES

S Mukherjee*[1], IJ Robbie[2], D Allen,[1] CC Gaffney, [1]DWO Tilsley[1], PJ Barrett-Lee[1], [1]Velindre Hospital, Cardiff CF4 7XL, [2]Bro Taf Health Authority

**Background** Docetaxel, a semi-synthetic agent of the taxoid family has shown great potential in the management of advanced breast cancer, and is now widely used as a second line agent in the treatment of metastatic breast cancer. To the best of our knowledge, this is the first audit of the use of this drug in a hospital setting.

Funding for the use of docetaxel as a second line agent for metastatic or locally advanced breast cancer was obtained from Bro Taf Health Authority, SE Wales, in September 1998, which agreed to fund 24 patients per year from the Bro Taf population.

Patients aged less than 65 years with metastatic or locally advanced breast cancer, who had received prior anthracyclines, and had an anticipated survival greater than 3 months, were considered eligible.

We have audited the use of this drug at the end of one year of its use.

**Results** 15 patients were treated (Mean age 47.5 years, range 31–61 years), all of whom had prior anthracyclines and 13 of whom (87%) had more than one site of metastasis. 9 of the patients (60%) completed 6 cycles, and 8 (53%) of them started on 100 mg/m2.8 of the 15 patients (53%) showed response to the drug with a median survival of 204 days. 6 of the patients (40%) required admission due to neutropenic sepsis, one of whom was admitted on 5 occasions. 1 patient died of neutropenic sepsis. Interestingly, 5 of the 6 patients (83%) with neutropenic sepsis responded to docetaxel chemotherapy.

**Conclusion** The response rate and the duration of survival achieved using docetaxel in our hospital are at least comparable to that reported in literature[1]. However there has been a higher incidence of patients admitted with neutropenic sepsis. Neutropenic sepsis may be a more significant problem than encountered in the trials and may be more common in patients with multiple sites of involvement and liver disease. It might also be a predicator of response. Further studies are necessary to elucidate this aspect of docetaxel chemotherapy.

1 O'Brian MER, Leonard RC, Docetaxel in the community setting: An analysis of 377 breast cancer patients treated with docetaxel (Taxotere) in the UK
Barrett-Lee PJ, Eggietor SPH, Bizzari JP, on behalf of UK Study Group (1999) *Annals of Oncology* **10:** 205.

### P84 LARGE VARIATIONS IN UK MANAGEMENT OF INTRADUCTAL CARCINOMA OF THE BREAST

D Landau*[1], E Hall[2], G Ross[12], The Royal Marsden Hospital and Institute of Cancer Research London SW3 6JJ, UK

**Background** Ductal carcinoma in situ (DCIS) accounts for 20% of screen-detected breast cancers. There are however a lack of mature clinical trial data and consequently many unresolved issues in the area of clinical management.

**Aim** We performed a national survey of current UK practice in the management of DCIS in order to investigate variations in such practice and to inform the design of any future national prospective randomised trials in DCIS.

**Methods** A questionnaire was sent on 17/11/1999 to 271 consultant surgeons to whom the UK breast screening centres refer breast cancer patients and to 203 consultant clinical oncologists treating breast cancer patients at UK radiotherapy centres. It was re-sent to non-responders on 18/1/2000. The results of questions related to workload and current clinical practice are presented here.

**Results** 182 (67%) surgeons and 131 (65%) clinical oncologists returned completed questionnaires. The combined results are as follows:

| Workload: new patients per month (n=308) | | Indications for Radiotherapy (n=298) | | Indications for Tamoxifen (n=280) | |
|---|---|---|---|---|---|
| <1 | 26% | None | 14% | None | 21% |
| 1–2 | 44% | All DCIS | 14% | All DCIS | 26% |
| 3–4 | 19% | All HG/comedo | 45% | All postmenopausal | 10% |
| 5 or more | 10% | Specific size | 29% | All ER+ | 27% |
| Indications for Mastectomy (n=302) | | Close margin | 34% | All HG/comedo | 11% |
| | | Family history | 5% | Widespread or multifocal DCIS | 16% |
| >1 quadrant | 78% | Microinvasion | 48% | Microinvasion | 31% |
| Specific size | 51% | Combin.factors | 45% | Bilateral | 15% |
| Family history | 12% | Radiotherapy: Use of boost* (n=117) | | Family history | 15% |
| Combin. factors | 44% | | | Combination of factors | 18% |
| Axillary Surgery (n=298) | | Not used | 76% | | |
| No patients | 59% | Routine | 9% | | |
| Selected patients | 41% | Close margins only | 15% | | |
| Re-excision if margins involved (n=296) | | | | | |
| Yes | 97% | | | | |

*surgeons not asked this question
*n = number of respondents to each question*

**Conclusions** We believe this to be a representative overview of current UK practice. It reveals large variations in the management of a common form of breast cancer regarding indications for mastectomy, radiotherapy and tamoxifen and confirms the need for evidence-based guidelines.

### P85 SPECIALIST PALLIATIVE CARE IN CANCER OUTPATIENTS: WHAT ROLE?
V Lidstone*, C Sinnott, T Beynon, M Richards, Dept of Palliative Medicine, St. Thomas' Hospital, London SE1 7EH, UK

**Aims** To determine the views of Oncologists regarding functions and acceptability of Specialist Palliative Care (SPC) in Oncology out-patients.

To determine the views of SPC staff regarding actual and potential role in Oncology clinics.

To define the most effective and acceptable model of input to aid future service planning.

**Design** Oncologists and SPC doctors and nurses regularly involved in Oncology Clinics at Guys and St. Thomas' Hospitals were asked to agree to semi-structured interviews. The interview included general questions about current running of the Clinics and specific questions about current and appropriate future provision of SPC. Interviews were recorded on audio-cassette for internal validation.

**Subjects** 12 Oncology Consultants. 10 Oncology Specialist Registrars. 4 SPC staff.

**Results** Interviews revealed a high level of interest in SPC involvement in the clinics. Doctors found SPC input particularly beneficial when it provided a link with the community service and an opportunity for information exchange. Symptom control was highlighted by oncologists as an important function of the team particularly when symptoms were multiple, difficult to control or related to poly-pharmacy. Counselling/psychological support was also a valued role. These roles were recognised by the SPC staff who also highlighted the building of relationships with oncology staff, and introduction of patients to the concept of SPC as important functions of their presence in the clinics. All but one of the oncologists interviewed felt that further placement of SPC in their clinics would be beneficial, although SPC staff currently placed in out-patients feel they are under-used. Most Oncologists would prefer the support of a SPC Nurse rather than a SPC Doctor, and SPC staff themselves agree nurses are probably more appropriate. Half favoured a parallel clinic model, half joint clinics.

**Conclusions** SPC services in out-patients are highly valued and widely acceptable to Oncologists, many of whom view it as an essential part of patient management and would like expansion of this service in the future. However, despite this high level of enthusiasm SPC staff who are currently placed in oncology clinics felt that Oncologists were under-referring and this is likely to be related to the limited perception they had of the role of SPC. Expansion of the service in out-patients will require education of both parties in order to provide the most efficient model of care for patients.

### P86 PROVISION OF SPECIALIST PALLIATIVE CARE IN CANCER OUTPATIENT CLINICS: A NEEDS ASSESSMENT
V Lidstone*, C Sinnott, T Beynon, M Richards, Dept of Palliative Medicine, St. Thomas' Hospital, London SE1 7EH, UK

**Aim** To define and prioritise the need for Specialist Palliative Care (SPC) in Cancer Outpatient Clinics.

**Design** A validated 29-item Symptoms/concerns checklist.

**Setting** Guys and St. Thomas' Hospitals out-patients departments.

**Subjects** 480 patients with a histologically proven diagnosis of cancer (n=60 for each of: lung, breast, gastrointestinal (G.I), gynaecological, urological, genitourinary (G.U), brain and lymphoma).

**Results** This study defined the prevalence and severity of 29 common symptoms and concerns in an oncology out-patient population. Recruitment was trouble-free (97%), and patients answered on average 27/29 of the questions, reporting around 10 items as current problems. The highest overall level of need, as defined by the highest number and most severe symptoms/concerns, was reported by the patients with lung tumours, followed by those with brain tumours. The lowest levels of need were reported by those with lymphoma and G.U. tumours. Those with advanced disease reported a higher level of need than those with non-advanced disease; those in remission had the lowest level of need. Those on current radio/chemotherapy had higher levels of need than those not although the differences were not significant. Those aged 30–39 and 40–49 yrs had the highest level of need, and the need declined after the age of 50 yrs. Of the six symptoms known to be improved by SPC intervention the group of patients with lung tumours reported the highest frequencies of mild/moderate/severe symptoms for five of these, and highest frequencies of moderate/severe symptoms for three. The group of patients with G.I. tumours reported the second highest levels for three of these symptoms.

**Conclusions** The checklist represents a feasible and acceptable tool for audit of symptoms/concerns in cancer out-patients. A high proportion of these patients have multiple problems. Some may be alleviated by treatment/care. More than half of the sample studied reported one or more moderate/severe problem that may benefit from SPC intervention. With regards to the tumour groups studied there is no single agreed measure of need in this context. However, using a variety of approaches patients with lung cancer appear to have the highest level of need followed by those with brain tumours, G.I. tumours, breast tumours and head and neck cancer. Patients with lymphoma and gynaecological or G.U. primaries and appear to have lowest levels of need, though individual patients may have high levels of need. Advanced patients are likely to benefit more from intervention but this is relative and non-advanced and patients in remission should not be excluded.

### P87 CAN THE ADMISSION OF MEDICAL ONCOLOGY PATIENTS TO INTENSIVE CARE BE JUSTIFIED?
G Hall*[1], E Harrer[1], T Perren[1], P Selby[1], [1]ICRF Cancer Medicine Research Unit, St James's University Hospital, Leeds, UK

The use of intensive care beds in the UK has recently been the focus of considerable interest. The admission of any patient receiving cancer treatment to the intensive care unit (ICU) frequently causes considerable debate regarding whether such an admission can be justified in view of the underlying diagnosis. We have therefore reviewed the medical oncology admissions to the ICU at our centre.

Medical oncology admissions to ICU between January 1993 and July 1999 were identified by comparing the electronic databases of both units. Admission to our ICU reflects a need for ventilatory support whereas other acutely ill patients are managed on the general oncology ward. Case notes were reviewed to identify the reason for admission to exclude patients admitted to ICU following primary surgical resection. Patient age, diagnosis, duration of admission, outcome (death or discharge from ICU) and survival from date of admission were subsequently recorded. A comparison of these data with all patients recorded within both the oncology and ICU databases were made.

41 patients were identified with a median age of 48.2 years (range – 18 to 74 years). The three most common underlying diagnoses were lymphoma (12 patients), breast (8 patients) and germ cell tumours (5 patients). The most frequent reason for admission was respiratory support associated with neutropenic sepsis. A total of 316 'bed-days' were used by these admissions with an average of 8 days per patient. Eighteen patients died on ICU, a further four within 7 days of discharge. Five patients died more than one month after discharge from ICU and ten patients are still alive (range – 6 to 77 months). 34.1% of patients therefore survived more than one month following discharge from ICU. This compared favourably to the survival of other patients admitted to ICU.

This review confirmed our impression that cancer patients admitted to ICU do not have a uniformly poor prognosis. It reinforces the need to assess each seriously ill patient on an individual basis to ensure that the stigmata of their diagnosis does not restrict their access to life-saving facilities.

### P88 LONG TERM OUTCOME OF PATIENTS WITH LOCALLY ADVANCED THYROID CARCINOMA
J Powles*, L Vini, RA 'Hern, C Harmer, P Rhys-Evans, Head, Neck and Thyroid Unit, Royal Marsden Hospital, Fulham Road, London SW3 6JJ, UK

**Aim** This is a retrospective study to assess the long term outcome of patients with locally advanced (pT4) differentiated thyroid carcinoma.

**Patients** Of 1404 patients with differentiated thyroid carcinoma treated at the Royal Marsden Hospital over the last 60 years, we identified 255 who had locally advanced tumours at diagnosis (pT4). Of these 94 were male and 161 female (M:F= 1:1.71), with a mean age at diagnosis of 56 (range: 7–85 years). Most patients (85%) presented with a thyroid nodule or a palpable neck node. Mean follow up was 21 years (range: 1–60 years).

**Results** There were 193 papillary, 52 follicular and 10 Hurthle cell carcinomas; 48% of all tumours were well differentiated and 25% poorly differentiated. 148 patients were recorded as having involved lymph nodes, 76 were ipsilateral (N1a), and distant metastases were found in 46 cases (most commonly in lungs and bones). Initial management included total thyroidectomy in 138 cases, lobectomy/hemithyroidectomy in 59 and biopsy/enucleation in 59. A modified radical neck dissection was performed in 50 patients while simple nodal excision occurred in 54. Radioactive iodine was given to 238 patients, with 31 receiving an ablative dose and the rest receiving more than 1 dose (cumulative activity: 8.5–24GBq). External beam radiotherapy was given to 72 patients. Local recurrence (thyroid bed, neck nodes or mediastinum) occurred in 93 patients and was managed by further surgery, radioiodine or external beam radiotherapy 46 patients (18.04%) relapsed at distant sites. Overall cause specific survival at 10, 20 and 30 years was 60%, 45% and 45% respectively. Age at diagnosis and extent of surgery were found to be independent prognostic factors for local recurrence as well as for survival, while external beam radiotherapy was a significant prognostic factor for local control.

**Conclusions** Locally advanced differentiated thyroid carcinoma is associated with aggressive behaviour and should be managed with total thyroidectomy and selective neck dissection (if involved) followed by radioactive iodine. Radiotherapy should be also considered, especially in older patients, to reduce the risk of local recurrence.

### P89 AN AUDIT OF GERM CELL TUMOURS REFERRALS TO A CANCER CENTRE

VR Bulusu*[1], J Robson[1], MV Williams[1], MA Ward[2], [1]Oncology Centre, and [2]Clinical Audit Department, Addenbrooke's Hospital, Cambridge CB2 2QQ, UK

**Aims** 1. To audit the time taken for a patient with suspected germ cell tumour (GCT) of the testis from presentation to the GP to the treatment. 2. To identify the bottlenecks if any, in the referral pathway. 3. To compare our data with the national guidelines for GCT referrals.

**Material and Methods** 41 case records of patients with GCT of testis referred to the Oncology centre, during the period 1998–1999, have been audited. The following information was obtained from the GPs, surgeons and the oncologist. 1. Date of GP referral 2. Date of surgical consult 3. Date of orchidectomy 4. Date of referral to oncologist 5. Date seen by oncologist 6. Date decision made re. Treatment 7. Date treatment commenced. The mean (± SD) and median were calculated for the waiting times. These were compared with the national/departmental guidelines for GCT referrals.

**Results** There were 66% patients with Stage I GCTs and 22% were recommended surveillance. The mean duration of symptoms before attending the GP was 15.4 weeks (range 1–104 wks). 78% had tumour markers done preoperatively and only 61% had pre oncology referral CT scan of the chest, abdomen and pelvis. The mean, median and st. deviations are given in the table I. 66% of the patients were seen by the oncologist within four weeks of the orchidectomy. As shown in the table, the total waiting time is the composite effect of delays occurring at most steps of the referral pathway.

| Days | Total Waiting time | GP-Uro log y | Urology-surgery | Surgery-oncology referral | Oncology referral-oncol. OP | Oncol.OP decision Rx | Decis. -Rx start |
|---|---|---|---|---|---|---|---|
| Mean | 71 | 17 | 7.5 | 14 | 11.5 | 10 | 11 |
| Median | 73 | 10 | 6 | 9 | 10 | 7.5 | 7 |
| SD | 29.2 | 19 | 7 | 15.5 | 9 | 11 | 12.5 |

**Conclusions** We believe there are still significant delays occurring in the referral pathway of patients with GCTs of testis. 77% of the total delay seem to occur prior to oncology consultation. Only 66% of the patients were within the time frame recommended by the COIN guidelines, 1999.(1). We propose a fast track referral process to minimise the delays in the referral pathway. Patient education is essential in reducing the delays in seeking medical advice.

1 Howard G & Mead GM et al (1999) COIN and SIGN Guidelines on the management of adult testicular germ cell tumours.

### P90 COLORECTAL CANCER REFERRALS TO A DISTRICT HOSPITAL – DO WE NEED AN INTEGRATED CARE PATHWAY?

B Lovett*, D Hassanally, C Ingham-Clark, MR Lock, RAM Al-Mufti, Department of Surgery, The Whittington Hospital, Royal Free and University College Medical School, London N19 5NF, UK

**Aim** To evaluate the referral patterns and current clinical practice for colorectal cancer patients referred to a colorectal cancer unit. To compare these to NHS Executive guidance on improving outcomes in colorectal cancer[1] and with the guidelines issued by the Royal College of Surgeons of England[2].

**Method** The clinical notes of all patients referred with colorectal cancer in a one year period were reviewed retrospectively by a single observer. Recorded factors included the time from GP referral to first consultation, time from first symptoms to diagnosis, pre-operative diagnostic investigations, urgency of surgery, speciality of operating surgeon, requirement for stoma formation, use of adjuvant therapy and mortality rate.

**Results** The case notes of all 49 patients referred with colorectal cancer were reviewed. 68% of cases were seen within 2 weeks of referral. The longest delays in diagnosis, (median 4.5 months) was due to the prolonged interval between onset of symptoms and GP consultation. 36% of patients presented as emergencies. 17% of patients underwent full colonoscopy before surgery, 45% prepared Ba enema and 15% unprepared Ba enema. 39% of cases were biopsied preoperatively. 89% of elective cases and 56% of emergency cases were performed by a colorectal surgeon. The procedure was regarded as curative in 63% of cases and a stoma was required in 30%. Histology confirmed 6% Dukes A, 41% Dukes B and 41% Dukes C tumours. 34% of patients received adjuvant chemotherapy, 9% received radiotherapy and 4% received both. 55% of patients were discharged within 14 days of admission. Mortality was 6%.

**Discussion** Our current practice in the management of colorectal cancer referrals does not meet NHS executive guidelines. We identified a need for an integrated care pathway designed to streamline colorectal cancer diagnosis and treatment.

1 *NHS Executive* (1997) Guidance on commissioning cancer services. Improving outcomes in colorectal cancer. Department of Health

2 *The Royal College of Surgeons of England and Association of Coloproctology of Great Britain and Ireland* (1996) Guidelines for the management of colorectal cancer.

### P91 MANAGEMENT OF MALIGNANT PHAEOCHROMOCYTOMA: A RETROSPECTIVE REVIEW OF THE USE OF MIBG AND CHEMOTHERAPY IN THE WEST MIDLANDS

Hartley A*[1], Spooner D[1], Brunt AM[2], Lewington V[3], [1]Queen Elizabeth Centre for the Treatment of Cancer, Birmingham B15 2TH, [2]Staffordshire Oncology Centre, Stoke-upon-Trent, [3]Department of Nuclear Medicine, Southampton General Hospital, UK

**Introduction** Phaeochromocytoma is a rare disorder with no randomised and little prospective data to facilitate choice between the two main treatment modalities, chemotherapy and radiolabelled monoiodobenzylguanidine(mIBG). In the last decade the latter modality has been preferred and radiological response rates of 30% have been reported. There are fewer patients reported in the literature who have received chemotherapy but one prospective trial of chemotherapy reported radiological response rates of 57%. A recent prospective trial combining the two modalities has been disappointing with only one patient completing the treatment schedule. We present six patients with malignant phaeochromocytoma or paraganglioma who received mIBG therapy. Four patients also received chemotherapy.

**Method** A retrospective review of the case notes was performed. Radiological and hormonal responses were determined. Time to progression after each modality were calculated.

**Results** One partial hormonal response was seen with mIBG treatment. One complete and one partial hormonal response and one radiological partial response were seen with chemotherapy. The median time to disease progression from commencement of mIBG was 12 months (range 3–44) and from commencement of chemotherapy used as 1st or 2nd line was 22.5 months (range 7–25).

**Conclusions** Chemotherapy may be a more active modality in this disease than previously considered. MIBG uptake may increase following a partial radiological response to chemotherapy enabling subsequent mIBG therapy. Future trials looking at combined therapy should consider administering chemotherapy prior to mIBG for reasons we outline.

### P92 MANAGEMENT OF LUNG CANCER IN ABERDEEN: COMPARISON OF TREATMENT AND OUTCOMES WITH THE NATIONAL AVERAGE

Marianne C. Nicolson*, Donald Bissett, Muriel Reid and Robin Legge, ANCHOR Unit, Aberdeen Royal Infirmary, on behalf of the Aberdeen Lung Cancer Group

**Introduction** We know from the data presented last year that lung cancer is undertreated in Scotland. The practice in Aberdeen differs in that all patients treated come through this hospital and for five years now we have had a multidisciplinary group who meet weekly to discuss the new patients' diagnoses and management in addition to having bimonthly research meetings. The treatments and outcomes were audited for 1994, before the arrival of oncologists interested in lung cancer, and again in 1997. There has been a marked change in practice in the stable population in the North East of Scotland and this is reflected in outcomes here and in comparison with the rest of the country.

The number of patients diagnosed was 422 in 1997. The demographics are unremarkable with 265 males to 157 females and age range 35 to 95 years with mean 69 years. The diagnosis was confirmed histologically or cytologically in 71.3% of cases (62% in Scotland 1995). Squamous and adenocarcinoma histologies were equal in incidence, each accounting for 26.8% of patients. Small cell was diagnosed in 15.2% only. Primary treatment was chemotherapy in 22.3% of patients with non-small cell lung cancer compared with 16.6% in the national study. The majority of these patients were treated in a clinical trial. The percentage resected was 17% compared with 11.6% in Scotland in 1995. For small cell lung cancer, only 61% received chemotherapy as primary treatment, and a further 8 patients (12.5%) received second line chemotherapy.

In 1994, no patients in Grampian with non-small cell lung cancer received chemotherapy. Survival data for the 1997 audit will be available and further comparative data will be presented.

### P93 SCOTTISH AUDIT OF SURGICAL MORTALITY: AUDIT OF 182 DEATHS FROM PANCREATIC CANCER

MA Jahan*[1], H Burton[2], SJ Nixon[2], AM Thompson[1,2], [1]Department of Surgery & Molecular Oncology, University of Dundee DD1 9SY, [2]Scottish Audit of Surgical Mortality, Royal College of Physicians and Surgeons, Glasgow G2 5RJ, UK

The Scottish Audit of Surgical Mortality (SASM) conducts peer review of the management of all patients who die in surgical wards. The first round of audit of 334 patients who died with pancreatic cancer (1994–1996) identified a high rate of emergency admission, adverse surgical management and poor utilisation of perioperative monitoring resulting in some 25% of deaths being potentially avoidable. The aim of this study was to conduct a second round of audit using the most recent SASM data (1997–98) to evaluate changes.

During 1997–1998, 182 patients died with pancreatic cancer on surgical wards in Scotland. Compared with the previous audit (*1994–1996 figures in italics*), 77(42%) were emergency admissions (*qv 75%*) and 94 (40%) had terminal care (*qv 28%*). There was a reduction in potentially avoidable causes of death to 37/182 (20%) from 25% Nineteen (10%) deaths followed endoscopic procedures under sedation (*qv 23/334, 7%*). Adverse factors in surgical management (usually post-operative complications, detected too late and managed by inexperienced staff) were identified in 7 (11%) patients who underwent surgery (*qv 12%*).

Since the last audit, there has been a reduction in emergency admission rate and deaths due to potentially avoidable causes, but an increase in the proportion of patients receiving terminal care for pancreatic cancer on a surgical ward. However, endoscopic procedures, currently the subject of more detailed audit, and adverse factors in surgical management remain causes for concern.

### P94 A PROSPECTIVE AUDIT OF THE DELAYS IN REFERRAL AND TREATMENT OF MALIGNANT SPINAL CORD COMPRESSION

SG Russell[1] and LT Tan[1], [1]Dept of Oncology, Addenbrooke's Hospital, Cambridge CB2 2QQ, UK

**Introduction** Malignant spinal cord compression (MSCC) is a common condition and a major cause of morbidity in patients with cancer. The outcome depends on the functional status at the time of treatment. Delay is associated with loss of neurological function which may be irreversible. This prospective audit assesses the delays in presentation, investigation and treatment of MSCC at Addenbrooke's Oncology Centre.

**Methods** 40 consecutive patients with MSCC were interviewed with a structured questionnaire. The following data was collected: dates of onset of symptoms, presentation to GP, admission to District General Hospital, admission to Addenbrooke's, commencement on dexamethasone, MRI and definitive treatment. Functional status was also assessed at each of these times.

**Results** The median age was 66 (range=38–94) years. The commonest malignancy was prostate followed by breast and lung. The median delay from onset of symptoms to first consultation was 8 (range=0–38) days. The median delay after presentation to the GP was 2 (range=0–28) days. The median delay at the District General Hospital was 1 (range=0–5) day. The median delay at the Cancer Centre was 0 (range=0–2) day. 93% of patients were treated on the day of referral. Deterioration in functional status followed a similar pattern to the delays in referral: 25% of patients deteriorated before consulting a doctor, 10% after presentation to the GP and 5% at the District General Hospital. MRI was performed on the same working day on 38/40 (95%) patients; 1 patient was scanned the next working day while the other refused due to claustrophobia. 36/40 (90%) patients were started on dexamethasone the same day the clinical diagnosis of MSCC was made.

**Conclusion** The main delay in referral of MSCC in this audit was by the patient. It would therefore seem logical to target specific groups with a relatively high probability of developing MSCC for patient education. This may include patients with breast, prostate and lung cancer, myeloma and those with known bone metastases. These patients could be given an advice card outlining the symptoms of MSCC (pain, weakness, sensory disturbance, ataxia and bladder dysfunction) and advertising a direct referral route to the Cancer Centre. We feel that the relatively short delays experienced after the patient had presented to a doctor was in part due to the implementation of a co-ordinated multi-disciplinary approach involving oncology, neurosurgery and radiology with the aim of reducing delay.

### P95 MALIGNANT SPINAL CORD COMPRESSION: A SCOTTISH AUDIT

P Levack[1], I Kunkler[2], J Graham[2], R Grant[2], A Gibson[2], D Collie[2], P Statham[2], R Rampling[3], L Slider[3], D Hurman[4], [1]Royal Victoria Hospital, Dundee; [2]Lothian Univ Hospitals NHS Trust, Edinburgh; [3]Western Infirmary, Glasgow; [4]Aberdeen Royal Infirmary, Aberdeen, UK

Early diagnosis and treatment of malignant spinal cord compression (MSCC) are important in optimising the outcome of treatment. Delay in diagnosis or treatment may compromise neurological outcome. Little is known on a national basis of the process of care of MSCC. A prospective multidisciplinary audit was carried out of 300 patients in three Scottish cancer centres including the onset of symptoms and signs of MSCC and institution of radiological investigations and treatment. A preliminary analysis is presented on 200 patients. 80% had evidence of malignancy at least 2 weeks before the diagnosis of MSCC. Predominant primary sites were lung>prostate>breast. At presentation 14% were ambulant, 37% were ambulent with assistance and 44% were unable to walk (see Table). Radicular pain occurred in 83% at diagnosis and in one third was 10/10 on the VAS scale of severity. The median time from first reporting symptoms to a medical practitioner to diagnosis of MSCC was 75 days. 86% first reported symptoms to their GP. Most patients were initially investigated by their GP with plain radiographs. Many with thoracic MSCC (70% of all compressions) had had lumber spine films only. An additional 40% of patients had bone scans incurring further delay in diagnosis of MSCC. 90% were diagnosed-on-MRI. Eleven of the 30% referred for a neurosurgical opinion underwent surgery (2% for biopsy and 9% for definitive surgery). 86% were treated with radiotherapy of whom 7% received postoperative radiotherapy.

| Ambulation | Lung | Prostate | Breast | GI | Kidn | NK |
|---|---|---|---|---|---|---|
| None | 32 | 32 | 27 | 12 | 8 | 11 |
| With help | 27 | 23 | 14 | 12 | 10 | 7 |
| Walks alone | 9 | 11 | 14 | 7 | 3 | 6 |

**Conclusions** 1. A high proportion of patients present with MSCC unable to walk despite a long history of severe root pain suspicious of epidural disease. 2. Rapid access to MRI for patients with known malignancy and root pain might lead to earlier diagnosis and more effective treatment by surgery and/or radiotherapy.

### P96 AN AUDIT OF CLINIC CONSULTATION TIMES IN A CANCER CENTRE – IMPLICATIONS FOR NATIONAL MANPOWER PLANNING

RJ Benson*, NG Burnet, MV Williams and LT Tan, Oncology Centre, Addenbrooke's Hospital NHS Trust, Cambridge CB2 2QQ, UK

**Introduction** A departmental audit was conducted to assess the frequency, extent and causes of late completion of oncology clinics.

**Methods** Data were collected prospectively from clinical, medical, haematological and multidisciplinary oncology clinics. Factors recorded included clinic start and finish time, number of patients seen, type of consultation, number of doctors in each clinic, time spent by the doctor with the patient and other factors that may have contributed to late completion of clinics.

**Results** 848 patient consultations were recorded in 81 clinics. Of 67 clinics in which finish time was recorded, 19 (28%) were completed on time while 48 (72%) were late by a mean of 49 minutes. The mean time spent by consultants with new, follow-up and chemotherapy patients was 37, 21 and 22 minutes respectively. This time did not include time spent reviewing notes, dictation or ordering investigations. There was no significant difference in the time spent by specialist registrars compared to consultants, or clinical oncologists, medical oncologists and haematologists. Unforeseen problems such as difficult consultations (e.g. breaking bad news, difficult symptom control) occurred in 228/848 (27%) consultations while delays due to missing information occurred in 33/848 (3.9%) consultations. Unplanned interruptions occurred in 48/81 (59.3%) clinics and late starts in 13/67 (19.4%) clinics. 10/81 clinics (12%) were overbooked. There was no significant difference in the time lost to unforeseen factors between those clinics which finished late and those which finished on time. The mean delay of multidisciplinary clinics was longer than for non-multidisciplinary clinics (59 and 31 minutes respectively), despite a higher ratio of doctors to patients in the former (1:5.4 and 1:7 respectively).

**Conclusion** The audit found that the main cause of delay in clinics in our department was the longer than anticipated time spent by doctors with patients. Consultations are taking longer because of the increasing complexity of non-surgical cancer treatment and the greater emphasis placed on patient information and informed consent. The Royal College of Radiologists (RCR) has calculated that if a consultant oncologist sees a maximum of 315 new patients per year, the time available for each follow-up consultation would be 10 minutes. Our audit showed that follow-up consultations took an average of 21 minutes. These results suggest that the RCR recommendations for consultant expansion substantially underestimates the true number of consultants required for the treatment of cancer patients in the U.K.

### P97 THE EFFECTS OF RADIOTHERAPY WAITING TIMES ON THE TREATMENT OF GLIOBLASTOMA

M Kwok[1], JW Adlard*[1], G Gerrard[1], [1]Yorkshire Centre for Clinical Oncology, Cookridge Hospital, Leeds LS16 6QB, UK

**Introduction** Glioblastoma is an aggressive intracerebral glioma with a median survival of 9 months and a 5-year survival approaching 0%. Treatment with high doses of radiotherapy (RT) improves median survival by several months. A standard RT regimen is 60 Gy in 30 fractions over 6 weeks. A hypofractionated regimen of 30 Gy in 6 fractions over 2 weeks with a similar Radio-Biological Effectiveness has previously been piloted in poor prognosis high grade gliomas (1).

**Method** At the Authors' centre, patients with glioblastoma with poor prognosis (low Karnofsky Performance Status (KPS)/elderly) are treated with the hypofractionated (HF) regimen and the remainder with the standard (SD) regimen. Prior to a recent increase in linear accelerator capacity the RT waiting time has been longer for patients offered the more fractionated regimen. A retrospective study was performed to compare the survival outcome of the two groups, to determine whether patients deteriorate prior to treatment and to assess the potential impact of increased radiotherapy waiting times.

**Results** A total of 72 cases (43 SD group, 29 HF group) treated between March 1998 and January 2000 were reviewed. Pretreatment prognostic factors were worse for the HF group compared with the SD group (Mean age 60.8 years vs. 50.2 years, KPS 50–60 vs. 70–90). The mean RT waiting time was shorter for the HF group (17 days vs. 38 days). In the SD group two patients died and 8 deteriorated prior to starting RT compared with and one death and 2 deteriorations in the HF group. There was no significant difference in survival between the two groups (Median survival: HF group 8.3 months vs. SD group 10.9 months).

**Conclusion** This study suggests that increased radiotherapy waiting times are deleterious in glioblastoma and may eliminate any potential benefit of more fractionated regimens. Where resource limitations lead to unavoidable prolonged waiting times the hypofractionated regimen may be useful, but should be compared to standard regimens in randomised trials.

(1) Thomas R, James N, Guerrero D, et al (1994) *Radiotherapy and Oncology* **33**(2): 113–6

### P98 AUDIT OF SHORT COURSE PREOPERATIVE RADIOTHERAPY FOR OPERABLE RECTAL CANCER

A Hartley*, S Giridharan, JI Geh, The Cancer Centre at the Queen Elizabeth Hospital Birmingham B15 2TH, UK

**Introduction** Short course (25 Gy in 5 fractions over one week) preoperative radiotherapy (RT) has been shown to reduce the risk of local recurrence and improve overall survival in patients with operable rectal cancer. However, in view of improved surgical techniques, this advantage may be lessened. In addition, preoperative RT may not be necessary in all patients and there is a potential risk of postoperative complications, late toxicity and impaired functional results using this approach. The aim of this audit was to identify the proportion of patients in whom either short course RT may have been avoided or a prolonged fractionation may have been more appropriate.

**Method** An audit of all rectal cancer patients treated in this centre with pre operative RT during 1998 was performed. Information from clinical notes, surgical findings and pathology reports were obtained. All patients who received short course RT were included.

**Results** A total of 88 patients were identified; 70 patients with clinically operable tumours received short course RT, 13 patients with fixed tumours received prolonged fractionation, information on 5 patients pending. Of these 70 patients 64 (91%) had curative resection, 5 had palliative resection (3 had liver or peritoneal metastasis) and 1 was unresectable. Of the 64 who had curative resection, 57 (89%) had clear circumferential margins, 7 (11%) had positive margins. Pathological staging identified Dukes A 9 (14%), Dukes B 29 (45%) and Dukes C 26 (41%). Four (6%) patients died within 3 months of commencing RT and there were 26 treatment related early complications in 20 (29%) patients (wound complications (11); thromboembolic episodes (4); pelvic abscess (4); anastamotic leak (1); others (6)).

**Conclusion** A significant proportion of the 70 patients receiving pre-op RT were found to be incurable (9%) or to have Dukes A disease (13%). In addition, the 10% of patients who had positive circumferential margins may have had greater benefit from a more prolonged course of RT. Therefore, 32% of patients may have received short course RT inappropriately. In future, to avoid the problems identified, a greater emphasis on more accurate pre-operative assessment and staging is desirable. The results of the MRC CR07 trial, comparing short course preoperative RT with selective postoperative chemoradiation may help to resolve these uncertainties.

### P99 AN AUDIT TO COMPARE PLANNING TARGET VOLUMES USING CONVENTIONAL CT SCAN GREY SCALES PLANNING AND THE CT ACQSIM SYSTEM FOR RADICAL RADIOTHERAPY OF CARCINOMA OF THE PROSTATE

D Power, JF Money-Kyrle, S Allen, RSD Brown & HA Payne, Meyerstein Institute of Oncology, Middlesex Hospital, London W1N 8AA, UK

**Aims of Study** To evaluate the accuracy of target definition in prostatic carcinoma using AcQSim virtual simulation computer software and a Picker PQ 5000 CT Simulator (Picker, Ohio, USA) compared with our existing method of conventional CT grey scale planning. Study endpoints were the size of margin around clinical target volume (CTV), the size of planning target volume (PTV), and the possibility of geographical miss.

**Methods** Concurrent AcQSim and conventional grey scales CT planning was performed on 19 sequential patients. PTV's were calculated and compared for both groups.

**Results** For AcQSim plans, PTV were smaller in 14 patients and larger in 5 patients. The mean reduction in area when planned with AcQSim was 4.5% (246 $mm^2$). The maximum area reduction was 35.1% with a maximum increase of 22.9%. Length of PTV by AcQSim was reduced in 15 cases (maximum reduction 18 mm), unchanged in 2 cases and increased in 2 cases. Change in the PTV were largely determined by the smaller field length on AcQSim rather than the difference in area outlined. There was little difference in the posterior border (rectal) using the two methods. One partial geographical miss was seen with grey scale planning.

**Conclusions** The AcQSim gives a comparable PTV to the grey scales, but by using smaller CT cuts, the length of the volume is more accurately determined. The composite shape appeared to be more accurate on AcQSim, although this had less impact clinically. The system was easy to use and beam's eye lead can be applied to reduce rectal toxicity.

### P100 DO ENQUIRERS TO CANCER INFORMATION SERVICE HAVE SUPPORT AT HOME?

M Boudioni[1]*, J Mossman[1], A Jones[2], M Boulton[3], K McPherson[4], [1]CancerBACUP, 3 Bath Place, Rivington Street, London EC2A 3DR; [2]Royal Free Hospital, Pond Street, London NW3 2QG; [3]Social Science. & Law, Oxford Brookes University, Gipsy Lane Campus, Oxford OX3 0BP; [4]Epidemiology & Population Health, LSHTM, London WC1E 7HT, UK

**Aims** The aim of the study was to ascertain whether people living alone are more likely than those in multi-person households to contact independent cancer information services. It was postulated that the availability of support from others within a household would diminish the need for support from an organisation such as CancerBACUP.

**Methods** An Enquirer Record Form (ERF) is completed for every fifth enquirer to the Cancer Information Service. Demographic characteristics, subjects of enquiry and advice given are systematically and routinely recorded, but information on living arrangements is not collected.

A questionnaire was developed to record living arrangements of enquirers who were patients, relatives or friends. It was used in association with the routine ERF, for a five-week period (13/07/98 to 14/08/98). The Information nurse specialists were briefed on the purpose of the survey and how to apply it. Those callers who were too upset or emotional were not asked.

**Main findings** Of the 677 people who constituted the sample, 552 (82%) callers specified whether they were living alone or not. 250 (45%) of them were diagnosed patients and 302 (55%) were relatives/friends. 481 (87%) were living with someone, while 71 (13%) were living alone. These data were compared to the Great Britain Population[1] and it was found that a slightly higher percentage of people living alone contacted the service than those in the general population (13% Vs 11%). Living alone or with others was significantly associated with type of enquirer. 72% of those living alone were diagnosed patients compared with 41% of those living with others (chi-square=21.952, p=0.000). The recorded subjects of enquiry were also analysed and it was found that patients and relatives/friends living alone had different concerns from those living with others.

**Conclusions** This analysis provides an insight into the characteristics and needs of people using independent support services. Although there are no significant differences in the living arrangement of enquirers compared with the GB population as a whole, there are differences in the needs between those living alone and those living with others. These findings may enable healthcare professionals to begin to identify groups where there is a greater need for support.

1. Household and Families (1998, 1999) Social Trends 28 & 29. Office for National Statistics, London

### P101 SPECIAL SCREENING OF FAMILY HISTORY PATIENTS – DOES IT MEET GENERAL SCREENING STANDARDS?

K Hogben*, J Viggers, J Davey, G Curling, N Burch, B Kirkman, Newman M, G Walsh and G Gui, Breast Diagnostic Unit, The Royal Marsden Hospital NHS Trust, London SW3 6JJ, UK

Women with a significant family history of breast cancer attend specialist clinics for high risk screening, usually selected by a calculated lifetime risk greater than 1:8 compared to the population risk of 1:12. Such women are screened annually from age 35 years but there is currently no data to support the practise or to compare results against the National Health Breast Screening Programme (NHSBP). Women with a risk between 1:8 and 1:12 are considered low risk and advised to attend the NHSBP from age 50 years.

Women were categorised into lifetime risk of between 1:12 and 1:8 (low risk) and greater than 1:8 (moderate/high risk), based on Claus predictive tables. 2578 women were seen between June 1993 and August 1994 with a mean follow up of 120 (range 8–357) months in this retrospective study. Screening consisted of annual review and mammography.

| | Low risk | High risk | Total |
|---|---|---|---|
| Patients, n | 1500 | 1078 | 2578 |
| Follow-up in women years | 5902 | 4328 | 10230 |
| Median age at diagnosis | 54.5 (38–63) | 45 (26–66) | |
| DCIS, n | 0 | 2 | 2 |
| Invasive cancers <10 mm, n | 2 | 4 | 6 |
| 10–20 mm, n | 7 | 10 | 17 |
| 21–30 mm, n | 3 | 1 | 4 |
| Unknown, n | 0 | 2 | 2 |

As all women were already attending for screening at the start of the study, the cancer incidence was 2/1000 per year in Group 1 and 4.4/1000 per year in Group 2. NHSBP targets are to detect 4/1000 cancers per three yearly screen, which is equivalent to 1.3/1000 years. 24% of all invasive cancer in Group 2 were <10 mm.

The screening of high risk women meets and exceeds the rigorous targets set for the detection of invasive cancer in the incident screens of the NHSBP directed at the general population. The annual screening of high risk women with clinical examination and mammography is justified.

### P102 RESULTS OF THE NORTH THAMES DEANERY TRAINEE SATISFACTION SURVEY FOR CLINICAL AND MEDICAL ONCOLOGY SHO'S & SPR'S 1999

RSD Brown[1], D Power[1], M Aitken[2] & E Paice[2], [1]SpR in Clinical Oncology, Middlesex Hospital, London W1A 8AA, [2]North Thames Postgraduate Medical and Dental Education, 33 Milman Street, London WC1N 3EJ, UK

**Introduction** The North Thames deanery is the UK's largest, where 25% of all UK specialist registrars are trained. In November 1998 to January 1999 a North Thames deanery survey was widely distributed to North Thames hospitals to assess trainee satisfaction as part of the Calman training reforms. Results were available for all main medical subspecialties.

**Methods** 25 questions relevant to Clinical and Medical Oncology trainees were analysed. Tests for significant differences from other medical specialities were performed using Chi squared and students t-tests. Nine key questions were used to rank trainee satisfaction across the range of medical specialties.

**Results** Oncology SpR's ranked in the top half of medical specialty trainee satisfaction in 4 of 9 comparisons; night time rest [1st/13, p=0.03], nursing support [4th/13], hands on experience [5th/13] & overall post rating [5th=/13]. In 5 of 9 comparisons satisfaction was in the lower range; hours of formal education [7th/13], consultant supervision [10th/13], job intensity [10th/13], induction to post [12th/13, p=0.04], & feeling forced to cope [13th/13].

Oncology SHO's also ranked in the top half of medical specialty trainee satisfaction in 4 of 9 comparisons; feeling forced to cope [1st/13], nursing support [2nd/13, p=0.01], night time rest [5th/13, p<0.05], consultant supervision [6th/13, p=0.03]. In 5 of 9 comparisons, satisfaction was in the lower range; job intensity [8th/13], induction to post [11th/13], overall post rating [13th/13, p<0.05], hands on experience [13th/13] & hours of formal education [13th/13, p=0.04].

**Conclusion** Overall satisfaction scores among North Thames oncology trainees are generally high. Areas for possible improvement compared with other medical specialties are defined.

### P103 WOMEN WITH OVARIAN CANCER: THEIR PERCEPTIONS OF CT SCANNING

BA Crosse*[1], J Spencer[2], TJ Perren[3], [1]Huddersfield Royal Infirmary, HD3 3EA,[2] Department of Clinical Radiology and [3]Imperial Cancer Research Fund Cancer Medicine Research Unit, St James's University Hospital, Leeds LS9 7TF, UK

Abdomino-pelvic CT scanning makes an important contribution to the management of women with ovarian cancer. The optimum method of disease monitoring is much debated. [1]Women may undergo several CT scans during the course of their disease. In clinical practice, this procedure appeared to cause anxiety for some, and considerable distress for a few. The impact of scanning on cancer patients has been studied infrequently,[2] and there is no published work in the context of ovarian cancer. A semi-structured questionnaire was designed to elicit the views of women with ovarian cancer on the experience of CT scanning. 61 fixed response items were derived from unstructured interviews with 6 women, and with clinical and radiology personnel. Items were ordered chronologically, covering the period prior to the scan, preparation for the scan, the scan procedure and the period afterwards. Items included anxieties occasioned by the scan, physical symptoms, knowledge of the procedure, and issues of consent and control. Women were asked to refer to their most recent scan when responding. Open responses were invited to questions about change of experience with repeated scanning, the best and worst aspect of scanning and areas for improvement. 50 women received the questionnaire, and 45 responded. Their ages ranged from 38 to 83 years (median 58) and the number of scans per woman from 1 to 12 (median 4). 33% of women dreaded the scan because it was so unpleasant. Physical symptoms occasioned by the oral and intravenous contrast fluids (particularly diarrhoea and vomiting), and the unpleasant nature of the oral contrast were distressing aspects. Anxieties related to ignorance of the procedure, invasive elements of the process, physical consequences, the scan environment, and waiting to hear the results. Important minorities of women felt they had no choice about having a scan, or that they had no control over unpleasant or invasive aspects. Fear of the procedure generally lessened with repeated scanning, but physical symptoms did not. Women expressed confidence in the accuracy of scanning and its contribution to their management.

Ovarian cancer is an unpleasant and distressing disease. This study presents evidence that CT scanning causes distress for some women, and this should be considered when decisions about disease monitoring are made.

1. Rustin GJ, Nelstrop AE, McClean P et al. (1996) *J Clin Oncol*; **14:** 1545
2. Peteet JR, Stomper PC, Ross DM, Cotton V, Truesdell P, Moczynski W (1992) *Radiology* **182:** 99.

### P104 WEIGHT GAIN DURING CHEMOTHERAPY MAY BE A SIGNIFICANT PROBLEM FOR BREAST CANCER PATIENTS – AND SHOULD BE ADDED TO INFORMATION SHEETS

KJ Lankester*, PA Lawton, JE Phillips, Centre for Cancer Treatment, Mount Vernon Hospital, Rickmansworth Rd, Northwood HA6 2RN, UK

Several reports in the literature have shown that weight gain can occur during adjuvant chemotherapy for breast cancer. Despite this, current dietary advice in many centres is to eat small frequent snacks (such as biscuits) to combat nausea, which could contribute to weight gain. We conducted an audit to investigate weight change in our own centre and to examine possible contributory factors.

**Methods** Case notes from 50 patients who had received either adjuvant or neoadjuvant FEC or CMF chemotherapy for breast cancer were studied. Cancer stage, menopausal status, type of chemotherapy, weight at each cycle of chemotherapy, use and dose of steroids, antiemetics and tamoxifen were recorded.

**Results** The mean weight change was a gain of 3.9 kg (range –3.2 kg to 17.9 kg, standard deviation 3.7 kg). This was highly significant with a p-value of <0.001. Weight gain in this study appeared to be independent of steroid use, antiemetics, menopausal status and tamoxifen.

Gaining a few kilograms may be dismissed as a trivial side effect. However, it can impact significantly on self-image and hence quality of life and general health. We propose that patients should be made aware of possible weight gain and that the current dietary advice should be modified.

### P105 AUDIT OF A NURSE LED PERIPHERALLY INSERTED CENTRAL VENOUS CATHETER (PICC) PRACTICE

K Clayton*, S Hummerston, JD White, PJ Woll, J Carmichael, Cancer Research Campaign Dept. of Clinical Oncology, Nottingham City Hospital, Nottingham, UK

**Introduction** Due to the costs and complications of Hickman lines in our unit we introduced peripherally inserted central catheter (PICC) lines in preference.

**Methods** A retrospective audit of our experience using PICCs was undertaken. A database (using SPSS version 8.0, Chigaco, IL.) was established. This included indication for insertion, complications and their management, time remaining in situ. Protocol for insertion included a check CXR.

**Results** 47 PICCs were inserted in 41 pts (22 M and 19 F) over a 34-month period, median age 56 years (19–82). Underlying tumours were colorectal 27, breast 5, pancreas, sarcoma, germ cell, cholangiocarcinoma each 3 and 3 other tumours. Indications for PICC insertion were intermittent continuous infusion chemotherapy 45%, continuous infusion chemotherapy 34%, poor peripheral access 17% and 4% other. Regimes administered were 45% De Gramont, 28% continuous infusion 5-FU and 28% 12 other regimes 83% of PICCs were single lumen. The left arm was used for 64% of insertions. 62% of PICCS were inserted successfully at first attempt, 22% needed two, 13% three and 2% required 4 attempts to cannulate. Complications occurring at the time of insertion 59% nil, 27% inability to cannulate leading to repeat attempts, median 1 (1–4), 5% of PICCs were mal-positioned and 7% could not be advanced. Indications for removal of line were 60% completion of treatment, 8% each for thrombosis, patient choice and mal-position and 3% each for fracture, occlusion and leakage. PICC fracture occurred at the hub (1) and entry site (1), necessitating repair and removal. Line removal (2) and systemic anticoagulation (1) treated the three cases of thrombosis, axillary (2) and subclavian (1). One PICC complicated by leakage was treated by removal. PICC infections consisted of 2-exit site (treated 1 with oral and one with oral and i.v. antibiotics) and a probable bacteraemia treated with oral antibiotics. There were no proven cases of bacteraemia. 7 PICCs were complicated by mechanical phlebitis. PICC occlusion occurred twice one treated by urokinase bolus and one by removal. There was a trend to longer PICC survival for lines placed in the right arm. Whilst not achieving statistical significance the mean time catheters remained in situ was longer for single lumen PICCs compared to double lumen. Median overall PICC survival was 76 days (2–212); corresponding figures for PICCs removed due to complications and those removed on completion of treatment were 44.5 (2–128) and 107 (16–212).

**Conclusion** This method facilitates easy recording and audit of our PICC practice and in the future other central venous access devices.

### P106 METHYLATION AT THE TELOMERASE RNA GENE PROMOTER, INDUCES ALTERNATIVE METHODS OF TELOMERE LENGTH MAINTENANCE IN HUMAN CELLS

SF Hoare*, L Bryce, S Muir and WN Keith, CRC Department of Medical Oncology, University of Glasgow, CRC Beatson Laboratories, Bearsden, Glasgow G61 1 BD, UK

We have studied methylation of the promoters of the telomerase genes hTERC (the RNA component) and hTERT (the catalytic subunit). Telomerase is present and active in most tumours but absent in normal somatic cells. It acts by maintaining telomere length, thus preventing cells from senescing. Both genes have promoter regions with a high CpG content, so have the potential to be regulated by methylation. Methylation was studied by both Southern blotting and methylation specific pcr (m.s.p.) on DNA samples from tumour and normal tissues and cell lines. Methylation status was confirmed by sequencing. Of the normal samples tested none showed methylation for either gene, suggesting that the absence of telomerase was not due to promoter methylation. The tumours types studied were lung, breast, colon, cervix, ovarian and brain. No methylation of the hTERT gene was observed in any tumour sample, but 7/49 ovarian tumour samples showed partial hTERC methylation. None of the other tumours showed hTERC methylation. 21 cell lines were tested, of the 18 cell lines which expressed telomerase, the hTERC promoter was unmethylated in 11 and partially methylated in 7. Full methylation of the hTERC promoter was only seen in the three ALT Cell lines, SUSM -1, KMST -6 and W138 -SV40, none of which express telomerase. Two other ALT cell lines were both unmethylated, but these cell lines express hTERC. This suggests that methylation might be the mechanism used by some ALT cell lines to switch off telomerase expression, forcing telomere length to be maintained by alternative methods. hTERT results, however, did not correlate with telomerase expression. Of the 20 cell lines tested, 1 cell line (SUSM -1) showed complete methylation, 5 cell lines were unmethylated and 14 were partially methylated. 10/20 cell lines showed similar methylation status in the promoters of both genes. We conclude that promoter methylation is unlikely to be involved in the regulation of telomerase expression except possibly in the ALT cell lines, where promoter methylation necessitates telomere length to be maintained by methods other than telomerase. Further studies are underway to investigate whether telomerase expression can be reactivated in the ALT cell lines by treatment with the demethylating agent 5-aza-cytidine.

### P107 METHYLATION STATUS OF P16 IN BREAST CANCER CELL LINES, NORMAL BREAST TISSUE, BREAST TUMOURS AND LYMPHOCYTES

VH Deshmane*[1,2], S Droufakou[1], AM Hanby[2], WH Harris[2], IS Fentiman[2] and IR Hart[1], The Richard Dimbleby Dept of Cancer Research/ICRF Laboratory[1], St Thomas' Hospital & Breast Unit, Guy's Hospital[2], London, UK

The cell cycle is strictly controlled by a complex system of molecules, which regulate the progress of the proliferating cell through various checkpoints. These include the retinoblastoma gene product (pRB), cyclins, cyclin dependent kinases (CDKs), and CDK inhibitors (CDKIs). One such CDKI is $p16^{INK4}$ which controls progress from G1 to S phase. Inactivation of p16 occurs commonly in human neoplasia. It is the predominant tumour suppressor gene located on 9p21, a region frequently affected by LOH. However, homozygous deletions and mutations of p16 are rare, which raises questions as to how this gene is inactivated. Methylation of the promoter region of this gene provides one possible mechanism.

We have analysed the methylation status of the p16 promoter following modification by sodium bisulphite. Using Methylation Specific PCR (MSP) cell lines (n=4), normal breast tissue (n=8), breast cancer tumours (n=65) and circulating lymphocytes (n=6), all have been examined. MSP with the T47D cell line demonstrated a methylated promoter with a product of 150 bp, whereas MDA MB 435, BT474 and ZR75 cells have an unmethylated promoter generating a product of 15 bp. Specificity of MSP products was confirmed by sequencing, demonstrating the conversion of all cytosines to uracil in the unmethylated promoter. Normal breast specimens all (8/8) appeared to have a methylated promoter, in circulating lymphocytes 4/6 (67%) were unmethylated while in 2/6 (33%) there was some degree of methylation, along with a strong unmethylated signal. Among tumour samples 52/65 (80%) were methylated, while 13/65 (20%) were unmethylated. On further analysis grade I tumours, lymph node-negative tumours, patients with <3 positive lymph nodes and ER/PR positive tumours showed a definite trend to have only unmethylated alleles i.e. active p16. However, after a median follow-up of 10 years, methylation status of the p16 promoter did not correlate with disease free survival (p=0.738) or overall survival (p=0.879). Our results indicate that while p16 appears to be activated to a greater degree in those with apparently good prognosis tumours, this does not associate with survival. These findings suggest that while inactivation of p16 gene expression by methylation may appear to provide a frequent means of silencing this CDK1 in breast cancers this also occurs in normal tissues.

### P108 THE ROLE OF $P21^{CIP1/WAF1}$ AND $P27^{KIPI}$ IN THE GROWTH INHIBITION OF COLON CARCINOMA CELLS BY TRANSFORMING GROWTH FACTOR-β1

JL Limer*, JA Royds and J Lawry, Institute for Cancer Studies, Division of Oncology and Cellular Pathology, Sheffield University Medical School, Beech Hill Road, Sheffield, South Yorkshire S10 2RX, UK

Transforming Growth Factor-β1 (TGF-β1) is a potent repressor of epithelial cell proliferation, inducing cell cycle arrest at the G1 restriction point. Neoplastic transformation of colonic epithelia is frequently associated with the acquisition of a TGF-β1 resistant phenotype. At present, the molecular mechanisms responsible for this occurrence are poorly understood. In order to investigate TGF-β1 insensitivity we have identified two adenocarcinoma cell lines (HT29 and SW742) with opposing responses to the cytokine. The HT29 cell line is significantly growth inhibited by 1 ng/ml TGF-β1, demonstrating concurrent G1 accumulation and decreased S-phase synthetic activity. TGF-β1 exposure induced upregulation of the cyclin dependent kinase inhibitor (CDKI) proteins $p21^{CIP1/WAF1}$ and $p27^{KIPI}$, as detected by flow cytometry. Immunoprecipitation studies confirmed increased CDKI interaction with CDK2 and CDK4 in the presence of TGF-β1. Contrastingly, SW742 demonstrates insensitivity the cytokine, exhibiting an increase in S-phase progression and decreased G1 accumulation. The SW742 cell line expresses elevated basal levels of $p27^{KIPI}$, however minimal CDKI-CDK binding was observed in response to TGF-β1. In summary, our results suggest a direct correlation between TGF-β1 insensitivity and a failure to inhibit G1 cyclin-CDK activity. We are currently utilising antisense oligonucleotide techniques to further investigate the role of $p27^{KIPI}$ in the TGF-β1 inhibitory response.

This work is funded by Yorkshire Cancer Research.

### P109 RAPID PROTEOSOME-DEPENDENT DEGRADATION OF P21 IN HUMAN TESTICULAR GERM CELL TUMOURS

Bate, TE*, Hickman, JA† & Chresta, CM, School of Pharmacy, University of Manchester M13 9PT, UK †Institut Servier, 92150 Suresnes, Paris, France

Overexpression of wtp53 is a frequent alteration in human testicular germ cell tumours (TGCT). Following etoposide treatment, cells derived from these tumours continue to proliferate with damaged DNA. We have investigated whether defects in the $p21^{waf-1/cip-1}$ pathway allow TGCT to tolerate these high levels of wtp53. In the TGCT cell line (833K) with wild-type p21, transcripts are unregulated in a p53-dependent manner in response to DNA damage. However, p21 protein is expressed at negligible levels and Rb remains in the inactive hyperphosphorylated form. The low levels of p21 protein resulted from reduced stability of the protein, as treatment with cycloheximide revealed the p21 of TGCT has a shorter half-life than that of bladder carcinoma cells (RT4) which undergo a stable cell cycle arrest. The reduced p21 stability results from enhanced proteolysis in the presence of proteosome inhibitors (MG132 and lactacystin) p21 protein accumulates to high levels. The effect of proteosome inhibitors on p21 stability is much greater in TGCT than in a wild-type p53 containing RT4 cells which has stable p21 protein. However, stabilisation of p21 in TGCT by proteosome inhibitors did not result in a functionally active protein as cell cycle analysis by bromodeoxyuridine staining showed no G1 phase accumulation. Additionally, analysis of Rb after proteosome inhibition showed no evidence of the hypophosphorylated form which would be consistent with a G1 arrest. We conclude that low levels of p21 protein occur in TGCT due to its rapid degradation by the proteosome: it may therefore act as an assembly factor, rather than an inhibitor of cyclin/CDK complexes. However, proteosome inhibitor treatment of TGCT does not result in the production of a functionally active p21 protein.

### P110 CELL PROLIFERATION AND APOPTOSIS CONTROL IN NON-HODGKIN'S LYMPHOMA

SF Al-Othman[1], JR Goepel[2], H Al-Husaini[–3], J Martin[4], Olivia Smith[2], J Lawry[1], [1]Division of Oncology and Cellular Pathology, [2]Department of Pathology, University of Sheffield Medical School, Beech Hill Road, Sheffield, S102RX. [3]Department of Histopathology, Riyadh Armed Forces Hospital; Riyadh; KSA, [4]Department of Histopathology, King Faisal Specialist Hospital & Research Centre, Riyadh, KSA

**Aims** To investigate by flow cytometry and immunohistochemistry, the expression of four regulatory proteins having a key role in the processes of cell proliferation & apoptosis control (p53, bcl-2, bcl-6 and cyclin D3) in non-Hodgkin's Lymphoma.

**Methods** 1– Three human cell lines were used: Raji, (Burkitt's) MC116 (undifferentiated lymphoma) and WILCL (normal B cell). All cells were maintained in suspension culture in RPMI-1640 supplemented with 10% fetal bovine serum. Cells were analysed by flow cytometry for expression of the four regulatory proteins.

2 – Paraffin wax embedded tissue was used in a parallel study from 35 cases of non-Hodgkin's Lymphomas (14 follicular lymphoma, 11 diffuse large B-cell Lymphoma of primary lymph node and 10 diffuse large B-cell lymphoma of primary gastric origin). Tissues have been stained with specific antibodies using immunohistochemical techniques. Samples were scored for staining distribution and intensity using appropriate controls.

**Preliminary results** (1) Cyclin D3 & bcl-6 were negative in all three-cell lines, but bcl-2 was positive in both WILCL and Raji, and very high expression of p53 in Raji. (2) Immunohistochemistry showed positive Cyclin D3 & p53 in about 50% cases of both nodal and gastric non-Hodgkin's Lymphoma, being more common in high grade. Bcl-6 was more commonly expressed in diffuse large B-cell lymphoma of primary gastric origin than its nodal counterpart. Bcl-2 staining was positive in 70–90% of the cases tested and was also more commonly expressed in follicular lymphoma.

**Conclusions** In-vitro studies indicated that the cell lines were a good model for the in-vivo studies, and apoptosis inhibition may be the key to this cancers development.

Low-grade disease was characterised by the lack of Cyclin D3 and p53 and conversely, by the presence of bcl-2. The reciprocal expression of these proteins may provide the "switch" from low grade to high grade B-cell non-Hodgkin's Lymphoma and also the cancers' apoptotic response.

Bcl-6 however, may play a more important role in the pathogenesis of gastric large B-cell lymphoma as these cases were characterised by elevated expression of bcl-6 compared to follicular and diffuse nodal lymphoma.

The Kingdom of Saudi Arabia and Yorkshire Cancer Research provided funding.

### P111 CELL CYCLE CONTROL AND APOPTOSIS IN NEUROBLASTOMA

SL Hankin*[1], M Gerrard[2], S Variend[2] & J Lawry[1], [1]Division of Oncology and Cellular Pathology, University of Sheffield, Beech Hill Road, Sheffield, S10 2RX, [2]Sheffield Children's Hospital, Sheffield S10 2TH, UK

**Introduction** Neuroblastoma is the most common extracranial malignant tumour in childhood, displaying a wide range of behaviour from spontaneous regression to highly malignant disease. The reasons for this diversity have yet to be elucidated using either growth control and/or apoptotic mechanisms.

**Aims** This study aims to decipher the specific growth control mechanisms that govern both the cell cycle control and apoptotic processes in neuroblastoma. We are trying to combine behaviour observed 'in vivo', using immunohistochemical methods, to an 'in vitro' analysis using two established neuroblastoma cell lines.

**Methods** Initial studies involved an 'in vivo' assessment of archival paraffin wax embedded tumours, with a panel of antibodies using immunohistochemical techniques. The antibody panel included cell cycle players such as CDK4, CDK6, and apoptotic associated-proteins such as Bax, Bcl-2 and CD95. Other antibodies employed included the prognostic markers N-Myc, NB84 and CD44. This same antibody panel was then used as part of an 'in vitro' investigation, which quantified protein expression using flow cytometry on the two cell lines Kelly and SK. N.SH. Results were expressed as fold increases, using the median fluorescence index (MFI) of each test protein, compared to the negative isotype control. The cell cycle phases (propidium iodide staining) and apoptosis (Annexin V staining) were also assessed in normal culture conditions and serum-deprived conditions.

**Results** Amongst other findings, preliminary results with immunohistochemistry indicate that neuroblastoma growth may be particularly associated with CDK4, as opposed to CDK6. Flow cytometry on the cell lines showed increased expression of CDK4 (Kelly MFI=3.5, SK.N.SH MFI=4.6) compared to CDK6 (Kelly MFI=1.7, SK.N.SH MFI=2.8). In addition, N-Myc protein was highly expressed (MFI=5.9) in both cell lines. N-Myc positivity was also evident in each clinical stage of neuroblastoma tested by immunohistochemistry, including the patients with no recorded N-Myc gene amplification.

**Conclusions** Initial data suggest that N-Myc protein expression, as opposed to gene amplification, may be a more valuable prognostic factor. It is anticipated that this dual approach of both cell cycle control and apoptotic analysis will provide essential data on an exact mechanism of growth control in neuroblastoma, which may provide information for therapeutic intervention.

Research is funded by Yorkshire Cancer Research.

### P112 DIFFERENTIAL EFFECTS OF ESTRONE- AND ESTRONE-3-*O*-SULFAMATE DERIVATIVES ON MITOTIC ARREST, APOPTOSIS AND MICROTUBULE ASSEMBLY IN HUMAN BREAST CANCER CELLS

Graham Packham*[1], MacCarthy-Morrogh[2], PA Townsend[1], A Purohit[2], HAM Hejaz[3], BVL Potter[3] and M Reed[2], [1]CRC Wessex Medical Oncology Unit, Cancer Sciences Division, School of Medicine, University of Southampton, Southampton, SO16 6YD, [2]Endocrinology and Metabolic Medicine and Sterix Ltd, Imperial College School of Medicine, St Mary's Hospital, London W2 1NY, [3]Wolfson Laboratory of Medicinal Chemistry and Sterix Ltd, Department of Pharmacy and Pharmacology, University of Bath, Bath BA2 7AY, UK

There is considerable interest in the potential use of estrogen derivatives for treatment and prevention of breast cancer. The naturally occurring estradiol metabolite, 2-Methoxyestradiol (2-MeOE2) does not bind the estrogen receptor, but does interact with tubulin and induce mitotic arrest in breast cancer cells, and has anti-angiogenic activity. We previously synthesised the sulfamoylated estrone derivative, 2-Methoxyestrone-3-*O*-Sulfamate (2-MeOEMATE), and demonstrated that this compound induced G2/M arrest and modest levels of apoptosis in breast cancer cells in vitro whereas the parent estrone derivative, 2-Methoxyestrone (2-MeOE1), did not, suggesting that sulfamoylation may be a useful way to increase the anti-cancer activity of estrogen derivatives (Purohit et al., 2000, Int J Cancer, *85*, 584). 2-MeOEMATE also induced breast tumor regression in vivo in intact rats. To further explore the significance of sulfamoylation on the anti-cancer activity of estrone derivatives and to elucidate their mechanism of action, we synthesised two additional agents, 2-Ethylestrone (2-EtE1) and 2-Ethylestrone-3-*O*-Sulfamate (2-EtEMATE). 2-MeOEMATE and 2-EtEMATE inhibited the growth of a panel of estrogen receptor negative and positive breast cancer cell lines in vitro, induced mitotic arrest and apoptosis and suppressed the long term clonogenic potential of MCF7 and CAL51 breast cancer cells. In each assay, the sulfamoylated estrone derivatives were greater than 10-fold more potent than their parent compounds. The sulfamoylated estrone derivatives were also significantly more potent inhibitors of cell growth than 2-MeOE2. 2-MeOEMATE and 2-EtEMATE functioned as anti-microtubule agents and inhibited the ability of paclitaxel to promote tubulin assembly in vitro. Like other anti-microtubule agents, the sulfamoylated estrone derivatives induced BCL-2 and $BCL-X_L$ phosphorylation and increased p53 expression. 2-MeOEMATE and 2-EtEMATE are novel anti-microtubule agents that have potent anti-cancer activity in breast cancer cells in vitro and may be beneficial as anti-cancer agents in vivo.

### P113 FUNCTIONAL AND MOLECULAR CHARACTERISATION OF THE VOLUME-REGULATORY CHLORIDE CURRENT IN THE EPITHELIAL DERIVED HUMAN BREAST CANCER CELL LINE ZR-75–1

J Killey*, AJ Nicholl, MW Saul, C Garner, Dept of Chemical and Biological Sciences, University of Huddersfield HD1 3DH, UK The Isle of Man Government has funded this project.

When a cell in isotonic solution is exposed to a hypotonic solution water will move into the cell causing it to swell due to the higher osmotic pressure in the cell. As a cell will always try to remain at a constant volume, under these conditions it will activate volume regulatory channels, resulting in a loss of specific ions, $Cl^-$ and $K^+$, from the cell and subsequently a loss of water. The molecular equivalent of the volume-regulatory $Cl^-$ current, $I_{clvol}$ has been proposed as either P-glycoprotein, the putative $Cl^-$ channel or $Cl^-$ channel regulator ($pI_{cl}$), or a member of the $Cl^-$ channel family (CIC). (Duan et al., 1997). The aim of this work was to identify the volume-regulatory $Cl^-$ currents in the ZR-75-1 cells using a hypertonic pipette solution in the whole-cell patch-clamp configuration. The molecular identity of this channel was also investigated using RT-PCR.

Cells bathed in NaCl isotonic saline (pH7.4; 285 mOsm/kg$H_2$O) and dialysed with hypertonic pipette solution (pH7.4; 370 mOsm/kg$H_2$O) containing 5 mM ATP, displayed an initial maximum outward $Cl^-$ current of 1.7±0.4 pA/pF (n=7, mean ±SEM). After 10–15 m the maximum outward $Cl^-$ current increased to 8 ±0.86 pA/pF (P<0.001 paired t-test). The current was outwardly rectifying and inactivated at depolarising potentials. The reversal potential, –40 mV was the same as the equilibrium potential for $Cl^-$, indicating the current was carried by $Cl^-$. The maximum outward current was inhibited by 100 μM DIDS 60–80% (n=3). Total RNA from ZR-75-1 cells was reversibly transcribed into first strand cDNA in an oligo (dT) primed reaction using AMV RT and PCR carried out using the specific primers for CIC2, CIC3, CIC5, CIC6 and $pI_{cl}$. Products were identified for all except CIC6.

This study identifies a functional volume-regulatory $Cl^-$ current in the ZR-75-1 cells and the mRNA for the ClC2, ClC3, ClC5 and $pI_{cl}$. P-glycoprotein and $pI_{cl}$ are not thought to be $Cl^-$ channels or part thereof, and previous results have shown that the ZR-75-1 cells do not possess the mRNA for the *MDR*1 P-glycoprotein (Killey et al., 1999). When expressed in apical membrane vesicles of HT29 cells, the characteristics of ClC2 differ from native $I_{clvol}$. The properties of expressed ClC3 and ClC5, however, have been shown to be similar to native $I_{clvol}$. This means that the most likely candidate for $I_{clvol}$ is ClC3 and/or ClC5 (Hagos et al., 1999). Future work will investigate the characteristics of the volume regulatory $Cl^-$ conductance in ZR-75-1 cells to confirm the molecular identity.

Duan D, Winter C, Cowely S, Hume JR, Horowitz B (1997) *Nature* **390:** 417–421
Hagos Y, Krick W, Burckhardt G (1999) *Eur J Physiology* **437:** 724–730
Killey J, Nicholl AJ, Saul MW, Garner C (1999) *J Cancer Research*: 40P, 46P

### P114 VOLUME REGULATION IN BREAST CANCER CELLS USING A VIDEO-IMAGING TECHNIQUE

AJ Nicholl*, J Killey and C Garner, Dept of Chemical and Biological Sciences, University of Huddersfield, Queensgate, Huddersfield HD1 3DH, UK

Most mammalian cells are able to regulate their volume when challenged with changes in osmolality. A decrease in external osmolality causes cell swelling as water enters. Transport pathways allow efflux of $Cl^-$ and $K^+$ with water following passively, this is known as regulatory volume decrease (RVD). An increase in external osmolality causes cell shrinkage as water leaves. Transport pathways allow a net influx of $K^+$ and $Cl^-$ and water enters passively, this is known as regulatory volume increase (RVI; Sachs, 1996).

This study investigates the mechanisms by which the epithelial derived human breast cancer cell line ZR-75-1 regulates its volume. Cells were bathed in $HCO_3^-$ buffered isotonic solution at 22 °C for 3 min, exposed to either a hypotonic or hypertonic solution for 15 min before being returned to isotonic solution for a further 12 min. Photographs were taken every minute and the area in pixels measured using the Scion Image computer software package. Volume was then calculated and expressed as relative volume (mean ± SEM) with respect to control values (Park et al., 1994) and % RVD or RVI was calculated. The role of ion channels, co-transporters and exchangers was investigated using specific inhibitors.

In 30% hypotonic solutions the cells swelled to 1.23 ± 0.040 (n = 7) in 4 min and recovered to 1.10 ± 0.026 within 15 min (56% RVD). RVD was reduced to 8.7% (n = 6) by 10 μM Tamoxifen and 10.7% (n = 7) by 100 μM DIDS suggesting the involvement of a volume regulated $Cl^-$ channel. In 30% hypertonic solutions cells shrunk to 0.84 ± 0.004 (n = 7) in 3 min and recovered to 0.96 ± 0.010 within 15 min (75% RVI). RVI was reduced to 40% (n = 6) by 500 μM DIDS, an inhibitor of the $Cl^-$-$HCO_3$ exchanger, 17.6% (n = 7) by 10 μM amiloride, an inhibitor of the $Na^+$-$H^+$ exchanger and 13.3% (n = 7) by 20 μM bumetanide, an inhibitor of the $Na^+$-$K^+$-$2Cl^-$ co-transporter. These results indicate the involvement of each of these exchangers and co-transporters in RVI.

In conclusion, ZR-75-1 cells act as perfect osmometers and are capable of both RVD and RVI. RVD involves volume regulated $Cl^-$ channels and probably $K^+$ channels. RVI involves uptake of $Na^+$ and $Cl^-$ via the coupled $Na^+$-$H^+$ and $Cl^-$-$HCO_3^-$ exchangers or via the $Na^+$-$K^+$-$2Cl^-$ co-transporter. The accumulated $Na^+$ is then exchanged for external $K^+$ via the $Na^+K^+$ ATPase pump, resulting in the net accumulation of KCl (Sachs, 1996).

Sachs JR (1996) Molecular Biology of membrane transport disorders, edited by SG Schultz et al. Plenem Press, New York 379
Park KP, Beck JS, Douglas IJ Brown PD (1994). *Journal of Membrane Biology* **141:** 193

### P115 ABROGATION OF SIGMA-MEDIATED SURVIVAL LEADS TO APOPTOTIC DEATH OF TUMOUR CELLS

BA Spruce[1], J Samson[1], D McLean[1], L Copeland[2], L Campbell[1] J Howie[1], D Belelli[2], J Lambert[2], A Thompson[1], S Eccles[3], [1]Depts of Surgery and Molecular Oncology, and [2]Pharmacology and Neurosciences, University of Dundee, Ninewells Hospital and Medical School, Dundee DD1 9SY; and [3]Institute of Cancer Research, McElwain Laboratories, Sutton, Surrey SM2 5NG, UK

Sigma receptors, once classed as members of the opioid receptor family, are now known to be molecularly distinct; a functional relationship exists, nonetheless. We have discovered that opioid systems participate in regulation of the apoptotic cell death programme and are dysregulated in tumorigenesis. Sigma receptors (subtypes 1 and 2), which reside at least partly on the endoplasmic reticulum, are widely present in human tumours *in vivo* and *in vitro*; an overabundance of sigma receptors in tumour compared to non-tumour tissues has permitted specific sigma ligands to be used in PET scanning for diagnostic tumour imaging. The biological significance of sigma receptors in the genesis of tumours is unknown.

We have shown that sigma ligands which are generally perceived to be antagonistic in their action (such as rimcazole and haloperidol) induce programmed cell death in tumour cell lines. Death induction by sigma antagonists does not require the presence of functional p53 protein, although modestly overexpressed p53 enhances their pro-apoptotic effect. Although death is induced in a range of tumour cell types, we have chosen to focus on breast carcinoma, in particular hormone insensitive, cells. This was partly because some oestrogen receptor negative breast cancer cells have constitutively activated NFkappaB which enhances sigma ligand-induced death. RT-PCR analysis has revealed that breast carcinoma cell lines express the sigma-1 receptor subtype; radioligand binding assays have confirmed the existence of high affinity sigma ligand binding sites on breast carcinoma cell membranes. We are extending these analyses to excised human breast tumour specimens.

Transient overexpression of the sigma-1 receptor, cloned from human breast cancer cells, markedly protects transformed human epithelial cells from p53-and Bax-induced apoptosis. *In vivo* experiments are underway to address anti-tumour properties of sigma ligands. These data indicate an anti-apoptotic function of the sigma-1 receptor, on which tumour cells have become reliant. This may provide a new therapeutic opportunity, particularly applicable to tumours which are resistant to existing treatments.

### P116

WITHDRAWN

### P117 5 ASA UPREGULATES 5FU INDUCED BAX EXPRESSION IN COLON CANCER CELLS IN VITRO

*D Adeyemo, S Toffa, M Lowdell¶ and M Winslet, Academic Departments of Surgery and Haematology¶, Royal Free and UCL Hospital Pond Lane, London NW3 2QG, UK

**Background** 5FU action in advanced colon cancer is by induction of apoptosis. Apoptosis in mammalian cells is regulated by the Bcl-2 class of proteins comprising the anti-apoptotic Bcl-2, Bcl-x, Mc1 and the pro-apoptotic Bax, Bad, Mad, Bak etc (1). NSAIDs induce apoptosis and reduce proliferation in colon cancer cell lines, lower incidence of colorectal cancer in humans while cause colon polyp regression (2). Epidemiological studies also found lower incidence of colon cancer in inflammatory bowel disease patients managed on 5 aminosalicylic acid or sulphasalazine than in the population at large(3).

**Aim** To evaluate additive effects if any, of 5 Aminosalicylic acid at colon concentrations on 5FU induced upregulation of Bax expression in colon cancer cells in vitro.

**Materials and Methods** Colo 201 and Colo 205 cells were cultured for 72 hours with 5 ASA, 5FU alone or combination of 5FU and 5 ASA. The cells were trypsinised, fixed, permeabilised and stained with anti-Bax antibody. Phycoerythrin labelled secondary was added. $10^4$ cells were analysed by flow cytometry. Bax expression was recorded as the difference between test and isotype control fluorescent values. Upregulation of Bax expression was calculated as test: control ratio. Concentrations used: 5 ASA 7 mM, 5FU-0.1 μM for Colo 201 and 1 μM for Colo 205.

**Results**

| | 5FU only | 5 ASA | 5 ASA + 5FU |
|---|---|---|---|
| Colo 201 | 1.1 (0.1) | 1.1 (0.3) | 3.3 (0.1) |
| Colo 205 | 1.0 (0.1) | 0.9 (0.03) | 4.1 (0.7) |

Bax fluorescence ratios n = 3 sd – ().

**Conclusion** Apoptosis is associated with significant upregulation of Bax expression. The above findings suggest additive pro-apoptotic effects of 5 ASA and 5FU in vitro. 5 ASA absorption is minimal, therefore concentrations employed in this study far exceed serum levels. Well tolerated and better absorbed NSAIDs may however augment 5FU chemotherapy in the systemic management of advanced colon cancer.

1 Oltvai NZ, Milliman CL, Korsmeyer SJ (1993) *Cell* **74:** 609
2 Goldgerg Y, Nassif I, Pittas A, et al (1996) *Oncogene* **12:** 893
3 Moody G, Probert J, Kay H, Mayberry J (1996) *Euro. Jo. Of Gastroenter and Hepatol* **8**(12): 1179

### P118 ANTIOXIDANTS AUGMENT 5FU UPREGULATION OF BAX EXPRESSION IN COLON CANCER CELLS IN VITRO

*D Adeyemo, S Toffa, M Lowdell[1] and M Winslet, Academic Departments of Surgery and Haematology[1], Royal Free and UCL Hospital, Pond Street, London NW3 2QG, UK

**Background** Chemotherapeutic action of 5FU in advanced colon cancer is by induction of apoptosis. Alteration of cell thymidylate metabolism by Folinic acid improves its poor response rate (1).

Apoptosis in mammalian cells is regulated by the Bcl-2 class of proteins comprising the anti-apoptotic Bcl-2, Bcl-x, Mc1 and the pro-apoptotic Bax, Bad, Mad, Bak etc (3). Oxidative DNA damage in cells is implicated in oncogenesis and continuing mutation possibly leading to drug resistance. Reactive oxygen species in submicromolar concentrations also act as signal transducers in cancer cells (2).

**Aim** To evaluate the effect of oxidant quenching on 5FU induced upregulation of Bax expression in colon cancer cells in vitro.

**Materials and Methods** Colo 201 and Colo 205 cells were cultured for 72 hours with N Acetyl Cysteine, Vitamin E or 5FU alone or a combination of 5FU and one of the antioxidants. The cells were trypsinised, fixed, permeabilised and stained with anti-Bax antibody. Phycoerythrin labelled secondary was added. $10^4$ cells were analysed by flow cytometry. Bax expression was recorded as the difference between test and isotype control fluorescent values. Upregulation of Bax expression was calculated as test: control ratio. Concentrations used: 25 mM NAC, 5 mM Vit. E, 5FU –0.1 μM for Colo 201 and 1 μM for Colo 205.

**Results**

| | 5FU only | NAC | Vit. E | NAC + 5FU | Vit. E + 5FU |
|---|---|---|---|---|---|
| Colo 201 | 1.1 (0.1) | 0.5 (0.1) | 0.9 (0.2) | 2.7 (0.4) | 3.0 (0.4) |
| Colo 205 | 1.1 (0.1) | 1.1 (0.5) | 0.9 (0.3) | 2.7 (0.4) | 3.1 (0.6) |

Bax fluorescence ratios n=4 sd –().

**Conclusion** Apoptosis is associated with significant upregulation of Bax expression. The above findings suggest that redox status alteration increase colon cancer cell susceptibility to 5FU in vitro. This is consistent with published reports. Oxidant quenching may act by abrogating continuing mutation and interfering with intracellular signal transduction. If reproduced in vivo, with tolerated doses of antioxidants, improved response rates and/or reduced toxicity or perhaps both may be achieved with antioxidant/5FU combination.

1 Colucci G, Gebbia V et al (1999) *Cancer* **85**(3): 535
2 Factor VM, Kiss A et al (1998) *The Jo Of Biol Chem* **273**(25): 15846
3 Oltvai NZ, Milliman CL, Korsmeyer SJ (1993) *Cell* **74:** 609

### P119 bFGF-INDUCED CELL DEATH OF EWING'S SARCOMA IS ASSOCIATED WITH ARREST OF CELLS AT THE $G_1$ CHECKPOINT AND ACTIVATION OF PRB1

*G Westwood and SA Burchill, Candlelighter's Children's Cancer Research Laboratory, ICRF Cancer Medicine Research Unit, St. James's University Hospital, Leeds LS9 7TF, UK

bFGF has been implicated in a multitude of physiological and pathological processes including limb development, angiogenesis, wound healing and tumour growth. It has been shown to be a potent mitogen for oligodendrocytes, schwann cells and astrocytes, and plays an important role in early neural crest development where it triggers mitosis and later commits cells towards a neural lineage. However, we have recently shown that bFGF decreases cell growth induced by cell death in the neural crest derived Ewing's sarcomas (1). The aim of this study was to investigate the effects of bFGF on the cyclin complexes that exert positive and negative control on the progression of cells through the $G_1$ phase of cell cycle in Ewing's sarcoma cell lines.

To characterise the mechanism of bFGF-induced cell death, cultures were synchronised and changes in cell cycle profile were identified by labelling with propidium iodide and FACs analysis. Two Ewing's sarcoma cell lines in which bFGF induced cell death (TC-32 and TTC-466), and one that did not respond to bFGF (A673) were studied. Regulation of cyclin complexes at the protein level was characterised by Western blotting and densitometry using mouse monoclonal antibodies against p53, p21, p27, cyclin D1, cyclin E, cyclin A, pRb1 and PCNA. In the TC-32 and TTC-466 cell lines, a significant decrease in the proportion of cells found in the $G_1$ phase of cell cycle was observed after treatment with bFGF (10 ng/ml) for 48 h ($p<0.05$) or 72 h ($p<0.001$). This was characterised by the upregulation of p21, p27 and p53, downregulation of PCNA and cyclin A and dephosphorylation of pRb1. Cyclin D1 and cyclin E levels were unchanged. bFGF (10 ng/ml) induced cell death was not observed in the Ewing's sarcoma cell line A673 (1) and had no effect on the proportion of these cells in the $G_1$ phase of cell cycle, phosphorylation state of pRb1 or the regulation of PCNA.

In summary, proteins responsible for the control of $G_1$ arrest are implicated in bFGF-induced cell death in Ewing's sarcoma, resulting in activation (dephosphorylation) of pRb1. We are currently investigating the expression of cell death associated proteins and their relationship with cell cycle regulation.

1 Sturla, L et al (2000) Cancer Research. Submitted.

### P120 THE DELAYED ACTION OF THE SPINDLE CHECKPOINT IN P53 MUTATED LYMPHOID TUMOUR CELLS

A Ivanov[1], MS Cragg[2], TM Illidge[2,3], Je Erenpreisa[1], [1]Laboratory of Tumour Cell Biology, [2]Biomedicine Centre, University of Latvia, Riga; [2]Tenovus Research Laboratory; [3]CRC Department of Oncology, Cancer Sciences Division, Southampton General Hospital, Southampton University SO16 6YD, UK

After DNA damage p53 mutated tumours are associated with abrogation of the G1/S checkpoint, delay and further adaptation of the G2 checkpoint and subsequent aberrant mitoses. Little is currently known regarding the fate of these mitoses. Given the well-documented differences in DNA damage sensitivity of wild-type (wt) and mutant (mut) p53 cells we therefore investigated these events in a variety of human lymphoblastoid cell lines with differing p53 status after irradiation with doses of 5, 10 and 15 Gy.

After 5 Gy, TK6 (p53 wt) cells undergo partial arrest in metaphase at the first cycle and then restitute normal mitotic events from day 5 onwards. After 10 and 15 Gy however, the majority of cells fail to enter mitosis and subsequently die. In contrast, the behaviour of p53 mut (W1-L2-NS) cells is strikingly different. Firstly, after 5 Gy, cells exhibit delayed recovery from day 7 onwards. Secondly at all 3 doses of irradiation p53 must cells undergo 2–3 cycles of aberrant mitoses followed by full arrest in metaphase, starting from day 4. This arrest is prolonged and dose dependent. Interestingly, cells arrested in metaphase have three potential fates. These include; (1) release into anaphase, (2) apoptosis, and (3) restitution into polyploid interphase. The first pathway occurs most frequently for p53 wt cells after 5 Gy, whilst the third occurs only in p53 mutated cells. Furthermore, the accumulation of cells arrested in metaphase appears directly proportional to the time spent in metaphasic arrest and is inversely proportional to the rate of the diploid cell line recovery, as well as the length of time spent arrested in G2. Such mitotic events may therefore be used as an indirect indicator of the DNA damage repair efficiency at interphase checkpoints. Our data indicate for the first time that the spindle checkpoint is delayed in p53 mutated cells. We believe this delay is due to failure of the sensing machinery such that the checkpoint is only engaged when the chromosome aberrations accumulated through several cell cycles are finally sufficient to prevent the p53 mut cells from propagating the grossly damaged genome. However, it would also appear that this control can be overridden by mitosis restitution.

### P121 CAFFEINE TRIGGERS EXIT FROM M PHASE INTO ENDOREDUPLICATION CYCLE AND APOPTOSIS AFTER IRRADIATION IN P53 MUTATED BUT NOT P53 WILD TYPE CELLS

A Yiakouvaki[1], MS Cragg[2], Je Erenpreisa[3], TM Illidge[1,2] [1]CRC, Department of Oncology, Cancer Sciences Division, Southampton General Hospital, Southampton University S016 6YD UK; [2]Tenovus Research Laboratory; [3]Laboratory of Tumour Cell Biology, Biomedicine Centre, University of Latvia, Riga

Caffeine is known to potentiate the cytotoxic effects of DNA damaging agents and increase the sensitivity of p53-deficient cells to irradiation (IRR). We investigated the cell cycle effects after IRR in the presence and absence of caffeine in haematological cell lines with p53 mutant and p53 wild type status. p53 mutant lines displayed an impaired G1 checkpoint and dose dependent G2/M arrest following IRR. This G2 delay was abrogated in the presence of 2.5 mM caffeine by inactivation of $p34^{Cdc2}$ kinase leading to cycle progression. Interestingly the decrease in $p34^{Cdc2}$ activity correlated with an increase of cells in G1 and a highly significant (3-fold) increase in the number of polyploid cells. This dramatic increase in polyploid cells initiated by Caffeine suggests a switch from mitotic to endoreduplication cycle in these cells. The subsequent breakdown of these polyploid giant cells represents the major source of the highly significant (P<0.01) increase in apoptosis seen within the first 144 hours for p53 mut cells treated with IRR in presence of 2.5 mM Caffeine but not seen with p53 wt cells. We have subsequently followed the fate of these polyploid cells with separation experiments and strongly believe that they represent an important source of mitotically competent cells that bring about the recovery of the stem-line. Therefore these results indicate that the cell cycle regulatory activities of caffeine are distinct from the cytocidal activity reported and that p53 mutated cells are capable of switching from mitotic to endoreduplication cycle and back again to mitotic cycle. Overall this data suggest that this tumour specific switch may be a therapeutically exploited strategy for overcoming the resistance of p53-deficient cancers to anti-cancer treatments.

### P122 HYPOXIC ACTIVATION OF NFκB IN MCF-7 AND T47D CELLS DOES NOT INVOLVE DEGRADATION OF 1κBA

CA Parker*, CE Lewis & JA Royds, Division of Oncology and Cellular Pathology, University of Sheffield Medical School, Beech Hill Road, Sheffield S10 2RX, UK

Hypoxia which occurs as a consequence of disorganised vasculature within solid tumours, has been implicated in tumour progression and resistance to treatment. The transcription factor NFκB responds to a diverse range of stress signals including hypoxia and has a role in cell survival, protecting cells against apoptosis. 1κBα regulates NFκB by sequestering it in the cytoplasm and Ubiquitin-mediated degradation of 1κBα allows NFκB to translocate to the nucleus. Activation of NFκB by hypoxia may be one mechanism by which tumour cells can survive low oxygen conditions.

Nuclear and cytoplasmic extracts from T47D and MCF-7 breast carcinoma cell lines cultured under normoxic and hypoxic (0.5%$O_2$) conditions were assayed by western blotting for RelA (a NFκB family member) and 1κBα protein levels.

RelA and 1κBα were confined to the cytoplasm in both cell lines under normoxic conditions, but within 30 mins of hypoxia RelA appeared in the nucleus of T47D cells and remained until after 24 hours. However, in MCF-7 cells nuclear expression of RelA did not occur until 2 hours of hypoxia and only continued until 12 hours. 1κBα was consistently present in the cytoplasm in both cell lines, but approximately 1 hour following RelA expression in the nucleus 1κBα also appeared in the nucleus and persisted until RelA was absent from the nuclear fraction.

Hypoxia causes nuclear translocation of NFκB and T47D cells exhibit a more rapid and prolonged activation than that seen in MCF-7 cells. In contrast to reports for other stimuli, hypoxia does not result in significant degradation of 1κBα. This could suggest an alternative mechanism for 1κBα regulation of NFκB. The presence of 1κBα in the nucleus following RelA translocation may indicate that 1κBα may have another level of regulation by directly attenuating the NFκB response.

This work is supported by Yorkshire Cancer Research.

### P123 NUCLEAR FACTOR KAPPA B (NF-κB) AND PANCREATIC CANCER TREATMENT

Y Kyung Kang, JP Neoptolemos and E Costello, Department of Surgery, University of Liverpool, UK

Pancreatic ductal adenocarcinoma shows especially poor responses to cytotoxic therapies; the mortality at one year is 90% and nearly 100% at five years. A common clinical problem in human cancer treatment is resistance of tumours to chemotherapy. Apoptosis seems to be a principle means whereby anti-cancer therapies such as chemotherapy and radiation exert their responses. Cell death induced by chemotherapy and radiation requires the activation of the caspase cascade, leading to proteolytic cleavage of a variety of important proteins and ultimately cleavage of cellular DNA. The induction, in some cancer cell lines, of the transcription factor NFκB however, in response to chemotherapy and radiation potently suppresses apoptosis. NFκB has been reported to be constitutively activated in pancreatic cancer tissue but not in normal pancreas.

**Aim** To establish whether NFκB is further induced in response to cytotoxic drugs.

**Methods** Electrophoretic mobility shift assays were carried out with nuclear extracts from six pancreatic cancer cell lines, Suit-2, MiaPaCa, BxPc3, Pancl, CFPAC1 and AsPc1 treated with five different cytotoxic agents, TNFα, Gemcitabine, 5-Fluorouracil, Daunorubicin and CPT11.

**Results** NFκB activation was observed in all cell lines in response to treatment with certain cytotoxic drugs. The level of NFκB induction varied from cell line to cell line and was more pronounced with some cytotoxic drugs than with others in a cell-line dependent manner.

**Conclusions** NFκB activation occurs in pancreatic cancer cell lines in response to treatment with cytotoxic drugs. We are currently investigating whether the induction of NFκB following cytotoxic drug treatment affects cell viability, and if so whether inhibiting its activity, through a gene therapy approach, can enhance the efficiency with which chemotherapeutic compounds kill pancreatic cancer cells.

### P124 EXPRESSION OF CYCLIN D1, CYCLIN E AND $P27^{KIP1}$ IN SCHISTOSOMAL VERSUS NONSCHISTOSOMAL BLADDER CANCER

AA Khan[1], KS Chaudhary[1], PD Abel[2], AM Shoma[3], M El Baz[3], GWH Stamp[1] and E-N Lalani[1], Depts of [1]Histopathology and [2]Surgery, Imperial College School of Medicine, Hammersmith Campus, London W12 0NN and [3]Dept of Pathology; Mansoura University, Egypt

Schistosomal bladder cancer is considered clinically more aggressive than its nonschistosomal counterpart but the molecular mechanisms underlying this difference are not clear. Altered expression of the GI-S checkpoint regulators, cyclin D1, cyclin E and $p27^{Kipl}$ is frequently observed in many human tumours, including bladder cancer. We compared the immunohistochemical expression of these important cell cycle regulators and the proliferation marker, Ki-67 in 29 invasive non-schistosomal transitional cell carcinomas (TCC) with 20 invasive schistosomal bladder carcinomas, including 10 squamous (SCC) and 10 transitional cell subtypes. Well-characterized monoclonal antibodies were used. For each marker, the percentage of immunoreactive tumour cell nuclei was determined and expressed as an index. Mean staining indices were used to compare subgroups. Overall, the Ki-67 proliferative index was significantly higher in the schistosomal group when compared with the nonschistosomal group ($P$ = 0.001). However, cyclin D1 and E expression was significantly lower in the schistosomal group ($P$ = 0.02 and 0.01, respectively). Within the schistosomal group, TCCs displayed significantly higher expression of cyclin E ($P$ = 0.006) when compared with SCCs. In contrast, the Ki-67 index was higher for the SCC subgroup, although this difference did not reach statistical significance. Schistosomal TCCs and SCCs were comparable with respect to tumour grade, pathologic stage and expression of $p27^{Kipl}$ and cyclin D1. Furthermore, no significant differences were observed between nonschistosomal and schistosomal TCCs with regard to any of the markers studied.

These results suggest that cyclin E expression is significantly associated with TCC regardless of etiology. Moreover, the higher proliferative index of SCCs observed in this study is consistent with their more aggressive clinical behaviour.

### P125 A NOVEL MDM2 BINDING PROTEIN (MTBP) INDUCES A G1 ARREST THAT IS SUPPRESSED BY MDM2

Nikolina Vlatkovic, Conor Magee and *Mark T. Boyd, Dept of Surgery, University of Liverpool, 5th Floor UCD, Daulby Street, Liverpool L69 3GA, UK

The MDM2 protein, through its interaction with p53 plays an important role in the regulation of the $G_1$ checkpoint of the cell cycle. In addition to binding to and inhibiting the transcriptional activation function of the p53 protein, MDM2 binds, *inter alia*, to RB and E2F-1 and in so doing may promote progression of cells into S-phase. Mice transgenic for *MDM2* possess cells that have cell cycle regulation defects and develop an altered tumour profile independent of their *p53* status. MDM2 also blocks the growth inhibitory effects of TGF-β1 in a p53 independent manner. To identify the pathway/s that mediate these p53-independent (or downstream) effects of MDM2 we have sought to identify novel MDM2 interacting proteins.

We have identified a novel gene that encodes an MDM2 binding protein hence *M*dm *T*wo *B*inding *P*rotein (MTBP). We have confirmed this interaction in yeast, *in vitro* and in mammalian cells in culture. MTBP is a 104 kD protein with no extensive detectable homology to other database sequences from higher eukaryotes. FASTA analysis of the *Sacharromyces cerevisiae* genome database identified two proteins Boi1p and Boi2p that possess 20–21% amino acid identity to MTBP in a 400 amino acid stretch. These molecules are both part of a G-protein dependent signal transduction pathway that regulates bud formation. Ectopic expression of Boi1p and Boi2p leads to growth arrest in yeast. Coincidentally, ectopic expression of MTBP leads to growth arrest in a variety of human tumour cell lines to a similar degree to that induced by p53. Flow cytometric analysis demonstrated that the growth inhibition induced by MTBP is a result of an induction of G1 arrest. We therefore examined whether MDM2 could abrogate this growth arrest much as it does for p53 and found that this is indeed the case. We are currently investigating the normal function of MTBP using anti-MTBP sera. We are also investigating the mechanism by which MTBP induces growth arrest. These data will also be presented. Our results suggest the existence of an additional growth control pathway that may be regulated, at least in part, by MDM2.

### P126 IN VIVO VARIATION OF P53 EXPRESSION IN RESPONSE TO SERUM OESTROGEN CONCENTRATIONS IN MCF7 BREAST CANCER XENOGRAFTS

D Ziyaie[1], D Dornan[2], TR Hupp[2], NM Kernohan[2], AM Thompson[1], [1]Dept. of Surgery & Molecular Oncology, [2]Dept of Molecular & Cellular Pathology, University of Dundee DD1 9SY, UK

Functional inactivation of otherwise normal, wild-type p53, whereby the p53 tumour suppressor activities could be inactivated by mechanisms other than p53 gene mutation, has been described in many forms of human cancer. Models describing mechanisms of p53 activation have demonstrated that, in response to cellular damage, p53 can (a) become activated and stabilised, (b) be activated in the absence of protein stabilisation or (c) despite being activated, exhibit reduced p53 protein levels.

We have investigated the in vivo variation of p53 expression in response to serum oestrogen concentrations in a xenograft model (ref) established from an MCF-7 breast cancer cell line, which contains wild-type p53. Western blot analysis results have demonstrated a distinctive pattern of p53 immuno-reaction in response to alterations of serum oestrogen concentrations. We observed that p53 protein levels were *reduced* in response to increasing levels of serum oestrogen, during which tumour kinetics in terms of growth rate reached maximum; conversely, p53 levels *rose* concomitant with the lowering levels of oestrogen, coinciding with static or minimal tumour growth.

This inverse correlation between p53 protein expression and serum oestrogen concentration suggests that oestrogen may either act as an inhibitor for p53 activation by promoting its degradation, or may be a p53 activator in a dose-dependent manner, despite the initial apparent lower levels of the p53 protein.

These data highlight (a) the importance of serum oestrogen concentration as an adjunct to p53 expression for in vivo studies and (b) the possibility of a dual role of action for oestrogen in the pathogenesis of breast cancers, acting not only as a true mitogen but also as a tumour behaviour-modifying agent.

AM Thompson, CM Steel, ME Foster, D Kerr, D Paterson, D Deane, RA Hawkins, DC Carter & HJ Evans (1990) Gene expression in oestrogen-dependent human breast cancer xenograft tumours. *Br J Cancer* **62:** 78–80

### P127 THE STUDY OF DNA-INDUCED P53 CLEAVAGE PRODUCT WITH INDUCIBLE SYSTEM *IN VIVO*

Ming Jiang* and Jo Milner, p53 Research Group, Yorkshire Cancer Research, Department of Biology, University of York, York YO10 5DD, UK

The p53 tumour suppressor gene plays a central role in the regulation of cell growth and is inactivated by mutation in more than 50% of human cancers. The p53 protein is activated in response to DNA damage by recognising and binding sides of DNA damage, determining the cells towards arresting or apoptosis. The previous work has found that the different damaged DNAs could induce different cleavages of p53 *in vitro*, but they all generate a key product of 35 kDa peptide that encompasses the p53 DNA-binding domain (1,2). The p35 cleavage product has protease activity and is able to cleave the N-terminus of full-length p53 to generate p50 (ΔN23) (2).

In order to investigate the functions of p53 cleavage products, we are using two inducible systems: TET-ON and LAC-ON. Both TET-ON and LAC-ON expression of p35 were shown to be dose dependent and reversible. Importantly the p35 core domain adopted the 'wild type' conformation for p53 ($246^+/1620^+/240$) and was both cytoplasmic and nuclear. Evidence that p35 can induce G1 arrest and trigger cell apoptosis after DNA damage in p53-null fibroblasts will be presented. In addition, we observed cleavage of full-length endogenous p53 in T3T3 cells expressing p35, consistent with the amino peptidase activity previously observed *in vitro* (see above). Our results indicate that the p35 cleavage product of p53 may function during the p53 responses to DNA damage *in vivo*.

1. Molinari M, Okorokov A and Milner J (1996) Interaction with damaged DNA induces selective proteolytic cleavage of p53 to yield 40 kDa and 35 kDa fragments competent for sequence-specific DNA binding. *Oncogene* **13:** 2077–2086
2. Okorokov A, Ponchel F and Milner J (1997) Induced N- and C-terminal cleavage of p53: a core fragment of p53, generated by interaction with damaged DNA, promotes cleavage of the N-terminus of full-length p53, whereas ssDNA induces C-terminal cleavage of p53. *EMBO J* **16**(19): 6008–6017

### P128 EVALUATION OF TYROSINASE STANDARD VS. LIGHTCYCLER™ RT-PCR IN MELANOMA PATIENTS

[1]Konstantin Stoitchkov, [2]Thierry Le Bricon, [1]National Center of Oncology. Sofia, Bulgaria, [2]Laboratory of Biochemistry A, Saint-Louis Hospital, Paris, France

Malignant melanoma (MM) is the most aggressive skin cancer. There is still need of reliable laboratory test for early detection of metastasis and residual disease to monitor therapy or to contribute to individual and overall prognosis.

The development of RT-PCR has made the search of circulating melanoma cells in peripheral blood possible. Tyrosinase seems to be the most appropriate target by now. In a previous study, we found RT-PCR positive in only 1 of 10 stage III and 3 of 20 stage IV melanoma patients. In the present study we investigated the influence of different RT-PCR techniques on tyrosinase positivity in melanoma patients with an emphasis on cDNA quality control.

Ten healthy subjects, six nonmelanoma skin cancer patients and 53 melanoma patients from Sofia, Bulgaria entered the study. Longitudinal studies were performed in 47 melanoma patients. A total number of 143 blood samples were analyzed. All samples after Ficoll treatment were carried in Paris. France for acid guanidinium thiocyanate-phenol-chloroform RNA extraction and reverse transcription. PCR were performed in 2 different laboratories using different protocols: 1/ in Paris on an HYBAID™ PCR machine and 2/ in Berlin. Germany* on a LightCycler™ instrument (Roche). Total cellular RNA was reverse transcribed with random hexamers and HTYR2 antisense primers for stage III and IV patients. cDNA were stored at – 20°C before PCR. In both laboratories, blood mRNA integrity was checked by PCR with primers for human β-2-microglobulin. PCR analysis of tyrosine transcripts was performed using nested PCR as previously described by Smith et al. PCR kinetics observed on the LightCycler system for β-2-microglobulin of tyrosinase RT-PCR positive samples paralleled that of the positive control. Only 2 stage IV patients out of 11 were found tyrosinase positive by standard PCR and by the LightCycler™ system (one reverse transcribed with random hexamers, one with specific antisense primer).

Cancer cell RNA from peripheral blood is generally obtained after gradient centrifugation or whole blood processing. We obtained excellent RNA quality after storage of Ficoll-treated samples at –80°C and transportation of samples in dry ice. While commercial kits work well on cultured cell lines, we recommend the use of the Chomezynski method for RNA extraction of clinical samples. The use of random oligo nuclcotide sequences as primers to generate cDNA or gene-specific sequences could lead of detecting or loosing positive signals, so it might be appropriate to do both. The LightCycler™ system allows the evaluation of cDNA quality by following kinetic aspects of housekeeping gene PCR. Poor cDNA quality might be considered as one of the possible reasons for low positivity rate of tyrosinase mRNA.

RT-PCR in peripheral blood as staging test for melanoma patients must be interpreted cautiously. A consensus for standardization of the RT-PCR technique for tyrosinase mRNA in peripheral blood is needed to make possible the comparison of results. It should include reliable quality control assays for each step of the technique. The LightCycler™ system, easy to run might be useful to monitor cDNA quality and further to quantify amplified PCR products.

### P129 ADJUVANT INTERFERON α IN HIGH RISK RESECTED MELANOMA APPEARS TO PROLONG DISEASE-FREE SURVIVAL

*DA Cameron[1], MC Cornbleet[1], RM Mackie[2], JAA Hunter[3], B Hancock[4], M Gore[5], JF Smyth[1] on behalf of the Scottish Melanoma Group, [1]Western General Hospital & [3]Royal Infirmary, Edinburgh, [2]Western Infirmary Glasgow, [4]Weston Park Hospital Sheffield & [5]Royal Marsden

Recent data suggest that for patients with stage II malignant melanoma, low dose interferon α may result in a disease-free survival advantage[1,2]. In March 1989, the Scottish melanoma group began a randomised study of α-Interferon for patients with surgically treated primary malignant melanoma who were at high risk of subsequent relapse. Patients were eligible if they had had a fully resected primary melanoma > 3 mm Breslow (stage II), or with regional node involvement (stage III). By August 1993, when the study was closed, 49 patients had been randomised to receive Interferon α 2b, 3MU (Schering-Plough) three times a week for six months, and 46 patients to observation only. The median age was 47 [range 16–79], with 35 (37%) patients having stage II disease and 60 (63%) stage III. The two arms were well balanced for age and stage. Patients were monitored monthly whilst on therapy, and no serious toxicity was seen. At a minimum follow-up of 6 years, 70 (73%) of the patients had relapsed (29% stage II, 71% stage III). Median disease-free survival was extended from 9 months to 22 months for interferon treated patients, and median overall survival from 27 to 38 months. These differences were not statistically significant, but are within the range reported from larger studies using higher doses and/or longer duration of therapy.

Since the lack of a statistically significant survival advantage may be due to the low number of patients recruited, these data suggest that even 6 months' adjuvant low-dose interferon may be beneficial in patients with high risk melanoma. Confirmation of the possible survival advantage of this regimen can only come from larger studies such as the AIM High trial, to which recruitment should be encouraged.

1 Grob JJ, Dreno B, de la Salmonière P, *et al* (1998) Randomised trial of interferon α-2a as adjuvant therapy in resected primary melanoma thicker than 1.5 mm without clinically detectable node metastases. *Lancet* **351:** 1905–1910

2 Pehamberger H, Soyer HP, Steiner A, *et al* (1998) Adjuvant Interferon Alfa-2a treatment in resected primary stage II cutaneous melanoma. *J Clin Oncol* **16:** 1425–1429

### P130 ADHESION-DEPENDENT CELL CYCLE CONTROL OF MELANOCYTE-DERIVED CELL LINES

R Gilchrist*, D Jenkinson, RS Camplejohn, IR Hart, JF Marshall, Richard Dimbleby Department of Cancer Research/ICRF Laboratory, Rayne Institute, St Thomas' Hospital, Lambeth Palace Road, London SE1 7EH, UK

We characterised a panel of C57/B1 mouse melanocyte-derived cell lines to study the control mechanisms involved in adhesion-dependent and -independent growth (Br. J. Cancer, **80**(2), 25, 1999). Using BrdUrd labelling techniques we report that, in suspension, Melan-a (immortal melanocyte) cells arrest immediately in $G_1$ whereas MT1.2 (polyoma middle T-transfected melanocytes), LTR Ras 3 (Ha-ras transfected melanocytes) and B16F10 cells (melanoma) retain proliferative capacity of varying degrees over a period of 24 hrs. $G_1$-phase cell cycle control proteins have been examined during these adhesion-mediated changes. Western blotting revealed that p16 is absent in all four cell lines (p16 is frequently deleted in melanoma). The pattern of Rb expression and phosphorylation showed the expected correlation with adhesion-dependent proliferation. In Melan-a cells, the unphosphorylated, hypophosphorylated and hyperphosphorylated Rb bands are detectable after 24 hrs monolayer growth. However, when cultured for 24 hrs in suspension, the hyperphosphorylated band is absent leaving only hypophosphorylated and, to a lesser extent, unphosphorylated Rb (60%:40%). No unphosphorylated Rb was detected in the remaining cell lines. In fact, after 24 hrs monolayer growth, only hyperphosphorylated Rb was detected. Following 24 hrs suspension culture, the MT1.2 cells showed a reduction in hyperphosphorylated Rb with an increase in hypophosphorylated Rb (23%:77%) whereas the LTR Ras 3 and B16F10 cells expressed equal amounts of hyperphosphorylated and phosphorylated Rb. Melan-a cells appear to have a much reduced expression of total Rb compared with the other three cell lines. P13-kinase activity has been implicated in adhesion-independent cell survival and in the progression of growth factor-deprived cells through the $G_1$ phase of the cell cycle. We therefore analysed the role of P13-K in adhesion-independent proliferation. Monolayer B16F10 and MT1.2 cells treated with 10 uM LY294002 for 16 hrs had reduced the S-phase fraction to 86% and 84% of the control value respectively, whereas the S-phase fraction of non-adherent cells was reduced to 41% and 43% respectively. These data correlated with a reduction in the hyperphosphorylated form of Rb and an increase in hypophosphorylated Rb. Thus, adhesion-independent growth of transformed cells of melanocytic origin appears to require a P13K-activated pathway which may offer a means of targeting cancer cell proliferation.

### P131 THE ROLE OF MITOGEN ACTIVATED PROTEIN (MAP) KINASES IN THE α-MSH MEDIATED MELANOGENIC AND ANTI-PROLIFERATIVE RESPONSES OF B16 MELANOMA CELLS

KSM Smalley and T Eisen, Department of Oncology, University College London, 91 Riding House Street, London W1P 8BT, UK

α-melanocyte stimulating hormone (α-MSH) is a 13-amino acid hormone which exerts its physiological effects via interaction with a family of G protein coupled melanocortin receptors (named $MC_1$-$MC_5$) (see Wikberg, *Eur. J. Pharmacol.* 375 295). A number of recent studies have implicated both the p38 and p44/42 mitogen activated protein (MAP) kinases in cell differentiation. The aim of the current study was to investigate the role of these kinases in the α-MSH-induced melanogenic and proliferative responses of B16 murine melanoma cells.

Treatment of cells with α-MSH led to time-dependent phosphorylation of both p38 and p44/42 MAP kinases, albeit with differing kinetic profiles. Treatment of cells with inhibitors of MEK1 (PD-98059, 10 μM) or phosphoinositide 3 (PI 3)-kinase, (wortmannin, 1 μM) inhibited α-MSH induced phosphorylation of p44/42 MAP kinase, demonstrating that P13-kinase was activated upstream of MAP kinase. Incubation of B16 cells in the presence of α-MSH (10 nM for 72 hours) was shown to induce melanogenesis as measured by an extracellular melanin accumulation colormetric assay (Siegrist & Eberle, *Anal. Biochem.* **159** 191). Treatment of cells with inhibitors of both protein kinase C (PKC) (Ro 31–8220, 2 μM) and p38 MAP kinase (SB 203580, 10 μM) were shown to significantly ($P$<0.05) inhibit the extent of α-MSH-induced melanogenesis. Pre-treatment of cells with either wortmannin or PD-98059 did not significantly alter the extent of α-MSH induced melanogenesis. Application of either kinase inhibitor alone induced increases in melanin production of 21.6 ± 6.4% and 17.3 ± 4.7% of the response to α-MSH (10 nM), respectively. Incubation of B16 cells with increasing concentrations of α-MSH ($10^{-10}$M – $10^{-6}$M) led to concentration dependent inhibition of B16 cell growth (47.9 ± 4.1% of control values, $pIC_{50}$ 8.22 ± 0.08). Pre-treatment of cells with inhibitors of MEK1 or P1 3-kinase were shown to potentiate the anti-proliferative effect of α-MSH (inhibition by 74.3 ± 4.5% and 71.6 ± 1.1% of control values, respectively). In contrast, pre-treatment of cells with SB 203580 abolished the anti-proliferative effect of α-MSH. In addition, it was subsequently demonstrated that inhibition of either PKC or p38 MAP kinase in the absence of α-MSH had no significant effects upon cell growth.

In summary, it appears that the α-MSH induced melanogenic and anti-proliferative effects are transduced through activation of p38 MAP in B16 melanoma cells. It also seems apparent that the constitutive activity of p44/42 MAP kinase is responsible for B16 cell growth, as well as negatively regulating melanogenesis.

### P132 THE ROLE OF P13-KINASE IN MIGRATION OF MELANOCYTIC CELLS

D Jenkinson*†, C Sawyer‡, IR Hart†, B Vanhaesebroeck‡, JF Marshall†, † Richard Dimbleby/ICRF Laboratory, Rayne Institute, St. Thomas' Hospital, Lambeth Palace Road, London, ‡Ludwig Institute for Cancer Research, Royal Free and University College Medical School Branch, 91 Riding House Street, London, UK

PI3-kinase has been implicated in the control of cell migration, cell adhesion and proliferation in various cell types. Recent work has indicated that the different PI3-kinase p110 isoforms may control these different cellular functions. We have established a panel of syngeneic mouse melanocyte derived cell lines which include immortal melanocytes (Melan-a), transformed tumorigenic melanocytes (LTR Ras3 and MT1.2) and a metastatic melanoma (B16F10) and have examined the role that PI3-kinase plays in the migration of these cells.

Using time-lapse video microscopy, combined with image analysis, we have measured the rate of migration over 24 hours of these cells in 'scratch wound' assays. Data showed B16F10 and MT1.2 had similar migratory rates (17.3 μm/hr and 18.0 μm/hr respectively), nearly double that of the LTR Ras3 (9.5 μm/hr) or Melan-a (10.9 μm/hr). Treatment with 10 μM of the PI3-kinase specific inhibitor, LY294002, did not affect the rate of migration of either the LTR Ras3 (9.8 μm/hr) or Melan-a (9.8 μm/hr) cell lines, whereas it reduced significantly the migration of both the MT1.2 (11.8 μm/hr) and B16F10 (10.3 μm/hr) cell lines. Confirmatory haptotactic migration assays indicated that addition of LY294002 reduced the migration of B16F10 cells on collagen types I and IV, but not on laminin substrates. These results imply that the B16F10 and MT1.2 cells have both PI3-kinase dependent and PI3-kinase independent migratory mechanisms. PI3-kinase reportedly signals through a variety of molecules, including the protein kinases AKT/PKB and ERKs via Raf-1, as well as phosphoinositide (3,4,5)-triphosphate. The expression levels of AKT/PKB, ERK1 and ERK2, by Western blotting, was similar in all 4 cell lines. However, both AKT/PKB and ERK2 showed lower levels of phosphorylation in B16F10 cells (30.4% and 19.8% phosphorylated respectively) relative to MT1.2 cells (81.2% and 84.5% phosphorylated respectively). Taken together these data imply that the basis for PI3-kinase mediated migration in melanocytic cell lines is not a consequence of these downstream signalling events. Irrespective of this, these results show that PI3-kinase is responsible for up to 50% of melanocytic cell migration and thus inhibition of PI3-kinase migration promoting signalling pathways represents novel target(s) to decrease the migratory and therefore invasive capacity of melanomas.

### P133 MELANOMA INVASION THROUGH FIBRONECTIN AND RECONSTRUCTED HUMAN SKIN

R Molife*[1,2] P Eves[2], C Layton[2], P Lorigan[1] and S. MacNeil[2], [1]Dept of Oncology, Weston Park Hospital, Sheffield S10 2SJ, UK, [2]University Section of Medicine, Northern General Hospital, S5 7AU, UK

The escape of melanoma cells from the primary tumour through the basement membrane (BM) is thought to be the first step in metastasis of melanoma. The process of invasion is complex involving changes in cell/extracellular matrix (ECM) and cell/cell interactions. Our aim was to investigate these interactions with 2 different *in vitro* models of invasion. In the first model, cells invade through fibronectin (FN) coated filters under serum free conditions over 20 hours and the total percentage of cell invasion is calculated by harvesting all cells (1). This model attempts to reflect simple melanoma/ECM interactions. We have developed the second model to shed light on the more complex melanoma/cell/ECM interactions. Human skin is sterilised and made acellular to form de-epidermised dermis (DED) but retain BM antigens, or BM antigens can be removed by treatment with dispase (2). To the DED, dermal fibroblasts (F), epidermal keratinocytes (K) and A375SM or HBL human melanoma cells are added to BM+ or BM– dermis. The resultant composites are cultured at an air-liquid interface for 2–3 weeks prior to histology and immunohistochemistry.

| | FN: %invasion ± SEM (n) | Composites –K –F (n) | +K +F (n) |
|---|---|---|---|
| **HBL** | 54.1+4 (12) | – (15) | ++ (13) |
| **A375SM** | 5 ± 0.7 (16) | ++ (6) | +/– (6) |

–, no invasion; +/–, little invasion; ++, extensive invasion

We found the invasive behaviour of the 2 cell lines to differ significantly in the two models. HBL cells invaded well through FN but failed to invade through the DED in the absence of Ks and Fs. The presence of Ks and Fs facilitated HBL invasion. A375SM cells in contrast were poorly invasive through FN, but invaded well through DED on their own, with Ks and Fs reducing the extent of their invasion. Similar results were obtained with and without BM for both cell lines. HBL cells were S100 negative but HMB45 positive. A375SM cells were S100 positive and only occasionally HMB45 positive. The data suggests that different melanomas may invade by different mechanisms and also illustrates the importance of cell/cell and cell/ECM interactions. The composite model clearly illustrates that adjacent skin cells can influence melanoma metastasis and will be useful in exploring approaches to preventing cells initially breaching the BM and invading the dermis.

1 Richardson B, Price A, Wagner M et al (1999) *Br J Canc* **80:** (12) 2025
2 Eves P, Layton C, Hedley, S et al (2000) *Br J Dermatol* **142:** 1

### P134 INTEGRIN αVβ6 PROMOTES MIGRATION OF SQUAMOUS CELL CARCINOMA CELLS ON LOW CONCENTRATIONS OF FIBRONECTIN THROUGH A PKC DEPENDENT MECHANISM

MR Morgan*, E Dex[1], GJ Thomas[1], SA Whawell[1], PM Speight[1], IR Hart and JF Marshall, Richard Dimbleby Department of Cancer Research/ICRF Lab, St. Thomas' Hospital, London. [1]Eastman Dental Institute, University of London, UK

Upregulation of the fibronectin (Fn) receptor αvβ6 has been observed in squamous cell carcinoma (SCC). In order to establish whether this could promote, rather than merely be the consequence of, disease we have generated cell lines from the αv-negative SCC line, H357. This parental line expresses only one fibronectin receptor, α5β1. Using retroviral transfer of β6 cDNA we derived VB6, a cell line which expresses similar levels of α5β1, which has 10 fold more αvβ6 than the control line C1. In haptotactic migration assays the motility of C1 cells increased over coating concentrations of 1–10 µg/ml plasma fibronectin (1 µg/ml=17.7%, 5 µg/ml=19.1%, 10 µg/ml=25.7%) whereas migration of VB6 cells decreased over the same concentration range (1 µg/ml=34.9%, 5 µg/ml=26.2%, 10 µg/ml=17.1%). These data are consistent with those of Palecek et al (Nature (1997)385:537–540) who showed maximal migration requires intermediate adhesiveness, which varies dependent upon integrin expression, integrin-ligand binding affinity and substrate concentration.

C1 cells migrated to a greater extent on cellular fibronectin than they did on plasma fibronectin (cellular Fn 1 µg/ml=8.6%, plasma Fn 1 µg/ml=5.4%), whereas VB6 cells were more migratory on plasma fibronectin (cellular Fn 1 µg/ml=5.9%, plasma Fn 1 µg/ml=16.8%). The reasons for this observation, which was highly repeatable, are unclear, but may relate to differences in the proportion of splice variant fibronectin in cellular, versus plasma, fibronectin.

Assays were repeated in the presence of integrin-blocking antibodies and/or specific inhibitors of cell signalling. Thus, VB6 cell migration was reduced to 0% by 10 µg/ml of L230 (anti-αv) but only to 67.4% by 10 µg/ml of P4C10 (anti-β1); suggesting that VB6 migration on fibronectin is αvβ6-dependent principally. Migration on fibronectin of H357 cells (αv-negative) was inhibited completely by the inhibition of α5β1 (P1D6 anti-α5). These data suggest that α5β1 activity is reduced in VB6 cells, possibly due to increased αvβ6 expression, while reduction of migration to 7.8% in the presence of 100 nM Calphostin C (PKC inhibitor), suggest a PKC-dependent role for αvβ6 mediated migration in VB6 cells.

The ability of αvβ6 to promote SCC cell migration on low concentrations of fibronectin, possibly, could be involved in promoting a more invasive phenotype.

### P135 FACTORS MODULATING INTEGRIN β1 EXPRESSION ON THE BREAST CANCER CELL LINES MCF-7 AND MDA-MB-231

CJ Pogson*[1], CMW Chan[2], L-A Martin[2], GPH Gui[1], Departments of Academic Surgery[1] and Biochemistry[2], The Royal Marsden and Institute of Cancer Research, Fulham Road, London SW3 6JJ, UK

The metastatic cascade describes a sequence of cellular events that forms the pathological basis of tumour progression[1]. Integrins represent a large family of cell adhesion receptors that are widely expressed and play a leading role in this complex process. Integrin and growth factor dependent signals co-operate functionally in a variety of biological processes[2].

The objective of this study is to improve our understanding of the role of integrins in breast cancer metastasis by investigating how growth factors (Epidermal Growth Factor (EGF), Insulin-like Growth Factor-1 (IGF-1)), Oestradiol (E2) and Tamoxifen, affect integrin β1 expression. The breast cancer cell lines MCF-7 and MDA-MB-231 were used and integrin expression measured by Western Blot. On the MDA-MB-231 cell line, we have demonstrated that EGF and IGF-1 up-regulate integrin β1 expression by 2.4 and 2.8 fold respectively. Integrin β1 expression was down-regulated by tamoxifen, with a maximal effect at $10^{-6}$ M (34% reduction).

On the MCF-7 cell line E2 up-regulated integrin β1 expression at high concentrations, whereas down-regulation occurred at lower concentration, with maximal effects at $10^{-7}$ M (1.7 fold induction) and $10^{-11}$ M (29% reduction) respectively. Conversely, high concentrations of tamoxifen resulted in a 26% down-regulation of integrin β1 expression at $10^{-8}$ M. Low concentrations of tamoxifen had no or minimal effect on expression.

IGF-1 up-regulated integrin β1 expression on the MCF-7 cell line, with a peak effect at 60 minutes, at a concentration of 25 ng/ml (1.8 fold induction). We have demonstrated that EGF and IGF-1 up-regulate integrin β1 expression. Invading cancer cells interact with the basement membrane, endothelial cells and the extracellular matrix, a step requiring the up-regulation of adhesion receptors. Down-regulation of integrin β1 expression by tamoxifen may render cancer cells less adhesive and therefore less invasive.

Tamoxifen effect on the oestrogen receptor (ER) negative cell line MDA-MB-231, suggests signaling through an alternative pathway and may explain why some ER negative patients respond well to tamoxifen.

Identification of factors that regulate integrin expression may lead to the development of novel anti-metastatic agents.

1 Hart IR, Saini A (1992) *Lancet* **329:** 1453
2 Giancotti FG (1997) *Curr Opin Cell Biol* **9:** 691

### P136 CHARACTERISATION OF A HUMAN COLON CELL LINE POSSESSING ACQUIRED RESISTANCE TO FLAVOPIRIDOL

V Smith, P Workman and LR Kelland, CRC Centre Cancer Therapeutics, Institute of Cancer Research, Sutton, Surrey SM2 5NG, UK

Flavopiridol is a synthetic flavone closely related to a natural product found in the bark of the Indian tree Dysoxylum binefactarum. It acts as a broad spectrum cyclin-dependent kinase (cdk) inhibitor by competitively binding to the ATP binding cleft and is currently in clinical trials. Mindful that drug resistance may limit its clinical efficacy, the aim of this study was to establish a laboratory model of acquired resistance and elucidate the underlying mechanism(s). Initially, the growth inhibition properties of Flavopiridol were assessed in a panel of colorectal cell lines by the sulforhodamine B assay. The 96 hr $IC_{50}$ ranged from 45 nM in HCT116 to 170 nM in HT29 cells. Acquired Flavopiridol resistance was established in the HCT116 cell line by continuous exposure to increasing amounts of drug. This resulted (after 4 months) in the HCT116/8FP subline that is 8-fold resistant. Initial mechanistic studies have focused on the multi-drug resistance efflux proteins PgP and MRP. HCT116/8FP cells did not show crossresistance to chemotherapeutics that are known substrates for these proteins (e.g. doxorubicin and etoposide). Moreover, western blotting did not show any detectable PgP protein or MRP protein in either the parent or the resistant cell lines. Comparison of growth inhibition by paclitaxel or doxorubicin in the presence of non-toxic doses of PSC833, a PgP modulator, revealed no difference between the resistant and parent cell line. Using a cell line stably transfected with MRP (*versus* its vector control) no difference in growth inhibition after treatment with Flavopiridol was observed indicating that Flavopiridol is not a substrate for MRP. In order to exclude drug accumulation as a possible mechanism of resistance, uptake experiments were carried out which showed that uptake is increased in the resistant cell line when treated at equitoxic doses. To date, the most significant difference observed between the pair of lines is an increase in cyclin E expression in the resistant line. In addition no cross-resistance was observed to another cdk inhibitor, the aminopurine-based compound, roscovitine. In summary these investigations indicate that, in the HCT116 cells, neither MRP nor PgP play a role in the acquired resistance to Flavopiridol.

### P137 ROLE OF THE FAS/FASL PATHWAY IN HORMONE RESISTANT PROSTATE CANCER

S Ganta*[1,2], MJ Thompson2, GA Choudry12, C Bradley12, GM Flannigan1, MC Bibby2, Bradford Royal Infirmary1 & Clinical Oncology Unit2, University of Bradford, UK

**Introduction** Fas is a cell-surface receptor involved in inducing apoptosis. Alterations of the Fas gene can lead to the loss of its apoptotic function and contribute to the pathogenesis of some cancers. Castration induced apoptosis in murine prostate has been shown to involve the Fas apoptotic pathway. The aim of this study is to define the expression of the factors in the Fas apoptotic pathway and to detect Fas gene alterations in hormone resistant prostate cancers.

**Materials and methods** Components of the Fas/FasL apoptotic pathway were analysed in 75 untreated samples of prostate cancer by immunohistochemistry, they were categorised into complete and partial/non responders based on the PSA value 3 months following androgen withdrawal. Samples were also available for 20 patients following relapse into an androgen resistant state. The paired samples were both analysed using immunohistochemistry and for Fas gene alterations to examine the role in acquired androgen resistance. The malignant cells were selectively micro-dissected from H&E stained sections of the paired samples. Conformational changes in a 150 bp segment of exon 9 (which codes for the functionally critical death domain) were analysed by PCR-SSCP. The student T- test for equality of means, and Pearson's correlation were used to analyse the data.

**Results** Fas, FasL and FADD (a down-stream mediator) were identified in all the tumour tissues, while FLIP (an inhibitor) was noted in only 45% of the tumours. There was no difference in the expression of the factors in the Fas apoptotic pathway between the paired samples. The expression of the factors did not assist in identifying a subset of patients who responded poorly to treatment. The conformational analysis of exon 9 of the samples so far reviewed has shown no detectable conformational changes.

**Conclusions** This is the first report of the analysis for Fas gene alterations in prostate malignancy. Our data suggest that expression of the Fas pathway is unaltered following androgen resistance, and no conformational changes are noted in the exon critical to the induction of apoptosis, suggesting a functional Fas receptor which may be exploited therapeutically.

### P138 RESPONSE OF HUMAN DNA REPAIR DEFECTIVE CELL LINES TO DNA INTERSTRAND AGENTS

PH Clingen*, EE Denholm, IU De Silva, A Hockey and JA Hartley, CRC Drug DNA Interaction Research Group, Dept of Oncology, Royal Free and University College Medical School, 91 Riding House Street, London W1P 8BT, UK

DNA interstrand crosslinking (ICL) agents are integral components of chemotherapeutic protocols utilised in the treatment of many human cancers. Mechlorethamine and melphalan are major groove binding alkylating agents capable of forming both monoadducts and ICLs. The importance of ICLs for cytotoxicity is reflected by comparison with their monofunctional derivatives which produce indistinguishable patterns of monoalkylation but which do not form ICLs. By using growth inhibition assays (MTT) bifunctional mechlorethamine and melphalan were approximately 500 and 40 fold more toxic than their respective monofunctional counterparts in normal human fibroblasts (1BR.3). The exact mechanism by which ICLs are processed in human cells is currently unknown. Data from model yeast and rodent DNA repair defective cells indicate that nucleotide excision repair (NER), especially the XPF-ERCC1 exinuclease activity and recombination systems are necessary for their repair. In contrast, comparison of cytotoxicity in a panel of human DNA repair defective fibroblasts including cells from NER defective xeroderma pigmentosum complementation groups A (XP1TUF), C (XP14BR), F (XP24BR) and G (XP125LO) and radiosensitive ataxia-telangiectasia cell lines (AT1BR, 290BR) and a cell line with a defect in DNA ligase IV (180BR) show no marked sensitivity to mechlorethamine or melphalan. All cell lines so far examined, demonstrated normal levels of unhooking of ICLs as measured using a modified 'comet assay'. We found no obvious overlap between DNA repair pathways for processing ICLs in model lower eukaryotic cell systems and in the human cell lines treated with mechlorethamine or melphalan. The hypothesis is that human Fanconi anemia gene products compensate for the repair defects in the panel of cells investigated. Fanconi anemia (FA) is a recessively transmitted genetic disease characterised by marked sensitivity to crosslinking agents. Current investigations aim to identify the role of FA proteins in the repair of ICLS.

### P139 DEFINING THE PATHWAYS INVOLVED IN THE INITIATION AND RESOLUTION STEPS OF DNA INTERSTRAND CROSSLINK REPAIR

PJ McHugh* and JA Hartley, CRC Drug-DNA Interactions Research Group, Dept Oncology, Royal Free and University College Medical School, UCL, 91 Riding House Street, London W1P 8BT, UK

We have previously reported that DNA double-strand breaks (DSBs) are a key intermediate generated during the repair of interstrand crosslinks, the toxic lesion produced by alkylating anticancer agents. In the yeast *Saccharomyces cerevisiae* these crosslink-associated DSBs are primarily repaired by homologous recombination with non homologous end-joining (NHEJ) acting as a minor, salvage pathway. The key activity required for homologous recombination in yeast is the product of the *RAD52* gene, and DSB repair is almost eliminated in strains where this gene is disrupted (*rad52* cells). The *RAD52* epistasis group has five other members *RAD51, 54, 55, 57* and *59*, but unlike *RAD52* none of these genes are indispensable for all homologous recombination repair activities but show varying contributions depending on the substrate. Consequently we have investigated the relative role of several of these factors in the repair of nitrogen mustard crosslink associated DSBs. In addition to *RAD52* only *RAD54* is absolutely required for DSB repair, and cells lacking the *rad51* and *rad59* genes show more minor repair defects. This can be explained in terms of the recent observation that *RAD54* is absolutely required for sister chromatid exchanges in haploid cells, and *rad51* and *rad59* are partially redundant for mitotic homologous recombination. A further activity investigated in this study was *MRE11*, a gene involved in both homologous recombination and NHEJ. Mre11 deficient cells demonstrate a level of hypersensitivity to nitrogen mustard and DSB repair defect indistinguishable to that seen in *rad52* cells indicating that during ICL DSB repair Mre11 plays an indispensable role in the homologous recombination pathway.

We have also investigated the origin of the crosslink associated DSBs, and surprisingly found that they still formed in cells defective in nucleotide excision repair (NER), the main activity required for the excision of nitrogen mustard crosslinks. To establish that the 5′ and 3′ endonuclease activities of the NER apparatus could not act independently to initiate DSB formation we generated a strain defective in both these activities (*rad1 rad2* mutant). DSBs formed with the same frequency in this mutant as the parental, repair wild-type strain. Surprisingly, examination of the repair of DSBs in NER mutants indicated they are completely unable to repair these breaks, indicating that the ICL still persists in association with the DSB. The DSB apparently cannot be repaired until the crosslink is first eliminated. This suggests that DSBs form as a prerequisite to NER, and that the excision of a crosslink is contingent upon the formation of a strand break in order to permit access to the adduct.

### P140 INVESTIGATION OF DOUBLE STRAND BREAK REPAIR PROFICIENCY AND FIDELITY IN TESTICULAR GERM CELL TUMOURS (TGCT)

Rebecca E. Foster* and Christine M Chresta, School of Pharmacy, University of Manchester, Manchester M13 9PL, UK

Testicular cancer is the commonest solid tumour in young men. It is unique in its sensitivity to current chemotherapy regimes. We have shown that chemosensitivity is related to a low threshold for apoptosis activation (1). Levels of etoposide & bleomycin-induced DNA damage that are sub-lethal to normal human fibroblasts and bladder tumour cells rapidly activate apoptosis in TGCT. Significantly TGCT cells are preferentially sensitive to activation of apoptosis by agents that directly damage DNA. We demonstrate here that the microtubule poison Paclitaxel induces similar levels of apoptosis in TGCT and transitional cell carcinoma of the bladder cell lines. Two hour treatment with 180 nM Paclitaxel resulted in approximately 30% apoptosis in both cell types 24 hours after drug treatment. This specific hypersensitivity to agents which directly damage DNA suggests that TGCT may be compromised in DNA repair.

We have investigated three mechanism of DNA strand break repair, single strand break repair and the two types of double strand break repair, non-homologous end-joining and homologous recombination repair. DNA strand break repair proficiency and fidelity in TGCT has been compared to that of cell lines from transitional cell carcinoma of the bladder and normal human fibroblasts. Alkaline elution demonstrated that TGCT are equally as proficient as other cell types in single strand break repair with less than 5% breaks remaining just one hour after etoposide or bleomycin treatment. We could not directly quantify chemotherapy-induced DNA double strand breaks in TGCT because neutral elution requires higher doses of damaging agents and these induce rapid apoptosis in TGCT. We have therefore used a shuttle plasmid assay to investigate direct end-joining of double strand breaks generated by a restriction endonuclease. Fidelity of end-joining was found to be similar in all cell types (84±9% in fibroblasts and 84±9% & 88±6% in the TGCT cell lines 833 K & GCT27 respectively). Surprisingly rejoining frequency was 2–3 fold higher in TGCT (22 & 28%) than normal human fibroblasts (9%). Enhanced non-homologous end-joining may be related to overexpression of p53 in TGCT cells. It has recently been reported that p53 directly enhances rejoining of DNA strand breaks with cohesive termini (2). We are currently investigating if sensitivity to DNA strand break-inducing agents is related to reduced efficiency of homologous recombination repair since p53 inhibits the homologous repair pathway. p53 associated with Rad51, a protein involved in homologous recombination repair of double strand breaks, and has been shown to inhibit Rad51 activity. The high levels of p53 seen in the TGCT cells may therefore reduce homologous recombination repair.

1 Chresta et al (1996) *Cancer Res.* **56:** 1834–1841
2 Tang et al (1999) *Cancer Res.* **59:** 2562–2565

### P141 MEASUREMENT OF RESISTANCE TO THIOPURINE DRUGS IN CELLS WITH DEFECTIVE MISMATCH REPAIR

LA Hogarth*, EC Matheson, L Minto, MA Little and AG Hall, LRF, Molecular Pharmacology, CRU, Medical School, Newcastle NE2 4HH, UK

Errors occurring during normal DNA synthesis may produce mismatched base pairs. The mismatch repair (MMR) pathway primarily functions to correct DNA replication errors to avoid accumulation of mutations. The thiopurine drugs (6-mercaptopurine, 6-MP and 6-thioguanine, 6-TG) undergo intracellular activation to give cytotoxic thioguanine nucleotides which are incorporated into the DNA of dividing cells in place of guanine. Cell death is thought to result from futile attempts to repair the drug mismatch and defects in DNA mismatch repair have been identified as a potential cause of resistance to thiopurine drugs.

Risinger *et al.*, (1998) have transfected the HEC-1-A (endometrial carcinoma) cell line, which is defective in the mismatch repair protein PMS2, with wild type PMS2. We have shown that the HEC-1-A cell line is more resistant to both 6-thioguanine (6-TG) and 6-mercaptopurine (6-MP) than the PMS2 transfected HEC-1-A cell line (WT1) using the sulphurhodamine B assay. Compared with the HEC-1-A cell line the WT1 cell line has been shown, by western blotting, to have increased protein expression of PMS2 and also increased repair activity using a functional assay for DNA-mismatch repair (Thomas *et al*, 1995). Currently, we are investigating the sensitivity of these cells to 6-MP and 6-TG by measuring clonogenic survival and apoptosis. Sensitivity to 6-TG and 6-MP is also being compared with incorporation of these drugs into the DNA in order to assess the role of MMR in determining the ability of the cells to respond to drug incorporation.

Risinger JL, Umar A, Glaab WE, Tindall KR, Kunkel TA and Barrett C (1998) *Cancer Research* **58:** 2978

Thomas DC, Umar A and Kunkel TA (1995) *A Companion to Methods in Enzymology* **7:** 187

### P142 HIGH THIOPURINE METHYLTRANSFERASE EXPRESSION CONFERS DIFFERENT LEVELS OF RESISTANCE WITH 6-THIOGUANINE COMPARED TO 6-MERCAPTOPURINE

S Coulthard*, L Hogarth, L Minto, C Redfern and A Hall, LRF Molecular Pharmacology Group, 2nd Floor, Medical School, Newcastle University NE2 4HH, UK

Acute lymphoblastic leukaemia (ALL) is the most common malignancy of childhood. Current treatment results in long term survival in over 70% of cases. In previous studies it has been found that thiopurines given as part of continuing therapy are key agents in preventing relapse. However optimal administration during continuing therapy is often not achieved. Variations in the level of thiopurine methyltransferase (TPMT) activity appear to be a major molecular determinant of the extent of thiopurine metabolism.

In order to help understand the effect of TPMT activity directly on the metabolism of the thiopurine drugs, wild-type TPMT cDNA was cloned into a Muristerone-inductible expression vector and transfected into an embryonic kidney cell line, EcR293 (Invitrogen[R]). The insect hormone analogue, Muristerone A, (MA) activates gene expression via an ecdysone receptor in these cells. TPMT expression was induced in these cells in order to elucidate sensitivity of these cells to 6-thioguanine (TG) and 6-mercaptopurine (MP).

Optimal induction was obtained using 3 μM MA, which gave a TPMT activity of 2.7 nmol/hr/mg of protein. Activity was stable for 3 days. Assays for clonogenicity and cell proliferation (using the Sulforhodamine B (SRB) assay) were used to study the effect of TPMT expression on cellular properties. Clonogenic assays showed little difference in sensitivity to either drug in the TPMT expressing cells, (IC50=1.5 μM for TG and IC50=1.2 μM for MP) and the non-expressing cells (IC50=1.6 and 1.5 μM for TG and MP respectively). Using the SRB assay those cells expressing TPMT showed a slight difference in potency between the TG and MP (IC50=3.9 μM and 1.2 μM respectively). However, the non-expressing cells were more than 7 fold more sensitive to TG than MP. (IC50 = 2.2 μM and IC50 = 15.0 μM respectively).

This difference in sensitivity may be due to differences in metabolism. MP is converted into methlythioinosine monophosphate (a strong inhibitor of *de novo* purine synthesis) by TPMT, thereby making high TPMT expressing cells more sensitive to MP than the non-expressing cells. These data suggests that the effect of the thiopurine drugs is not solely due to the incorporation of fraudulent bases into DNA.

We should like to thank the Leukaemia Research Fund for supporting this work

### P143 GLUTATHIONE HOMEOSTASIS IN BUTHIONINE SULFOXIMINE SENSITIVE AND RESISTANT NEUROBLASTOMA CELL LINES

MC Case* and AG Hall, LRF Lab, CRU, Medical School, Newcastle NE2 4HH, UK

Neuroblastoma is the most common extracranial tumour of childhood. Metastatic disease is associated with a poor prognosis using currently available therapy. Recently it has been reported that many neuroblastoma cell lines are sensitive to exposure to buthionine sulfoximine (BSO) an inhibitor of gamma-glutamyl cysteine synthetase (GCS), the rate limiting enzyme in glutathione (GSH) synthesis at concentrations 10–100 times below the 3,000 to 8,000 μM peak plasma levels reported in human trials. We have investigated the mechanisms underlying this chemosensitivity.

The sulforhodamine B (SRB) assay confirmed previous reports that some cell lines (eg SHEP, SHEP tet 21 and IMR32) were very sensitive to BSO ($IC_{50}$ <16 μM) whilst the SKNBE cell line was resistant to all concentrations tested ($IC_{50}$ >2048 μM). Another cell line tested, SHSY5Y, was of intermediate sensitivity. Unlike previous reports there was no correlation with MYCN copy number. BSO sensitivity was dependant on the length of time cells were in culture prior to drug exposure; some cell lines (eg SHEP tet21) were killed by BSO however long they were left prior to drug exposure, some (eg SKNBE) were resistant to BSO at all time points tested and some (eg IMR32) gained resistance to BSO as they become more confluent. We speculated that this varied response to BSO could be due to the ability of different cell lines to synthesise and maintain cellular GSH levels. In BSO resistant SKNBE cells GSH levels rose as cells became more confluent. Real time PCR demonstrated a corresponding fall in levels of GCS mRNA (heavy and light chain), suggesting that the rise in GSH was due to a decrease in utilisation or export rather than an increase in synthesis. In BSO sensitive SHEP tet 21 cells GSH levels fell as cells became more confluent and the levels of GCS mRNA were low at all time points tested, suggesting a deficiency in GSH synthesis. In all cell lines tested, addition of BSO led to depletion of GSH-down to 10% of resting levels in the resistant cell lines and to <1% in the sensitive cell lines. Levels of GCS mRNA rose markedly after BSO exposure.

These results suggest that the control of GSH homeostasis differs between BSO sensitive and resistant cell lines, an observation which may have important implications for therapy. *Supported by the Neuroblastoma Society and NECCRF*.

### P144 GLUTATHIONE DEPLETING AGENT BSO SENSITIZES CHOLANGIOCARCINOMA TO RADIOTHERAPY

MC Okaro*[1&2], FE Cotter[2], BR Davidson[1], [1]University Dept Surgery RF & UCMS London NW3 2PF & [2]Dept Exp. Hematology, St Barts. and The R L S of Medicine London E1 2AD, UK

Cholangiocarcinoma (CCA) is resistant to the apoptosis inducing effects of radiotherapy and chemotherapy. Glutathione, an important anti-oxidant, is produced in the liver and secreted in high concentrations into the bile (8–10 mm). Reduced glutathione has been shown to inhibit apoptosis by alter the redox state of vicinal thiols located in the permeability transition pore complex (PTPC) of the mitochondria and also by the neutralizing reactive oxygen species (ROS) generated in the mitochondria. The effect of glutathione depletion of the sensitivity of CCA to radiotherapy has not been established.

Egi-1 and Tfk-1 human cholangiocarcinoma cell lines, were used to study the effects of glutathione depleting agent BSO on the rate and amount of apoptosis following stimulation with UV light and high dose radiotherapy (500–1000c Gy) over 72 hrs. The thiol-replenishing agents N-Acetylcysteine and chloromethyl-x-rosamine (CMX-Ros) were used to counter the effects of BSO.

Apoptosis was monitored by an established method, measuring the collapse in the inner mitochondrial membrane potential (ΔΨ m) detected at single cell resolution by flow cytometry using the ΔΨ m sensitive lipophilic flourochrome DiOC6 (3) and by the annexin V assay.

Depleting cholangiocarcinoma cells of glutathione using BSO had a significant effect at sensitizing cells of the DNA damaging and oxidative effects of UV light and radiotherapy. There was a larger (3-fold increase, $P < 0.01$) and accelerated rate in the collapse of mitochondrial function and plasma membrane disruption (apoptosis). This effect was both time and dose dependent as well as totally reversible.

These results show that the control of apoptosis in cholangiocarcinoma is highly dependent on the redox state of the cell and suggests that glutathione may be playing an important part in contributing to radioresistance in cholangiocarcinoma. Altering the redox state within the biliary tree may be one way of increasing the efficacy of radiotherapy in the treatment of this disease.

### P145 FORMATION OF ATYPICAL CISPLATIN DNA ADDUCTS IN CERTAIN CANCER CELLS

A Azim-Araghi*[1], KA Wright, CJ Ottley[2], DG Pearson[2], PR Twentyman[3], MJ Tilby[1], [1]Cancer Research Unit, University of Newcastle, Newcastle Upon Tyne, NE2 4HH, [2]Geology Dept, University of Durham, [3]UKCCCR, Lincoln's Inn Fields, London, UK

The adenocarcinoma lung cancer cell line Mor was 3 fold more resistant to cisplatin than the small cell lung cancer cell line H69 (1). To investigate the level of drug-target interaction in these cell lines, they were exposed to a range of concentrations of cisplatin (2 h), DNA extracted, and adduct levels determined using a competitive ELISA technique based on a monoclonal antibody (2). For the same drug exposures, the sensitive H69 cells consistently accumulated approximately 2 to 3 fold more adducts than the resistant cell line. However, when the *total* amount of cisplatin bound to DNA was quantified by atomic absorption spectrometry (AAS) or inductively coupled plasma mass spectrometry (ICP-MS) the difference in adduct levels between the two cell lines was less marked.

In contrast to the total Pt measured by AAS and ICP-MS, the ELISA method determined only immunoreactive adducts. Adducts formed in pure solution and in cells were previously found to be equivalent (1). The amount of adducts needed to cause 50% reduction in immunoassay signal were calculated from the results of all 3 techniques. This value was consistently >2-fold higher for adducts formed in Mor cells than for adducts formed in H69 cells or on purified DNA. (Table).

| Expt | Adducts (fmole per well) causing 50% reduction in assay signal | | Ratio Mor/H69 |
|---|---|---|---|
| | H69 | Mor | |
| 1# | 2.6 | 7.5 | 2.9 |
| 2# | 2.3 | 12.9 | 5.6 |
| 3# | 1.9 | 6.6 | 3.5 |
| 4* | 1.2 | 2.4 | 2.0 |
| 5* | 1.1 | 3.3 | 3.0 |
| 6* | 0.7 | 1.5 | 2.1 |

Each value represents the mean of 2–4 separate samples for each cell line.
### AAS *ICP-MS

If ELISA detects the predominant Pt-DNA adducts, a 2-fold change in overall immunoreactivity indicates a considerable difference in some aspect of Pt-DNA adduct structure in the Mor cells. ICP-MS detection limits of <1pg Pt/g will permit future analysis of samples exposed to lower and more clinically relevant drug levels.

1 Tilby et al (1991) *Cancer Res.* **51:** 123
2 Twentyman et al (1992) *Cancer Res.* **52:** 5674

### P146 *IN VITRO/IN VIVO* CORRELATIONS OF CISPLATIN-DNA ADDUCT LEVELS IN LEUCOCYTES OF CHILDREN

GJ Veal[1], C Dias[1], AV Boddy[1], E Price[2], A Parry[2], J Hale[2], ADJ Pearson[2], DR Newell[1] and MJ Tilby[1], [1]Cancer Research Unit and [2]Department of Child Health, University of Newcastle upon Tyne, Newcastle upon Tyne NE2 4HH, UK

It is generally accepted that the antitumour activity of cisplatin results from its interaction with DNA, resulting in the formation of platinum (Pt)-DNA adducts. In addition, clinical response to cisplatin has been reported to correlate with levels of Pt-DNA adducts in peripheral blood leucocytes (PBLs) induced by therapy.[1] Despite reports that Pt-adduct levels formed *in vivo* are related to cisplatin exposure (AUC of unbound cisplatin in plasma),[2] recent findings in a paediatric patient population suggested that pharmacokinetic variation could only account for a small proportion of the interpatient variation in adduct levels.[3] The present study investigates possible relationships between levels of Pt-DNA adducts determined *in vivo*, levels of DNA adducts formed following incubation of pretreatment blood samples with cisplatin *in vitro* and the pharmacokinetics of cisplatin in children with cancer.

Pt-DNA adducts were measured in PBLs isolated from 7 children at 0, 6, 24 and 48 h following the start of administration of cisplatin (50–100 mg/m$^2$) as a 24 h intravenous infusion. Pretreatment samples of whole blood were also incubated with 50 µM cisplatin for 1 h prior to isolation of PBLs. Adduct measurements were performed by ELISA and total and unbound platinum concentrations at 0, 1, 2, 6, 24 and 48 h determined in plasma and plasma ultrafiltrate samples respectively by Atomic Absorption Spectroscopy (AAS).

A strong correlation ($r^2$=0.90) was observed between *in vivo* Pt-DNA adduct $AUC_{0–48\ h}$ and *in vitro* Pt-DNA adduct levels normalised to cisplatin dose. No significant correlation was seen between *in vivo* Pt-DNA adduct $AUC_{0–48\ h}$ and free (unbound) Pt $AUC_{0–48\ h}$ ($r^2$=0.08) or total Pt $AUC_{0–48\ h}$ ($r^2$=0.07) in plasma, indicating that cellular factors play a more important role than pharmacokinetic variation in determining cisplatin-DNA interactions *in vivo*. These preliminary results suggest that *in vitro* adduct levels following a 1 h incubation with cisplatin are a good predictor of Pt-DNA adduct levels formed *in vivo* and hence potentially of clinical response to cisplatin in children.

1 Reed E, Parker RJ, Gill I *et al* (1913) *Cancer Res* **53:** 3694–3699
2 Schellens JHM, Ma J *et al* (1996) *Br J Cancer* **73:** 1569–1575
3 Peng B, Tilby MJ, English MW *et al* (1997) *Br. J. Cancer* **76:** 1466–73

### P147 PRECLINICAL COMBINATION STUDIES IN HUMAN OVARIAN CARCINOMA CELL LINES WITH THE NOVEL PLATINUM COMPLEX, ZD0473 (*CIS*-AMMINEDICHLORO [2-METHYLPYRIDINE] PLATINUM(II) AND PACLITAXEL

PM Rogers*[1], F Boxall[1], CP Allott[2], T Stephens[2], and LR Kelland[1], [1]CRC Centre Cancer Therapeutics, Institute of Cancer Research, Sutton, Surrey SM2 5NG & [2]AstraZeneca, Alderley Park, Macclesfield, Cheshire, UK

ZD0473 is a novel "sterically hindered" platinum complex targeted to circumvent classical resistance mechanisms to cisplatin, especially resistance mediated by increased thiol-containing molecules such as glutathione and metallothionein. ZD0473 was shown to circumvent acquired cisplatin resistance in various human ovarian carcinoma cell lines and xenografts resulting in its selection for clinical trial. Myelosuppression was the dose-limiting toxicity observed in the initial phase I trial using intravenous infusion every 3–4 weeks. The drug is now undergoing phase II evaluation. The aim of this *in vitro* study was to determine the potential of combining ZD0473 with the widely used drug paclitaxel, in the clinic. Four human ovarian carcinoma cell lines have been used: two parent cisplatin-sensitive lines, CH1 and A2780; a 15-fold acquired cisplatin resistant subline, A2780cisR; and an A2780 subline stably transfected with the E6 human papillomavirus gene thereby disabling the wild-type p53 function of the parental line, A2780 E6. Growth inhibition (using the sulforhodamine B assay) has been determined for ZD0473 alone, and combined either simultaneously or administered sequentially for 24 hours with paclitaxel. The effect of the drug combinations has been analysed using median effect analysis. ZD0473 alone (96 hour continuous exposure) exhibited potent growth inhibition against these cell lines with $IC_{50}$ values µM) of 3.7 for A2780, 15.6 for A2780cisR, 8.6 for A2780E6 and 3.3 for CH1. Hence. A2780cisR was only 4.2-fold cross-resistant to ZD0473 in these studies. Paclitaxel alone also conferred potent growth inhibition with $lC_{50}$ values in nM of 2.5 for A2780, 3.5 for A2780cisR, 20.8 for A2780E6 and 3.1 for CH1. When exposed concomitantly with paclitaxel the combination index (CI) values at 50% fraction affected were synergistic for all 4 cell lines (A2780 CI of 0.49, A2780cisR CI of 0.55, A2780E6 CI of 0.41, CH1 CI of 0.69). However, when ZD0473 was applied for 24 hours before paclitaxel for 24 hours the effect was additive in A2780 (CI of 1.0) whilst synergistic effects were obtained in the other 3 cell lines (A2780cisR CI of 0.4, A2780E6 CI of 0.51 and CH1 CI of 0.61). When cells were exposed to paclitaxel for 24 hours followed by ZD0473 for 24 hours a strong synergistic effect was only observed in A2780cisR (CI of 0.37) with the other cell lines giving weak synergism (A2780E6 CI of 0.89), an additive effect (A2780 CI of 1.1) or an antagonistic effect (CH1 CI of 2.4). These data indicate that, depending upon the cell line, the sequence in which ZD0473 and paclitaxel are administered is of importance in determining growth inhibition when combined.

### P148 PROSPECTIVE COHORT STUDY OF THE USE OF MAGNESIUM SUPPLEMENTATION WITH CISPLATIN CHEMOTHERAPY

JW Adlard*[1], J Watters[1], [1]Yorkshire Centre for Clinical Oncology, Cookridge Hospital, Leeds LS16 6QB, UK

Cisplatin chemotherapy can cause glomerular injury leading to decreased glomerular filtration rate and tubular damage manifesting as urinary electrolyte wasting, particularly of magnesium. The aim of the study was to assess the effectiveness of routine magnesium supplementation in preventing hypomagnesaemia in patients receiving cisplatin-containing chemotherapy. 85 consecutive patients were evaluated in this prospective cohort study. Eligible patients were those with adequate renal function, who had not previously received cisplatin-containing chemotherapy. Renal function and electrolytes including serum magnesium levels were measured prior to each cycle of chemotherapy. Intravenous magnesium chloride supplementation was given routinely with each cycle. Doses of magnesium chloride per cycle varied from 8 mmol to 72 mmol dependent on the particular chemotherapy regimen, though most patients (66%) received 16 mmol per cycle. Continuous oral magnesium glycerophosphate supplementation (8 mmol three times daily) was given from the following cycle when serum magnesium levels fell below 0.65 mmol/l.

A total of 264 cycles of cisplatin-containing chemotherapy were given to the 85 patients. Serum magnesium level fell significantly from the second cycle of chemotherapy onwards. Mean serum magnesium prior to cycle 1 = 0.77 mmol/l (95% C.I. 0.752–0.787) and prior to cycle 4 = 0.695 mmol/l (95% C.I.0.663–0.728) P<0.001. Hypomagnesaemia (<0.65 mmol/l) occurred in 26% of patients and complicated 14% of all chemotherapy cycles. Larger doses of intravenous magnesium used with some regimens such as BEP (Bleomycin, Etoposide and Cisplatin) given over several days did not appear to protect against hypomagnesaemia. There were no definite recorded symptomatic episodes. There were no other significant changes detected in renal indices or electrolytes. Continuous oral magnesium supplements appeared promising in improving hypomagnesaemia occurring despite intravenous supplementation. The cost of cisplatin for the duration of the study was approximately £9900, compared with £1500 for the intravenous magnesium chloride and £630 for oral magnesium supplementation.

Hypomagnesaemia remains common despite current intravenous magnesium supplementation, though is rarely symptomatic. Oral magnesium supplements may help maintain magnesium levels, but add to the costs of treatment and are of uncertain benefit in asymptomatic patients.

### P149 HYPERSENSITIVITY REACTIONS TO CARBOPLATIN

VR Bulusu*[1], F Ives[1], E Sugden[2], [1]Oncologycentre, Addenbrooke's Hospital, Cambridge CB2 2QQ and [2]Dept of Clinical Oncology, Churchill Hospital, Headington, Oxford OX3 7LJ, UK

**Aim** 1. To characterise the clinical features of hypersensitivity reaction (HSR) to carboplatin. 2. To correlate the relationship of the severity and duration of HSR to the carboplatin infusion rate and concentration and time interval between initial exposure and subsequent rechallenge with carboplatin.

**Material and Methods** A proforma has been developed to document the clinical features of HSR to carboplatin. The HSR was graded in to I, II and III on the basis of the severity of symptoms. 24 patients (19-ovarian cancer, 3-fallopian tube cancer, 1-primary peritoneal cancer, 1-adenocarcinoma with unknown primary) who have experienced a HSR to carboplatin were included in the study. The overall incidence in all patients treated was 4.5% and in patients who were rechallenged with carboplatin was 10.5%. The mean age was 57.8 years (range 33–75 years). 6 patients had prior history of an allergic diathesis. The time interval, duration and concentration of carboplatin infusion, the grade and duration of reaction, the clinical features, and the management were documented.

**Results** The mean time interval between the initial exposure and to carboplatin and the subsequent development of HSR was 26.5 months (range 5–96 months). 50% (12/24) have developed the HSR on the 2nd cycle of the rechallenge. The mean duration of HSR was 44 min (range 10–180 min). The most common symptoms were itchy hands, facial flushing, generalised erythema, palpitations and nausea. Grade 1 reaction was noted in 45%, grade II in 25% and grade III in 30% of patients. There was no correlation between the time interval, infusion rate, drug concentration and the severity of HSR. The mean time of onset of symptoms (n=10) was 14.5 min (range 5–20 min). No patient developed HSR after completing the infusion. No ITU admission was required. None of the reactions were fatal. All patients had their infusion stopped, and treated with iv hydrocortisone and antihistamine, and oxygen as required. 9 patients were rechallenged with a platinum compound and 6/9 (66%) developed a HSR despite full steroid and antihistamine cover.

**Conclusions** We have highlighted the clinical features and the pattern of HSRs to carboplatin. Patients who are rechallenged are at a significantly higher risk of developing a HSR. Hypersensitivity typically occurred on the 2nd cycle of rechallenge. Relapsed ovarian cancer patients to be rechallenged with carboplatin should be made aware of the possibility of a HSR. Patient education regarding the symptom recognition may help prompt diagnosis and treatment. Chemotherapy nurses managing these patients should be equally aware of the clinical features and management of the HSRs. We do not recommend re challenge with carboplatin in patients who have had a prior HSR.

### P150 TOXICITY, CELLULAR UPTAKE AND DNA CROSSLINKING STUDIES ON THE NOVEL BIOREDUCTIVE ANTICANCER DRUG RH1

TH Ward*[1], Nic Coe[1], R Hargreaves[1] J Butler[1] and AT McGown[1], [1]CRC Drug Development Group, Paterson Institute, Christie Hospital Manchester M20 4BX, UK

DT-diaphorase; (EC. 1.6.99.2) is an obligate two-electron reductase that reduces quinones to hydroquinones. With certain quinone anticancer drugs, this two-electron reduction can produce a more biologically reactive species which can cross link DNA. DT-diaphorase has been shown to be over expressed in many tumours relative to the normal tissue and consequently represents a target for bioreductive anti-cancer drugs.

RH1 (2,5-diaziridinyl-3-[hydroxymethyl[-6-methyl-1,4-benzoquinone) is a novel bioreductively activated DNA crosslinking agent which is an efficient substrate for DT-diaphorase. This agent has been shown to be effective in a variety of xenograft models and in particular, against ovarian tumours. RH1 is currently to undergo Phase I/II trials as a joint collaboration between the CRC and the NCI. The toxicity, cellular uptake and DNA crosslinking ability of this drug has been investigated in diaphorase rich (H460) and diaphorase deficient (BE) cell lines. In addition a comparison between this drug and other clinical DNA crosslinking anticancer agents has been made using cultures of primary ovarian cancer cells. Uptake of $^{3}$H RH1 was shown to be both time and concentration dependent. Uptake appeared maximum after 2 hrs and correlated well with DNA crosslinking as measured by the comet assay. DNA crosslinking could be detected at concentrations of drug as low as 10 nM after only 2 hrs exposure. The time dependency of DNA Crosslinking showed a rapid initial phase over the first 4 hrs exposure reaching a maximum within 8 hrs. The DNA crosslinking activity of three clinically used anti-cancer drugs (Melphalan, mitomycin C and CisPt) in cultures of primary ovarian cancer cells was compared to RH1. The Comet crosslinking assay showed that DNA crosslinking occurred at a significantly lower concentration (100×) with RH1 than with any of the other drugs used. Furthermore the amount of initial crosslinking produced by all drugs appeared to decrease on incubation in drug free medium overnight suggesting possible repair of the DNA adducts. Cytotoxicity assays comparing these and other clinical anticancer drugs showed that RH1 was 10 times more toxic than taxol and 50 time more toxic than mitomycin-C in these cells. Clearly RH1 represents a potent bioreductive anticancer drug and may prove effective in the treatment cancers, particularly those over expressing DT-diaphorase.

### P151 EVIDENCE FOR ENHANCED ALKYLATION OF G-RICH REGIONS OF DNA BY MELPHALAN IN CELLS COMPARED TO PURE SOLUTION

H McCartney* and MJ Tilby, Cancer Research Unit, University of Newcastle, Newcastle Upon Tyne NE2 4HH, UK

Monoclonal antibodies MP5/73 (1) and Amp4/42 (2) appeared to recognise respectively, N7 guanine adducts formed by melphalan and the corresponding alkali-induced formamidopyrimidine ring-opened structure. However, analysis of DNA extracted from drug-treated cells implied that adducts recognised by MP5/73 and Amp4/42 were not equivalent and that adducts formed in cells differed from those formed *in vitro*.

Denatured DNA was reacted with $^{3}$H-melphalan and exposed for various periods to alkali. Immunoreactivities of adducts to each antibody were determined by competitive ELISA. Half-lives for loss of MP5/73 immunoreactivity were ca. 66 and 11 min., however, immunoreactivity to Amp4/42 increased with half-lives of ca 23 and <2 mins, in 0.01 and 0.1 M NaOH respectively. This difference in rates confirmed a difference in properties of adducts recognised by each antibody. At 100 °C, pH7, the rate of loss of immunoreactivity of adducts on RNA was approximately 10 times slower than the rate for adducts on DNA (t1#fr2 = 68 and 6 mins for RNA and DNA respectively). The effects of alkali and heat both rule out the involvement of phosphotriesters in antibody recognition. To test the hypothesis that local DNA sequence influenced the immunoreactivity of adducts, synthetic DNA oligomers were covalently immobilised to wells of 96-well plates and exposed to a range of melphalan concentrations. The binding of Amp4/42 and MP5/73 to the wells was investigated +/– prior incubation with 0.1 M NaOH. All sequences containing guanine showed the formation of adducts recognised by antibody MP5/73. However, adducts on oligo dG were recognised very poorly by Amp4/42. These results were confirmed by competitive ELISA experiments.

In drug-treated cells, adducts recognised by Amp4/42 formed a 10-fold smaller proportion of the total adducts than for DNA alkylated in pure solution (2). The present results indicate that this resulted from a greater proportion of the alkylation in cells occurring in G-rich regions. Unlike previous comparisons of sequence dependent DNA alkylation *in vivo* and *in vitro*, the immunoassay data reflects the properties of the total cellular DNA rather than a specific small region. The results could indicate that in cells, DNA alkylation occurs predominantly in regions associated with a specific chromatin organisation (e.g. telomeres). It will be of interest to identify these regions and to determine the rate DNA repair.

1. Tilby et al (1987) *Cancer Res.* **47:** 1542
2. Tilby et al (1995) *Carcinogenesis* **16:** 1895

### P152 ANTI-TUMOUR PROPERTIES OF NOVEL DIAZIRIDINYLQUINONES

Angela M Di Francesco[1,2], Robert HJ Hargreaves[2], Timothy W Wallace[3], Stephen P Mayalarp[4], Ali Hazrati[5], John A Hartley[5] and John Butler[1,2], [1]Departments of Biological Sciences and [3]Chemistry, Salford University, Salford M5 4WT, UK, [2]CRC Section of Drug Development, Paterson Institute for Cancer Research, Christie Hospital NHS Trust, Manchester M20 9BX, UK, [4]PE Biosystems, & Kingland Grange, Warrington WA1 4SR, UK, [5]CRC Drug-DNA Interactions Research Group, Department of Oncology, Royal Free and University College Medical School (UCL), London W1P 8BT, UK

Diaziridinylquinones (DQs) have been extensively studied as potential anti-tumour agents, which are bioreductively activated to form toxic species. DQs can target the obligate two-electron reducing enzyme DT-diapohrase (DTD), which is over-expressed in some breast, colon, ovarian and non small cell lung cancers (NSCLC). Depending upon the chemical structure of diaziridinylquinones, DNA strand breaks, monoalkylations of DNA and proteins, and DNA cross-links can be produced after bioactivation. 2,5-diaziridinyl-3-phenyl-1,4-benzoquinone (PDZQ) is a known DNA cross-linking agent with cytotoxicities ranging from 2 to 16 nM ($IC_{50}$ values) in different cell lines. The present study has so far concentrated on ester derivatives of PDZQ which appear to be as much as 70 times more toxic than PDZQ in colon, lung, ovarian and leukaemia cell lines. Although these compounds did not produce high toxicity differentials between DTD-rich and deficient cell lines, they were also found to be substrates for the enzyme. Two ester isomers were selected for more detailed investigations. They were shown to be cleaved by esterases to form a stable *meta*-phenol and an unstable *para*-phenol. Flow cytometry studies on A2780 cell line, showed that after overnight treatment at $10\times IC_{50}$ concentrations, PDZQ produced a much stronger block in $G_2$-M than the two esters and the *meta*-phenol. On the other hand a plasmid assay on the A2780 cell line showed that at 1 µM concentrations and after chemical reduction, the *meta*-phenol and the two esters were much better DNA cross-linkers than PDZQ. This finding was confirmed by a COMET assay performed at 50 nM concentrations in the same cell line. An apoptosis assay on A2780 cell line using fluorescein-12-dUTP, did not detect any evidence for extensive apoptosis with any of the compounds tested. It is therefore not possible at this stage, to derive a clear mechanism which can explain the difference in the cytotoxicities of PDZQ and the phenol/esters. However, the overall conclusion so far is that the toxicities are not simply due to the differences in DNA cross-linking efficiencies. The similarity between both the structures of the *meta* and *para*-phenols and those of known kinase inhibitors, would suggest a possible involvement of kinases. Further studies are therefore underway to investigate the role of kinases in the cytotoxicities of these phenols and esters.

The funding for this project comes from the Association for International Cancer Research.

### P153 COMBINATORIAL PARALLEL SYNTHESIS AND EVALUATION OF CELL GROWTH INHIBITORY PROPERTIES OF SUBSTITUTED CHALCONES

NJ Lawrence,[1,*] D Rennison,[1,2] AT McGown,[2] S Ducki,[1,2] LA Gul[1] and N Khan[1], [1]Dept Chemistry, UMIST, PO Box 88, Manchester M60 1QD, UK: 2. CRC Section of Drug Development, Paterson Inst., Manchester M20 4BX, UK

A series of 644 chalcones was prepared by solution phase parallel combinatorial synthesis using the Claisen-Schmidt base-catalysed aldol condensation of substituted acetophenones and benzaldehydes. The cytotoxicity of these chalcones was determined directly from the crude products in 96 well microtitre testplates. This method revealed seven chalcones of $IC_{50}$ less than 1 µM of which 4-hydroxy-2,4,6,3′-tetramethoxychalcone was the most active [$IC_{50}$ (K562) 30 nM].

In total, 23 substituted acetophenones were applied in this study along with 28 substituted benzaldehydes (selected randomly) to afford 644 chalcones. This provided 16 hits, a 'success' rate of 2.5%. The saving in time is impressive. The 644 chalcones were prepared and evaluated in approximately 20 days. We estimate that it would take approximately 850 days to prepare and test the same number of chalcone in a conventional serial manner. The most active compound 4-hydroxy-2,4,6,3'-tetramethoxychalcone is impressively cytotoxic ($IC_{50}$ 30 nM) and presents a compound of great potential. We will report on its mode of action and its potential use as a drug to target tumour vasculature. The method clearly shows that *in vitro* cell growth inhibition can be used as an assay for high throughput screening.

### P154 SYNERGISTIC EFFECTS BETWEEN LY294002, A SPECIFIC PI-3 KINASE INHIBITOR AND DOXORUBICIN IN MDA-MB-231 BREAST CANCER CELLS

LSM McCarragher[1], KA Al-Sakkaf[2], BL Brown[1] & PRM Dobson[2], [1]Department of Human Metabolism and Clinical Biochemistry; [2]Institute for Cancer Studies, Sheffield University Medical School, Sheffield, UK

The need for new approaches to cancer therapy is clear and cellular signalling pathways offer special targets. These novel modalities could overcome, at least in part, the major problems which occur with the current generation of drugs. Although it should be possible to use anti-signalling agents as the drugs of choice, the aim of this work was to investigate possible synergistic reactions between signalling drugs and 'classical' chemotherapeutics, with the objective of lowering the doses of DNA-damaging drugs. The PI-3 kinase pathway is a major survival pathway expressed in the majority of cancers including those of the breast. We investigated the possible synergistic effects of two different treatments, LY294002, a specific PI-3 kinase inhibitor and doxorubicin, a classical cytotoxic agent. The aim of this study was to achieve a similar effect seen using a high dose of doxorubicin, but using a much lower dose of the drug in order to reduce its non-specific cytotoxicity, in combination with a low dose of LY294002. MDA-MB-231 breast cancer cells were preincubated with LY294002 (1 µg/ml) for 30 minutes followed by 8–1000 ng/ml doxorubicin for 48 hours. Growth inhibition was assessed by measuring cell numbers using a Coulter counter. Cell cycle analysis was carried out using flow cytometry and the *In Situ* Nick Translation assay was used to detect DNA fragmentation (used as a monitor of apoptosis). Drug synergy was detected with lower doses of doxorubicin (8 & 10 ng/ml) giving a percentage growth inhibition of 45% as single treatments and 61% and 72% respectively in the presence of 1 µg/ml LY294002 (4.3% growth inhibition as a single treatment). Significant differences were seen between doxorubicin alone and in combination with LY294002 ($F=7.29$; $P<0.01$) suggesting synergistic affects. Growth inhibition, as a measure of percentage loss of control, was supported by cell cycle analysis showing enhanced G2 arrest. At these low doses of doxorubicin, DNA fragmentation was not detected. This study supports the investigation of the inhibition of survival pathways as direct targets for cancer therapy.

We are grateful to Yorkshire Cancer Research for funding.

### P155 ZOLEDRONATE INDUCES APOPTOSIS OF BREAST CANCER CELLS *IN VITRO* IN COMBINATION WITH PACLITAXEL AND TAMOXIFEN: EVIDENCE FOR SYNERGISTIC EFFECTS

S Jagdev*[1], P Croucher[2], R Coleman[1], [1]Weston Park Hospital, Sheffield, [2]University of Sheffield, Sheffield, UK

Bisphosphonates are widely used in the treatment of breast cancer-induced bone metastases both alone and in combination with other antineoplastic therapies. In the adjuvant setting, recent clinical data suggest that the use of bisphosphonates may affect the development of extra-skeletal as well as skeletal metatstases. We have previously shown that the potent third generation bisphosphonate zoledronate significantly reduces MCF7 and MDA 231 cell number and induces apoptosis in a dose-dependent fashion *in vitro*. We have also recently demonstrated that these effects on breast cancer cell apoptosis may be mediated via inhibition of enzymes of the mevalonate pathway. The aim of this present study was to investigate the effects of short term exposure to zoledronate on MCF7 breast cancer cell apoptosis. We also investigated the effects of combining zoledronate with tamoxifen and with taxol on at clinically relevant concentrations on breast cancer cell apoptosis. MCF7 cells were exposed to zoledronate (100 µM) for 2, 6, 24 and 72 hours. There was a significant increase in the proportion of apoptotic cells, as measured by a flourescence *in situ* nick translation assay, after 2 and 6 hours of exposure to zoledronate with a corresponding reduction in healthy cell number compared with control. Treatment with a combination of zoledronate (10 µM) and taxol (2 nM) resulted in a four-fold increase in the levels of apoptosis compared with either drug alone. The combination of zoledronate (10 µM) and tamoxifen (0.01 µM) resulted in a greater than two-fold increase in the proportion of cells showing changes in nuclear morphology characteristic of apoptosis. These data suggest that exposure to zoledronate for as short a period as two hours is effective in inducing significant levels of MCF7 cell apoptosis. The combination of zoledronate and taxol may have additive effects on breast cancer cell apoptosis and there may be evidence for a synergistic effect when tamoxifen and zoledronate are combined. These observations warrant further *in vitro* and *in vivo* investigation.

### P156 SELECTIVE TARGETING OF THE NOVEL CYCLOPENTA[g]QUINAZOLINE-BASED THYMIDYLATE SYNTHASE (TS) INHIBITORS CB300638 AND CB300907 TO α-FOLATE RECEPTOR OVER-EXPRESSING CELLS *IN VITRO*

D Theti*, C Melin, V Bavetsias, L Skelton and AL Jackman, CRC Centre for Cancer Therapeutics, Institute of Cancer Research, Sutton, Surrey, UK

The α-isoform of the folate receptor (α-FR) is a high-affinity, low-capacity transporter of folic acid and reduced-folate co-factors ($K_d=0.1$–1 nM) when upregulated or constitutively over-expressed. Although levels of the α-FR are either absent or present at low to moderate levels in normal tissues, the receptor can be constitutively over-expressed to very high levels in some solid tumours e.g. in ovarian with high incidence. Thus, over-expression of the α-FR may be an exploitable property of some tumours, allowing the selective delivery of chemotherapeutic agents into tumour cells and consequent inhibition of intracellular targets such as TS. Quinazoline-based TS inhibitors such as raltitrexed (RTX) and ZD9331 bind to the α-FR with high affinities (close to that of folic acid), but preferentially utilise the ubiquitously expressed reduced-folate carrier (RFC) to gain entry into cells (Km's=1–2 µM). However, compounds with lower affinities for the RFC, but high affinities for the α-FR are predicted to be highly selective for α-FR over-expressing cells compared with normal tissues. The pure 6S-isomers of the novel cyclopenta[g]quinazoline-based compounds (with dipeptide-based ligands), CB300638 and CB300907 are potent TS inhibitors ($Ki_{app}=0.52$ and 0.16 nM respectively) with low affinities for the RFC (Km's>100 µM) and high affinities for the α-FR (close to that of folic acid). Consistent with these data, under physiological conditions of reduced-folates (20 nM leucovorin; LV), CB300638 and CB300907 are ~250-fold more potent against A431-FBP cells (transfected with the α-FR) ($IC_{50}=4$ and 15 nM respectively) compared to neo-transfected A431 cells ($IC_{50}=800$ and 1500 nM respectively). A 160-fold reversal in the activities of these compounds against A431-FBP cells in the presence of 1 µM folic acid is consistent with compound uptake via the α-FR. Under the same conditions, the quinazoline-based compound CB3717 which has a moderate affinity for the RFC (Km=43 µM) and high affinity for the α-FR (higher than that of folic acid) is less selective for A431-FBP cells over A431 cells ($IC_{50}=270$ and 1500 nM respectively). CB300638 and CB300907 have $IC_{50}$'s of ~ 1000 nM in non-α-FR expressing cell lines. However, human KB cells which constitutively over-express the α-FR are highly sensitive to these agents, with $IC_{50}$'s ranging from 3.7–24 nM, which increases by up to 100-fold in the presence of 1 µM folic acid. This is in contrast with ZD9331 and RTX, which have similar activities in the presence and absence of 1 µM folic acid. Together, these data suggest that in the over-expressed-state, the α-FR can be exploited to selectively deliver TS inhibitors into tumour cells and that CB300638 and CB300907 are two examples of compounds with these properties.

### P157 USE OF cDNA ARRAY METHODOLOGY TO ASSESS RESISTANCE TO TS INHIBITORS AND POTENTIAL FOR CO-EXPRESSION OF IMPORTANT ONCOGENES

W Wang*, J Cassidy and HL McLeod, Dept of Medicine and Therapeutics, University of Aberdeen, Foresterhill, Aberdeen AB25 2ZD, UK

Thymidylate synthase (TS) catalyses the *de novo* synthesis of dTMP from dUMP and is an attractive target for the design of anticancer drugs. TS inhibitors (e.g. 5-fluorouracil [5FU] and tomudex [TDX]) have been used for treatment of various kinds of human cancers. Chemoresistance is still a major barrier to the clinical use of these agents. The development of cDNA array techniques makes it possible to quantitate the expression of thousands of genes in a single experiment. This technique has been used to analyze aberrant gene expression in a variety of human cancers. In this project, the expression patterns of 1176 genes was observed from 5 pairs of TS inhibitor resistant cell lines (colorectal cancer: H630-TDX, H630–5FU and RKO-TDX; breast cancer: MCF-7-TDX; lymphoblast: W1L2-TDX). The aim of this work is the analysis of global gene expression patterns in drug resistant cell lines to identify potential molecular targets for drug development. Expression of 20–35% genes on the array was detected in the different cell lines. The expression levels of drug resistant cell lines were compared with those of parent cell lines. Since the variation of 95% genes from the same cell line was lower than 2-fold, this standard was used to judge over- or under-expression. cDNA array results demonstrated that gene expression patterns were highly variable between different cell lines and more genes were overexpressed in 5FU resistant H630 cell line than the TDX resistant cell lines. Only TS mRNA is overexpressed in all of the drug-resistant cell lines (1.8 to 4.5 fold). Northern blotting analysis confirmed the cDNA array results. TS protein is overexpressed in every resistant cell line and at much higher level comparing to mRNA. This suggests that post-transcriptional and translational manipulations are also involved in the TS dysregulation. Southern blotting indicates that TS overexpression is derived from gene amplification. *c-yes* oncogene, situated 50 kb away from TS, was also amplified and the overexpression of *c-yes* mRNA and protein was detected in 4/5 drug-resistant cell lines. Unexpectedly, *c-yes* gene was amplified, but mRNA and protein down-regulated, in the drug-resistant lymphoid cell line W1L2. Conclusions: 1. cDNA array is a reliable measure for molecular genetic analysis of drug resistance. 2. TS appears to drive gene amplification, carrying along adjacent genes such as c-yes oncogene. Overexpression of the latter may confer the drug resistant tumour more aggressive biological behavior. 3. Some as yet unknown factors block the expression of C-Yes in drug-resistant non-malignant cell line W1L2.

### P158 CASSETTE DOSING IS A VALID APPROACH TO EVALUATE THE PHARMACOKINETICS OF TRISUBSTITUTED PURINES SUCH AS OLOMOUCINE, ROSCOVITINE AND BOHEMINE

FI Raynaud, BP Nutley, PM Goddard, P Fischer, S McClue, D Lane and P Workman, CRC Centre for Cancer Therapeutics, The Institute of Cancer Research, 15 Cotswold road, Sutton, Surrey, UK; *Cyclacel Limited, Dundee Technopole, James Lindsay Place, Dundee, UK

Pharmacokinetic analysis is frequently a bottleneck in drug development. Cassette dosing or cocktail dosing allows high pharmacokinetic throughput by administering to animals a combination of analogues. The use of liquid chromatography mass spectrometry (LC-MS/MS) with multiple reaction monitoring allows the specific and sensitive measurement of the compounds in the same sample. In order to validate the use of cassette dosing, Balb $C^-$ mice were injected with either a mixture of olomoucine, roscovitine and bohemine each at 16.6 mg/kg, or with the individual compounds at 50 mg/kg. Separation of the 3 compounds and the internal standard, 6 benzylaminopurine was achieved on 5 cm ABZ+ column with a flow rate of 0.6 ml/min with a 3 minutes gradient of 20–100% methanol in formic acid. Selected reaction monitoring of the appropriate parent ions and daughter fragments was subsequently performed. A standard curve was produced in mouse plasma. Pharmacokinetic parameters were calculated using non-compartmental analysis. The pharmacokinetic parameters estimated derived from compounds dosed in cocktail showed that the overall ranking of the compounds was maintained; roscovitine having the highest AUC and the lowest clearance. Furthermore, the pharmacokinetic parameters of drugs in combination showed highly significant correlation with that of drugs alone (spearman test r= 0.90 for olomoucine, r=0.87 for bohemine and r=0.99 for roscovitine). The results suggest that cassette dosing is a valid approach to evaluate the pharmacokinetics of trisubstituted purine analogues. Our data suggest that roscovitine, which is the most potent analogue of the 3 compounds and shows the best pharmacokinetic profile, is a suitable pharmacokinetic standard for cassette dosing of trisubstituted purine derivatives. *This work was supported by the Cancer Research Campaign and Cyclacel Ltd.*

### P159 OXALIPLATIN (L-OHP) DOES NOT INFLUENCE THE PHARMACOKINETICS OF 5-FLUOROURACIL (5-FU)

SP Joel[1], F Richards[2], F Halstead[2], M Seymour[2], ICRF Depts of Medical Oncology, St Bartholomew's Hospital, London and Cookridge Hospital Leeds, UK

**Background** The combination of 5-FU and L-OHP is used increasingly in the treatment of colorectal cancer (CRC). We have previously suggested that L-OHP may alter the pharmacokinetics (PK) of 5-FU, based on a between-patient comparison (ASCO 1998, ABS 777). This has been investigated further in a crossover study.

**Patients/Methods** Ten patients with advanced CRC received, in random order two weeks apart, two cycles of chemotherapy using the Modified de Gramont (MdG) and Oxaliplatin-MdG (OxMdG) regimens, defined as follows: MdG: 350 mg leucovorin (LV) i.v. infusion over 2 hrs; then 5-FU 400 mg/m$^2$ i.v. bolus over 5 minutes; then 5-FU 2400 mg/m$^2$ i.v. infusion over 46 hours. OxMdG: the same as MdG, except for the addition of L-OHP 85 mg/m$^2$ i.v. infusion over 2 hours concurrent with LV. Blood samples were taken at ten timepoints out to 4 hours, and at 24 hours. Samples were immediately cold-spun and the plasma frozen. Plasma 5-FU was determined by HPLC analysis. 5-FU PK were derived using a 1 compartment model with linear and non-linear elimination pathways, as previously described (*Br J Cancer 70:724–8, '94*). The $K_m$ of the non-linear pathway was set at 1.95 µg/mL (15 µM), representing dihydropyrimidine dehydrogenase catabolism. The $AUC_{0-24}$ was determined by the trapezoidal method.

**Results** The mean 5-EU concentration-time curves with and without L-OHP are virtually identical. PK data are summarised in the table (values ± s.d.). There are no differences in any PK parameter for 5-FU with or without L-OHP (paired t-test >0.10).

**Table 1.** 5-FU PK ± L-OHP (Mean ± SD)

| | Vd | Vmax | Kelim | $AUC_{0-24}$ |
|---|---|---|---|---|
| | (L) | (µg/ml.hr$^{-1}$) | (hr$^{-1}$) | (µg/ml.hr$^{-1}$) |
| 5-FU alone | 17.3 ± 4.9 | 87.3 ± 10.1 | 3.7 ± 0.8 | 23.5 ± 4.0 |
| 5-FU + L-OHP | 17.0 ± 7.8 | 81.7 ± 6.4 | 3.8 ± 0.6 | 24.8 ± 4.8 |

**Conclusions** Using a within patient comparison L-OHP does not influence the PK of 5-FU administered using this schedule. These data confirm our previous finding that, with meticulous sampling and assay techniques, 5-FU PK show only moderate interpatient variability.

### P160 FACTORS INFLUENCING THE PHARMACODYNAMICS OF THE HPMA DOXORUBICIN COPOLYMER, PK1

R Mikaty and PM Loadman, Clinical Oncology Unit, University of Bradford, Bradford BD7 1DP, UK

PK1 is a synthetic N-(2-hydroxypropyl) methacrylamide (HPMA) copolymer-doxorubicin (Dox) conjugate which is currently undergoing Phase II trials in the UK. The activity of PK1 is dependent on endocytosis of the polymer and the release of Dox from the conjugate via thiol dependent proteases. Murine colon tumours MAC15A and MAC26 have previously been shown to have differing responses to PK1 thought to be due in part to differences in enhanced permeability and retention (EPR) and release profile of doxorubicin *in vivo* (Clin. Can. Res., 1999, 5 3682–88). In order to address in more detail the factors which influence the pharmacodynamics of PK1, we investigated both the lysosomal protease expression and the in vitro Dox release from the polymer in a wide range of murine tumours. We also investigated the anti-tumour response in the tumour with the highest protease expression to confirm whether increased protease expression in tumours would lead to increased activity of PK1 *in vivo*.

Dox was measured by an established HPLC assay and the lysosomal cysteine protease activity by combining specific inhibitors with the measurement of naphthylamide cleavage from specific protease substrates. Lysosomes were prepared from tumour homogenates by differential centrifugation.

The rate at which Dox is released from the tumour preparations including MAC 32, 13, 31 26, 15A, 16A and MAC29, varied greatly from 1242 ng/h/mg protein in the MAC 30T to <100 ng/h/mg from the less responsive MAC 26. Lysosomal expression of cathepsin H, L and B varied between tumour type with MAC30T again having the greatest activity of cathepsin H ($830 \pm 10 \times 10^{-5}$ U/mg) cathepsin L ($144 \pm 20 \times 10^{-5}$ U/mg) and cathepsin B ($115 \pm 9 \times 10^{-5}$ U/mg). These data were supported by immunohistochemical observations.

*In vivo* anti-tumour activity against the high cathepsin expressing MAC30T grown in NMRI mice was impressive (6 days growth delay p<0.05, PK1 40 mg/kg iv) when compared to Dox alone (10 mg/kg iv) where no significant growth delay was seen compared to controls (p>0.05). EPR in MAC30T measured by an established Evans Blue assay showed that the EPR effect was not as great as shown previously with the responsive MAC15A (Clin. Can. Res., 1999, 5 3682–88).

We have shown that protease expression is clearly going to influence the pharmacodynamics of PK1 but must be considered in conjunction with the EPR properties of a tumour.

### P161 DESIGN, SYNTHESIS AND DEVELOPMENT OF NOVEL INHIBITORS OF HUMAN DNA-TOPOISOMERASE I

DJ Mincher[a]*, G Kay[a], JEL McDonald[a], A Turnbull[a], MC Bibby[b] and JA Double[b], [a]School of Life Sciences, Napier University Edinburgh EH10 5DT, UK, [b]Clinical Oncology, University of Bradford BD7 1DP, UK

We have shown that anthraquinones conjugated to amino acids via **conformationally-flexible** simple spacer groups such as polymethylenic chains affords a library of novel topoisomerase inhibitors (coded NU:UB) with broad spectrum anti-tumour activity both *in vitro* and *in vivo*. [DJ Mincher, A Turnbull, MC Bibby, JA Double, PS Gilmour, G Lowe, G Kay and ID Hickson, Br. J. Cancer (1999), **80**, 50]. The majority of conjugates bind to DNA by **mixed modal** part intercalative, part minor groove binding mechanisms and show dual inhibitory effects against topo I and topo II α and β isoforms. Towards defining the structural requirements for topo I vs. topo II selectivity, we now show that the conformation of the spacer group can act as a 'molecular switch' between topo I and II. For example, the **conformationally unrestrained** butyl-spaced L-Phe conjugate NU:UB 77 is a catalytic inhibitor of topo II (inhibition of pBR 322 plasmid DNA-relaxation complete at 25 μM and 50 μM, α and β isoforms respectively) but does not stimulate enzyme mediated DNA cleavage. In stark contrast the **conformationally restricted** L-Phe analogue NU:UB 150 containing a rigid 1,4 diaminocyclohexane spacer, does not inhibit either topo II or topo I mediated DNA relaxation. NU:UB 150 does, however, stimulate topo I mediated **cleavage** of DNA (45% nicked plasmid DNA at 25 μM) in a manner analogous to camptothecin. We conclude that the switch from topo II to topo I observed between NU:UB 77 and NU:UB 150 is directly related to the concomitant switch between principally intercalative binding ($QE_{50}$ 2.5 μM; $QH_{50}$ 4.8 μM) shown by NU:UB 77, and the principally groove binding ($QE_{50}$ 6.5 μM; $QH_{50}$ 2.32 μM) mechanisim observed for NU:UB 150 (as shown by fluorescence quenching experiments). The modulation of the DNA binding mode by design of appropiate conformationally restricted spacers, which direct the ligand to the minor groove, favours the design of new topo I inhibitors. NU:UB 150 has shown notable *in vitro* activity against the highly refractory MAC15A murine adenocarcinoma cell line [$IC_{50}$ 3.5 μM (camptothecin 2.0 μM)]. The pattern of cell kill shown by a series of analogues of the prototype topo I inhibitor NU:UB 150 is correlated to the expression of the enzyme and formation of cleavable complexes in the MAC15A cell line. The data is valuable for the design of **clean** inhibitors of topo I which do not suffer from disadvantages of the structural lability of the camptothecin family.

### P162 NOVEL DUAL TOPOISOMERASE (I AND II) INHIBITORS DISPLAYING HIGH SELECTIVITY IN COLON CARCINOMA

DJ Mincher[a]*, A Turnbull[a], S Pettersson[a] MC Bibby[b] and JA Double[b], [a]School of Life Sciences, Napier University Edinburgh EH10 5DT, UK, [b]Clinical Oncology, University of Bradford BD7 IDP, UK

We have shown that spacer-linked anthraquinone-amino acid/peptide conjugates exhibit a spectrum of inhibitory activity against DNA-topoisomerase enzymes *in vitro* and broad-spectrum cytotoxicity in panels of human and animal tumour cell lines. [International Patent No. WO: 99/65886 publ. 23 Dec 1999]

The (propyl-spaced) L-proline conjugate NU:UB 31 and the (butyl-spaced) L-proline conjugate NU:UB 43 have emerged as ***dual*** inhibitors of human DNA topoisomerase I *and* the individual isoforms (α and β) of topoisomerase II. Their *dual* inhibiting activity may allow circumvention of drug resistance mechanisms associated with changes in expression of one of the proteins. The pattern of enzyme inhibition is complex because the conjugates exhibit characteristics of both pure catalytic inhibitors and topoisomerase poisons. NU:UB 31 and 43 each inhibited topo I-mediated relaxation of plasmid pBR322 DNA ($IC_{50}$ 50 μM) and topo II-mediated DNA-relaxation [$IC_{50}$ 10 μM (α-isoform); $IC_{50}$ 25 μM (β-isoform)]. The conjugates are also able to stimulate topoisomerase-mediated **cleavage** of plasmid DNA for example, NU:UB 31 (at 10 μM) produced 62% nicked DNA compared to camptothecin which gave 90% cleavage at 10 μM concentration. Unlike camptothecin, however, both NU:UB 31 and 43 antagonised the cleavage reaction at higher concentrations. Greater levels of DNA cleavage were observed with topo I than either topo IIα or IIβ at equivalent drug concentrations.

*In Vitro* chemosensitivity data from a broad range of tumour cell lines has now indicated **high selectivity** for **colon cancer** in which topo I levels are commonly over-expressed. NU:UB 31 (typically had $IC_{50}$ values in the 3–5 μM range and NU:UB 43, containing the longer spacer, had $IC_{50}$ values between 1 and 4 μM against a panel which include COLO 205; HCC-2998; HCT-116; HCT-15; HT29; KM12; SW-620. Intrinsic topo I levels in this panel varied by no more than 2-fold but overall log-linear correlations between NU:UB-induced cleavable complexes and growth inhibition indicate the importance of cleavable complexes and complex stabilisation in the mechanism of cell kill in this panel of cell lines. The pattern of cell kill, and topo-mediated DNA cleavage are correlated to the 3-dimensional structures of NU:UB 31 and NU:UB 43 and to the p53 status of the cell lines.

### P163 *IN VITRO* ACTIVITY OF XR5944, A DUAL TOPOISOMERASE I AND II INHIBITOR

B Kofler[1], W Dangerfield[1], L Smith[1], AJ Stewart[1], S Okiji[1], BC Baguley[2], WA Denny[2], P Charlton[1], [1]Xenova Ltd, Slough SL1 4EF, UK, [2]Auckland Cancer Society Research Centre, University of Auckland, NZ

Topoisomerases are enzymes involved in the resolution of topological problems arising during the processes of DNA replication and recombination. Topoisomerase I (Topo I) cleaves single stranded DNA whilst topoisomerase II (Topo II) cleaves double stranded DNA. Both enzymes are thus essential for the viability of all eukaryotic cells.

Inhibition of topoisomerases is a mechanism by which many established anticancer agents function. For example camptothecins and etoposide target Topo I and Topo II respectively. However the efficacy of compounds, which only target a single enzyme, is often impaired due to the development of resistance resulting from alterations of expression levels of that enzyme. Several agents have been recently reported which are dual inhibitors of Topo I and Topo II (DACA/XR5000 (acridine carboxamide) and TAS-103 (quinoline derivative)) and are proposed to avoid this atypical drug resistance. Here we describe the *in vitro* properties of a novel bisphenazine, XR5944 which is also a dual topoisomerase inhibitor.

XR5944 was shown to be an exceptionally potent cytotoxic agent in a range of tumour cell lines of various origin (lung, colon, breast, ovarian, leukaemic). $IC_{50}$S for XR5944 were typically sub-nanomolar making XR5944 considerably more potent than either XR5000 or TAS-103 and indeed superior to many current clinical agents (Doxorubicin, Topotecan, Paclitaxel). Studies with cell lines displaying atypical drug resistance revealed that XR5944 retained the activity seen in the corresponding parental cell line hence avoiding this mechanism of resistance. XR5944 also retained significant potency in P-glycoprotein (P-gp) and Multidrug Resistant Protein (MRP) expressing cell lines.

Purified enzyme assays, designed to look at formation of topo-dependant stabilised cleavable complexes, confirmed that XR5944 poisoned both Topo I and Topo II although XR5944 may have a preference for Topo I. Slight differences in cleavage patterns to TAS-103 and XR5000 indicated that there may be subtle differences in mechanism of action between the three stated molecules. However as with the cytotoxicity assays XR5944 appeared to be more potent than XR5000 or TAS-103 and the action of XR5944 on both Topo I and Topo II may be due to its ability to intercalate DNA as shown by DNA unwinding assays.

In summary, the combination of potency and dual activity of XR5944 makes this molecule a promising candidate for anticancer treatment.

### P164 THE *IN VIVO* ANTITUMOUR ACTIVITY OF XR5944, A NOVEL, POTENT TOPOISOMERASE INHIBITOR

H Lancashire, DE Bootle, MB Baker, P Mistry, C Liddle, W Dangerfield, WA Denny[1], PA Charlton, Xenova Ltd, Slough SL1 4EF, UK, [1]Auckland Cancer Society Research Centre, University of Auckland, NZ

Inhibitors of topoisomerase are widely used in the treatment of cancer. Such treatments include inhibitors of topoisomerase I (camptothecin analogues), or topoisomerase II (etoposide and doxorubicin). Recently, several compounds have been demonstrated to have activity against both topoisomerase I and II (e.g. XR5000, intoplicine and TAS-103). This approach has the advantage of avoiding drug resistance due to alterations in cellular levels of either of the two enzymes, and may also allow the drug to act at several points in the cell cycle. We report here a novel bis-phenazine, XR5944, which is also an inhibitor of both topoisomerase I & II. *In vitro*, XR5944 demonstrated exceptional activity when tested against a panel of human cell lines, and exhibited greater potency than several other topoisomerase inhibitors (e.g., topotecan, doxorubicin, etoposide and TAS-103). In addition, XR5944 was unaffected by atypical drug resistance, and retained potent activity in cell lines overexpressing P-glycoprotein (P-gp) or Multidrug Resistance Protein (MRP). In the present study, we have evaluated the *in vivo* activity of XR5944.

In a hollow fibre model, XR5944 was active against a panel of human tumour cell lines. In this model, where cells are grown in 'cell max' fibres which are implanted subcutaneously or intraperitoneally into female CD1 nude mice, XR5944 (5 mg/kg i.v. qdx5) evoked almost complete inhibition (77–94%) of proliferation against three human cell lines (H69 SCLC, HT29 colon and HCT-15 colon). In comparison, TAS-103 induced a 13–66% inhibition at its MTD, using the same schedule.

This exceptional activity of XR5944 translated well to solid human tumour xenograft models. Treatment of female CD1 nude mice bearing H69 SCLC xenografts with XR9544 (5 mg/kg iv. q4dx3) caused tumour stasis for >20 days, whilst 10–15 mg/kg i.v. q4dx3 induced complete tumour regression. In contrast, topotecan (20 mg/kg i.v. q4dx3) only slowed the tumour growth rate compared with control animals. In the resistant HT29 colon carcinoma model, XR5944 (15 mg/kg i.v. q4dx3) induced tumour regression, whereas TAS-103, dosed at its MTD, (45 mg/kg i.v. q7dx3) only induced a delay in tumour growth, compared with control tumours. In all studies, XR5944 was well tolerated at all the efficacious doses and schedules used.

These data show that XR5944 exhibits exceptional antitumour activity, and good tolerance at efficacious doses, making it a promising compound for further development.

### P165 MODULATION OF TOPOISOMERASE IIα EXPRESSION AT CONFLUENCE BY MINOR GROOVE BINDERS

B Tolner, JA Hartley and D Hochhauser, Royal Free and University College Medical School, University College London, 91 Riding House Street, London W1P 8BT, UK

This study aims to understand processes involved in cellular alteration of topoisomerase IIα (top2α) gene expression at confluence and how this is altered in tumour cells. The expression of top2α is regulated by cellular proliferation with transcriptional downregulation in confluent cells. Confluence induced downregulation is modulated through the inverted CCAAT box 2 (ICB2) at position –108 to –104 (J. Biol. Chem. 271: 16741–7, 1996) and may increase resistance to poisons such as etoposide. We examined the DNA-protein interactions within the top2α promoter in exponential and confluent phase NIH3T3 cells. Using electrophoretic mobility shift assay and *in vitro* DN*ase* I footprint experiments, ICB2 was shown to bind the transcription factor NF-Y in nuclear extracts from cells growing in both exponential and confluent conditions. However, a distinct difference in antibody supershift (NF-YA and B) patterns and ICB2 footprinting was observed when comparing extracts from exponential and confluent phase cells. This suggests involvement of an additional factor (to NF-YA and B) which is expressed at confluence and results in top2α downregulation via ICB2. The DNA minor groove binding agents distamycin, Hoechst 33342 and Hoechst 33258 were demonstrated to occupy regions surrounding the inverted CCAAT box in the top2α promoter and displaced proteins binding to this box when incubated with cell extracts. Addition of both Hoechst 33342 and Hoechst 33258 to NIH3T3 cells at confluence resulted in increased expression of top2α. Therefore, DNA binding drugs which block transcription factor activation of the promoter may deregulate top2α and this strategy may be of value in modifying gene expression and modulating chemosensitivity.

### P166 ACTIVATION OF CB 1954 BY NQO2 – A NOVEL AND DIRECT ANTI-TUMOUR THERAPY

Richard J Knox*, Roger G Melton, Surinda K Sharma and Philip J Burke, Enzacta Group Plc, Porton Down Science Park, Salisbury SP4 OJQ, UK

A novel prodrug activation system has been discovered that is endogenous in human tumor cells. A latent enzyme-prodrug system is switched on by an engineered non-biogenic, small molecule co-substrate. This ternary system is inactive if any one of the components is absent. CB 1954 [5-(aziridin-l-yl)-2,4-dinitrobenzamide] is an anti-tumor prodrug that is activated in certain rat tumors via its 4-hydroxylamine derivative to a potent difunctional alkylating agent. However, human tumor cells are resistant to CB 1954 because they are unable to catalyze this bioactivation efficiently. A human enzyme has been discovered that can activate CB 1954 readily and shown it to be commonly present in human tumor cells. The enzyme is NQO2 (NAD(P)H quinone oxidoreductase 2) but its activity is normally latent and a non-biogenic co-substrate such as NRH [nicotinamide riboside (reduced)] is required for enzymatic activity. There is a very large (100–3000-fold) increase in CB 1954 cytotoxicity towards either NQO2-transfected rodent or non-transfected human tumor cell lines in the presence of NRH. A large anti-tumour effect is observed against human tumour xenografts treated with CB 1954 and NRH *in vivo*. There was no significant anti-tumour effect when these agents were administered separately.

Other reduced pyridinium compounds can also act as co-substrates for NQO2. In particular, l-carbamoylmethyl-3-carbamoyl-1,4-dihydropyridine was shown to be a co-substrate for NQO2 with greater stability than NRH, with the ability to enter cells and potentiate the cytotoxicity of CB 1954. *In vivo*, the combination of this compound with CB 1954 produced an even greater anti-tumour effect than that achieved with CB 1954 and NRH. Further, this agent is synthetically accessible and suitable for further pharmaceutical development.

NQO2 is a novel target for prodrug therapy and has a unique activation mechanism that relies on a synthetic co-substrate to activate an apparently latent enzyme. Our findings may reopen the use of CB 1954 for the direct therapy of human malignant disease.

### P167 A NOVEL NITROREDUCTASE FOR CB 1954 ACTIVATION IN CANCER GENE THERAPY

GM Anlezark[1], T Vaughan[2], NP Michael[3], NP Minton[1], H Murdoch[1], MA Sims[1], E Fashola-Stone[1], S Wigley[1], [1]Centre for Applied Microbiology and Research, Porton Down, Salisbury SP4 OJG, [2]Dept of Biology, Univ of York YO10 5YW; [3]Nycomed-Amersham, Cardiff Laboratories CF4 7YT, UK

A novel nitroreductase that can activate the prodrug CB 1954 has been isolated from a *Bacillus* species by ammonium sulphate precipitation and ion exchange chromatography. The N-terminal protein sequence identified the encoding gene as a homologue of a hypothetical NAD(P)H oxidoreductase of *B. subtilis*. The gene was obtained as a PCR product by reverse genetics, cloned and the entire nucleotide sequence determined. The recombinant protein was overexpressed in an *E. coli* host, and its properties compared with those of the previously known *E. coli* B nitroreductase (NTR) and Walker DT-diaphorase (DTD) both of which activate CB 1954. The new protein reduced the prodrug CB 1954, and HPLC analysis of the products demonstrated that only the cytotoxic 4-hydroxylamine derivative was formed. In this property it more closely resembles DTD than NTR. Its affinity for CB 1954 and turnover of the substrate were higher than that of NTR or DTD. In common with both DTD and NTR it could use the quinone, menadione as electron acceptor and its activity with this substrate was potently inhibited by dicumarol. However, in contrast to the other enzymes it showed a marked preference for NADPH as cofactor and therefore could not be classified as a DT-diaphorase (EC 1.6.99.2). It also used the flavin FMN as acceptor with high affinity, and FAD, although with a four fold lower affinity. It is a flavoprotein with a monomeric molecular mass of 21.5 kDa by calculation from the deduced protein sequence and SDS-PAGE. In *in vitro* cytotoxicity tests an enhanced cytotoxicity of CB 1954 was demonstrated when V79 cells were incubated with prodrug, NADPH and enzyme. The improved properties of this enzyme compared with those of the previously known NTR and DTD indicate that it may overcome their limitations for applications in cancer gene therapy.

### P168 SYNTHESIS AND ANTI-CANCER ACTIVITY OF DT-DIAPHORASE ACTIVATED NAPHTHOQUINONE PRO-DRUGS AND COMPARISON WITH EO9

G Steans[1,2], PM Loadman[1], DJ Maitland[2] and RM Phillips[1], [1]Clinical Oncology Unit; [2]Chemistry and Forensic Science, University of Bradford, Bradford BD7 1DP, UK

The indoloquinone EO9 was developed as a potential DT-diaphorase (DTD, NAD(P)H:Quinone oxidoreductase, EC 1.6.99.2) directed bioreductive anti-cancer drug. EO9 was selected for clinical trial due to its unique mode of action, selectivity and potency, both *in vitro* and *in vivo*. 3 partial remissions were seen in Phase I trials, but these results were not replicated in Phase II. The failure in the clinic is thought to be due to inadequate drug delivery to the tumour as a result of rapid pharmacokinetic elimination in conjunction with poor penetration through multicell layers (Phillips *et al.*, 1998, Br J Cancer 77; 2112). The aims of this project were to develop a drug with similar properties to EO9 in terms of bioreductive activation by DTD but with improved pharmacological characteristics in terms of drug delivery. From a series of 12 substituted naphthoquinones, 3 were identified as having comparable biochemical and cytotoxicity profiles to EO9. These were the compounds GS2 (2-aziridinyl-5-hydroxy-1, 4-naphthoquinone),GS3 (2,3-diaziridinyl-5-hydroxy-1,4-naphthoquinone) and GS12 (2-aziridinyl-6-hydroxymethyl-1,4-naphthoquinone). GS3 was comparable to EO9 in terms of substrate specificity for purified human DTD (13.02 ± 2.51 and 19.9 ± 0.5 μmol/min/mg respectively, whilst GS2 and GS12 were considerably better substrates (208.9 ± 5.9 and 199.8 ± 6.0 μmol/min/mg respectively) for DTD than EO9. Sensitivity to 3 DTD rich cell lines (H460, A549, HT29) and the DTD deficient cell line (BE) was determined following a two hour drug exposure using the MTT assay. Selectivity ratios ($IC_{50}$ BE/$IC_{50}$ H460) were 250 (GS2), 231 (EO9), 149 (GS3) and 71 (GS12). Similar results were seen with the remaining 2 cell lines. Pharmacological properties were determined by assessing the stability of compounds in murine whole blood at 37°C *in vitro* by HPLC analysis. EO9 has a half life of 9 ± 4 min. in whole blood suggesting that the rapid elimination of EO9 *in vivo* is due in part to metabolism by the blood. GS3, GS12 and GS2 have half lives in murine whole blood of 589 ± 36, 55 ± 1 and 31 ± 2 min respectively. These results suggest that GS3 in particular may have improved pharmacological properties in terms of decreased pharmacokinetic elimination rates *in vivo* over EO9. In addition, GS3 retains the desirable properties of EO9 with regards to DTD activation and selectivity for DTD rich cells. Further studies are required to determine whether or not these results translate into improved anti-tumour activity *in vivo* compared with EO9.

### P169 A PHASE I DOSE ESCALATION TRIAL AND PHARMACO KINETICS OF THE GDEPT PRODRUG CB1954

C Palmer, G Chung-Faye, R Barton*, D Ferry, J Clark, J Baddeley, D Anderson, L Seymour, DJ Kerr, CRC Institute for Cancer Studies, University of Birmingham, Edgbaston, Birmingham B15 2TA, UK

CB1954 (5-(aziridin-l-yl)-2,4-dinitrobenzamide) is a substrate for the bacterial enzyme nitroreductase which converts it into a potent bifunctional alkylating agent. CB1954 is therefore a candidate prodrug in Gene Directed Enzyme Prodrug Therapy (GDEPT) protocols.

CB1954 was administered by bolus intravenous injection on a three weekly cycle. 26 patients (age range 23–78, median age 62) received between one and six doses of drug. 19/26 (73%) patients were male. 13/26 (50%) of cancers were colorectal, 3/26 (12%) gastric, 12% oesophageal and 12% mesothelioma. The first dose level explored was 3 mg/m$^2$, no significant toxicity was seen until the fifth dose level of 24 mg/m$^2$.

Toxicity of intravenous CB1954

| Dose level (mg/m$^2$) | Number of patients | Toxicity |
|---|---|---|
| 24 | 4 | Diarrhoea (G2) |
| 30 | 5 | ALT/AST (G3) |
| 37.5 | 3 | Diarrhoea (G4)<br>Dehydration (G4)<br>ALT/AST (G2) |

The maximum tolerated dose (MTD) was 37.5 mg/m$^2$ with gastrointestinal and hepatic dose limiting toxicity. No alopecia, haematological toxicity or nephrotoxicity was seen. Pharmacokinetics of CB1954 indicated a linear relationship between dose and area under the data (AUD) for 3 to 24 mg/m$^2$ but there appeared to be a non-linear effect at the higher doses. The median elimination half-life was 51 minutes and less than 5% of the drug appeared unchanged in the urine. From *in vitro* studies of cells expressing nitroreductase, the $IC_{50}$ of CB1954 is between 0.1 and 10 μM. At a dose of 30 mg/m$^2$, the serum levels were greater than 1 μM for 2 hours and greater than 0.1 μM for more than 4 hours. One patient with colorectal cancer had a fall in carcino-embryonic antigen (CEA).

In conclusion, CB1954 is a well tolerated prodrug for the GDEPT approach. We are now conducting phase I trials of IV CB1954 plus virally delivered nitroreductase in patients with liver metastases.

### P170 *IN VITRO* & *IN VIVO* STABILITY OF PURIFIED MFE 23::CPG2 FUSION PROTEIN FROM *PICHIA PASTORIS*

J Bhatia, SK Sharma; BR Pedley; G Boxer; L Robson; RHJ Begent and K Chester, CRC Targeting and Imaging Group, Dept of Oncology, RFUCMS, UCL, Royal Free Campus, London NW3 2PF, UK

The efficiency of antibody directed enzyme prodrug therapy (ADEPT) has been demonstrated in animal studies and in clinical trails[1]. A new approach to achieving ADEPT involves the use of recombinant MFE-23::CPG2 fusion protein expressed in *Pichia pastoris*. MFE-23 is a phage-derived single chain Fv antibody reactive with carcinoembryonic antigen (CEA)[2]. CPG2 is carboxypeptidase G2, an enzyme that cleaves glutamate to activate a prodrug. MFE-23::CPG2 was initially expressed in *E. coli*, purified using CEA-affinity chromatography and shown to specifically localise to a tumour with favourable tumour to normal tissue ratios after 48 hr of injection; these results showed significant improvement over those obtained with a chemical conjugate of CPG2 with anti-CEA[3]. However, *E.coli*-expressed MFE-23::CPG2 gave low yields (0.5–1.0 mg/ml). To obtain greater yields and improve the ease of purification, MFE-23::CPG2 with a hexahistidine tag was expressed from the yeast *Pichia pastoris*. This resulted in a 100-fold increase in yield using purification with immobilised metal affinity chromatography. Specific enzyme activity of the fusion protein was 81 U/mg. Immunoreactivity to CEA was confirmed by ELISA and the presence of glycosylation by lectin binding. *In vitro* stability was assessed by SDS-PAGE, western blotting, enzyme activity, size exclusion and ELISA. These showed that MFE-23::CPG2 was stable when stored at –80°C for more than a year. *In vivo* stability was determined by sub-millimetre biodistribution of $^{125}$I-labelled MFE-23::CPG2 in nude mice bearing colorectal tumour xenografts. This revealed that the fusion protein penetrated well into the tumour mass and selectively targeted viable regions as early as 4 hr after injection. Furthermore, using $^{125}$I-labelled MFE-23::CPG2, a novel approach was developed to investigate the *in vivo* stability in LS174T xenografts. This involved homogenising the isolated xenograft and separating the proteins by SDS-PAGE. The $^{125}$I-labelled MFE-23::CPG2 within the homogenised tumour mass was visualised using autoradioluminography. This test showed that the radiolabelled product was intact 4 hr after injection. At this time point, in the same animal model, the tumour: plasma ratio of CPG2 enzyme activity obtained with MFE-23::CPG2 was 19:1. The results confirm that *Pichia pastoris* expressed MFE-23::CPG2 is stable in *in vivo* human colorectal xenograft models. The fusion protein is at present being prepared for a phase I clinical trial.

Research funded by the Cancer Research Campaign and the Ronald Raven Chair in Oncology Trust.

1 Napier et al. (2000) *Clinical Cancer Research* **6**: (2000)
2 Chester et al. (1994) *Lancet* **343**: 455–456
3 Bhatia et al. (2000) *Int J Cancer* **85**: 571–577

### P171 MUTATING ADEPT ENZYME CPG2 TO REDUCE ITS IMMUNOGENICITY

DIR Spencer[1]*, D Purdy[2], N Minton[2] NR Whitelegg[3], AR Rees[3], RHJ Begent[1] & KA Chester[1], [1]CRC Targeting & Imaging Grp., Dept Oncology, RFUCMS, UCL, London NW3 2PF, [2]CAMR, Salisbury SP4 OJG, [3]Dept. Biol. Biochem, Bath Univ., Bath BA2 2AY, UK

**Background** Carboxypeptidase $G_2$ ($CPG_2$) is an enzyme derived from *Pseudomonas aeruginosa* that natively cleaves c-terminal glutamic acids from peptides. No equivalent exists in humans and hence this enzyme has been used successfully for antibody directed enzyme prodrug therapy (ADEPT) in the treatment of drug resistant epithelial carcinomas. These express novel tumour markers that can be targeted selectively with antibodies. However, $CPG_2$ is immunogenic in humans. Previously a phage display library representing the immune response of mice immunised with $CPG_2$ was used to epitope map a region of $CPG_2$ that is immunogenic in humans. Two antibody single chain fragments (scFvs) bound to this region, CM79 and CM12. Using surface enhanced laser desorption ionisation affinity mass spectrometry (SELDI-AMS) this region of $CPG_2$ was defined as incorporating the c-terminus and two loops from sequentially remote sequences bound to both CM79 (c-terminus and one loop) and CM12 (c-terminus and both loops). Mutating this region to epitopes no longer recognised by CM79 and CM12 may result in $CPG_2$ variants with decreased immunogenicity.

**Results** Using the epitope information from CM79 and CM12 binding to $CPG_2$ we have produced, using recombinant technology, a series of $CPG_2$ mutants where each amino acid of a selected epitope region was in-turn replaced with alanine. Two variants were produced with an epitope region replaced with sequences based on human peptides modelled to have similar conformation to the native sequence. These $CPG_2$ mutant variants were tested for enzyme activity by a spectrophotometric assay and for CM79 and CM12 antibody binding by ELISA. Out of 15 $CPG_2$ variants tested one of the alanine mutations where arginine 162 was replaced with an alanine resulted in a $CPG_2$ variant that completely lost CM79 binding activity and but still retained some CM12 binding activity. The $CPG_2$ variant retained enzyme activity. This new variant of $CPG_2$ is the first to prove that immunogenic sites of this clinically important enzyme can be removed without significantly losing enzyme activity.

**Discussion** Modifying the principle immunogenic sites on $CPG_2$ may reduce overall immunogenicity but also gives potential to have alternative versions of the enzyme which can be given to patients who have developed an immune response to the original $CPG_2$.

This work is supported by the Cancer Research Campaign and The Ronald Raven Chair in Oncology Trust.

### P172 *IN VIVO* CHARACTERISTICS OF AN ENGINEERED FUSION PROTEIN FOR USE IN ADEPT

SK Sharma*[1], RB Pedley[1], J Bhatia[1], N Minton[2], KA Chester[1] and RHJ Begent[1], [1]CRC Targeting and Imaging Group, Royal Free and UCL Medical School, London NW3 2PF, [2]CAMR, Porton Down, Salisbury, Wilts, UK

Antibody directed enzyme prodrug therapy (ADEPT) has shown feasibility as a treatment for cancer. ADEPT has the potential to generate high concentrations of cytotoxic agent selectively at tumour sites. To realise this potential, the targeted enzyme must be present in high concentrations at the tumour sites and be rapidly cleared from blood and normal tissues before prodrug administration. To achieve this, a biologically active recombinant fusion protein, comprising an anti-CEA scFv MFE-23) antibody fused to an enzyme carboxypeptidase G2 (CPG2), has been constructed for use in the next ADEPT system. This fusion protein has been expressed in *Pichia pastoris* and is stable *in vitro* and *in vivo*. Biodistribution studies of enzyme activity measurements were performed in nude mice bearing the human colon carcinoma xenograft LS174T, using an hplc assay. MFE::23-CPG2 fusion protein (1000 units/kg) was given intravenously into the tail vein, and showed localisation in tumours at earlier time points than was observed with the A5B7-F(ab′)$_2$-CPG2 conjugate. MFE-23::CPG2 cleared rapidly from plasma within 6 hours after injection (0.0052+/–0.001 units/ml plasma) but enzyme activity persisted in tumours (1.3 +/–0.2 units/g tumour at 6 hrs), resulting in a tumour to plasma ratio of 250:1. Tumour to liver, kidney, lung and spleen ratios were 254, 245, 158 and 160 respectively. This allowed prodrug to be given at 6 hours after fusion protein injection, resulting in significant growth delay of the tumour with no toxicity in mice.

The rapid clearance from blood and normal tissues combined with selective enzyme retention in tumours observed with MFE-23::CPG2 fusion protein gives the potential to deliver multiple cycles of ADEPT for greater clinical efficacy before development of immune response to CPG2.

Supported by the Cancer Research Campaign.

### P173 INFLUENCE OF TUMOUR CELL RESPONSE AND NUCLIDE SELECTION ON THE EFFICACY OF RIT

JLJ Dearling, AA Flynn, C Pagoulatos, GD Wilson, M Woodcock, E Denholm, J Hartley, RHJ Begent and RB Pedley, CRC Targeting and Imaging Lab, Dept of Oncology, RFUCMS, Royal Free Campus, Hampstead, London NW3 2PF, UK

**Introduction** Radioimmunotherapy (RIT) uses antibodies, labeled with a radionuclide, to deliver a therapeutic dose of radiation selectively to cancer deposits. Treatment efficiency depends on a number of factors but is ultimately determined by the number of surviving cells. In this study we used a mathematically defined parameter, the biologically effective dose (BED), to relate the number of surviving cells to absorbed dose and its pattern of delivery. The dose delivered to the tumour was calculated for a range of radionuclides ($^{90}$Y, $^{131}$I, $^{32}$P, $^{186}$Re, $^{188}$Re, $^{64}$Cu) linked to a tumour specific anti-CEA IgG.

**Methods** The response of three human colorectal cancer cell lines (SW1222, LOVO and LS174T) was characterised by the *in vitro* measurement of radiosensitivity, rate of repair and proliferation using clonogenic assay, comet assay and flow cytometry respectively. True dose was calculated for each cell type and radionuclide and was standardised to a typical dose received by bone marrow during radiotherapy (4 Gy).

**Results** The results are summarised in the table below.

| | | SW1222 | LOVO | LS174T |
|---|---|---|---|---|
| Radiosensitivity (α/β (Gy)) | | 310.48 | 14.68 | 12.26 |
| Repair half life (minutes) | | 8.32 | 16.77 | 4.13 |
| Rate of proliferation ($T_{pot}$ (hours)) | | 12.96 | 10.44 | 11.90 |
| BED (Gy) | $^{32}$P | 20.86 | 10.78 | 10.91 |
| | $^{64}$Cu | 5.84 | 3.74 | 3.74 |

The SW1222 cells were found to be considerably more radiosensitive than the other two lines. Repair half-lives were within the normal range (5–55 minutes) and the rates of proliferation of the three lines were similar. The highest BED was for $^{32}$P and the lowest was for $^{64}$Cu. Nuclides with shorter half-lives tended to have a higher relative effectiveness (RE) but deliver their dose over a shorter period of time, their net therapeutic effect being less than that of the longer lived nuclides.

**Discussion and Conclusions** The mathematical model predicted that SW1222 was the most responsive cell line to RIT, was confirmed in comparative therapy experiments *in vivo*. It also showed that radionuclides with longer half-lives were the most therapeutically effective. The model can now be used to optimize RIT by matching radionuclides with antibodies and tumour type.

### P174 RADIOLABELLING OF CAMPATH-1H MONOCLONAL ANTIBODY (Mab) WITH $^{111}$INDIUM USING DOTA-MALEIMIDE AS THE CHELATING AGENT

P Hadjiyiannakis*[1], G Hale[2], A Hall[1], G Harden[1], R Clutterbuck[1], R Brooks[1], VR McCready[1], A Horwich[1], MJS Dyer[1], 1: The Institute of Cancer Research and 2:The Therapeutic Antibody Centre, Oxford, UK

**Background** The therapeutic unconjugated humanised IgG1 CD52 Mab, CAMPATH-1H has reduced efficacy against lymphomatous nodes, which can be treated with radioimmunotherapy. A stably radiolabelled Mab, with preserved immunoreactivity, is a prerequisite for such work. $^{111}$Indium ($^{111}$In) is a better imaging agent than $^{131}$Iodine ($^{131}$I). $^{111}$In-Mab is used to predict the biodistribution of Mabs labelled with $^{90}$Yttrium ($^{90}$Y). The latter emits higher energy beta particles than $^{131}$I which potentially allow a more homogeneous dose to be delivered in large masses. Macrocyclic chelating agents such as DOTA (1,4,7,10-tetraazacyclododecane-N,N,N,N tetraacetic acid) have higher stability for both $^{111}$In and $^{90}$Y than DTPA (diethylene triamine pentaacetic acid).

**Aim** To radiolabel CAMPATH-1H with $^{111}$In for experimental and clinical use.

**Methods** A two step conjugation methodology was employed. Two thiols were incorporated per antibody molecule using N-Succinimidyl 3-(2-pyridyldithio)propionate (SPDP) followed by reduction with dithiothreitol. DOTA-maleimide was incubated with $^{111}$In (in 0.2 M HCl) for a minimum of two and half hours at 47°C using 0.1 M ammonium acetate buffer pH7.4, on a magnetic stirrer/hot plate. The DOTA: $^{111}$In molar ratio was 250:1. (For $^{90}$Y (in 0.05 M HCl) 0.025 M ammonium acetate buffer pH5.4 was used, at 100–250:1 DOTA: $^{90}$Y, for 45 mins at 47°C). The $^{111}$In-DOTA-maleimide was reacted with thiolated Mab (molar ratio 2:1 DOTA: Mab) for one hour at room temperature, followed by a 10 fold excess DTPA (in 0.1 M ammonium acetate buffer pH 5.0) strip for 90 minutes at room temperature to remove any loosely bound $^{111}$In. The final purification step was via a Microcon microconcentrator (50 kD cut off). Ascorbic acid (10 mg) in PBS (pH 7.4) was added as a radioprotectant.

**Results** >80% of the $^{111}$In was incorporated by the DOTA-maleimide. Significant differences ($p<0.05$) were seen by varying DOTA: $^{111}$In molar ratios, length of incubation, and temperature of incubation. The DOTA-maleimide retained its reactivity for thiols when incubated for up to 6 hrs at 47°C. Labelling efficiency of the whole procedure was 50% with specific activity of 58 MBq/mg. Protein recovery was 73% for the microconcentrator. The radiochemical purity of the $^{111}$In-Mab was 95% by size exclusion HPLC. Immunoreactivity was >80% as assayed by two affinity HPLC methods. The radiolabelled antibody was stable to DTPA challenge and plasma (both *in-vivo* and *in-vitro*).

**Discussion** Biodistribution of $^{111}$In-CAMPATH-1H was investigated in SCID/Nod mice bearing human lymphoma xenografts. There was a high tumour uptake (15% injected activity per gram tumour) with a favourable therapeutic ratio, once Fc receptors were blocked with normal human immunoglobulin. Human studies are planned.

### P175 COMPLETE REGRESSION OF POST-TRANSPLANT LYMPHOPROLIFERATIVE DISEASE WITH EPSTEIN BARR VIRUS SPECIFIC ALLOGENEIC CYTOTOXIC T CELLS

T Haque[1], C Taylor*[1], GW Wilkie[1], P Murad[1], S Beath[2], P McKiernan[2], DH Crawford[1], [1]Basic & Clinical Virology Group, University of Edinburgh, EH9 1QH, [2]Liver Unit, Birmingham Children's Hospital, B4 6NH, UK

Autologous Epstein-Barr virus (EBV)-specific cytotoxic T lymphocyte (CTL) have recently been used to treat or prevent EBV associated post-transplant lymphoproliferative disease (PTLD) as a form of adoptive immunotherapy. In a UK-wide multicentre trial, we are generating a bank of polyclonal EBV-specific CTL lines from healthy blood donors. As PTLD lesions are aggressive and rapidly growing, these CTLs are ready for infusion if the disease arises. These lines are selected for transplant patients on the basis of best HLA match, and on specific killing of patient's cells in *in vitro* cytotoxicity assays. Here we report the first successful regression of PTLD in a solid organ transplant recipient using partially HLA-matched EBV-specific CTL grown from an unrelated healthy blood donor.

Withdrawal of immunosuppression had failed to treat an EBV positive PTLD in an eighteen month old liver and bowel transplant recipient. One infusion of EBV-specific CTL was given at a dose of $10^6$ cells/kg body weight.

The patient's tumour showed signs of regression within a week. There was no toxicity associated with the infusion and no evidence of graft versus host disease. The EBV load in peripheral blood dropped to undetectable levels within one week. Limiting dilution analysis (LDA) assays showed no CTL precursor (CTLp) activity before the infusion and high numbers of CTLp at 4 hours and 24 hours post CTL infusion. There was a reversal of the CD4/8 ratio in peripheral blood and an increase in the percentage of HLA-DR positive CD8 cells. Allospecific IgM against the donor CTL was detected at 6 weeks after infusion. The patient has been in complete remission for eight months.

Allogeneic CTL infusion to treat more PTLD patients is currently underway. If successful in clinical trials, the use of allogeneic antigen-specific CTLs could become an important addition to tumour therapy.

### P176 DENDRITIC CELLS FOR THE TREATMENT OF PATIENTS WITH RELAPSED METASTATIC BREAST CANCER – A PHASE ONE CLINICAL TRIAL

FE Nussey*[1], I Downing[2], M Waterfall[1], J Campbell[2], N Hunter[2], J Parker[3], J Innes[2], K Samuel[2], Gordon Cook[2], ML Turner[4], RCF Leonard[1], University of Edinburgh, Department of Oncology, Western General Hospital, Crewe Road, Edinburgh EH4 2XU, [2]Scottish National Blood Transfusion Service, Edinburgh, [3] Biomira Inc', Edmonton, Canada, [4]University of Edinburgh, John Hughes Bennett Laboratory, Western General Hospital, Crewe Road, Edinburgh EH4 2XU, UK

Human MUC-1 is a high molecular weight trans-membrane glycoprotein which is expressed on most epithelial cells, but in breast cancer there are alterations in distribution and intensity of expression, as well as changes in carbohydrate structure. Dendritic cells (DC's) are potent antigen processing and presenting cells which, when pulsed with MUC-1 tumour antigens are able to induce MHC restricted antigen specific CD4 and CD8 positive T cells in vitro. DC's are the only antigen presenting cells known to induce and specifically prime naive cytotoxic T lymphocytes.

Since August 1999 DC's have been produced by co-culture, in the presence of GM-CSF and IL4, of monocytes derived from the buffy coat of 6 patients with relapsed metastatic breast cancer (BC). These cells were then pulsed with liposomal MUC-1 ("Biomira Inc") and returned to the patients by subcutaneous injection. Each patient received one or two injections depending on the number of cells produced, but all received a dose equivalent to between 0.07 and $0.13\times10^6$ CD1a positive cells per kilogram. Patients were observed overnight and followed up at 7, 14, 28 days and 3 months following the completion of therapy. Disease status was assessed at entry to the study and at 28 days and 3 months. There was no acute toxicity noted. Fatigue (lasting median 2.5 days) was reported in 4 patients; grade 1 in 3 and grade 2 in 1. There were no grade 3 or 4 toxicities noted. All patients have now completed follow up of minimum 28 days and maximum 3 months. At one month one patient had stable disease after prior progression, two had continuing stable disease and three experienced further progression.

We have demonstrated feasibility and tolerability of the first two dose levels. It is projected that a further 3 patients at each of two dose levels will be entered into the study. In order to escalate the dose of DC's given to these patients monocytes will be collected by a 2–3 hour leucopheresis. Further, larger, clinical studies are requested to assess potential theraputic benefit. Studies of the specific humoral and cellular immune responses in these patients are in progress.

This work is supported by a grant from "The Melville Trust for the Care and Cure of Cancer".

### P177 THE ROLE OF COMPLEMENT IN ELIMINATING TUMOUR CELLS FROM GUINEA PIGS*

KS Kan[1], CY Yu[1] & GT Stevenson[2], [1]School of Biological & Applied Sciences, University of North London, Holloway, London N7 8DB, [2]Tenovus Research Laboratory, Southampton University Hospital, Tremona Road, Southampton SO16 6YD, UK

The role of complement in removing leukaemic cells from the guinea pigs was studied using two different approaches. In the first approach, normal strain 2 and abnormal C3-deficient guinea pigs were employed. In this study, $0.2 \times 10^5$ $L_2C$ leukaemic B lymphocytes were inoculated into two groups of ten strain 2 and ten C3-deficient animals. Twenty four hours after the inoculation, they were injected intravenously with 1 mg of anti-idiotypic mouse/human chimeric $FabFc_2$ antibody in which the $Fc\gamma$ is of human origin. The profiles of survival were found to be similar between the two groups and their life-span was prolonged by an average of 14 days as compared with the control. In the second approach, the disulphide bonds at the hinge of the $FabFc_2$ antibody were disrupted by DTT reduction followed by alkylation with N-ethylmaleimide. This procedure rendered the $Fc\gamma$ incapable of activating complement. The inability of the open-hinge $FabFc_2$ antibody to activate complement lysis was confirmed by the $^{51}Cr$ release assay on the $L_2$ C cells. Twenty four hours after inoculating the $L_2C$ cells into two groups of ten leukaemic strain 2 guinea pigs, the animals were injected intravenously with 1 mg of either closed-hinge or open-hinge $FabFc_2$ antibody. The profiles of survival were also found to be comparable between the two groups and their life-span was prolonged by an average of 7 days. These observations indicate that complement does not play a significant role in eliminating leukaemic cells in guinea pigs.

### P178 THE RELATIONSHIP BETWEEN LYMPHOCYTES OF THE CD103 PHENOTYPE AND E-CADHERIN EXPRESSING BLADDER CANCERS

*J Cresswell, H Robertson, JA Kirby and DE Neal, Dept of Surgery, The Medical School, Newcastle University, Newcastle upon Tyne, UK

**Background** CD103 is a marker of the $\alpha$ subunit of the intra-epithelial lymphocyte integrin $\alpha_E\beta_7$; the principle ligand for this molecule is the epithelial adhesion molecule E-cadherin. Expressed by most intra-epithelial lymphocytes of the gut, to our knowledge this is the first report of CD103+ lymphocytes in human bladder.

**Aim** To demonstrate the presence of lymphocytes bearing this integrin in normal and cancerous human bladder and to investigate the relationship with E-cadherin expression.

**Methods** Cryostat sections of normal bladder and Transitional Cell Carcinomas (TCC) were treated with antibodies specific for CD103 and E-cadherin. Visualisation was performed by immunoperoxidase and alkaline phosphatase development. Dual staining was used to assess the relationship between the antigens.

**Results** A few CD103+ lymphocytes were found in normal bladder confined to the epithelium and lamina propria. In 20 TCC sections examined, E-cadherin expression varied in accordance with the histological grade. CD103+ lymphocytes were found predominantly in the stroma surrounding the tumours, although some cells were noted to be infiltrating the periphery of tumour islands. Dense aggregates of E-cadherin positive tumour cells were shown to be minimally infiltrated by CD103+ cells.

**Conclusion** Lymphocytes of the CD103+ phenotype were observed in normal bladder and cancer specimens; however, aggregation of these cells was predominantly observed in the stroma surrounding tumour cells. It is possible that immune evasion strategies utilised by the tumour cells prevent infiltration by CD103+ T cells. Alternatively, the dense homotypic adhesion between E-cadherin positive tumour cells may mask the epitope required for CD103 interaction. Further studies are underway to examine the functional significance of this pattern of antigen distribution.

### P179 EXPRESSION OF THE NOVEL PRO-INFLAMMATORY POLYPEPTIDE EMAP-II IN LUNG TUMOURS: ASSOCIATION WITH INFLAMMATORY INFILTRATES

MPR Tas, L Jones, W Ward, C Clelland[1] and JC Murray*, University of Nottingham Laboratory of Molecular Oncology, CRC Department of Clinical Oncology and [1]Department of Histopathology, City Hospital, Hucknall Road, Nottingham NG5 1PB, UK

The novel pro-inflammatory polypeptide EMAP-II (endothelial monocyte-activating polypeptide-II) modulates the behavior of endothelial cells, monocytes and granulocytes *in vitro*. Injection *in vivo* induces an acute inflammatory response and tumour regression in mice. Biologically active EMAP-II is detected in supernatants of cultured tumor cells; however, little is known about its expression by tumours *in vivo*. We studied EMAP-II distribution and processing in normal human lung and lung tumours, and its relationship to the presence of inflammatory infiltrates. Immunohistochemistry was performed with polyclonal antibodies against recombinant human protein, and immuno-blotting with the same antibodies was used to assess the conversion of EMAP-II to processed forms. Neuroendocrine differentiation was assessed by immunohistochemical staining for N-CAM, and sections were scored for the presence of inflammatory infiltrates. In normal lung only alveolar macrophages were positive for EMAP-II expression. In lung carcinoids, which were devoid of inflammatory infiltrates, tumour cells were strongly positive for EMAP-II, occurring primarily as the 34 kDa cytoplasmic form. In small cell lung cancers (SCLC), all of which contained inflammatory infiltrates, there was weak, diffuse staining of some tumour cells, and stronger staining of tumor stroma. In these tumors, EMAP-II occurred primarily as a 26–28 kDa processed form, normally found in the extracellular compartment. The stroma of adenocarcinomas and squamous cell carcinomas also showed diffuse staining. We conclude that (i) EMAP-II expression and processing in lung tumours depends upon histological type, and (ii) recruitment of inflammatory cells into lung tumours is associated with the presence of processed forms of EMAP-II, and not with expression of EMAP-II *per se*.

*This work is supported by the Cancer Research Campaign*

### P180 PROSTATE ADENOCARCINOMA CELLS RELEASE PRO-INFLAMMATORY POLYPEPTIDE EMAP-II IN RESPONSE TO STRESS

G Barnett, MPR Tas, A-M Jakobsen, K Rice, J Carmichael, and JC Murray*, University of Nottingham Laboratory of Molecular Oncology, CRC Department of Clinical Oncology, City Hospital, Hucknall Road, Nottingham NG5 1PB, UK

Endothelial-monocyte activating polypeptide-II (EMAP-II) is a pleiotropic effector of endothelial cells, monocyte/macrophages and neutrophils, first described in supernatants of murine tumour cells. It induces coagulation, E- and and P-selectin expression, and release of von Willebrand factor from endothelial cells *in vitro*, is chemotactic for neutrophils and monocytes, and induces release of myeloperoxidase activity from neutrophils *in vitro*. Local injection of EMAP-II evokes an acute inflammatory response in the mouse footpad, and haemorrhage, inflammatory infiltration, and regression of tumours. EMAP-II occurs intracellularly as a 34 kDa precursor, which can be proteolytically cleaved and released in an active 20–22 kDa form, by an unknown mechanism. The amino acid sequence at the cleavage site, and the observation that apoptotic cells release EMAP-II, suggests the involvement of a caspase-like enzyme. We have studied the effects of chemical and physiological stresses on the processing and release of EMAP-II by the human prostate adenocarcinoma cell lines LNCaP and DU-145. We first demonstrated by RT-PCR that both cell lines express EMAP-II transcripts. We then used western blotting with polyclonal antibodies raised against recombinant human EMAP-II to detect and characterise the EMAP-II polypeptides. Both cell lines expressed EMAP-II primarily as a cytoplasmic 34 kDa form. The cells were then treated with agents known to induce apoptotic or necrotic cell death, and the relationship between form of cell death, and EMAP-II processing and release was examined. In response to inducers of apoptosis (etoposide, camptothecin, ionomycin, and thapsigargin) EMAP-II was processed to a 26–28 kDa intermediate and released. Etoposide and ionomycin treatment also led to the appearance of the 20–22 kDa form. Antimycin-A and saponin, which induce necrosis, also stimulated release and processing of EMAP-II. Hypoxia (2.5% $O_2$), which induces primarily necrotic cell death in these cell lines, caused the release of biologically active EMAP-II. We conclude that EMAP-II may become biologically available and active in prostate tumours following a variety of stresses. The release of EMAP-II may have implications for the physiology of tumours as well as their response to treatment.

*This work is supported by the Cancer Research Campaign and the Association for International Cancer Research*

### P181 A MODEL TO PREDICT BIOCHEMICAL (PSA) CONTROL AFTER RADICAL RADIOTHERAPY WITH INITIAL ANDROGEN SUPPRESSION FOR CLINICALLY LOCALISED PROSTATE CANCER

DP Dearnaley, C Parker, A Norman, R Huddart, A Horwich, Royal Marsden Hospital and Institute of Cancer Research

**Introduction** Phase III studies have demonstrated the clinical benefit of combining short course neo-adjuvant androgen deprivation (NAD) with radical radiotherapy (RT) for clinically localised prostate cancer. We have developed a nomogram to predict the likelihood of PSA control.

**Patients and Methods** 517 men with histologically proven ca prostate were treated with 3–6 months NAD (LHRHa + 3 weeks cyproterone acetate) and radical prostate radiotherapy (64 Gy in 6#fr1/2> weeks) between 1988–98. Median presenting PSA was 20 ng/ml and 56% of patients had T3/T4 disease. Gleason Scores of 2–4, 5–7, 8–10 were reported in 14%, 66%, and 20% of cases respectively. PSA failure was defined by two consecutive rises in PSA ≥ 2 ng/ml, and the time of failure taken as the date of first PSA value ≥ 2 ng/ml or date of restarting normal treatment. Univariate/multivariate (MV) analysis of pre-treatment factors was performed and a nomogram constructed to predict PSA failure free survival probability.

**Results** After a median follow-up of 35 months, 224 men had developed PSA failure. Clinical T stage, presenting PSA and histological grade were all highly predictive of PSA failure on MV analysis. The nomogram gave the maximum coefficient (PSA>50) the value of 100. The score for an individual patient is given by the summation of T stage (T1,2=0: T3,4=29), PSA (<10 ng/ml=0: 10–19 ng/ml=12: 20–49 ng/ml=37: >50 ng/ml=100) and histology (Gleason 2–4=0: 5–7=42: 8–10=79) values.

| Patient Score | 0 | 50 | 100 | 150 | 200 |
|---|---|---|---|---|---|
| 2 year PSA failure free Survival (%) | 91 | 83 | 69 | 50 | 20 |
| 5 year PSA failure free Survival (%) | 74 | 55 | 31 | 11 | 6 |

**Conclusion** These results are at least equivalent to those reported using surgery or higher doses of RT alone. Simple graphical display of the nomogram can inform both clinician and patient of the likely outcome of treatment.

### P182 A POSSIBLE ROLE FOR INTERMITTENT ANDROGEN SUPPRESSION (IAS) IN THE EARLY MANAGEMENT OF LOCALISED PROSTATE CANCER

D Farrugia*[1], W Ansell[1], F Chinegwundoh[2], G Williams[2], P Wilson[1] and RTD Oliver[1], Departments of Medical Oncology[1] and Urology[2], St Bartholomew's Hospital, London EC1A 7BE, UK

IAS has been shown to be a feasible strategy in the management of advanced prostate cancer. Several phase II studies including over 200 patients (pts) showed that repeated remissions can be induced with successive cycles on and off treatment, thus reducing the duration of side effects from androgen suppression (AS) without compromising the duration of disease control or survival (Gleave M et al. 1998, Prostate Cancer and Prostatic Diseases, 1:289). In localised disease (LD), debate continues over the roles of radical surgery (S) and radiotherapy (RT), as well as the role of early AS versus "watchful waiting". We studied 31 pts in 3 groups with LD (≤T3N0M0) who had either RT followed by observation (group A, n=7), S/RT with concurrent IAS (group B, n=11), or IAS alone as primary therapy (group C, n=13). AS consisted of either LHRH analogue alone (LHRHA) or combined androgen blockade (CAB). Pts stopped AS when PSA had normalised with no clinical evidence of residual disease, and remained off treatment until clinical recurrence or elevation of PSA >20 ng/ml when AS was again introduced. Pts achieving PSA and clinical remission again stopped AS until relapse. Results in the 3 categories are summarised below:

| Patient Characteristic | Treatment Category | | |
|---|---|---|---|
| | A | B | C |
| No. of patients/median age | 7/65 | 11/63 | 13/69 |
| Median ± range baseline PSA (ng/ml) | 18 (14–58) | 58 (1.6–242) | 27 (5.3–166) |
| Type of AS: LHRHA/CAB | – | 6/5 | 5/8 |
| Duration on/off primary AS[1] | – | 12/25 | 9/9 |
| Response: CR/PR/NR/NE | – | 11/0/0/0 | 12/1/0/0 |
| Time to progression (1) | 29 | 37 | 21[2] |
| Relapsed pts starting AS/still on Rx | 7/0 | 7/3 | 9/5 |
| Duration on/off As for relapse | 10/20 | 13/15 | 7/33 |
| Response: CR/PR/NR/NE | 5/2/0/0 | 3/2/0/2 | 6/2/1/0 |
| Time to progression (2) | 33 | 28 | 34[2] |
| Median follow-up (range)/no. dead | 89(31–244)/2 | 59(12–115)/1 | 35(15–134)/1 |

[1]= Durations are median in months; [2]=differences not significant

Thus disease control was equivalent for primary IAS alone (C) and radical RT alone (A), but combined treatment may be superior. Only 33% of time was spent on AS in groups B and C. Whether combined IAS + RT up front is superior to IAS alone with delayed RT is under investigation in a prospective study.

### P183 EVALUATION OF THE OPTIMAL COPLANAR FIELD ARRANGEMENT FOR USE IN THE BOOST PHASE OF DOSE ESCALATED CONFORMAL PROSTATE RADIOTHERAPY

VS Khoo*[1], JL Bedford[2], S Webb[2], DP Dearnaley[1], [1]Academic Unit of Radiotherapy, and the Joint Department of Physics, ICR & Royal Marsden NHS Trust, Sutton SM2 5PT, UK

**Introduction** The aim was to determine the optimal coplanar beam arrangement for use in the boost phase of conformal prostate radiotherapy from 64 Gy to 74 Gy for a variety of three-field (3F), four-field (4F) and six-field (6F) plans. The boost phase planning target volume was altered from that used for the first 64 Gy and comprised the prostate gland alone without a planning margin.

**Methods** In ten patients, three optimised plans (3F 0°,90°,270° plan, 4F 45°,90°,270°,315° plan, and 6F 40°,90°,115°,245°,270°,320° plan) were selected from a variety of plan arrangements and compared with the following reference plans: 3F (0°,120°,240°) plan, 4F (0°,90°,180°,270°) plan, 6F (55°,90°,125°,235°, 270°,305°) and 6F (50°,90°,130°,230°,270°,310°) plans. All plans were for 6 MV photons. Beam weightings were inversely optimised for each patient. Plans were compared by means of rectal volumes irradiated to greater than 50% ($V_{50}$), 80% ($V_{80}$), and 90% ($V_{90}$) of the prescribed dose. Irradiated volumes were also measured for the bladder ($V_{90}$) and femoral heads ($V_{70}$).

**Results** All selected 3F, 4F and 6F plans gave lower rectal $V_{80}$ and $V_{90}$ than their corresponding reference plan. The 3F (0°,90°,270°) plan consistently provided greater rectal sparing at $V_{50}$ to $V_{90}$, with acceptable bladder and femoral head doses, compared to the other plans in the study. When the 6F (50°,90°,130°,230°,270°,310°) plan used at our institution for the boost phase was compared to the 3F (0°,90°,270°) plan, there were reductions of rectal $V_{50}$ from 20.8 ± 5.2% to 12.6% ± 5.1%, rectal $V_{80}$ from 8.7 ± 2.9% to 6.5 ± 3.1%, and rectal $V_{90}$ from 5.5 ± 2.1% to 3.9 ± 2.0% (all $p < 0.001$). The bladder $V_{90}$ and femoral head $V_{70}$ were equivalent.

**Discussion** Using a reduced planning target volume for the boost phase, the 3F beam arrangement using coplanar gantry angles of 0°, 90°, and 270° provided the best rectal sparing at the high dose regions with acceptable bladder and femoral head doses. This plan significantly improved on the 6F 40/40 plan being used at our institution. It is anticipated that this 3F plan would provide the least increase in high dose rectal irradiated volume when combined with any phase 1 plan. These plans were individually inversely optimised using conformal shaped blocks. Similar results were found when the beam weightings were standardised thereby providing a class solution. Other factors may impact on the practicability of implementation such as differences in multileaf collimator (MLC) shaping vs shaped blocks. MLC leaf widths (1 cm vs 3–5 mm), limitations in MLC orientation using integral wedges and these will need to be addressed.

### P184 OPTIMISATION OF 3-, 4-, AND 6-FIELD COPLANAR BEAM ORIENTATIONS FOR USE IN CONFORMAL DOSE ESCALATION OF PROSTATE RADIOTHERAPY

VS Khoo*[1], JL Bedford[2], S Webb[2], DP Dearnaley[1], [1]Academic Unit of Radiotherapy, and the Joint Department of Physics, Institute of Cancer Research & Royal Marsden NHS Trust, Sutton SM2 5PT, UK

**Introduction** We have evaluated the optimal coplanar beam arrangement for use in dose escalated conformal prostate radiotherapy. Two different clinical target volumes: prostate only (PO) and prostate plus seminal vesicles (PSV), a variety of three-field (3F), four-field (4F) and six-field (6F) plans and the impact of two different doses (64 and 74 Gy) were studied. *Methods*: Series of 3F, 4F and 6F plans were created for PO and PSV volumes in each of ten patients. The optimal plan for each of 3F, 4F and 6F were chosen from a variety of symmetrically and asymmetrical field arrangements and compared to their respective reference plans: 3F 0°, 120°, 240° plan, 4F 0°, 90°, 180°, 270° (box) plan, and 6F 55°, 90°, 125°, 235°, 270°, 305° plan (plan designated 35/35 describes the angle of the oblique beams (ant/post) from the mid-coronal plane). Comparisons were performed using the rectal volume irradiated to greater than 50% ($V_{50}$), 80% ($V_{80}$), and 90% ($V_{90}$) of the prescribed dose, femoral head (FH) $V_{70}$ and bladder (B) $V_{90}$, normal tissue complication probability (NTCP) for rectum (R), B, and FH. Over 1800 plans were computed.

**Results** The mean statistics (±1SD) for the best 3F, 4F, and 6F plan were compared with their respective reference plans:

| Plan Type | Reference 3F, 4F, and 6F plans | | | Optimised 3F, 4F, and 6F plans | | |
|---|---|---|---|---|---|---|
| | 3F | 4F | 6F | 3F | 4F | 6F |
| *PO Group %* | *0,120,240* | *box* | *35/35* | *0,90,270* | *35,90,270,325* | *50/25* |
| R $V_{50}$ | 51.3 ± 10.4 | 66.7 ± 14.6 | 40.9 ± 7.8 | 31.7 ± 6.6 | 49.8 ± 11.0 | 38.8 ± 7.7 |
| R $V_{80}$ | 35.2 ± 8.0 | 26.0 ± 5.8 | 26.3 ± 5.9 | 22.8 ± 5.5 | 24.0 ± 5.4 | 24.4 ± 5.3 |
| R $V_{90}$ | 30.3 ± 7.1 | 21.4 ± 5.2 | 21.7 ± 4.6 | 18.4 ± 5.7 | 19.5 ± 5.4 | 18.6 ± 4.9 |
| R NTCP 64 Gy | 2.0 ± 0.8 | 1.3 ± 0.5 | 1.3 ± 0.4 | 0.8 ± 0.3 | 0.9 ± 0.3 | 0.8 ± 0.2 |
| R NTCP 74 Gy | 8.9 ± 3.1 | 6.0 ± 2.1 | 6.2 ± 1.9 | 4.1 ± 1.5 | 4.5 ± 1.5 | 3.8 ± 1.2 |
| B $V_{90}$ | 9.9 ± 5.3 | 10.1 ± 5.3 | 8.9 ± 4.5 | 9.3 ± 5.1 | 8.5 ± 4.5 | 8.9 ± 4.5 |
| FH $V_{70}$ | 0.0 ± 0.0 | 0.1 ± 0.4 | 16.7 ± 23.2 | 0.6 ± 1.9 | 1.3 ± 2.6 | 7.4 ± 19.7 |
| FH NTCP 74 Gy | 0.0 ± 0.0 | 0.0 ± 0.1 | 4.4 ± 13.2 | 0.1 ± 0.1 | 0.0 ± 0.1 | 1.4 ± 4.2 |
| | 3F | 4F | 6F | 3F | 4F | 6F |
| *PSV Group %* | *0,120,240* | *box* | *35/35* | *0,90,270* | *20,90,270,340* | *65/30* |
| R $V_{50}$ | 83.8 ± 7.5 | 93.4 ± 7.6 | 74.8 ± 9.4 | 56.6 ± 6.5 | 82.2 ± 8.1 | 78.5 ± 8.4 |
| R $V_{80}$ | 65.7 ± 9.0 | 47.3 ± 5.5 | 49.4 ± 5.6 | 41.9 ± 5.8 | 44.2 ± 5.0 | 47.5 ± 6.3 |
| R $V_{90}$ | 58.8 ± 8.8 | 41.6 ± 5.1 | 42.3 ± 4.8 | 35.5 ± 5.9 | 37.0 ± 6.7 | 40.1 ± 6.8 |
| R NTCP 64 Gy | 5.7 ± 1.8 | 3.1 ± 0.7 | 3.7 ± 0.7 | 2.3 ± 0.6 | 2.4 ± 0.6 | 3.0 ± 1.0 |
| R NTCP 74 Gy | 21.4 ± 5.3 | 13.2 ± 2.3 | 15.1 ± 2.5 | 10.2 ± 2.4 | 10.6 ± 2.1 | 12.6 ± 3.5 |
| B $V_{90}$ | 19.5 ± 9.3 | 19.0 ± 9.5 | 15.7 ± 7.5 | 18.0 ± 8.9 | 18.5 ± 9.4 | 17.1 ± 8.6 |
| FH $V_{70}$ | 0.0 ± 0.0 | 0.0 ± 0.0 | 37.5 ± 25.4 | 3.4 ± 6.9 | 1.3 ± 3.0 | 3.9 ± 10.6 |
| FH NTCP 74 Gy | 0.0 ± 0.0 | 0.0 ± 0.0 | 9.1 ± 23.1 | 0.3 ± 0.4 | 0.0 ± 0.0 | 0.6 ± 1.8 |

**Conclusion** The optimal rectal sparing field arrangement with acceptable bladder and FH doses was a 3F plan with coplanar angles of 0°, 90°, and 270°. This finding was consistent for both PO and PSV volumes, and when dosed to either 64 Gy or 74 Gy. However, if the target volumes (using 1 cm margins) remained unchanged to 74 Gy, the irradiated rectal volumes were high and rectal NTCPs were predicted to increase by 4–5 fold. Therefore, it is prudent to reduced planning volumes for dose escalation to 74 Gy.

### P185 USE OF A DEPTH HELMET TO ASSESS AND IMPROVE ACCURACY OF A GTC STEREOTACTIC RADIOTHERAPY FRAME

KE Burton[1], D Whitney[2], DS Routsis[1], SJ Thomas[2], RJ Benson[1], NG Burnet[1]*, Departments of [1]Oncology and [2]Medical Physics, Addenbrooke's Hospital, Cambridge CB2 2QQ, UK

The value of using the GTC stereotactic radiotherapy frame is in improving accuracy of relocation for high precision radiotherapy. We sought to measure and improve the accuracy of our system, by use of a depth helmet. This consists of a perspex hemisphere with a set of 24 measuring portals. This attaches to the stereotactic frame once the patient is in the treatment position. A measuring rod is used to measure the distance from scalp to helmet.

Measurements were taken at the initial fitting, localisation and verification CTs, and for each treatment fraction for 20 consecutive patients, a total of 291 episodes. The localisation CT data were used as the reference set. The frame was refitted until the recorded measurements were within acceptance criteria: no worse than 1 reading >2 mm or 3 readings >1.5 mm. After the treatment course, each depth measurement was entered into a spreadsheet to calculate the vector displacements of the isocentre.

The mean displacements (and standard deviations) for the whole sample together were left-right 0.002 mm (0.620 mm), anterior-posterior 0.116 mm (0.532 mm) and superior-inferior 0.229 mm (0.594 mm). The maximum 95% confidence intervals were 1.22, 1.16, and 1.39 mm for left-right, anterior-posterior and superior-inferior dimensions respectively. Only 1% of treatments had a displacement exceeding 2 mm. These did not necessarily occur in the same patient or direction.

Use of all suitable measurements, together with the spreadsheet, gave greater reliability and true evidence of the variation of isocentre position. Variations caused by differences in pressure applied to the measuring rod are removed by calculation of the vector displacements in the left-right and anterior-posterior dimensions. Measurement of the depth onto the occipital plate, which supports the back of the head, proved a useful tool in repositioning the occipital plate in 1 case where it came loose. Comparison of measurements to the reference data set without the spreadsheet, on which the acceptance criteria are based, suggest much greater discrepancies than actually occur in the isocentre position calculated from vector displacements.

Errors attributable to the patient's relocation within the frame are very small using this system. Use of the helmet, and spreadsheet to calculate real isocentre movements, confirm the accuracy for individual patients. Therefore, we are introducing use of the spreadsheet so that the true vector displacements are calculated prior to the treatment fraction, and acceptance criteria will be based on these results. Considering the 95% confidence intervals, which are all ≤ 1.39 mm, with other potential errors, we now use a CTV-PTV margin of 3 mm for this high precision technique.

### P186 RADIOTHERAPY FOR PREVENTION OF HORMONE-INDUCED GYNAECOMASTIA AND MAMMALGIA IN ADVANCED PROSTATE CANCER

RSD Brown[1], JF Money-Kyrle[1], HA Payne[1] and GM Duchesne[2], [1]Middlesex Hospital, Mortimer Street, London W1N 8AA, [2]Peter MacCallum Cancer Institute, Melbourne Australia

**Purpose** Low-dose diethylstilbestrol (DES 1 mg) therapy may produce a sustained response in advanced prostate cancer but causes gynaecomastia and nipple tenderness. We have reviewed our experience with prophylactic nipple irradiation in preventing this complication.

**Patients and Methods** Fifty-nine patients with relapsed prostate cancer due to commence DES 1 mg were treated with short distance $^{60}Co$ γ rays using an applied dose of 15 Gray in 3 fractions to a 6 cm circular nipple field. Each patient was examined and scored for mammalgia and gynaecomastia pre-radiotherapy, and at 3, 6, and 12 months post-radiotherapy.

**Results** 41/59 patients had neither gynaecomastia nor mammalgia prior to radiotherapy and 36 (88%) of these remained unaffected. 5/41 patients developed gynaecomastia or mammalgia despite prophylactic radiotherapy. No patient developed more than mild to occasional mammalgia (Grade I) or questionable gynaecomastia (Grade I). For patients with pre-existing gynaecomastia or mammalgia (18/59), stabilisation occurred in 14/18 (78%), improvement occurred in 3/18 patients (17%) and progression occurred in 1 patient (5%). No acute side effects were noted from radiotherapy treatment.

**Conclusion** Prophylactic nipple irradiation is an effective modality in preventing 1 mg DES-induced gynaecomastia and mammalgia in patients with advanced prostate cancer. The effectiveness and low acute side effect rate suggest the possibility of considering prophylactic nipple irradiation in earlier stage adjuvant treatment of prostate cancer if other drugs that causes similar breast changes (e.g. oral anti-androgen therapy) are to be considered. Long term follow up would be needed to check for late effects.

### P187 POLYMORPHISMS IN TNF∝ AND RISK OF BLADDER CANCER

H Marsh*[1,2], N Haldar[1], M Bunce[1], S Marshall[1], A Harris[2], K Welsh[1], [1]Tissue Typing Laboratory Churchill Hospital, Oxford OX3 7LJ, [2]ICRF, John Radcliffe Hospital, Oxford OX3 9DU, UK

**Introduction** TNF∝ is known to play a key role in the regulation of the angiogenic enzyme thymidine phosphorylase (1) and TNF∝ deficient mice have a greatly reduced tumour growth and tumour induction in response to chemical carcinogens. (2). There are at least 14 known polymorphisms in and around TNF and several of these polymorphisms have been implicated in a number of conditions including malignant, inflammatory and granulomatous disease. (3).

**Aims** We set out to assess the association between TNF∝ polymorphisms and the risk of bladder cancer by determining the frequency of TNF polymorphisms in bladder cancer patients and a control population.

**Methods** We looked for 12 polymorphisms in 100 bladder cancer patients and 100 controls (renal donors — no malignancy). DNA was extracted from peripheral blood and amplified by PCR using sequence specific primers. PCR products were then electrophoresed in 1% agarose gels and visualised with UV illumination.

**Results** Initial results have shown a highly significant association with 2 polymorphisms in TNF∝. At locus –488, GA was found in 30% of patients compared with 11% of controls, p=0.0005 (p=0.006 with bonferoni correction) and at –238 GA was found in 1% of patients compared to 15% of controls p=0.0003 (p=0.004 corrected).

**Conclusions and significance** This preliminary result shows a highly significant association between 2 TNF polymorphisms and bladder cancer. It must be confirmed with a second set analysis and the remaining TNF polymorphisms must be assessed. If correct then we will follow this up with functional studies. Ultimately this work could lead to new methods of detecting patients at risk of bladder cancer and new therapeutic techniques such as TNF antagonism.

1 Leek RD et al (1998) *Br J Cancer* **77**: 2246
2 Moore RJ et al (1999) *Nat Med* **5**; 7:828
3 Mulligan CG et al (1999) *Genes and Immunity* **1**: 137

### P188 THE GENETIC BASIS OF ADENOCARCINOMA OF THE BLADDER WITH SPECIAL EMPHASIS ON THOSE ARISING IN "CLAM" BLADDERS

Appanna TC*[1], Croft J[2], James S[2], Parry EM[2], Parry JM[2], Stephenson TP[1], [1]Department of Urology, University Hospital of Wales, Cardiff, [2]Centre for Molecular Genetics and Toxicology, U.W.C.S., Swansea, UK

**Introduction** Since its introduction in 1982 the "clam" enterocystoplasty has become widely used for the treatment of detrusor hyperreflexia and instability. Over 400 operations a year are being performed in the U.K. There is thought to be a significant risk of malignancy in these bladders and in our unit alone there have been four such tumours. They were all highly aggressive adenocarcinomas in the native bladder segment. Filmer reported a latency period of between 5 and 29 years for tumour development. There are potentially over 4000 patients in the U.K. alone at risk a lot of whom are quite young. Ideally we would like to identify those most at risk so that we could follow them up more closely. Our first aim therefore is to identify genetic irregularities peculiar to these tumours as reference points.

**Materials and methods** DNA is extracted from the slides of 2 clam tumours that we have had and 2 de novo adenocarcinomas of the bladder using a commercially available kit (PROMEGA). DNA has also been extracted from biopsy samples taken from the bladder remnant of clam patients. The DNA is then subjected to a technique called Comparative Genomic Hybridisation (CGH). Firstly the DNA is nick translated with a fluorescent marker (Fluoro Green) and then competitively hybridised with control DNA to a normal human metaphase spread. The hybridisation is visualised by the use of 2 different fluorochromes. The ratio of fluorescence intensities along each chromosome reflects the relative ratios of test and reference sequences. We can therefore show amplifications and/or deletions anywhere in the chromosomal series.

**Results** The analysis of the tumours has shown several amplifications most notably on 8 p on the clam tumours and 21 q common to both clam and de novo adenocarcinomas. Biopsies from clam patients without tumour show greater genetic instability nearer the bowel to bladder anastamosis than those from further away.

**Discussion** The spectre of the possibility of clam cancer still hangs over us. We need to be able to try and predict which patients are most at risk. If we can identify a sequence of DNA changes leading to tumour formation then we can use them as indicators for the clam patients. If any of them develop similar DNA changes then they will require closer follow up.

### P189 CYCLOOXYGENASE2 EXPRESSION IN SUPERFICIAL TRANSITIONAL CELL CARCINOMA OF BLADDER

P Maheshkumar[1]*, JE Martin[2], VH Nargund[1] and DM Berney[2], Dept of Urology and [2]Dept of Histopathology and Morbid Anatomy, Barts and The London NHS Trust, London, EC1A 7BE, UK

**Objectives** Cyclooxygenases (Cox) catalyse the synthesis of prostaglandins from arachadonic acid. Two isoforms of Cox exist, Cox1 and Cox2. Cox1 is constitutively expressed and has a physiologic role in the cell metabolism. Cox2 is induced by various stimuli such as growth factors, carcinogens and tumour promoting phorbol esters. The constitutive isoform of Cox1 is essentially unaffected by these factors. Previous studies have shown that Cox2 is increased in invasive bladder cancer. Animal studies have also shown the anti tumour effects of Piroxicam (NSAID) in spontaneous canine transitional cell carcinoma (TCC) bladder. Recently, selective Cox2 inhibitors have been approved for clinical use in the UK, they have fewer side effects than traditional NSAID's. The purpose of this study was to determine the Cox2 expression in superficial TCC bladder and any possible relationship with grade, stage or disease progression.

**Materials** Formalin fixed tumour tissues (n=169) were obtained for all newly diagnosed superficial bladder cancer between 1982 to 1988. These were evaluated for Cox2 expression by immunohistochemistry following microwave antigen retrieval.

**Results**

| TCC-grade and stage | Cox2 +ve | Cox2 –ve |
|---|---|---|
| pTa (n=120) | 37 (31%) | 83 (69%) |
| pT1 (n=49) | 13 (27%) | 36 (73%) |
| G1 (n=66) | 19 (29%) | 47 (71%) |
| G2 (n=62) | 21 (34%) | 41 (66%) |
| G3 (n=41) | 10 (24%) | 31 (76%) |

Cox2 expression is seen in a small proportion of superficial TCC and is unrelated to grade or stage. Subset analysis of those TCC's that subsequently progressed (n=39) also did not reveal any association with Cox2 expression.

**Conclusions** COX2 expression does not provide useful diagnostic or prognostic information in superficial TCC. The biological role of this enzyme in superficial TCC bladder cancer remains unclear.

### P190 LOSS OF HETEROZYGOSITY ON CHROMOSOME 9 AS A POTENTIAL MARKER OF RECURRENCE AND PROGRESSION IN BLADDER CANCER

J Edwards*[1], P Duncan[1], JJ Going[2], AD Watters[1] and JMS Bartlett[1], Dept of Surgery[1]/Dept of Pathology[2], Glasgow Royal infirmary, Glasgow G31 2ER, UK

Approximately two thirds of patients diagnosed with superficial transitional cell carcinoma (TCC) of the urinary bladder will recur within 2 years. It was recently reported that loss of chromosome 9 in primary TCC identifies a subset of patients at high risk of recurrence (Bartlett *et al.* 1998, BJC. 77 (12), 2193–2198).

This study explores in further detail genetic alterations at chromosome 9 as possible predictors of recurrence and/or progression. Forty-seven patients with full follow-up were retrospectively selected and categorised into 2 groups, (non-recurrent TCC of the bladder (NR, n=18) or recurrent TCC of the bladder (REC, n=29). The REC group was further divided into recurrer non progressors (RNP, n=18) and recurrer progressors (RP, n=11). Patient DNA was microdissected and extracted from archival normal/tumour tissue sections and analysed for loss of heterozygosity (LOH) at 9 loci on chromosome 9. Fluorescent PCR was performed and genotyping carried out on a Perkin Elmer AB1377™ sequencer.

The level of LOH in primary tumours of the patient groups was studied. In the NR group 83% of patients exhibited at least one LOH at the loci tested, this ranged from 1/9 loci per patient to 6/9 loci per patient, 0 patients had LOH at all loci tested. In the REC group 100% of primary tumours exhibited at least one LOH at the loci tested, this ranged from 1/9 loci per patients to 9/9 loci tested per patient. LOH at all loci tested was seen in 24% of REC patients. A significant difference between the NR and REC (p=0.04) group was observed. However when the REC group was sub divided and compared a significant difference was only seen between the primary NR and RNP tumours (p=0.03). When LOH at individual loci was compared no difference was found between groups, except when the markers spanning 9q34 were investigated, as it was noted that 11% of NR primary tumours lost this region compared to 51% of REC primary tumours (p=0.01). The total level of LOH in NR tumours was 33% compared to 70% in the REC patients. The level of LOH inpatients recurrent tumours was also investigated. There was no increase in level of LOH observed with repeated tumour recurrences in the RNP group. However the level of LOH increased at the tumour recurred and progressed in the RP group, the level of LOH observed in the post invasive tumours was 61% compared to 37% in the primary tumour (p=0.003).

A higher rate of LOH, in particular at 9q34 is seen in primary tumours of REC patients when compared to NR patients, however only LOH at all informative markers on chromosome 9 was demonstrated to predict recurrence. LOH at multiple loci on chromosome 9 implies that more than one tumour suppressor gene located on chromosome 9 is involved in recurrence of TCC of the urinary bladder.

### P191

WITHDRAWN

### P192 A PHASE I/II STUDY OF SYNCHRONOUS CHEMORADIOTHERAPY FOR POOR PROGNOSIS LOCALLY ADVANCED BLADDER CANCER

SA Hussain*[1,2], DD Moffitt[1], JG Glaholm[2], D Peake[2], DMA Wallace[2], ND James[1,2], [1]CRC Institute For Cancer Studies, University Hospital Birmingham, [2]Queen Elizabeth hospital, Edgbaston, Birmingham, UK

**Purpose** To investigate synergy between radiotherapy and synchronous chemotherapy using 5-fluorouracil (5-FU) and Mitomycin-C (MMC) and evaluate the toxicity and activity in patients with muscle invasive bladder cancer in a phase I/II study.

**Method** Patients with T2-T4 NO/Nx MO muscle invasive bladder cancer were entered into this single centre study. Patients received 55 Gy/20 fractions/4 weeks of radiotherapy. Concurrent chemotherapy was given with MMC 12 mg/m$^2$ day 1 and 5-FU 500 mg/m$^2$/24 hours weeks one and four of radiotherapy for five or seven days on each occasion. Primary end point for phase-I part of the study was toxicity. Primary end point for phase II part of the study was pathological response rate at 3 months, secondary endpoints for both parts were toxicity, disease-free and overall survival. Dose escalation was carried out by increasing the duration of continuous infusion of 5-FU chemotherapy from 10 to 14 days after 20 patients.

**Results** A total of 31 patients have entered the trial from March 1998 to December 99 (22 5-day, 9 7-day schedule). Date of censor was 6th February 2000. Median age was 68 (range 58–79) years, 23 males and 8 females; 2 patients were node positive; T2 9 (29%), T3a 4 (12%), T3b 9 (29%), T4 9 (29%), TCC grade 2, 8 (26%) and grade 3, 23 (74%); 14/31 patients had hydronephrosis. Toxicity was mild to moderate with the 5-day schedule (NCI-CTG grade 3: thrombocytopenia 2/22; diarrhoea 2/22). More severe toxicity was seen with the 7-day schedule (NCI-CTG grade 3: leucopenia 1/9; diarrhoea 2/9; cystitis 2/9). 4/9 patients failed to complete planned therapy. There was 1 death on treatment from stroke, probably unrelated to therapy. We have now reverted to the 5-day schedule for an extended phase II study. Of 17 patients with the 5-day schedule eligible for local response assessment, pathological complete response at 3 months was 70%; 3 patients were not evaluable due to metastatic spread. 9 patients received the 7-day regimen, of the eight patients due for response assessment the 3 month pathological response rate was 6/7 (84%) pathological CR (3) 42% + pathological PR (3) 42%. Actuarial 12-month survival was 57%. Of patients still alive from either schedule, 16/18 retain a functioning bladder at median follow up of 13.6 months.

**Conclusion** Chemoradiotherapy with the 5-day schedule is feasible in the management of elderly patients. The regimen has encouraging activity with acceptable toxicity in poor prognosis patients. A randomised trial using the 5-day schedule compared to radical radiotherapy alone is being launched.

**Key words** *Bladder cancer Chemoradiation Organ preservation*

### P193 LOCAL ANAESTHETIC PHOTODYNAMIC THERAPY FOR SUPERFICIAL BLADDER CANCER: EARLY CLINICAL RESULTS

D Shackley*[1,2], C Whitehurst[1], JV Moore[1], A Gilhooley[2], CD Betts[2] and NW Clarke[2,3], [1]CRC Department of Experimental Radiation, Oncology, Paterson Labs, Christie Hospital Manchester M20 4BX; [2]Department of Urology, Hope Hospital, Salford M6 8HD. [3]Department of Urology, Christie Hospital, Manchester M20 4BX, UK

**Introduction** Photodynamic therapy (PDT) is a potential new treatment for bladder tumours. Presently, this requires a general anaesthetic (GA), bladder irrigation and rigid cystoscopy. We have developed a system whereby PDT is given via a flexible cystoscope under local anaesthetic (LA) in a clinic environment. This system may help treat patients who cannot have a GA. We present our initial clinical results.

**Patients and Methods** Eight patients (five men, three women; mean age 75, range 66–83) with recurrent superficial bladder cancer (stage Ta, grades 1–2) were sensitised with 50 mls of intravesical 3% Aminolaeuvulinic acid (ALA) solution. After 4 hours, this was replaced by 40 mls of 2% lignocaine for 45 minutes and then by 150 mls of saline. 50 J/cm2 of 633 nm laser light was then delivered via a flexible cystoscope.

**Results** The procedure was well tolerated and painfree. Thereafter, bladder frequency was noticed in all patients usually settling within 1 week. One patient had irritability which lasted 4 weeks before settling completely. There were no skin photosensitivity episodes nor post-PDT changes in objective bladder function tests. Serum lignocaine levels were insignificant. Intravesical temperature measurements via a thermocouple demonstrated no hyperthermic effect of the treatment. Independent cystoscopic follow-up at 2 months showed 6 patients had a substantial response to treatment (2 had a complete response and 4 had a partial response (>50% reduction in size/numbers of visible tumours)). A smaller response was noticed in 1 of the remaining 2 patients.

**Conclusions** Local anaesthetic bladder PDT is possible, safe and effective in recurrent superficial TCC. Larger therapeutic studies are now possible under LA to evaluate dose-response characteristics.

### P194 A NEW HIGHLY SPECIFIC MONOCLONAL ANTIBODY AGAINST PLACENTAL ALKALINE PHOSPHATASE (PLAP): A POTENTIAL MARKER FOR AN EARLY DETECTION OF TESTIS TUMOUR

AME Nouri, AME, Dabare AANPM, Torabi-Pour N and Oliver RTD, Department of Medical Oncology, The Royal London Hospital. Whitechapel London E1 1BB, UK

The aim of this study was to develop specific monoclonal antibodies (Mab) against human germ cell tumours. For this, the preparation of a single cell suspension obtained from tumour tissue fragments (consisting of both tumour and normal compartments) from a patient with seminoma was used as an immunogen. Spleen cells from the immunised mice were used to develop Mabs. Analysis of tissue specificity, biochemical characteristics, as well as competitive studies were carried using techniques which included immunocytochemical staining, dot blot and a Western blot analysis for the identification of target antigen(s).

The results showed that: 1. The immunisation protocol led to the development of 107 hybridomas, 90 of which were negative against the original tissue biopsies. The remaining 17 showed positivity against various tissue compartments. One chosen Mab i.e. ATC2 showed specific staining on germ cell tumours but not on normal tissues. It also showed positive staining with some human tumour cell lines. 2. The nature of target antigen for ATC2 Mab was found to be PLAP based on:

a. Western blot analysis compared with commercially available PLAP.
b. Comparison of the data with another well-known anti-PLAP Mab i.e. H17E2 although the two Mabs recognised different antigenic epitopes.
c. Heat resistance characteristics.
d. HPLC analysis of the ATC2 target antigen and comparison with purified PLAP.

These data confirm the exquisite specificity of ATC2 for human germ cell tumours. The results also showed that the target antigen for ATC2 Mab was PLAP, although the antigenic epitope(s) was different from those recognized by H17E2 Mab. The ATC2 may prove to be useful for monitoring sera levels of PLAP in testis cancer patients. It may also prove to be relevant for detecting cancer cells in semen of individuals suspected of testis cancers particularly in those with equivocal ultrasound.

### P195 THE EFFECT OF TREATMENT ON THE HEALTH OF LONG TERM SURVIVORS OF TESTICULAR CANCER

RA Huddart, A Norman, D Coward, E Nicholls, G Jay, M Shahidi, A Horwich, DP Dearnaley, Royal Marsden Hospital and Institute of Cancer Research

The long term effects of treatment are an important consideration when deciding treatment options in cancers which are largely curable such as testicular cancer. To evaluate the effects of treatment on the health of survivors of testicular cancer we have undertaken a cross-sectional study of patients treated at our institution with a minimum of 5 years follow up. Patients completed general health and quality of life questionnaires, underwent biochemical and hormonal profiles and clinical review. Of 1603 patients treated between 1982 and 1992; 1203 are alive and fulfilled study entry criteria. Of these, 730 patients entered the study. Information on cardiovascular disease (CVD) and health problems was obtained on a further 202 patients not under regular review. To study treatment effects, patients were divided into four groups according to clinical management; surveillance (S) (N=233), radiotherapy (RT) (N=219), chemotherapy (C) (N=371) and both chemotherapy and radiotherapy (C/RT) (N=123). Median follow up was (in years) S 11.0, RT 9.1, C9.2, C/RT 11.1.

There was an increase risk of CVD in patients receiving RT (relative risk (RR) 2.2; p=0.026), C (RR 1.8, p=0.09), and C/RT (RR 2.0, p=0.08), compared to patients on surveillance. This was associated with a trend to increased risk of hypertension in patients receiving any treatment (14% v 9% for S p=0.1) and detectable biochemical changes including lower serum $Na^+$ (RT C/RT) and $Mg^{2+}$ (C/RT); increased $K^+$ (RT, C, C/RT), creatinine, (RT, C, C/RT) and protein, (C, C/RT). There was no difference in smoking history or cholesterol in these groups.

A trend to increase in non testicular second malignanacies was also seen in patients receiving treatment, (RR: RT 1.9, C 1.6, C-RT 2.55 p=0.2). Chemotherapy treatment was associated with an increased frequency of sensory peripheral neuropathy (C 20%, S 7% p<0.001), Raynauds syndrome (C 22%, S1% p<0.001), tinnitus (C 24%, S 12% p=0.066) and hearing changes (both objective and subjective p<0.001). Radiotherapy was associated with a reduced WBC (median($\times 10^9$) RT 6.2, S 6.9 p=0.005), Hb (median(g/dl) RT 14.2, S 14.5 p=0.006) and Platelet counts (median ($\times 10^9$) RT 217, S 228p=0.053) and an increased risk of GI symptoms (RT 8%, S 3% p=0.03). There were high levels of gonadal dysfunction in all groups (low testosterone 16%, raised FSH 49%, raised LH 12%).

Long term survivors of testicular cancer treatment are at an increased risk of cardiovascular disease, increased risk of second malignancy and other health effects. This data will be helpful in the counselling of patients and definition of treatment and follow up, particularly in patients with Stage I disease.

### P196 PACLITAXEL CHEMOTHERAPY IN THE SALVAGE OF MULTIPLY RELAPSED AND PLATINUM REFRACTORY GERM CELL TUMOURS

JN Staffurth[1]*, J Nichols[1], RA Huddart[1], DP Dearnaley[1], A Horwich[1], [1]Royal Marsden NHS Trust, Sutton, UK

70–80% of patients with metastatic germ cell tumour will be cured with initial platinum-based chemotherapy. Although 40% of the relapsing patients will be salvaged with either ifosfamide-based conventional regimens or high-dose chemotherapy, patients with multiply relapsed or platinum refractory tumours have an extremely poor outlook. We investigated paclitaxel combination chemotherapy in this patients group.

From 1996–99, nine patients with platinum refractory or multiply relapsed germ cell tumours have been treated at our centre with paclitaxel containing regimens. Five patients received paclitaxel with cisplatin/carboplatin, the remaining four received paclitaxel and ifosfamide, with cisplatin/carboplatin. All patients had a testicular primary tumour, two seminomas and seven malignant teratomas. The median age of the patient was 38 years (range, 30–49). 6 patients (66%) had ≥2 metastatic sites. Median number of previous platinum cycles was nine (range, 5–15). Six had received high dose carboplatin and etoposide with peripheral stem cell rescue. Two patients were late relapses.

One patient (11%) had a complete response to the paclitaxel regimen, with only differentiated teratoma in the resected specimen; his response lasted seven months. No other patients responded.

The major toxicity was haematological, 66% of the patients suffering grade III/IV neutropenia.

Paclitaxel has previously been shown to have a single-agent response rates of 10–26% in salvage of selected patients with relapsed germ cell tumours, and can be incorporated safely into combinations with ifosfamide and cisplatin. However, in our experience of heavily pre-treated patients, only one patient had a complete response, lasting only seven months.

### P197 LATE RELAPSE OF TESTICULAR GERM CELL TUMOURS AND IMPLICATIONS ON LONG TERM FOLLOW UP

M Shahidi[1], AR Norman[2], J Nicholls[3], DP Dearnaley[1], RA Huddart[1], A Horwich[1], [1]Academic Unit of Radiotherapy & Oncology, Royal Marsden NHS Trust and the Institute of Cancer Research, [2]Department of Computing and Information, [3]Bob Champion Unit, Royal Marsden NHS Trust Downs Road, Sutton, Surrey, UK

Testicular germ cell tumours (GCTs) are highly curable, however 10–30% of patients relapse after initial treatment. The time course of relapse has implications for duration of follow up. This study was undertaken to assess the time course of relapse and to identify patients with higher risk of late relapse. A review was performed of 1264 patients with primary testicular germ cell tumors presenting to the Royal Marsden Hospital between December 1979 and December 1993. In all 256 episodes of relapse were documented (including 44 patients with multiple relapses). Relapse-free survivals were calculated (table). 54 patients (16 seminoma; 38 NSGCT) relapsed more than 2 years after initial presentation. A multivariate analysis of risk of relapse after 2 years identified advanced disease to be the only patients (1 seminoma; 14 NSGCT) relapsing after 5 years, 14 had metastatic disease at presentation. Relapse are rare in testicular GCTs and follow up to detect recurrence may not be needed beyond 5 years except in those presenting with metastatic non-seminomatous GCTs.

**Relapse free survival (No of patients at risk)**

| | 2 year | 5 year | 10 year |
|---|---|---|---|
| Seminoma Stage 1 | 93.7% (344) | 91.4% (314) | 91.4% (158) |
| Seminoma Stage 2 | 85.7% (93) | 81.0% (81) | 80.0% (53) |
| Seminoma Stage 3–4 | 75.8% (25) | 75.8% (24) | 75.8% (19) |
| NSGCT Stage 1 | 79.9% (273) | 76.6% (233) | 76.6% (132) |
| NSGCT Stage 2 | 91.6% (144) | 90.2% (127) | 87.9% (74) |
| NSGCT Stage 3–4 | 81.4% (151) | 76.9% (131) | 74.5% (90) |

### P198 TEN YEAR REVIEW OF SURVIVAL OF PATIENTS WITH ADVANCED NON-SEMINOMATOUS GERM CELL TUMOURS (NSGCT): THE WESTON PARK EXPERIENCE

SI Ahmed*, J George, JM Coleman, CR Radstone, JM Horsman, MQ Hatton, RE Coleman, YCR Department of Clinical Oncology, Weston Park Hospital, Sheffield, UK

Germ cell tumours constitute 40% seminomas and 60% non-seminomas (NSGCT). Surgery and cisplatin-based chemotherapy are the main treatment modalities for metastatic NSGCT, resulting in a survival rate of 80–90%. In an attempt to target toxic treatments to patients at highest risk, categorisation of patients into prognostic groups has resulted in the International Germ Cell Cancer Collaborative Group (IGCCCG) prognostic grouping. This grouping considers the site of the primary tumour, three markers (AFP, βhCG, LDH) and the presence of non-pulmonary visceral metastases. The 5 year survival for good, intermediate and poor prognosis patients were reported to be 92%, 80% and 48% respectively[1]. Although BEP (bleomycin, etoposide, cisplatin) remains the standard treatment for metastatic NSGCT, more intensive regimens have been suggested for the intermediate and poor prognosis patients including the POMB/ACE (cisplatin, vincristine, methotrexate, bleomycin/dactinomycin, cyclophosphamide, etoposide) regimen.

**Aim & Objectives** To determine survival rates of patients with advanced NSGCT with respect to age at diagnosis, prognostic group and chemotherapy regimen, as compared to those published by the IGCCCG.

**Method** A retrospective analysis of survival data of *all* patients with advanced NSGCT treated at Weston Park Hospital, between January 1989 and November 1999 was undertaken. Data was obtained from the Trent Regional Testicular Tumour Registry Database. Patients with stage I disease on surveillance were excluded. Disease free (DFS) and overall survival (OS) were determined.

**Results** 143 male patients with NSGCT were identified. The proportion of patients in the good, intermediate and poor prognostic groups were 76 (53%), 29 (20%) and 38 (27%) respectively. The 5-year OS for the good, intermediate and poor prognostic groups were 95.8%, 77.5% and 57.1% respectively. This compares well with the IGCCCG survival rates. The IGCCC prognostic group was the only statistically significant variable impacting on DFS (p=0.002) and OS (p=0.004).

**Conclusion** 5 year survival rates at this single Cancer Centre were found to be higher than those published by the IGCCCG in all groups, except the 5-year OS for the intermediate prognostic group. The significantly better survival rate found in this study, for the poor prognostic group may be due to the use of the POMB-ACE regimen.

1 IGCCCG (1997) *JCO* **15:** 594

### P199 GENETIC ANALYSIS OF A POTENTIALLY PRE-MALIGNANT PHENOTYPE IN OVARIES OF WOMEN WITH BRCA MUTATIONS

J Gale*, I Campbell, D Eccles, N Singh, P Englefield, CRC Wessex Medical Oncology Unit, Southampton General Hospital, Southampton, UK

Prophylactic oophorectomy specimens from six women were examined; five had BRCA1 and one a BRCA2 mutation. Atypical features were identified, and microdissected from sections of paraffin-embedded ovarian tissue; matching normal cells were obtained from other areas of the same section. DNA was extracted from dissected tissue and leucocytes for each individual, and examined using microsatellite PCR for loss of heterozygosity (LOH) at candidate loci on all chromosome arms. Most sections revealed inclusion cysts; for one individual there were also multiple areas of microscopic malignancy. Material from the BRCA2 mutant showed no LOH.

Amongst the remaining samples the following LOH was detected.

| Chromosome arm | LOH inclusion cyst (n=9) | LOH microscopic malignancy (n=9) |
|---|---|---|
| 2p | 1 | 1 |
| 5p | 1 | – |
| 5q | 1 | 1 |
| 6q | 2 | 4 |
| 7q | – | 1 |
| 8q | 1 | 1 |
| 9p | 2 | – |
| 9q | – | 2 |
| 11q | 2 | – |
| 13q | 3 | 1 |
| 15q | 4 | 5 |
| 17q | 4 | 5 |
| Xq | 1 | – |

LOH on 5q, 6q, 9p, 11q and 17q has been previously reported for benign, borderline, and low grade malignant ovarian tumours. For all those chromosome arms demonstrating LOH within areas of microscopic malignancy, losses have been previously described in ovarian carcinoma. The data collected supports the concept of a histological pre-malignant phenotype in the ovaries of women with BRCA1 mutations.

### P200 A PHASE I STUDY OF SEQUENTIAL TOPOTECAN AND INFUSIONAL ETOPOSIDE PHOSPHATE IN RECURRENT OVARIAN CANCER

Deplanque G, Propper D, Levitt N, Bates N, Jones P, Flanagan E, Perry J, Joel S, Ganesan TS, ICRF Medical Oncology Unit, Oxford Radcliffe Hospitals, Oxford OX3 7LJ, UK and ICRF Medical Oncology Unit St. Bartholew's Hospital, London EC1A 7BE, UK

**Background** Topotecan (T) and etoposide phosphate (EP) are both active single agents in the treatment of recurrent ovarian cancer. Preclinical *in vitro* and *in vivo* studies suggest that they may act synergistically in sequential combination when inhibition of topoisomerase I> by T is followed by upregulation of the EP target, topoisomerase II.

**Study design** Phase I study to determine the toxicity and maximum tolerated dose (MTD) of the sequential combination of topotecan iv day 1 to 5 followed by 120 hrs infusional etoposide phosphate (day 6 to 11) targeting specific plasma etoposide concentrations. Pts were evaluated for response at 3 cycles and responding patients received a maximum of six cycles.

**Patient characteristics** A total of 18 pts have been entered into the trial and 10 pts are evaluable for toxicity and response. Median age is 55.5 years (31 to 66). Pts were treated in second line (2), third line (5) and fourth line (3). Eight pts were resistant to platinum. All the pts in dose levels 1, 2 and 1/5 at dose level 3 had received paclitaxel previously in addition to platinum.

| Dose level | Topo mg/m$^2$ | EP (μg/ml) | Pts | Response |
|---|---|---|---|---|
| 1 | 1 | 2 | 2 | 2PR |
| 2 | 0.85 | 2 | 3 | 1PR 2PD |
| 3 | 0.85 | 1 | 5 (+4) | 1PR 1SD 3PD (4NE) |
| (4) | (0.85) | (1.5) | (4) | (Not evaluable) |

**Toxicities** 46 cycles have been administered. Myelosuppression was the dose limiting toxicity (DLT). At dose levels 1 and 2, grade 4 neutropenia was observed in all pts. At dose level 3, grade 4 toxicity was observed in 1/5 evaluable pts. Alopecia (grade 2) and nausea and vomiting (grade 1) were the other toxicities observed.

**Efficacy** 4 PR for an ORR of 40%, with PR seen at each dose level.

**Conclusion** Sequential T plus infusional EP is feasible and promising, and warrants further investigation in recurrent ovarian cancer. The maximum tolerated dose for phase II trials has not yet been reached.

### P201 WEEKLY CISPLATIN AND ORAL ETOPOSIDE AS TREATMENT FOR RELAPSED OVARIAN CANCER

T Meyer*, AE Nelstrop, M Mahmoudi and GJS Rustin, Dept Medical Oncology Mount Vernon Hospital, Rickmansworth Road, Northwood, Middlesex HA6 2RN, UK

van der Berg has previously reported high response rates to weekly cisplatin and oral etoposide in patients with platinum refractory relapsed ovarian cancer (van der Berg 1996 Proc Am Soc Clin Onc 15: Abs 772). We analysed 42 women with relapsed, advanced ovarian cancer who were treated using a similar regimen with cisplatin 60 mg/m$^2$ on days 1,8,15,29,36 and 43 and oral etoposide 50 mg given from day 1–15 and day 29–43. In those who were responding and tolerating treatment (n=13) oral etoposide 50 mg was continued for two further cycles days 1–21 repeated every 28 days. The regimen was given as 1st line relapse therapy in 9 patients, as 2nd line in 19, 3rd line in 7 and 4th line in 8 patients. The mean age of the patients was 57.1 years (range 33.8–80.7). The median time since previous treatment with cytotoxic chemotherapy was 120 days and the median time since previous platinum administration was 223 days. A median of 5 doses of cisplatin was given to each patient, with 17 receiving the full 6 doses. A median of 28 doses of etoposide was given.

33 patients were evaluable for response according to CA 125 criteria (as defined in Rustin et al 1996 J Clin Oncol 14: p1545) giving an overall response rate of 39.3% (30.2% if non-evaluable patients were included). The response rate in evaluable patients declined with increasing numbers of previous treatments: 57.1% with 1 prior treatment, 40% with 2, 33.3% with 3 and 20% with 4. The response rates in evaluable patients who had received prior platinum chemotherapy within 6 months (median 121 days) prior to treatment was 41.7% (5/12), while in those who had received platinum more than 6 months previously (median 366 days) the response rate was 35% (7/20). Treatment was reasonably well tolerated with the only significant non-haematological toxicity being nausea and vomiting in 4 patients experiencing greater than grade 2 toxicity. The number of patients experiencing haematological toxicity more than grade 2 was as follows: haemoglobin 3, white blood count 12, platelets 6. 16 patients had dose delays and 2 had dose reductions.

We conclude that this short but intensive regimen provides worthwhile response rates, even in those patients who would ordinarily be considered refractory to platinum, and has an acceptable toxicity profile.

### P202 PRELIMINARY RESULTS OF A PHASE II STUDY OF WEEKLY PACLITAXEL AND ETOPOPHOS CHEMOTHERAPY IN PATIENTS WITH RELAPSED OVARIAN CARCINOMA

SI Ahmed*, J Higgins, T Briggs, SD Pledge, RE Coleman, YCR Department of Clinical Oncology, Weston Park Hospital, Sheffield S10 2SJ, UK

Although the prognosis for advanced ovarian cancer has improved, the median time to relapse remains around 18 months, often with platinum resistant disease. Paclitaxel (Taxol™) is usually administered on a 3-weekly schedule. A number of small studies have identified that weekly Taxol is feasible and has produced responses even after recurrences or progression on standard Taxol-based regimens. Etoposide has shown activity in ovarian cancer in the salvage situation. Etoposide phosphate (Etopophos™) has been recently formulated allowing administration as 5 minute bolus injection. Taxol and Etopophos may act synergistically.

**Aim & Objectives** This is an ongoing study to assess efficacy and tolerability of weekly Taxol and Etopophos given over 12 weeks, in patients with recurrent ovarian cancer. Principle end-points are response rate, time to progression, toxicity and assessment of quality of life (EORTC QLC30 questionnaire).

**Method** Patients with recurrent disease following at least one platinum and/or taxane based chemotherapy, were recruited to receive Taxol 60 mg/m$^2$ and Etopophos 100 mg/m$^2$ on a weekly schedule over 12 weeks in out-patients.

**Results** 13 patients have been recruited to date, all had relapsed within 12 months of receiving platinum-based chemotherapy, thus likely to represent drug resistant relapse. Six of these women had also previously received taxanes. Disease was assessed with fortnightly CA125 and CT scanning of measurable disease at 12 weeks. The main dose limiting toxicities were Gl toxicity and neutropenia. No patients required cessation of treatment due to toxicity. There were 7 delays in treatment due to grade III neutropaenia, no grade IV neutropenia was seen. 1 patient had progressive disease during treatment. 3 patients died during treatment. 2 succumbed to non-neutropenic pneumonia and 1 patient developed a pulmonary embolus. 3 patients with bulky intra-abdominal recurrence prior to starting chemotherapy developed DVTs requiring anti-coagulation. 4 patients have had radiological evidence of partial response.

**Conclusions** In this small phase II study of weekly Taxol and Etopophos, the regimen is well tolerated and responses have been seen in patients with probable drug-resistant relapse. Weekly scheduling of taxanes may improve their efficacy particularly in cases of drug-resistance. Recruitment to this study continues.

### P203 PACLITAXEL AND CARBOPLATIN DOSE-INTENSITY AND DURATION OF INFUSION IN THE TREATMENT OF OVARIAN CANCER

AV Boddy, MJ Griffin, JG Wright, JA Sludden, HD Thomas, K Fishwick, R Plummer, M Highley, AH Calvert, Cancer Research Unit, University of Newcastle upon Tyne, UK

The combination of carboplatin and paclitaxel is an active treatment for ovarian cancer. While different infusion durations have been studied for paclitaxel, the pharmacological and clinical effects of shorter infusions have not been explored thoroughly.

Eighteen patients with ovarian cancer were treated on a study where the dose of carboplatin was determined by GFR, to attain a target AUC or 6 or 7 mg/ml.min. Paclitaxel dose was 175 or 200 mg/m$^2$, administered over 1 hour or 3 hours. The order of treatment was randomized at the first course, crossing over to the alternative duration of infusion for the second course. Blood samples were analyzed for carboplatin, paclitaxel and for ethanol and Cremophor.

Overall the three-weekly schedule of administration of the combination of carboplatin and paclitaxel was well-tolerated. Also, there was no clinical difference in the toxicities observed between courses where a 1 h infusion was used, compared to a 3 h infusion. Pharmacokinetics of carboplatin indicated that the target AUC was achieved (mean ± standard deviation 114±20% of target) in the majority of cases. Analysis of paclitaxel pharmacokinetics did not show a difference in the AUC or time above a pharmacological threshold for the two infusion durations. The peak concentration of drug obtained at the end of the infusion (9.1 vs 4.5 µg/ml), and the plasma ethanol concentration (40.0 vs 20.5 mg/dl) were higher following the shorter duration infusion. Peak concentrations of Cremophor were not different between the two infusion durations.

These data indicate that the combination of paclitaxel at a dose of 175 mg/m$^2$ and carboplatin at a target AUC of 6–7 mg/ml.min can safely be administered every 3 weeks. Also, paclitaxel can be given as a 1 h infusion with no clinical disadvantage over a 3 h infusion and these durations of administration are pharmacologically equivalent.

### P204 PACLITAXEL IN PLATINUM-RESISTANT EPITHELIAL OVARIAN CARCINOMA

AM Wardley*, A Jenkins, DL Alison, and TJ Perren, ICRF Dept. of Medical Oncology, St. James' Hospital, Beckett Street, Leeds LS9 7TF, UK

Platinum based regimens continue to be the mainstay for treatment of epithelial ovarian carcinoma, and treatment of platinum-resistant disease typically results in low response rates and short survival. First-line treatment with Paclitaxel/platinum combinations has improved response rates and progression free survival. We performed a retrospective analysis of the use of paclitaxel in 97 patients treated with second-line paclitaxel at a single large cancer centre. Of these, 85 patients had progressive disease on or within 3 months of platinum-based chemotherapy. Eleven (13%) patients died before response could be assessed. There were no complete and 19 (22.5%) partial responses, 23 (27%) with stable and 32 (37.5%) with progressive disease. The median survival was 8 (95% CI, 4.75–11.5) months and the progression free survival was 2 (1.2–3.1) months. Patients aged 40 years or less had significantly worse progression free survival and overall survival than older-patients. There was no difference according to number of previous courses of platinum-based chemotherapy, but patients who had received up to 2 previous chemotherapy regimens in total had PFS 1.5 (1.3–1.75) compared to 4.4 (2.6–6.2) months ($p < .01$), for patients who had received ≥ 3 previous chemotherapy regimens. The progression free survival of patients with responsive disease (5.75 ± 0.25 months) was significantly greater than those with stable disease (4.5 ± 0.5 months, $p < .005$), and both of these groups had a highly significant improvement in PFS compared to patients with progressive disease (1.3 ± 0.1 months; $p < .0000$). The overall survival of these groups was 18.8 ± 2.6, 12 ± 1.4 and 5.1 ± 1 months ($p < .0000$, log rank). There is a clear survival advantage in patients who respond to paclitaxel in terms of progression free and overall survival. There appear to be a group of patients whose disease is more responsive to chemotherapy. Paclitaxel is not a panacea for all patients with platinum-resistant disease and there remains a need to find alternative treatments.

### P205 THE *EX VIVO* EFFECT OF HIGH CONCENTRATIONS OF DOXORUBICIN ON RECURRENT OVARIAN CARCINOMA

MH Neale[1], A Lamont[2], IA Cree*[1], [1]Dept of Pathology, Institute of Ophthalmology, Bath Street, London EC1V 9EL, [2]Dept of Radiotherapy & Oncology; Southend Hospital; Westcliff-on-Sea; Essex SSO ORY, UK

The toxicity of anthracyclines has largely prevented dose intensification, but the use of liposomal preparations allows much higher intra-tumoural concentrations to be achieved. However, it is uncertain how much this will improve response rates over standard dose single agent anthracycline therapy. The ATP-based chemosensitivity assay (ATP-TCA) has been used to develop new regimens for several tumour types, to investigate the molecular basis of chemosenstivity, and shows considerable promise as a clinical method for individualising chemotherapy (1).

In this study, we have used the ATP-TCA to determine the concentration responsiveness of tumour-derived cells to concentrations of doxorubicin achievable with liposomal preparations. The 21 tumour samples included were obtained from 19 heavily pre-treated patients with recurrent ovarian cancer, and seven had previous anthracycline exposure, four as part of the CAP regimen.

The results show >95% inhibition at clinically achievable concentrations in 12/21 tumours tested. Of the rest, 7 showed a plateau effect between 80 and 95% inhibition, suggesting that there might be a subset of resistant cells present which could not be killed by high concentrations of doxorubicin. Two tumours showed complete resistance: neither of these had received previously anthracycline therapy. As it has been suggested that gemcitabine might enhance anthracycline sensitivity in combination, and we have had good results with gemcitabine modulation of alkylating agents in the assay (2), we have tested the combination of doxorubicin + gemcitabine under assay conditions in 11 tumours with little indication of improvement.

In conclusion, doxorubicin at concentrations achievable with liposomal preparations shows strong *ex vivo* activity against pre-treated recurrent ovarian cancer in just over half of the cases tested.

1 Cree IA, Kurbacher CM (1997) *Anti-Cancer Drugs* **8**: 541–8
2 Neale MH, Myatt N, Cree IA, Kurbacher CM, Foss AJE, Hungerford JL, Plowman PN (1999) *Br J Cancer* **79**: 1487–93

### P206 OUTPATIENT CHEMO-RADIOTHERAPY FOR CERVIX CANCER USING LOW DOSE CISPLATIN – PRELIMINARY RESULTS OF A MULTICENTRE LGOG STUDY

MEB Powell[1] & PR Blake[2], on behalf of the London Gynae-Oncology Group, [1]Dept of Clinical Oncology Barts & TheLondon NHS Trust, [2]Dept of Gynae-Oncology Royal Marsden Hospital NHS Trust

**Introduction** In 1999 the National Cancer Institute issued a rare clinical announcement stating strong consideration should be given to adding concurrent cisplatin based chemotherapy to radiotherapy for cervix cancer. This was prompted by abstracts describing five phase 3 studies (four have now appeared as peer reviewed papers) using chemoradiation and showing a reduction in risk of death from cervix cancer by 30–50%. A criticism of these trials is the sub-optimal delivery of radiotherapy, with median treatment times of 50, 58, 63 and 63 days. It is well recognised that protracted treatment times can reduce local control rates and based on this, most UK clinicians aim to complete treatment in less than 50 days.

**Aims** This study aimed to evaluate whether chemoradiation could be safely incorporated into UK style shorter radiation schedules.

**Methods** Any patient with cervix cancer suitable for radical radiotherapy, with a GFR>50 ml/min, performance status ≤2. Pelvic radiotherapy to a dose of 45–50 Gy in 25–28 fractions was prescribed, followed by intracavitary brachytherapy delivering a total dose to point A of 70–80 Gy. Cisplatin chemotherapy was given as an outpatient either twice weekly (20 mg/m$^2$) or once weekly (40 mg/m$^2$). Toxicity was measured using WHO criteria and response initially assessed clinically and radiologically at three months post treatment. Follow-up was 3 monthly thereafter.

**Results** Since April 1998 over 50 patients with a median age of 50 (range 28–72) have been treated. Some degree of neutropaenia occurred in 70% of patients with 27% showing grade 3) or severe (grade 4) effects. Thrombocytopaenia occurred in 12%. Median duration of treatment was 48 days (range 39–69). Treatment delays, mainly due to neutropaenia, were necessary in 11 out of the first 20 patients treated. This led to a change from twice weekly to once weekly cisplatin and a recommendation to use white cell growth factors. Subsequently only 2 patients have needed a delay in treatment. Preliminary data on response will be presented.

**Conclusion** Chemoradiotherapy using a weekly outpatient cisplatin containing regimen is feasible to deliver and well tolerated by patients. Significant haematological toxicity is a problem and without the use of white cell growth factors could lead to unacceptable protraction of treatment which could ultimately compromise cure.

### P207 CHEMO-RADIOTHERAPY FOR CARCINOMA OF THE CERVIX – THE ADDENBROOKE'S EXPERIENCE

SG Russell, LA Burgess and LT Tan, Oncology Centre, Addenbrooke's Hospital, Cambridge CB2 2QQ, UK

**Introduction** Cisplatin-based chemo-radiotherapy (chemo-RT) has been advocated as the treatment of choice for patients with advanced carcinoma (Ca) of cervix with a survival advantage of 10–17% compared to radiotherapy (RT) alone. However acute toxicity is a serious concern and may lead to delays in RT which could offset any benefit from the addition of chemotherapy. This prospective audit assesses the acute toxicity, and subsequent delays in RT, of chemo-RT for Ca cervix at Addenbrooke's Oncology Centre.

**Methods** Since May 1999, 25 patients have received chemo-RT for advanced Ca cervix. The median age was 50 (range=32–69) years. The stage distribution was as follows: Ib = 7, IIa = 2, IIb = 8, IIIb = 6 and IVa = 1.2 patients had positive pelvic nodes. Patients received 45–50 Gy in 25 daily fractions to the pelvic using opposed anterior and posterior fields (8/25 = 32%) or a 3/4 field brick technique (17/25 = 68%). Chemotherapy consisted of single agent cisplatin 40 mg/m$^2$ given at weekly intervals during external beam RT. On completion of external beam RT, all patients proceeded to a single brachytherapy (BT) insertion to a total dose of 65–75 Gy to point "A" at a dose rate of 1.07–1.48 Gy/hr.

**Results** 17 patients (68%) had gastrointestinal (GI) toxicity during chemo-RT. Urological toxicity occurred in 3 patients (12%), haematological toxicity in 7 (28%) while 14 patients (56%) complained of general malaise. 1 patient with hydronephrosis had a temporary deterioration in renal function which improved after further chemo-RT. Only 1 patient had Grade 3 or higher toxicity of any system – she developed neutropaenic pyrexia following her second cycle of chemotherapy. 17 patients (76%) completed the prescribed course of 5 chemotherapy cycles. In the remaining 8 patients, 1–3 cycles of chemotherapy were omitted for GI-toxicity (4), deteriorating renal function (1) and visual disturbance (2). 1 patient died of intra-abdominal thrombosis during the second week of chemo-RT. Another patient sustained a massive pulmonary embolus within days of her BT treatment. In both patients, chemotherapy was not considered a significant contributory factor to death. None of the 24 patients who completed the prescribed course of external beam RT experienced any delays in RT.

**Conclusions** In our experience, chemo-RT for Ca cervix is reasonably well tolerated and does not result in delays in RT. We believe that the lack of RT delays in our patients was achieved by the vigilance of the gynaecology oncology team and early action, including delay/omission of chemotherapy, in patients experiencing toxicity.

### P208 A STUDY OF THE SAFETY OF INCREASED FOLINIC ACID (FA) RESCUE FOR METHOTREXATE (MTX) TOXICITY IN PATIENTS WITH PERSISTENT TROPHOBLASTIC DISEASE (PTD)

R Agarwal*, S Strickland, M Foskett, IA McNeish, ES Newlands and MJ Seckl, Dept. of Medical Oncology, Charing Cross Hospital, London W6 8RF, UK

**Introduction** MTX (50 mg IM) on days 1, 3, 5 and 7 with FA rescue (7.5 mg PO, 30 hours post MTX) on days 2, 4, 6 and 8 (MTX-FA)[1] is the standard treatment for low and medium risk PTD, at Charing Cross Hospital (CXH). Although well tolerated, some patients develop significant mucositis which may result in treatment delays or a change to another chemotherapy regime. Increasing the dose of FA rescue to 15 mg usually ameliorates mucositis. However, this may potentially increase the subsequent risk of MTX resistance. Here we have explored the safety and efficacy of using an increased dose of FA for rescue in those patients with significant mucositis.

**Method** All patients treated with MTX-FA for PTD at CXH, between 1/1/97 to 1/1/99 were included in the study. Patients who developed more than one mouth ulcer, or an ulcer which failed to heal in time for the next course of treatment, had their FA rescue increased to 15 mg. The response to treatment was monitored with twice weekly serum human chorionic gonadotrophin (hCG) measurements. Patients who developed a plateau or rising hCG on MTX-FA were defined as MTX resistant and were changed to actinomycin D, if the hCG was below 100 iu/l; otherwise they were treated with etoposide, MTX, actinomycin D alternating weekly with cyclophosphamide and vincristine. Since the completion of treatment all patients have been monitored with regular serum and/or urine hCG to detect any relapses.

**Results** A total of 105 patients were followed for an average of 19.2 months from the start of chemotherapy. Increased FA rescue was required in 43% (n=45) of these patients due to mucositis. No other treatment changes in the MTX-FA regime were necessary in either group due to mucositis or other toxicity. The risk of developing MTX resistance was not significantly different between patients receiving 7.5 mg (FA-7.5) or 15 mg FA rescue (FA-15) (38.3% and 37.7% respectively, p=0.95). The relapse rates following chemotherapy were also similar in the two groups (1.7% with FA-7.5 and 2% with FA-15, p=0.84). In addition, the total duration of chemotherapy was not significantly different either (average of 108 days in the FA-7.5 group and 107 days in the FA-15 group, p=0.23).

**Conclusion** The use of 15 mg of FA rescue in patients with mucositis enables the continuation of MTX-FA chemotherapy with minimal morbidity, without compromising the safety or efficacy of the treatment. We currently recommend that all patients being treated with MTX-FA should receive 15 mg folinic acid rescue.

1 Bagshawe KD, Dent J, Newlands ES, Begent RH and Rustin GJS (1989) The role of low-dose methotrexate and folinic acid in gestational trophoblastic tumours (GTT). *Br J Obstet Gynaecol* **96**: 795

### P209 INCREASED ENDOGENOUS *N*-NITROSATION IN THE HUMAN COLON: A RESPONSE TO RED AND WHITE MEAT?

AJ Cross*[1], JRA Pollock[2], SA Bingham[1], [1]MRC Dunn Human Nutrition Unit, Hills Rd, Cambridge CB2 2XY, [2]Pollock and Pool Ltd, Ladbroke Close, Woodley, Reading RG5 4DX, UK

Diets containing high levels of red meat have been associated with an increased risk of sporadic colorectal cancer. One possible mechanism is via elevated levels of faecal apparent total *N*-nitroso compounds (ATNC), which have been shown in response to increased red meat intake (Bingham, 1996, Carcinogenesis, 17, 515). These *N*-nitroso compounds may be DNA-alkylating agents capable of causing mutagenic damage as seen in animal models. This study was designed to elucidate whether this increase in ATNC is unique to red meat or whether white meat produces similar effects.

Five healthy male volunteers were assigned to a randomised sequence of 15-day dietary periods (60 g red meat/day, 420 g red meat/day, 420 g white meat/day) whilst living in a metabolic suite. The diets were isocaloric, low in nitrate and heterocyclic amines and deionised water was used for cooking and drinking.

Faecal samples were analysed on day 14, for ATNC by thermal energy analysis (Pignatelli, 1987, Analyst, 112, 945).

Endogenous production of ATNC was significantly increased (p=0.012) on the high red meat diet (293 ± 25 μg/day) when compared with the low red meat diet (123 ± 25 μg/day). However there were no significant differences (p= 0.370) in faecal ATNC when comparing the high white meat diet (88 ± 18 μg/day) with the low red meat diet.

High levels of white meat do not therefore affect endogenous production of ATNC in the human colon, whereas high red meat diets significantly enhance production of these compounds. The effect of red but not white meat may explain epidemiological associations with cancer risks.

### P210 COLORECTAL MUCOSA ADENOMA FORMATION AND O$^6$-ALKYLGUANINE DNA-ALKYLTRANSFERASE LEVELS: A CASE-CONTROL STUDY

*NP Lees*[*1,2], KL Harrison[1,3], CN Hall[2], GP Margison[1], AC Povey[3], [1]Paterson Institute Manchester M20, [2]Dept of Surgery, Wythenshawe Hospital, Manchester M23, [3]School of Epidemiology and Health Sciences, Medical School, Manchester M13, UK

**Introduction** The promutagenic adduct *O*$^6$-methylguanine is found in the human colon and rectum, and is repaired by *O*$^6$-alkylguanine DNA-alkyltransferase (ATase), inactivating the protein in the process (1). ATase is inducible in experimental animal systems (2). Colorectal ATase activity is lower in those with K-*ras* GC→AT transition mutations in colorectal cancer than in those with colorectal cancer lacking this mutation (3). Very little is known about ATase activity associated with colorectal adenoma formation where K-*ras* mutation may first occur. We sought to investigate ATase activity in the non-neoplastic and adenoma bearing large bowel.

**Methods** With ethical approval and patients' consent, mucosal pinch biopsies were taken adjacent to small (<1 cm) and large (≥1 cm) adenomas found during colonoscopy and from the same bowel segment of age- and sex-matched controls, free of neoplasms during complete colonoscopy. ATase activities of extracts were measured by quantifying the enzyme-dependent transfer of [$H^3$]-labelled methyl groups from calf thymus DNA, an established technique. Matched pairs were assayed simultaneously.

**Results** Comparing mean ATase activity: **1**; in the rectum (n=70), 250±116 fmol/mg protein and in the colon (n=100), 203±120 fmol/mg protein, P<0.01. **2**; in small adenoma cases (n=22), 214±146 fmol/mg protein and in matched controls (n=22), 199±116 fmol/mg protein, p>0.1. **3**; in large adenoma cases (n=63), 250±112 fmol/mg protein and in matched controls (n=63), 207±117 fmol/mg protein, P<0.05.

**Conclusions** This is the first systematic study of ATase activity in benign colorectal neoplasia. There is significantly higher ATase activity in the rectum, the commonest site of large bowel neoplasia, than in the colon. There is evidence of increasing ATase activity in normal colorectal mucosa associated with adenoma progression. Further studies should address whether alkylating, or other, agents cause ATase upregulation in the human large bowel.

Work was supported by a grant from the AICR. The laboratory is supported by CRC.

1 Pegg AE, Dolan ME and Moschel RC (1995) *Prog Nucleic Acid Res Mol Biol* **51**: 167
2 Chinnasamy N, Rafferty JA, Margison GP, O'Connor PJ and Elder RH (1997) *DNA Cell Biol* **16**(4): 493
3 Jackson PE, Hall CN, O'Connor PJ, Cooper DP, Margison GP and Povey AC (1997) *Carcinogenesis* **18**(7): 1299

### P211 METABOLISM AND BIOAVAILABILITY OF THE CHEMOPREVENTIVE AGENT CURCUMIN IN RATS

CR Ireson[1]*, S Orr[3], DJL Jones[1], S Donald[1], MW Williams[2], CK Lim[1], RD Verschoyle[1], WP Steward[2] and A Gescher[1], [1]MRC Toxicology Unit and [2]Department of Oncology, University of Leicester and [3]Human Tissue Bank, De Montford University, Leicester, UK

Curcumin (diferuloylmethane) is the major constituent of the spice turmeric, a yellow pigment that can be isolated from the rhizome of the *Curcuma Longa* plant. Whilst there is substantial evidence for the cancer chemopreventive efficacy of curcumin in animals, little is understood regarding its bioavailability and metabolism. Therefore we studied the disposition of curcumin in rats *in vivo* and in rat and human hepatocytes *in vitro*. Rats received a single dose of curcumin either *via* the *i.v.* (40 and 10 mg/kg) or oral routes (0.5 and 2.5 g/kg). A sensitive HPLC method was developed for the detection of curcumin. Following *i.v.* administration curcumin disappeared within 30 min from the plasma, and after gastric intubation only trace levels of parent compound could be detected. The following metabolites were identified in the plasma following *i.v.* administration using liquid chromatography-electrospray mass spectrometry in the selected ion monitoring mode: tetrahydrocurcumin (THC, m/z=371), hexahydrocurcumin (HHC, m/z=373), hexahydrocurcuminol (HHCO m/z=375), curcumin sulphate (m/z=447) and curcumin glucuronide (m/z=543). Following oral administration only conjugates of curcumin could be detected in the plasma. To confirm the identity of the conjugates, plasma extracts were incubated with either β-glucuronidase or aryl sulphatase. In each case the size of the conjugate peaks was reduced, with a concurrent increase in that of the parent compound. Hepatocytes were isolated from rats and humans and incubated with curcumin (100 μM) for 1–2 hr. Curcumin was metabolised to THC, HHC and HHCO, but only to a very slight degree to its sulphate and glucuronide conjugates. The results suggest that i) the systemic bioavailability of curcumin is poor, ii) the metabolism of curcumin in rat and human hepatocytes is qualitatively similar, and iii) hepatic metabolites are only minor biotransformation products of curcumin *in vivo*. It is possible that the metabolites of curcumin play a role in its pharmacological activity.

### P212 REGULATION OF UNCOUPLING PROTEIN-1 AND -2 GENE EXPRESSION BY A LIPID MOBILIZING FACTOR

ST Russell*[1], MJ Tisdale[1] and C Bing[2], [1]Pharmaceutical Sciences Institute, Aston University, Birmingham B4 7ET, [2]Department of Medicine, University of Liverpool, Liverpool L69 3GA, UK

Loss of adipose tissue is part of the syndrome of cancer cachexia, which occurs in about 50% of all untreated cancer patients. We have recently isolated a lipid mobilizing factor (LMF) from the urine of weight losing patients with cancer, which produces direct triglyceride hydrolysis in isolated adipocytes. *In vivo* studies show specific depletion of carcass lipid with increased oxygen uptake of brown adipose tissue (BAT), providing evidence for both increased lipid mobilization and utilization. In the present study LMF was purified from the urine of cachectic cancer patients by a combination of ion-exchange and hydrophobic interaction chromatography, and injected iv (8 μg protein, b.d) into exbreeder male NMRI mice. A decrease in overall body weight of 11±2% was observed within 48 h, without an effect on food consumption. There was a decreased size of the fat pads and accumulation of fat in the liver. There was a decrease in serum glucose (58%; p<0.01), glycerol (54%; p<0.05) and an increase of (10%) in nonesterified fatty acids. Northern blotting of BAT showed increased UCP-1 mRNA levels in LMF treated mice (+96%; p<0.01) and increased UCP-2 (+146%; p<0.05) mRNA in gastrocnemius muscle. UCP-3 mRNA was also increased in gastrocnemius muscle (+110%) but this did not reach statistical significance. There was a small elevation (+0.6 ± 0.2°C) in body temperature in LMF-treated animals suggesting an increase in thermogenesis through increased UCP-1 activity. The effect of LMF maybe mediated through a beta-3 adrenoceptor, since lipolysis *in vitro* was attenuated by the specific beta-3 antagonist SR59230A and LMF significantly increased cyclic AMP levels in CHO K1 cells transfected with the human beta-3 adrenoceptor. These results suggest that LMF may play an important role in the increase of energy expenditure associated with tumour growth.

### P213 EFFECT OF A TUMOUR PRODUCED LIPID-MOBILISING FACTOR ON ADIPOSE TISSUE AND SKELETAL MUSCLE

Islam-Ali B*, Tisdale MJ, Pharmaceutical Sciences Institute, Aston University, Birmingham B4 7ET, UK

A lipid-mobilising factor (LMF) was purified from the urine of patients with cancer cachexia using a combination of ion-exchange and hydrophobic chromatography. It had the ability to directly induce lipolysis in isolated epididymal murine white adipocytes. In order to study this effect initial studies were carried out using membranes isolated from white adipose tissue taken from NMRI mice bearing an experimental cachexia inducing tumour model, MAC16 adenocarcinoma, known to produce LMF. Immunoblotting of membranes taken from mice with increasing degrees of weight loss showed an upregulation of stimulatory G proteins (Gs) paralled with a downregulation of inhibitory G proteins (Gi). A reversal of this effect was observed in mice treated orally with EPA, a polyunsaturated fatty acid. Human adipose tissue taken from cachectic cancer patients demonstrated an increase expression of Gs with a decrease in Gi. An increase in Gs was noted in white adipose tissue taken from mice administered LMF. These changes are thought to be related to the increased lipid mobilisation seen in cancer cachexia.

LMF also increased protein synthesis *in vitro* using $C_2C_{12}$ myoblast cells in a dose dependent manner. Maximum stimulation in protein synthesis was achieved at 50 μg of protein over a 24 hr period giving an increase of 41% in comparison to control. The observed effects were partially attenuated by pre-treating the cells with propanolol (10 μM) and a polyclonal antibody raised to human Zinc-$\alpha_2$-glycoprotein (Zn$\alpha_2$gp). The increase in protein measured by tritiated phenylalanine incorporation, was attributed to the stimulation of adenylate cyclase (AC). The effect of LMF was attenuated in the presence of the adenylate cyclase inhibitor $MDL_{12330A}$ (20 μM) and the protein kinase A inhibitor H8 (10 μM). Dibutyryl-cAMP (0.5 mM and 1 mM) and forskolin (25 μM) increased protein synthesis and these responses were additive to LMF. Thus the increase in protein synthesis appears to be mediated by AC possibly through a β-adrenergic receptor.

### P214 COPY NUMBER GAIN AT 12Q12–14 MAY BE ASSOCIATED WITH POOR OUTCOME FOLLOWING THE TRANSFORMATION EVENT IN NON-HODGKIN'S LYMPHOMA

RE Hough, JR Goepel, HE Alcock, BW Hancock, PC Lorigan, DW Hammond*, Institute for Cancer Studies, Division of Oncology and Cellular Pathology, University of Sheffield Medical School, Sheffield S10 2RX, UK

Diffuse large B cell lymphoma (DLBL) arising from follicle centre cell lymphoma (FCL) is typically refractory to treatment and median survival is only one year following transformation.

Paired samples of paraffin-embedded FCL and subsequent transformed DLBL from the same patient were obtained in 24 cases. DNA was extracted from tumour cells which had been microdissected from tumour sections. Tumour DNA was compared to same sex reference DNA using comparative genomic hybridisation. Gains on chromosomes 2q, 7p, 12q and 17q and losses on 5p and 8q were present only in transformed DLBL. Gain at 12q12–14 was found in 12 of the 23 transformed DLBL (adequate DNA could not be obtained in one case) and was not seen in the FCL counterpart.

Of the 11 patients not demonstrating gain at 12q12–14 on transformation (Group A), 6 were male and median age at presentation was 55 years. Five patients had stage III-IV disease at diagnosis. Eight of the 12 patients demonstrating gain at 12q12–14 at transformation (Group B) were male. The median age at presentation was 51 years and 7 patients had stage III-IV disease. Mean time from diagnosis to transformation was similar in both cohorts, 60 months for Group A and 56 months for Group B. Median survival following transformation was 35 months for Group A and 23 months for Group B (p.0.05). Salvage therapy was similar in both groups. A non-significant trend was also observed in overall survival with median survival in Group A of 116 months and in Group B of 97 months.

Gain at 12q12–14 was present in 52% patients with histologically proven transformed DLBL. These patients have a trend towards a poorer survival following transformation. Amplification of a gene at this site may be associated with resistance to currently used cytotoxic agents and radiotherapy, contributing to a more aggressive clinical course.

Supported by Yorkshire Cancer Research.

### P215 IDENTIFICATION OF NOVEL REGIONS OF AMPLIFICATION AND DELETION WITHIN MANTLE CELL LYMPHOMA DNA BY COMPARATIVE GENOMIC HYBRIDIZATION

JE Allen*, JR Goepel, RE Hough, S Bottomley, GA Wilson, HE Alcock, EA Vandenberghe, BW Hancock and DW Hammond, Division of Oncology and Cellular Pathology, University of Sheffield, UK

Mantle cell lymphoma (MCL) is a recently classified entity of non-Hodgkin's lymphoma which has an aggressive clinical course despite being previously considered to be low grade. We have used comparative genomic hybridization (CGH) to characterise alterations in the frequency of DNA sequences corresponding to either amplifications or deletions of specific chromosome regions in archival cases of MCL. A series of local cases was compiled and a diagnosis of MCL confirmed by a combination of histological review by an expert pathologist and immunohistochemistry using a cyclin D1 antibody (NCL-CYCLIN D1-GM; Novocastra laboratories). The status of the BCL-1 locus was investigated by FISH and PCR. DNA was extracted from formalin fixed, paraffin embedded biopsy material and used for CGH experiments. The DNA from each case was labelled with SpectrumGreen-dUTP (Vysis) and co-hybridised with DNA from a normal individual labelled with SpectrumRed-dUTP (Vysis) to normal male metaphase target slides. Digital images of target metaphases were analysed using Quips Software (Vysis). Regions of genomic imbalance were identified in all cases examined (n=30). Chromosome regions with amplification in several cases included 3q (21/30), 6p (19/30), 7p (7/30), 7q(8/30), 12p (8/30), 12q (9/30) and 17q11.1q21 (8/30). 19 cases (63%) shared a common region of amplification of 3q28q29 which was highly amplified in 3 cases, suggesting the possible presence of a MCL related oncogene in this region. Chromosome regions with deletions in several cases included 1p13p32 (10/30), 5p13p15.3 (9/30), 6q14q27 (11/30), 8p (7/30), 11q13q23 (8/30) and 13q (18/30). There was a minimal common region of deletion in 10 cases (33%) at 6q25q26. This is of considerable interest, since although deletions of 6q are frequently found in B-cell lymphoma, no MCL specific locus has previously been identified. The 6q25 region may therefore contain a tumour suppressor gene specifically involved in the development of MCL.

This work is supported by Yorkshire Cancer Research.

### P216 DETECTION OF NOVEL TRANSLOCATIONS INVOLVING *BCL 10*, A TUMOUR SUPPRESSOR GENE BY FLUORESCENT IN SITU HYBRIDISATION

Achuthan R*[1], Bell SM[2], Horgan K[1], Markham AF[2], MacLennan KA[3], [1]Department of Surgery, Leeds General Infirmary, Leeds LS1 3EX, [2]Molecular Medicine Unit and ICRF Cancer Medicine Unit, St James's University Hospital, Leeds LS9 7TF, UK

**Aims** *BCL 10* is a novel tumour suppressor gene thought to play an important role in tumour progression in multiple tumour types. This study aims to seek novel translocations of this gene using fluorescent in situ hybridisation (FISH) and to determine its role as a candidate gene in the development of lymphomas.

**Materials and methods** Fluorescent in situ hybridisation (FISH) was performed on metaphases of patients (n = 20) diagnosed with lymphomas using a specific probe for *BCL 10* gene to detect novel translocations. In addition DNA was extracted from archival fresh frozen tissue of these 20 and a further 12 (total=32) patients diagnosed as having lymphoma. DNA was also obtained from three normal nodes as control. We performed single strand conformational polymorphism (SSCP) analysis on these to seek mutations in the *BCL 10* gene. Additionally direct $^{33}$P sequencing was performed on selected cases.

**Results** FISH confirmed a novel translocation t(1;2)(p22;p12) involving the *BCL 10* gene and the kappa immunoglobulin light chain region on chromosome 2 in a case of extra nodal, marginal zone lymphoma (MALT type). Previously changes involving this gene have only been reported to the immunoglobulin heavy chain region. In the remaining 19 patients no other changes were identified involving *BCL 10*. Interestingly we identified 3 cases (3/20) with recurrent breakpoints in the 1p3 region, telomeric to the *BCL 10* gene. This region harbours many candidate tumour suppressor genes suggesting the involvement of one such gene in these cases. These changes are being further characterised.

Analysis for identifying mutations *BCL10* by SSCP revealed band shifts in the exon 1 region of the gene. Direct $^{33}$P sequencing detected polymorphisms in the gene however failed to detect mutations.

**Conclusions** Using FISH we have found a novel translocation involving the *BCL 10* gene in a case of MALT lymphoma. Mutation analysis did not reveal any mutations of this gene at the DNA level and analysis at the post transcriptional level is ongoing. In view of the translocation identified it appears that the role of *BCL 10* may be limited to the pathogenesis of MALT lymphomas. We have incidentally identified recurrent breakpoints in the 1p3 region and this may be of importance as this region contains many candidate tumour suppressor genes. Further work is underway in this direction.

### P217 ISOTYPE SWITCH VARIANTS REVEAL CLONALLY-RELATED SUBPOPULATIONS IN DIFFUSE LARGE B CELL LYMPHOMAS: INSIGHTS INTO THE BIOLOGY OF GERMINAL CENTRE LYMPHOMAS

CH Ottensmeier*[1], BS Wilkins[2], JW Sweetenham[1] and FK Stevenson[3], [1]CRC Wessex Oncology Unit, Dpt of Histopathology[2], Molecular Immunology Group Tenovus[3], Cancer Sciences Division, Southampton Medical School, Southampton SO16 6YD, UK

Primary diffuse large cell lymphomas (DLBCL) are aggressive tumors accounting for ~40% of B-cell malignancies. Their immunoglobulin (Ig) variable region genes of heavy ($V_H$) and light chain have undergone rearrangement and are commonly somatically mutated. The majority of tumours show intraclonal variation indicating that somatic mutation has continued post-transformation. Typically, cells of DLBCL express Ig of a single isotype, but there are often accompanying cells which express alternative isotypes. In order to probe the status of the isotype switch process in DLBCL, we have investigated six cases for tumour-derived constant region transcripts of all isotypes. Using PCR, cloning and sequencing, multiple $V_H$ gene products were analyzed for each case. For each of the immunoglobulin isotypes separate PCRs were then amplified and sequenced in separate assays. Following identification of the tumour related VDJ segments, the presence of the major isotype expected from immunohistochemical analysis was confirmed at the RNA level. A further 3–4 alternative isotypes were revealed in all cases, some of which were also demonstrable by immunohistochemistry. All cases were somatically mutated with intraclonal variation. In two cases, we found clearly distinct patterns of somatic mutation between isotypes, consistent with independent evolution of the tumor sub-populations. There was clustering of mutational patterns into either an IgMD/IgG3/IgA set, or an IgG1/IgA set, indicating that switch to IgA can occur by different routes. We conclude that alternative isotype expression is evident in DLBCL, both at the RNA and protein levels. The pattern of mutation indicates that switching is occurring in subpopulations of the tumor after malignant transformation. The findings support the concept that isotype switching may be a feature of DLBCL and illustrate the germinal centre origin of these tumors. Additionally the findings demonstrate that the malignant cells can respond to mechanisms, active in normal B cells without being able to achieve full differentiation.

### P218 PRIMARY NON-HODGKINS LYMPHOMA (NHL) OF THE GASTRO-INTESTINAL (GI) TRACT – THE SHEFFIELD LYMPHOMA GROUP (SLG) EXPERIENCE (1989–1998)

Koh PK,* Horsman J, Hancock H, Hancock BW, YCR University Department of Clinical Oncology, Weston Park Hospital, Sheffield S10 2SJ, UK

The GI tract is one of the commonest sites of extranodal lymphoma; the concept of MALT (mucosa associated lymphoid tissue) origin and recognition of the role of Helicobacter pylori has revolutionised clinical management. The SLG experience reflects that of most other studies. Primary (Stage $I_E$ $II_E$) GI lymphoma was seen in 71 cases (comprising 45 (63%) stomach, 16 (23%) small intestine and 9 (13%) large intestine) These cases accounted for 26% of all extranodal presentations and 6% of all NHL seen by the group. Clinical presentation was most often (80%) dyspepsia/pain in gastric lymphomas; intestinal lesions presented commonly (70%) with obstructive features. Histopathology review (according to the Revised European American Lymphoma (REAL) classification) showed that MALT lymphoma was the commonest type (54% of all cases, 87% arising in the stomach). Diffuse large B cell lymphoma accounted for 21% of cases, most frequently (60%) arising in the intestine. Treatment was often surgery followed by observation or anti-helicobacter therapy (for MALT tumour) or by combination chemotherapy (for diffuse large 'B' cell lymphomas). Surgery was less often the primary treatment in stomach lymphomas seen later in the study period. The 5 and 10 year survivals were 52% and 45% respectively, comparable to results from other centres. In this small series univariate analysis showed significantly better survival only for Stage I vs II. The survival for primary GI NHL was similar to that for extranodal lymphoma, seen by the SLG, as a whole.

### P219 PROGNOSTIC FACTORS FOR SURVIVAL IN MALIGNANT LYMPHOMA – A RETROSPECTIVE ANALYSIS OF 1198 PATIENTS SEEN BY THE SHEFFIELD LYMPHOMA GROUP (SLG)

SE Low*, J Horsman, C Radstone, H Hancock, BW Hancock, YCR University Department of Clinical Oncology, Western Park Hospital, Sheffield S10 2SJ, UK

Many haematological, biochemical and immunological indices have been considered important as prognostic markers in lymphoma, culminating in prognostic models such as those of the International Prognostic Factors Project for aggressive non-Hodgkin's lymphoma and advanced Hodgkin's disease (N. Eng. J. Med 1993; *329*: 987 and 1998; *339*: 1506, respectively).

We have retrospectively evaluated, by univariate analysis, putative markers that were routinely recorded on patients seen by the SLG between 1970 and 1998. 1198 patients were evaluable for overall survival. Broad histological categories (Hodgkin's Disease and Non-Hodgkin's Grade I and II – according to British National Lymphoma Investigation classification) were used.

p values for Log Rank test for equality of Survival distributions

| | WCC | WCN | WCL | Hb | ESR | LDH | IgM | Alb |
|---|---|---|---|---|---|---|---|---|
| HD | | | * | * | | | * | *** |
| | | | <1.0↓ | <11.5↓ | | | <0.5↓ | <35↓ |
| NHL I | | | ** | * | ** | * | ** | *** |
| | | | <1.0↓ | <11.5↓ | >50↓ | High↓ | Lower↓ | <35↓ |
| NHL II | *** | *** | *** | *** | *** | ** | *** | *** |
| | High↑ | 2.6–5↑ | Lower↓ | Lower↓ | >20↓ | High↓ | <0.5↓ | Lower↓ |

* $p < 0.05$; ** $p < 0.01$; *** $p < 0.001$; HD – Hodgkin's Disease; NHL – Non-Hodgkin's Lymphoma; WCC – Total white cell count; WCL – Lymphocyte count ($\times 10^9$/L); Hb – Haemoglobin (g/dL); ESR – Erythrocyte Sedimentation Rate (mm/Hr); LDH – Lactate dehydrogenase (U/L); IgM – Immunoglobin M (g/L); Alb – Albumin (g/L).

In summary, on univariate analysis, significant adverse prognostic markers for all or some histological categories were low serum albumin, low serum IgM, low haemoglobin, low lymphocyte count, raised ESR, raised LDH and raised white cell count.

### P220 EXPOSURE TO LOW CONCENTRATIONS OF ETOPOSIDE REDUCES THE DIFFERENTIATION AND APOPTOTIC CAPABILITY OF LEUKAEMIC CELL LINES

WM Liu, PR Oakley and SP Joel, ICRF Department of Medical Oncology, St Bartholomew's Hospital, London, UK

The development of secondary leukaemia following treatment with etoposide has been highlighted in a number of studies. The risk of incidence appears to be schedule dependent, although the exact mechanism of this secondary leukaemogenesis has not been fully elucidated. We have investigated the effect of low etoposide concentrations on the ability of cells to initiate apoptosis and undergo cellular differentiation in three leukaemic cell lines.

The incubation of CEM and HL60 cells with a low concentration of etoposide (LC-etoposide: 0.04 μM) had no effect on cell proliferation or cell cycle dynamics. Following a continuous culture with LC-etoposide for 5-days, cells were allowed to recover for one week in drug-free medium, then exposed to 0.3 μM etoposide for 4-days ($IC_{60}$). There was a significant increase in viability (%V) and decrease in apoptosis (%A) compared to cells without the pre-treatment with LC-etoposide (%V: 93.1 ± 2.1% vs. 57.8 ± 5% and %A: 1.0 ± 0.4% vs. 8.5 ± 0.4%; $p < 0.001$), associated with a decrease in bax-protein levels. Additionally, there was an 82% reduction in growth factor-induced haemoglobinisation in K562 cells pre-treated with LC-etoposide compared to K562 cells without (6.5 ± 4.8% vs. 40.1 ± 3.3%; $p < 0.001$). In contrast, the pre-culture of cells with a low cisplatin-concentration did not suppress the cytotoxicity of subsequent exposure to cisplatin. Our data has shown that there was no change in the karyotype in these cells, but a two-fold increase in the number of chromosomal aberrations. Results also revealed that a 3-day-minimum duration of exposure to LC-etoposide was required to induce this suppression in apoptosis and differentiation.

These data confirm that etoposide can induce effects which are maintained in the absence of drug and which subsequently affect apoptosis and differentiation. This cell system may provide a model for the induction of chromosomal changes which can give rise to secondary leukaemias.

### P221 LONG-TERM OTUCOME OF PATIENTS WITH ACUTE LEUKAEMIA

INM Micallef, AZS Rohatiner, M Carter, S Slater, JAL Amess and TA Lister, ICRF Medical Oncology Unit and Dept of Haematology, St. Bartholomew's Hospital, London ECIA 7BE, UK

Between 1972 and 1988, 834 consecutive patients (pts., median age 48 years [yrs.], range 15–81 yrs.) received treatment for newly diagnosed acute leukaemia, with curative intent. In 5 pts. treatment involved an allograft, in 5 pts. high-dose treatment supported by autologous bone marrow transplantation. Patients were seen quarterly for the first 5 yrs., and annually thereafter; only one patient was lost to follow-up. A retrospective analysis was conducted to review outcome in the 101 pts. (12%) who survived more than 10 yrs. from diagnosis (67/630 pts. [11%] acute myelogenous leukaemia [AML], 34/204 pts. [17%] acute lymphoblastic leukaemia [ALL]). With a median follow-up of 16 yrs. (range 10–28 yrs.), 88 of 101 pts. (88/834 of the total, 11%) are still alive. To date, 22 pts. have developed recurrent leukaemia, in 6 (AML 4, ALL 2) after 5 yrs. (6, 8, 8, 9, 11 and 20 yrs. respectively). Seventy-one pts. continue in first, 15 in second and 2 in third remission. Thirteen have died, 3 of leukaemia or of treatment related toxicity, 4 of another malignancy, 3 of heart failure and 3 of unrelated causes. Twenty-six children are known to have been born from 29 pregnancies in 19 pts. (10 men, 9 women). One of the children has a chromosome 15 abnormality and is developmentally delayed, another died shortly after birth with anencephaly and multiple organ abnormalities, one has epilepsy. Eight women were documented to have premature ovarian failure and 3 men had oligo or azoospermia. Twenty-one subsequent malignancies have been observed (haematological 4, skin 3, cervix 3, squamous cell 2, breast 2, gastrointestinal tract 2, lung 2, hydatidiform mole 1, astrocytoma 1, adenocarcinoma 1). Benign meningiomas have occurred in 2 pts.; both had received cranial irradiation (one prophylactic, one both prophylactic and therapeutic for CNS relapse). One additional pt. developed CNS radiation damage. Three pts. (all with ALL) have persistent alopecia. Long-term follow-up of pts. who have survived more than ten years following treatment for acute leukaemia reveals a significant number of other malignancies (21%), late recurrences (6%) and complications following cranial irradiation (4%). It is also clear that there is a persistent small risk of death (3%) from recurrent leukaemia. These data show the value of long follow-up and suggest that patients should not be discharged from the clinic.

### P222 LONG TERM OUTCOME OR PATIENTS WITH INVASIVE THYMOMA

L Vini, N Fersht, RA 'Hern, C Harmer, Dept of Clinical Oncology, Royal Marsden Hospital, Fulham Road, London, SW3 6JJ, UK

**Aim** This is a retrospective study aiming to assess the long term results of treatment and outcome of patients with thymoma.

**Patients** Over the last 30 years, 107 patients with thymoma were treated at the Royal Marsden Hospital. There were 56 men and 51 women with a median age at diagnosis of 47 years (range 5–83 years). Sixty-one patients (58%) presented with chest. Twenty-eight were asymptomatic when a mediastinal mass was found on a routine chest radiograph. In 15 cases the diagnosis was made following thymectomy for myasthenia gravis while in 3 the thymoma was discovered following investigations for red cell aplasia.

**Results** Tumours were staged according to Masaoka staging system: there were 2 stage I, 28 stage II, 32 stage III, 30 stage IVA and 15 stage IVB tumours. Initial management included complete surgical resection in 25 cases, microscopically incomplete resection in 38, surgical debulking in 18 and biopsy only in 26. Post-operative radiotherapy (20–60 Gy) was given to 65 patients and adjuvant chemotherapy to 38. With a median follow-up of 16 years (1–30 years) local recurrence occurred in 48 patients and distant metastases (mainly in bone and lung) in 15. Sixty patients have died (there were 12 deaths of unrelated causes), 27 are alive with no evidence of tumour progression and 17 are alive with disease. Overall 5 and 10-year survival was 55% and 38% respectively. Stage of disease ($p<0.001$), extent of surgery ($p<0.01$) and adjuvant radiotherapy ($p<0.01$) were important prognostic factors for local recurrence and survival.

**Conclusions** Surgical excision (total resection whenever possible) is the standard treatment for thymomas. The effect of post-operative radiotherapy and chemotherapy is not clear due to lack of prospective studies. Radiotherapy, however, seems to be of value in advanced and incompletely resected tumours.

### P223 LATE COMPLICATIONS OF MANTLE RADIOTHERAPY FOR HODGKIN'S LYMPHOMA

A Sibtain*, A Bradshaw, N Shah, PJ Hoskin, Marie Curie Research Wing, Mount Vemon Hospital, Northwood HA6 2RN, UK

**Aim** To identify the rate and type of complications from mantle radiotherapy in the treatment of Hodgkin's Disease.

**Design** A retrospective review of the BNLI database for Hodgkin's Disease patients treated at Mount Vernon Hospital between January 1970 and December 1985.

**Results** In the 141 sequentially registered patients in the database, 39 complications in 28 patients treated to the mantle area or neck were found. 2 of these also received radiotherapy to the para-aortic region. The median age at diagnosis was 37.5 (range 15–66). The median overall survival is 181 months. The treatment was predominantly delivered using a cobalt machine with only 5 patients treated on a 4 MV and 5 MV linear accelerator. Treatment technique was by either direct field alone (12 patients) or a direct field with a posterior mediastinal boost once or twice a week. Three patients were treated with daily opposed fields. The midplane dose was between 30 and 40 Gy, 12 patients receiving 40 Gy. In order to achieve this with low energy beams and this treatment technique, anterior applied doses of over 50 Gy were required in 13 patients.

The complications comprise 18 lung, 6 cardiac, 4 laryngeal, 5 second malignancy, 2 skin, 2 hypothyroidism, and one each of oesophageal stricture and jugular vein thrombosis. 16 of the 28 patients have died, with treatment related events contributing in 8 of these. Two bronchial carcinomas may have been incidental, leaving 6 patients whose death was unequivocally related to treatment complication, a treatment related mortality rate of 4.2%.

The median time of onset of complications from treatment was 89 months (range 8–332 m). The median time to cardiac complications was 143 months (range 67–237 m). The median time to lung complications was 61 months (range 8–303 m). Ten of these developed early lung complications between 8 and 31 months, whereas the other 9 developed late complications between 89 and 303 months after treatment. The second tumours seen were basal cell carcinoma at 90 months, lung carcinomas at 117 and 129 months, bowel carcinoma at 293 months and oesophageal carcinoma at 332 months. All were within the radiation field.

**Conclusion** An overall late complication rate after mantle radiotherapy for Hodgkin's disease of 28% and an absolute treatment related mortality of between 4.2% and 5.7% has been observed in this cohort of patients. This may reflect the use of low energy beams weighted anteriorly with consequent high applied doses. Modern techniques can be expected to produce a much lower treatment complication rate.

